# Overexpression of Grain Amaranth (*Amaranthus hypochondriacus*) AhERF or AhDOF Transcription Factors in *Arabidopsis thaliana* Increases Water Deficit- and Salt-Stress Tolerance, Respectively, via Contrasting Stress-Amelioration Mechanisms

**DOI:** 10.1371/journal.pone.0164280

**Published:** 2016-10-17

**Authors:** Julio A. Massange-Sánchez, Paola A. Palmeros-Suárez, Eduardo Espitia-Rangel, Isaac Rodríguez-Arévalo, Lino Sánchez-Segura, Norma A. Martínez-Gallardo, Fulgencio Alatorre-Cobos, Axel Tiessen, John P. Délano-Frier

**Affiliations:** 1 Centro de Investigación y de Estudios Avanzados del I. P. N., Unidad Irapuato, Km 9.6 del Libramiento Norte Carretera Irapuato-León, C.P. 36821, Irapuato, Gto., México; 2 Laboratorio Nacional de Genómica para la Biodiversidad, Cinvestav Irapuato, Km 9.6 del Libramiento Norte Carretera Irapuato-León, CP 36821, Irapuato, Gto., Mexico; 3 Instituto Nacional de Investigaciones Forestales, Agrícolas y Pecuarias, Km 13.5 Carrretera Los Reyes-Texcoco, C.P. 56250, Coatlinchán Texcoco, Estado de México, México; 4 Conacyt Research Fellow-Colegio de Postgraduados, Campus Campeche. Carretera Haltunchen-Edzna Km 17.5, Sihochac, Champoton, 24450, Campeche, México; 5 Laboratorio de Biología Molecular, Instituto Tecnológico de Tlajomulco, Jalisco, km 10 Carretera a San Miguel Cuyutlán, CP 45640 Tlajomulco de Zúñiga, Jalisco, Mexico; National Taiwan University, TAIWAN

## Abstract

Two grain amaranth transcription factor (TF) genes were overexpressed in Arabidopsis plants. The first, coding for a group VII ethylene response factor TF (i.e., AhERF-VII) conferred tolerance to water-deficit stress (WS) in transgenic Arabidopsis without affecting vegetative or reproductive growth. A significantly lower water-loss rate in detached leaves coupled to a reduced stomatal opening in leaves of plants subjected to WS was associated with this trait. WS tolerance was also associated with an increased antioxidant enzyme activity and the accumulation of putative stress-related secondary metabolites. However, microarray and GO data did not indicate an obvious correlation between WS tolerance, stomatal closure, and abscisic acid (ABA)-related signaling. This scenario suggested that stomatal closure during WS in these plants involved ABA-independent mechanisms, possibly involving reactive oxygen species (ROS). WS tolerance may have also involved other protective processes, such as those employed for methyl glyoxal detoxification. The second, coding for a class A and cluster I DNA binding with one finger TF (i.e., AhDof-AI) provided salt-stress (SS) tolerance with no evident fitness penalties. The lack of an obvious development-related phenotype contrasted with microarray and GO data showing an enrichment of categories and genes related to developmental processes, particularly flowering. SS tolerance also correlated with increased superoxide dismutase activity but not with augmented stomatal closure. Additionally, microarray and GO data indicated that, contrary to AhERF-VII, SS tolerance conferred by AhDof-AI in Arabidopsis involved ABA-dependent and ABA-independent stress amelioration mechanisms.

## Introduction

Grain amaranths, namely *Amaranthus hypochondriacus*, *A*. *cruentus* and *A*. *caudatus*, are C4 dicotyledonous plants that belong to the Amaranthaceae family within the Caryophyllales order [1,2]. They are known to be tolerant to adverse environmental conditions, including drought, poor and/ or saline soils, intense illumination severe defoliation and insect herbivory [1,3,4]. This tolerance has been associated with their C4 photosynthesis [5], a high water use efficiency [6,7], an indeterminate flowering habit, and the ability to develop long tap-roots and an extensive web of lateral roots [5,6,8]. Abiotic stress resistance has also been associated with the accumulation of compatible solutes [9], and the expression of stress-related genes [10–12] and transcription factors [9].

A recent transcriptomic analysis of grain amaranth plants subjected to various (a)biotic stresses revealed that more than 1900 genes are induced in one or more stress conditions [13]. The ongoing characterization of a selected number of these genes has already identified genes with interesting characteristics associated with both growth and stress tolerance, such as the *Ah24* orphan gene and the *AhNF-YC* transcription factor (TF) gene. Both genes were shown to significantly influence growth and increase tolerance to both biotic and abiotic stresses when overexpressed in tobacco and Arabidopsis [14,15].

ERFs belong to the APETALA2 (AP2)/ ERF superfamily, whose members are well known participants in the adaptation to several biotic and abiotic stresses [16], in addition to their involvement in the control of primary and secondary metabolism and of developmental processes [17,18]. Genome-wide analysis have uncovered the abundant representation of *ERF* genes in plants, as reported in Arabidopsis (147), rice (180) and several others [18]. AP2/ ERF transcription factors are characterized by the presence of at least one AP2 DNA binding domain, consisting of ca. 60 amino acid (aa) residues, that is organized into a typical three-dimensional conformation [19]. Based on the number and similarity of this and other DNA binding domains (i.e. the B3 domain), they were initially classified into five subfamilies, predominantly the AP2, related to ABSCISIC ACID INSENSITIVE3 (ABI3)/VIVIPAROUS1 (VP1) (RAV), dehydration responsive element binding proteins (DREB) and ERF subfamilies. The DREB/ ERF subfamilies are conformed mostly by proteins with a single AP2 domain and were initially sub-divided into the DREB (A1-6) and ERF (B1-6) subgroups, respectively [20]. A subsequent study used depurated genomic data from Arabidopsis and rice to reorganize their respective ERF families into 12 and 15 groups, respectively, based on the intron-exon structure of their genes and the presence of additional motifs [21]. Several reports have described the induced expression of ERF genes in response to several abiotic stresses, including excess salt or heat, drought, or low temperatures, in addition to changes in light availability. Moreover, ERF gene expression can be induced in plants subjected simultaneously to more than one stress condition. Additionally, the generation of transgenic plants capable of overexpressing *ERF* genes has been successfully employed to ameliorate the deleterious effects associated to biotic or abiotic stresses [18,22,23]. Based on the above, ERF TFs represent interesting targets for traditional or genetic engineering-assisted breeding of stress tolerance in plants.

Dof (DNA-binding with one finger) domain proteins are plant-specific transcription factors that are widely distributed in the plant kingdom. *In silico* analysis indicates that the number of *Dof* genes in different plant species can vary from 1 to 8 members, in algae, mosses ferns and gymnosperms, to 54 and 78 members in monots and dicots, respectively [24]. In addition, the 30 and 36 *Dof* genes predicted in Arabidopsis and rice were further classified into four major clusters, i.e., A-D, of orthologous genes [25], a system that was also applicable the classification of the 34 Dof TF genes predicted in tomato [26]. Dof TFs are 200-to-400 aa proteins having a complex modular structure characterized by the presence of two major domains, namely a single N-terminal conserved Dof DNA-binding domain and a C-terminal transcriptional regulation domain. The former is composed by 50-to-52 aa and includes a C2C2-type zinc finger motif, whereas the latter is conformed by diverse aa sequences having a highly variable structure that may contain specific protein-protein interaction domains and other regulatory elements. These are believed to act as transcriptional activators or repressors of several plant genes [24,27]. Such organization is consistent with the multiple regulatory functions *in planta* reported for the Dof TFs. These include a high diversity of biological processes unique to plants such as carbon fixation and nitrogen assimilation. Light-mediated gene regulation and phytochrome signaling, including the control of photoperiodic flowering and flower abscission is also mediated by Dof TFs, in addition to responses to phytohormones, seed maturation and germination, including accumulation of seed storage proteins and lipid metabolism in seeds. Likewise, Dof TFs have been reported to control secondary metabolism, and to regulate inter-fascicular cambium formation, vascular tissue development, leaf axial patterning and guard cell-specific gene expression, whereas a recent report described their participation in intercellular protein trafficking [24]. Moreover, the involvement of Dof TFs in the regulation of biotic and abiotic stress responses has been increasingly recognized in various plants species including tomato [26], Chinese cabbage [28], potato [29], carrot [30], and Arabidopsis [31]. Dof TFs participation in biotic stress tolerance has been associated with their ability to interact with cystatin proteinase inhibitors and indirectly, with their proposed regulation of the salicylic acid (SA) response [24].

The objective of this study was to characterize stress responsive ERF and Dof TF genes previously identified in the *A*. *hypochondriacus* transcriptome [13]. Further assays, in grain amaranth, showed that these TFs were responsive not only to salt stress or drought conditions but, also, to jasmonic acid (JA), benzothiadiazole (BTH), a SA analog, or abscisic acid (ABA). Interestingly, the overexpression (OE) of these genes in transgenic Arabidopsis plants conferred contrasting tolerance to abiotic stress, either to salt stress or water-deprivation stress, but not to both. The possible mechanisms underlying the differential abiotic stress tolerance observed in transgenic Arabidopsis are further discussed. Particularly interesting results were the exclusive nuclear localization of these TFs in root cells of Arabidopsis transgenic plants and the apparently ABA-independent regulation of stomatal number and aperture in water-stressed *AhERF* overexpressing plants. In addition, the overexpression of both TFs had a wide-ranging influence on other TFs and stress-related signaling pathways.

## Material and Methods

### Biological Material

Seeds of *Amaranthus hypochondriacus* cv. Revancha were kindly provided by Dr. Eduardo Espitia (INIFAP, México). Seeds were germinated and plantlets grown under controlled conditions of light and temperature, as described previously [13]. *A*. *hypochondriacus* plants were subjected to the diverse treatments described below, 5-to-6 weeks after germination. At this time point, they had reached a height of 17-to-22 cm and had 9-to-15 expanded leaves. Sampling of leaf and roots was performed in all stress assays performed, whereas tissues of diverse origin and age were taken to determine the basal expression of ERF and Dof genes in grain amaranth plants. *Arabidopsis thaliana* ecotype Columbia was used for the generation of homozygous, single-copy overexpressing (OE) transgenic plants required for the functional characterization of these genes (see below). Cultivation procedures for the germination, growth and maintenance of wild type (WT) and transgenic OE *A*. *thaliana* plants under controlled growing conditions were performed as described previously [14,15].

### Full-Length cDNA and Gene Amplification

In order to amplify the full-length *AhERF* and *AhDof* cDNAs, total RNA samples (1 μg) obtained from leaves of untreated and salt-stressed *A*. *hypochondriacus* plants (see below) were reverse-transcribed to generate the first-strand cDNA as described elsewhere [3]. Aliquots of this reaction mix (2 μl) were directly used as template for the PCR reactions in the presence of 100 pmol each of the specific primers (S1 Table), designed on the basis of the partial cDNA sequences obtained from the *A*. *hypochondriacus* transcriptome [13]. The subsequent amplification of the 5’ and 3’ cDNA ends were performed by the RACE (Rapid Amplification of cDNA Ends) procedure using the SMARTer RACE cDNA Amplification Kit (Clontech, Laboratories, Mountain View, CA), according to the manufacturer’s instructions. All PCR amplicons obtained were cloned using the pCR4-TOPO cloning kit (Thermo Fisher Scientific, Waltham, MA USA) and sequenced. The proteins encoded by the predicted open reading frames (ORF) of the *AhERF* and *AhDof* cDNAs were deduced with the aid of the FastPCR 6.0 program (http://en.bio-soft.net/pcr/FastPCR.html). Bioinformatics analyses designed to determine the possible biological functions of the AhERF and AhDof proteins and to search for conserved domains were performed using BlastN, BlastX and specialized Blast NCBI databases (http://blast.ncbi.nlm.nih.gov/), in addition to the Phyre^2^ databases to determine protein structure (http://www.sbg.bio.ic.ac.uk/phyre2/) and protein localization (TargetP 1.1 Server) (http://www.cbs.dtu.dk/services/). The aa alignment of AhERF and AhDof proteins with other members of their respective TF families, was performed with the Geneious program (http://www.geneious.com/), whereas the respective phylogenetic trees were constructed using MEGA Ver. 5.1 [32].

### Stress Treatments in *A*. *hypochondriacus*

Groups of six *A*. *hypochondriacus* plants were subjected to salt and water-deficit stress to confirm the induced expression of the *AhERF* and *AhDof* genes observed *in silico*. The experiments were performed in a growth chamber under controlled conditions of light (photosynthetic photon flux density ≈ 300 μmol m^-2^ s^-1^) and temperature (28°C, 16 h light/ 8 h dark), as described previously [14,15,33]. Leaf and root samples were collected at different after being exposed for different time periods (i. e, 2 to 8 days) to the stress conditions.

Bacterial infection assays were performed as described previously [34]. Phytohormone treatments consisted of applying 2 drops of the following solutions: 9.2 mM MeJA, 0.1 mM ABA or 2 mM of benzothiadiazole (BTH), a SA analogue known to induce the signal transduction pathway conductive to the systemic acquired resistance syndrome [35]. All solutions were prepared in 0.001% ethanol Triton X-100, and were applied to leaf number 5, counted from the bottom of 9-to-15 leaf amaranth plants. Both local (in leaf number 5) and systemic (in leaf number 8 or 9) responses were sampled 1, 3, 6, 12 and 24 h after phytohormone application. Control plants were treated with the 0.001% Triton X-100 solution only and were sampled at the same time points. Root samples was also collected in the water deficit and salt stress experiments performed. The plant tissue samples were flash frozen in liquid N_2_ and stored at -80°C until required for further analysis.

### Gene Expression Analysis by Quantitative Real-Time RT-PCR (qRT-PCR)

For gene expression, pools of plant tissues of the six Arabidopsis or grain amaranth plants employed per experiment per time point, tissue type or development stage were harvested. Each pool was subsequently subjected to three independent sampling procedures prior to analysis. The cDNA employed for the qRT-PCR assays was prepared from 4 mg total RNA. This RNA was diluted 10-fold in sterile deionized-distilled (dd) water prior to qRT-PCR. Amplifications were performed using SYBR Green detection chemistry and run in triplicate in 96-well reaction plates with the CFX96 Real Time System (Bio-Rad, Hercules, CA, USA). Reactions were prepared in a total volume of 20 μl containing 2 μl of template, 2 μl of each amplification primer (at 2 mM), 8 μl of IQ SYBR SuperMix (Bio-Rad) and 6 μl of sterile dd water. Quantitative real-time PCR was performed in triplicate for each sample using the primers listed in S1 Table. The *AhERF* and *AhDof* qPCR primers were designed on the basis of their complete cDNA sequences. Primer design for these genes was performed using the DNA calculator software (Sigma-Aldrich St. Louis, MO, USA) and included part of the unique 3’ non-coding segments of the *AhDof* cDNA, and segments that did not overlap with genes sharing similarity with *AhERF*, to ensure specificity. The protocol followed for all qRT-PCR analysis is described elsewhere [3,14,33].

Relative expression was calculated using the comparative cycle threshold method [36]. Transcript abundance data were normalized against the average transcript abundance of actin (isotig10321) and β-tubulin (isotig 5486) genes [13], or of the *ACT2* and *EF-1α* genes in grain amaranth and Arabidopsis, respectively. The fold change in expression of the target genes in each treatment was calculated using the following equation: 2^−ΔΔCt^, where ΔΔCt = (Ct target gene − average Ct reference genes)_treatment_ − (Ct target gene − average Ct reference genes)_control_. In transgenic Arabidopsis plants, foliar *AhERF* and *AhDof* transgene expression levels were calculated relative to trace background levels in WT plants. The ΔCt method was employed to quantify the relative *AhERF* and *AhDof* gene expression levels in different grain amaranth tissues. These were the following: axilar meristems, intermediate and young leaves, stems of young and mature plants, roots and panicles. Their expression levels were reported in relation to the expression levels in young stems, which were set at a value of 1.0. Relative gene expression was calculated according to the following equation: 2^ΔCt^, where ΔCt = (Ct of reference gene [actin] − Ct of target gene) [37]. Values reported are the mean of three repetitions ± SE of one representative experiment. The qRT-PCR expression analysis was validated in two independent experiments.

### Genetic Transformation of *Arabidopsis thaliana*

The complete ORFs of *AhERF* and *AhDof* were PCR amplified using specific primers as listed in S1 Table. These amplified 848 and 1023 bp fragments, respectively, which were subsequently utilized to generate stable transgenic plants as described previously [14,15]. All further analysis of the transgenic Arabidopsis plants, OE either the *AhERF* (OE-*AhERF*) or the *AhDof* (OE-*AhDof*) genes, were performed using T_2_ lines homozygous for the transgenes.

Seeds of WT controls and homozygous OE transgenic T_2_ Arabidopsis lines differing in transgene dosage of the *AhERF* (EL25, EL2 and EL15) or *AhDof* (DL2, DL31 and DL4) genes, were surface-sterilized and stratified as described above. Germination and growth of transgenic Arabidopsis employed for experimentation was performed as described elsewhere [14]. In these conditions, the life cycle of the Arabidopsis plants from planting to harvest of seeds was ca. 8 weeks. Four week-old plants were used for experimentation.

### Construction of the *GFP*::*Ah24* Fusion Construct and Transformation of *A*. *thaliana*

The coding sequences of the *AhERF* and *AhDof* genes, were fused with the 5’ region of the green fluorescent protein (GFP) driven by the CaMV35 promoter. The constructions were subsequently employed to transform *A*. *thaliana* plants. The above procedures were performed as described previously [14]. For GFP analysis, roots were observed under an inverted LSM510 confocal laser scanning microscope (Zeiss, Oberkochen, Germany). For visualization, seedlings were stained and mounted in 10 μg/ ml propidium iodide (PI) solution (Sigma). The green fluorescent protein (GFP) was excited with the 488 nm laser line of an argon laser, whereas PI was excited with the 514 nm laser line. The resulting images were acquired using the multi-channel mode. For GFP analysis of whole seedlings, a Lumar V.12 stereoscopic microscope with a GFP filter (Carl Zeiss Microscopy GmbH, Jena, Germany) was used.

### Measurement of Abiotic Stress Tolerance and Vegetative Growth in Arabidopsis Plants

After germination on MS plates, 10-day-old seedlings of the above transgenic lines were planted in 500 ml plastic trays containing a previously described soil mixture (see above). The seedlings were placed in the above growth chamber at 22 ± 1°C, 70% humidity, following the programmed long-day cycle (16 h light/ 8 h dark). For water-deficit stress tolerance assays, plants were grown in soil under an optimal watering condition for 3 weeks. Watering was then withheld, and observations were subsequently recorded for 7 d. The reduction in soil water potential as stress progressed was registered. It was very similar to the fall in soil water potential previously reported in comparable experiments [15]. At this stage of the experiments, WT plants exhibited severe dehydration effects. Both WT and transgenic plants were subsequently re-watered. The survival rate was determined one week after watering was re-established. For salt tolerance assays, plants were initially grown in the standard soil mixture, under optimal watering, for 3 weeks. Then, they were irrigated for three consecutive days with 50 mL water solutions containing diminishing salt concentrations. Thus, a 400 mM NaCl salt solution was applied on day 1, followed by the application of 100 mM NaCl salt solutions, on the subsequent 2nd and 3rd days. Following, this acute salt stress phase, all plants were again irrigated with water and maintained under optimal growing conditions. Plants were scored as susceptible to the salt stress applied if they were unable to flower two weeks after adequate watering was restored.

The effect on vegetative growth and seed yield was also determined in all transgenic lines employed in this study. Fresh and dry weights of rosettes, roots and inflorescences were measured in 6–7 week-old plants grown in the standard soil mixture. To determine the effect on yield, dry seeds were harvested manually from fully matured plants, approximately after 8 weeks of growth. All experiments were performed in conditioned growth chambers, as described above.

### Measurement of Osmolytes and Antioxidant Enzyme Activity in Arabidopsis Plants

Several biochemical parameters associated with responses to abiotic stress conditions were determined in leaves of WT and OE *AhERF* and *AhDof* Arabidopsis plants sampled during water-deficit stress, in recovery after re-watering and during the acute salt stress phase. Sampling was performed 6 days after acute water-deficit stress (“water-stress, WS”) and 1 day after watering was restored (“recovery, R”), in OE *AhERF* plants, and at the end of the three-day salt stress regime (“salt stress, SS”), in OE *AhDof* plants. Samples from OE *AhERF* and *AhDof* plants maintained under optimal conditions simultaneously with the stress treatments were used as controls (“optimal, Op”). Non-structural carbohydrates (NSCs) and proline determinations, in addition to the enzyme activity levels of superoxide dismutase (SOD, E.C. 1.15.1.1) and catalase (CAT, E.C. 1.11.1.6), were determined as described previously [15]. Glutathione reductase (GR, E.C. 1.8.1.7) activity was determined following the instructions included in the Glutathione Reductase Assay Kit (Sigma), except that the assay was modified to fit a microplate format. The GR activity assay is based on the generation of reduced glutathione from oxidized glutathione. Activity is measured by determining the coupled reduction of 5, 5´-dithiobis (2-nitrobenzoic acid) to 5´-thio-2-nitrobenzoic acid, at 412 nm.

### Microarray and GO Analysis

Microarray analysis was performed at the Microarray Unit of the Institute of Cellular Physiology of the National Autonomous University of Mexico (UNAM), as described previously [14]. The analyses were performed with rosette leaves of WT and transgenic *AhERF* and *AhDof* OE Arabidopsis plants sampled during WS and R conditions (*AhERF*), or SS conditions (*AhDof*), as mentioned above. They were compared to similar samples obtained in Op conditions. Microarray data was deposited in the GEO repository under accession number GSE77815.

The GO-term enrichment/ impoverishment analysis was performed using PlantGSEA platform [38]. Only differentially expressed genes were used for the GO analysis, whereas the whole repertoire of Arabidopsis’ genes was employed as background.

### Metabolic Profile of Arabidopsis Plant Rosettes

A full spectrum of ionizable metabolites was also measured in methanol extracts obtained from rosette leaves of WT and transgenic *AhERF* and *AhDof* OE Arabidopsis plants sampled during WS and R conditions (for *AhERF*), or SS conditions (for *AhDof*). They were compared to samples obtained in Op conditions, as above. The analysis was performed using direct injection electron spray ionization mass spectrometry (DIESI-MS) as described previously [14,39].

### Morphometric Measurement of Stomatal Aperture

The morphology of the stomata was observed in a digital microscope Keyence VHX5000 (Keyence, Japan), at 1000X of amplification at high magnification (VH-Z500R, Keyence, Japan) and LED illumination. The scanned leaf area was 4.5 mm^2^ while the image sizes were 12549 × 9204 pixels, captured in RGB colour format and stored in tagged image file format (.tiff). The image resolution was 4.83 pixels/ μm^2^. Each image was obtained from one preparation of leaf from each specimen. Regions of interest (ROI) of sample areas were 250 μm (diameter) segments cropped from the original image.

The aperture stomata quantification was analysed from ROI images of leaves with ImageJ v.1.49p software (National Institutes of Health, Bethesda, USA). Descriptors analysed were the length of the stomata or major length (L1) distance between two points of the guard cells and width of the stomata or shorter length (L2) between two points of the guard cells. Three individual ROI areas for each experimental replicas (ten samples) were used for each experiment. The aspect ratio was calculated as R = L1/ L2, [40]. In open stomata, R-values tend to be lower and to have values closer to one, which correspond to the circular shape of the stomata [41]. Closed stomata have an opposed tendency, reaching R-values of around four.

### Statistical Analysis

All statistical analyses of the physiological and biochemical data were done using JMP8 at the α = 0.05 level (SAS Institute Inc., Cary, NC). Data were analyzed using an ANOVA. A Tukey test was performed with each ANOVA. Where pertinent, bars representing mean values and vertical bars representing standard errors (SE) are shown.

## Results

### Isolation of the Full-Length *A*. *hypochondriacus AhERF* and *AhDof* cDNAs and Protein Coding Regions

This study was started using partial cDNA sequences obtained from the 454 pyrosequencing transcriptomic analysis of grain amaranth plants [13]. Isotig 12988, was annotated variously as group VII ATEBP/ ERF72/ RAP2.3 TF [21], whereas isotig 03355, was annotated either as Dof4, in *Glycine max*, or as an OBP4-like zinc finger protein, in *A*. *thaliana*. Both were selected for further study based on *in silico* data showing their induced expression under multiple stress conditions. Recent published genomic and transcriptomic data of *A*. *hypochondriacus* revealed the presence of 100 and 31 ERF and Dof genes, respectively [42]. Of this number, 18 ERF and Dof genes, respectively, shared the multiple stress responsiveness of the two genes reported in this study, and remain as attractive subjects for future studies.

**Fig 1:**
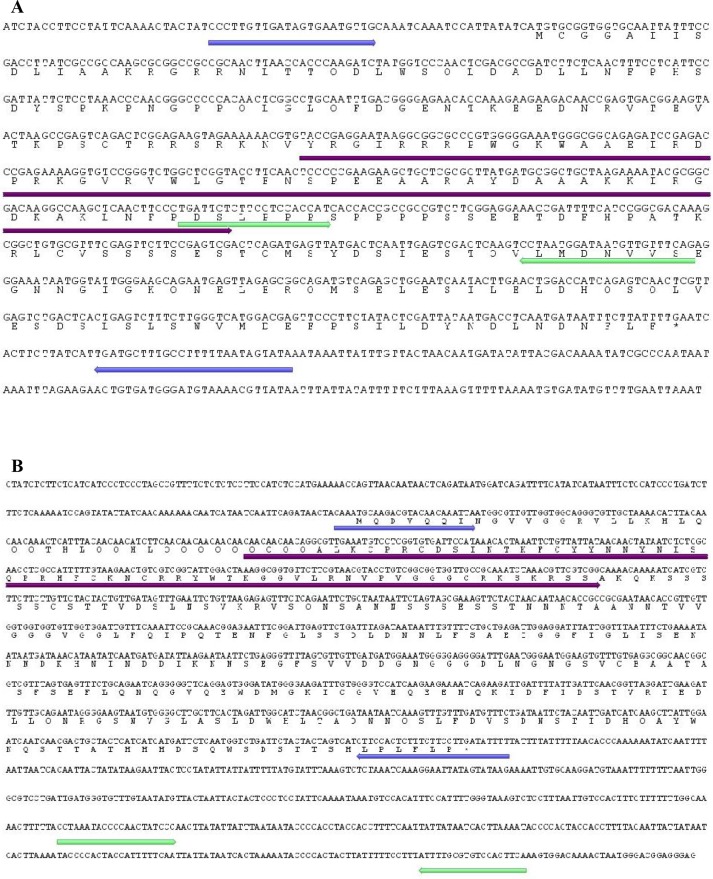
Complete cDNA sequences of the AhERF-VII and AhDOF-AI transcription factor genes and the predicted proteins coded by their open reading frames (ORF). (A) The AhERF-VII cDNA has an ORF of 1,022 bp and encodes a protein of 254 amino acids. (B) The AhDOF-AI cDNA has an ORF of 1,734 bp and encodes a protein of 337 amino acids. The sequences corresponding to the putative AP2 (in A) and Dof zinc finger (in B) DNA-binding domains are underlined in purple. Also shown, in blue and green, are sequence regions selected to design the primers used to isolate their cDNA sequences and to quantify their expression levels by RT-qPCR, respectively.

**Fig 2:**
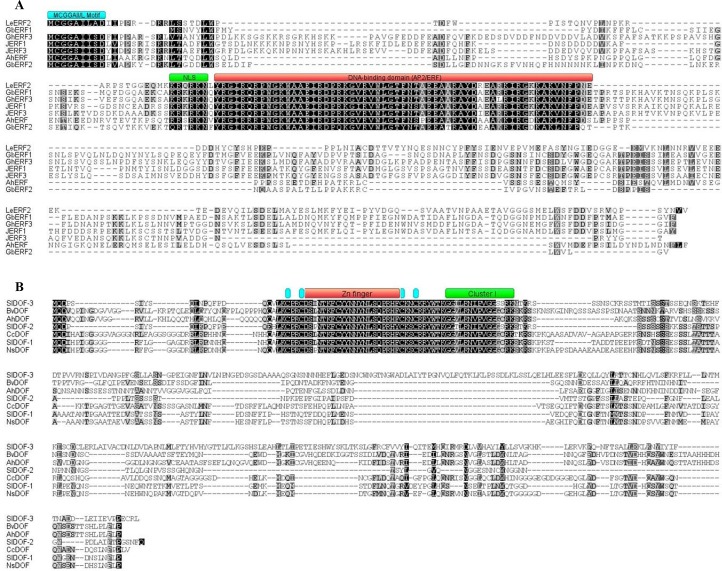
The amino acid alignment of AhERF-VII and AhDOF-AI proteins with other TFs family members from selected plant species. Amino acid residues that are conserved in at least three of the seven sequences are shaded, whereas identical amino acids are shown in black. In (A), the cyan line drawn above the sequences represents the highly conserved N-terminal MCGGAII/L motif of unknown function. A green line was drawn to represent putative nuclear localization signal (NLS), and the red line indicates the conserved DNA-binding domain (or AP2/ ERF domain). In (B) the cyan and red lines show the four regions associated with the zinc finger structure. The green line represents the cluster I conserved motif. Bv = *Beta vulgaris*; Cc = *Cajanus cajan*; Gb = *Gossypium barbadense*; Gh = *Gossypium hirsutum*; JERF = Jasmonate and Ethylene Responsive Factor; Le = *Lycopersicum esculentum*; Ns = *Nicotiana sylvestris*, and Sl = *Solanum lycopersicum*.

**Fig 3:**
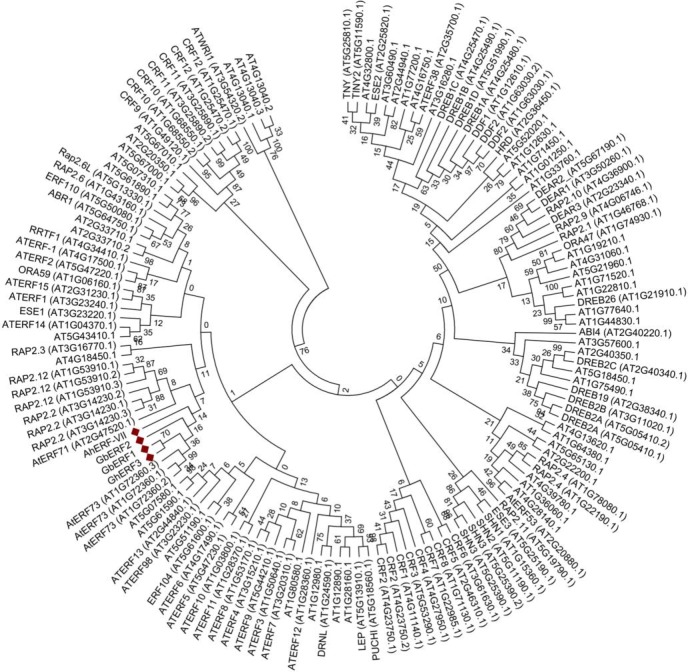
Phylogenetic tree including the AhERF-VII of *A*. *hypochondriacus* together with all the *Arabidopsis thaliana* ERF proteins. Also shown are the highly homologous GbERF1 and GbERF2 proteins from *Gossipum barbadense*, and the GhERF protein from *G*. *hirsutum*. The phylogenetic tree was constructed using the neighbor joining method with amino acid sequence data. It was drawn using the TreeView program, based on alignments obtained using MUSCLE software. The bootstrap values shown are in percent.

The resulting full-length *AhERF* cDNA is 1022 bp long and codes for a predicted 252 aa protein with a molecular mass of 28.6 kDa and an isoelectric point of 5.08 (Fig 1A). Similarly, the *AhDof* cDNA (1734 bp) codes for a predicted 337 aa protein with a molecular mass of 37.0 kDa and an isoelectric point of 5.80 (Fig 1B). The above information fully agreed with recently reported amaranth genomic and transcriptomic data [42]. Further bioinformatics analyses allowed the prediction of their secondary structure and conserved DNA binding domains (S1 Fig). The AhERF protein contains a conserved AP2 /ERF domain of 58 aa and two conserved N-terminal motifs. These correspond to the MCGGAII motif, also known as CMVII-1, which is a characteristic feature of group VII ERF genes [43], and RRSRKN, also known as CMVII-3, which was identified, together with the CMVII-2, in group VII Arabidopsis ERF genes as well [21] (Fig 2A). A phylogenetic analysis of this protein clustered it together with group VII Arabidopsis ERF genes. Thus, this TF will be subsequently referred to as AhERF-VII. The phylogenetic tree also revealed high similarity with two ERF TFs isolated from sea-island cotton plants (*Gossipum barbadense* (GbERF1 and GbERF2) [44], and one (GhERF3) reported in cultivated cotton (*G*. *hirsutum*) (Fig 3).

**Fig 4:**
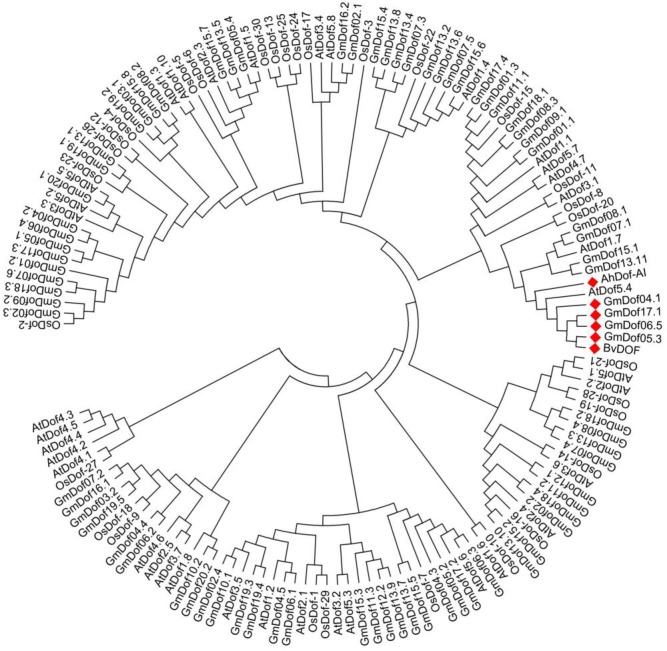
Phylogenetic tree of the AhDOF-AI protein of *A*. *hypochondriacus* together with all Dof domain-containing proteins from soybean (*Glycine max*, GmDof), *Arabidopsis thaliana* (AtDof), and rice (*Oryza sativa*, OsDof). Also shown is a highly homologous Dof protein from *Beta vulgaris* (BvDof). The phylogenetic tree was constructed using the neighbor joining method with amino acid sequence data. It was drawn using the TreeView program, based on alignments obtained using MUSCLE software. The 1050 bootstrap values shown are in percent.

The AhDof protein contained a conserved Zn finger domain of 29 aa and a conserved motif (GGVLRNVPVGGGCRK) that belongs to cluster I of Dof transcription factor proteins [24] (Fig 2B). This represents one of the six major clusters of orthologous and paralogous Dof genes, including the only Dof protein reported in *Chlamydomonas reinhardtii*. The predicted *AhDof* protein can be also assigned to group A, based on the recently reported organization of the Dof domain proteins in soybean, Arabidopsis, and rice [45]. Thus, this TF will be subsequently referred to as AhDof-AI. Phylogenetic analysis revealed that AhDof-AI clustered together with Arabidopsis AtDof5.4, soybean GmDof04.1, Gm05.3, GmDof06.5, and GmDof17.1, and one *Beta vulgaris* BvDof protein. (Fig 4).

### The *AhERF-VII* and *AhDof-AI* Genes Are Induced in Response to Abiotic Stresses and in a Tissue Specific Pattern, in Grain Amaranth

**Fig 5:**
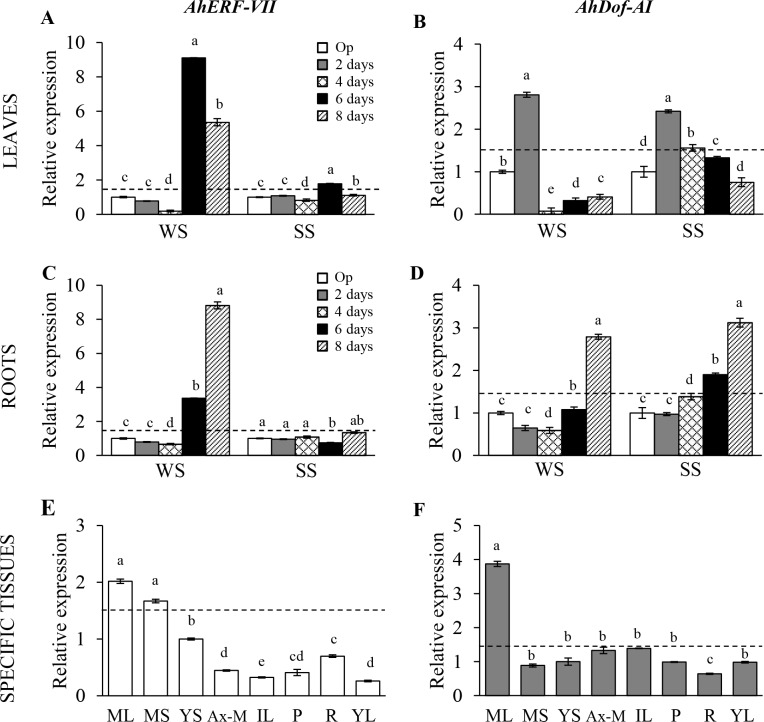
Tissue-specific- and stress-induced expression patterns of the *AhERF-VII* and *AhDOF-AI* transcription factor genes in *A*. *hypochondricus*. Panels (A) and (B), and (C) and (D) show the changes in *AhERF-VII* gene and *AhDOF-AI* expression levels, respectively, in leaves and roots of plants exposed for different time periods (2-to-8 days) to water-deficit (WS) or salinity stress (SS) conditions. The expression levels are relative to those detected in control plants maintained in optimal conditions (Op), whose expression was set to 1.0. Panels (E and F) show the tissue-specific expression levels of the *AhERF-VII* and *AhDof-AI* genes, respectively, measured in relation to those detected in young stems (YS), whose expression levels were set at 1.0. The tissues examined were axilar meristem (AxM), intermediate and young leaves (IL and YL, respectively), panicles (P) and roots (R). Bars and error bars indicate mean values and ES, respectively (n = 6). Different letters over the bars represent statistically significant differences at *P* ≤ 0.05 (Tukey Kramer test). The discontinuous horizontal lines indicate the 1.5-fold threshold value above which gene expression was considered to be induced.

Real time PCR analysis validated previous *in silico* data showing the diverse stress-responsive behavior of these genes [13]. Thus, strong *AhERF-VII* expression levels were detected in leaves (9.1 and 5.4 fold higher than controls) and roots (3.4 and 8.8 fold higher than controls) of grain amaranth plants subjected to WS for 6 and 8 days, respectively (Fig 5A and 5B). Interestingly, *AhERF-VII* expression levels also augmented, in a time-dependent manner (up to 8-fold increase at 8 days post infection), in grain amaranth plants infected with an avirulent *Pseudommonas syringae* pathovar [34], but remained unresponsive to a virulent pathovar (S2 Fig). Conversely, *AhDof-AI* was induced to similar levels of expression in roots and leaves of grain amaranth subjected to both SS and WS. The response in leaves was faster, but temporary (Fig 5C and 5D). The analysis of *AhERF-VII* and *AhDof-AI* expression levels in different tissues of grain amaranth plants indicated that both genes were predominantly expressed in mature leaves, although the *AhDof-AI* expression levels were approximately two-fold higher. *AhERF-VII* expression levels were also high in mature stems (Fig 5E and 5F). The expression levels in all other tissues examined did not vary much from the basal expression level, set as 1.0.

### The *AhERF-VII* and *AhDof-AI* Genes Respond to Phytohormone Treatments in Grain Amaranth

**Fig 6:**
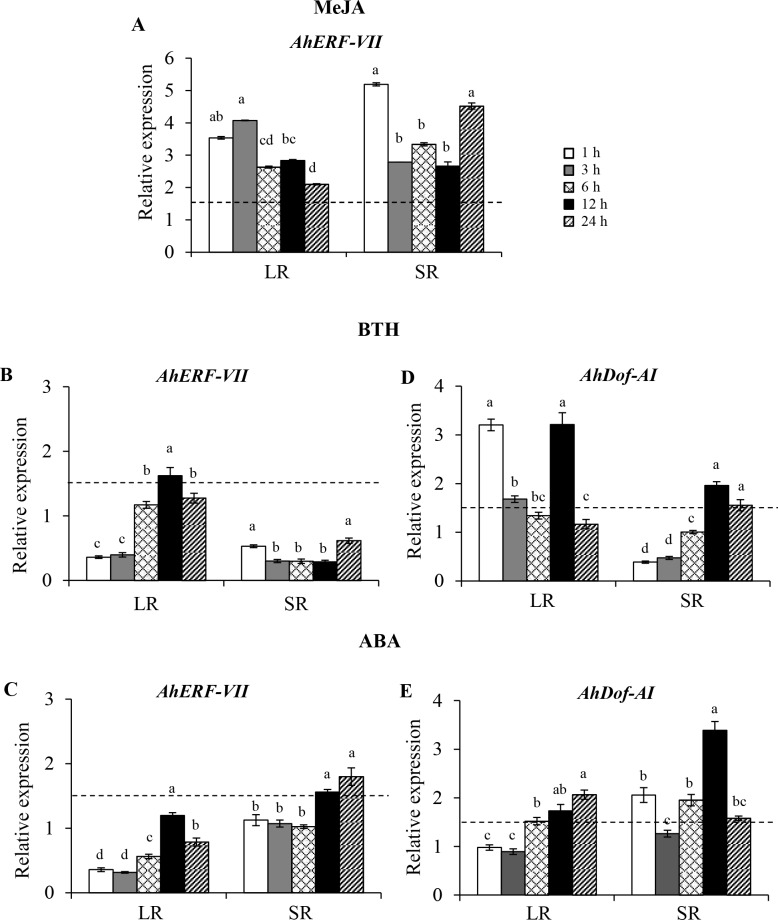
Relative expression levels of the *AhERF-VII* and *AhDOF-AI* transcription factor genes in *A*. *hypochondriacus* produced shortly after the application of exogenous phytohormones or phytohormone-like compounds. The expression of these genes relative to control was measured 1, 3, 6, 12 and 24 h after the application of methyl jasmonate (MeJA, panel A), benzothiadiazole (BTH, panels B and D), and abscisic acid (ABA, panels C and E). The response was measured in treated leaves (local response, LR) and in untreated, distal leaves (systemic response, SR). Bars and error bars indicate mean values and ES, respectively (n = 6). Different letters over the bars represent statistically significant differences at *P* ≤ 0.05 (Tukey Kramer test). The discontinuous horizontal lines indicate the 1.5-fold threshold value above which gene expression was considered to be induced.

The expression of *AhERF-VII* was rapidly induced in leaves of young grain amaranth plants treated with exogenous MeJA. This response was detected at similar levels, both locally and systemically. It also remained stable 24 h after MeJA treatment (Fig 6A). This gene was also strongly induced in response to severe mechanical defoliation in grain amaranth plants in a development-dependent manner (results not shown). ABA and BTH treatments had a weak effect on foliar expression of *AhERF-VII* (Fig 6B and 6C). In contrast, *AhDof-AI* did not respond to MeJA treatment (results not shown), but was induced locally and systemically by both ABA and BTH (Fig 6D and 6E). BTH induced a more rapid and pronounced local response, whereas ABA’s effect was mostly systemic.

### AhERF-VII and AhDof-AI Proteins Are Localized in the Nucleus

**Fig 7:**
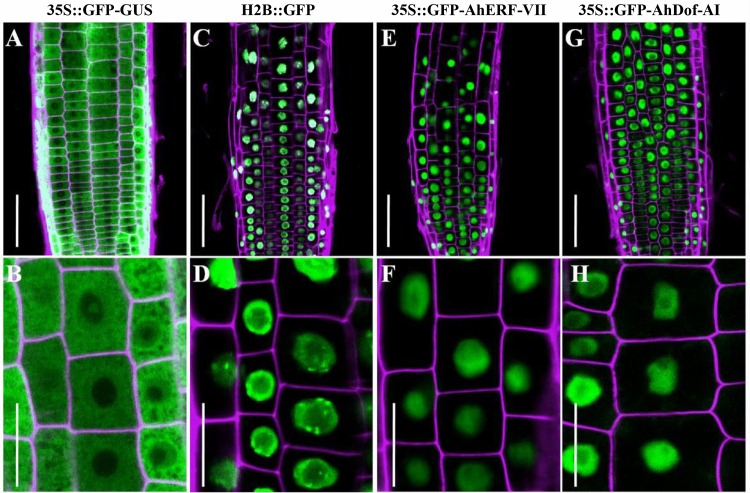
Localization by fluorescence microscopy of the GFP-ERF and GFP-DOF fusion proteins in root cells. A nuclear localization was observed in root cells near the root tip of transgenic Arabidopsis plants constitutively expressing the GFP-AhERF-VII (panels E and F) or the GFP-AhDOF-AI (panels G and H) fusion proteins. The results were compared with those obtained from control transgenic Arabidopsis plants constitutively expressing the 35S:: GFP-GUS (panels A and B) or the H2B:: GFP fusion proteins (panels C and D). The latter overexpress a histone 2B fused with GFP (H2B::GFP), which is a commonly employed nuclear marker.

Bioinformatics tools predicted that AhERF-VII and AhDof-AI might have a nuclear localization. This prediction was confirmed by fluorescence microscopy assays, which detected the predominant localization of the GFP-AhERF-VII and GFP-AhDof-AI chimeric proteins in the nucleus of root cells of the transgenic Arabidopsis plants examined (Fig 7).

### Overexpression of *AhERF-VII* and *AhDof-AI* Increase Tolerance to Drought and Salt Stress, Respectively, in Transgenic Arabidopsis Plants

Four T2 homozygous transgenic lines with a single T-DNA insertion were randomly selected for every *AhERF-VII* and *AhDof-AI* construction. The expression levels of the respective transgenes detected in the transformed OE-*AhERF-VII* and OE-*AhDof-AI* Arabidopsis plants were significantly different, ranging from 1 × 10^3^ to 1 × 10^4^ fold higher, relative to background expression levels in untransformed controls (S3 Fig). Three lines with different expression levels (high, middle and low) were selected for further experimentation. These were lines EL25, EL2 and EL15, and lines DL2, DL31 and DL4 for OE-*AhERF-VII* and OE- *AhDof-AI* transgenic plants, respectively.

**Fig 8:**
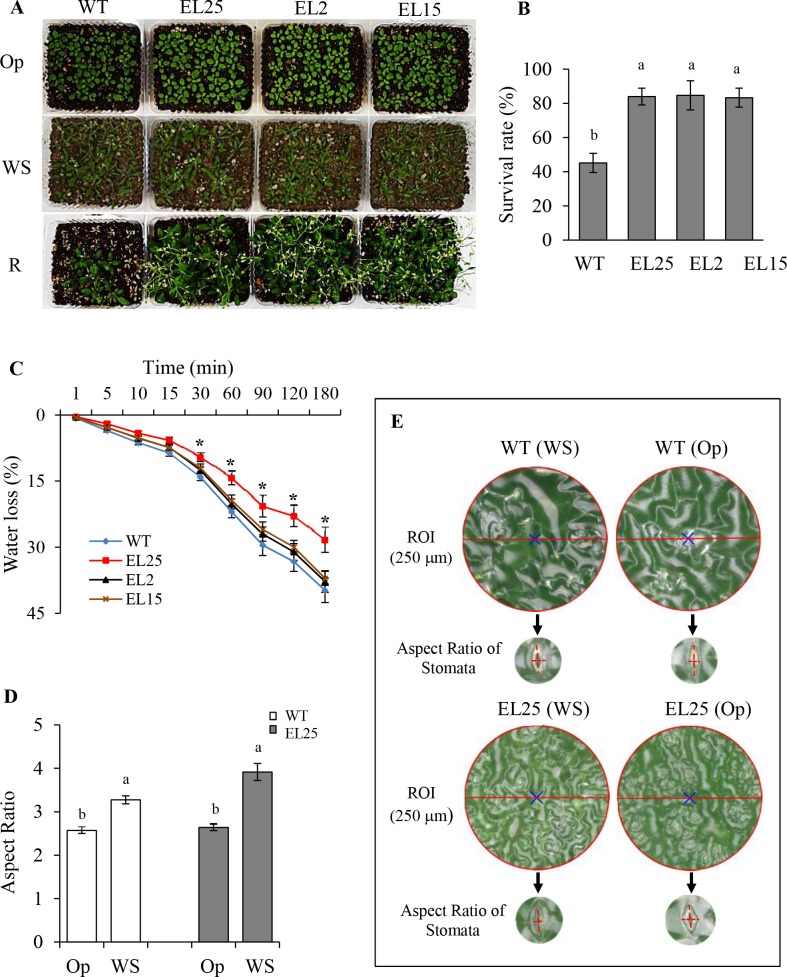
The overexpression of the *AhERF-VII* gene confers water-deficit stress tolerance in transgenic Arabidopsis plants. Panel (A) shows the aspect of WT control plants and of three lines of *AhERF-VII* overexpressing transgenic Arabidopsis plants (EL25, EL2, and EL15) in optimal conditions (Op), after 6 days of water-deficit stress (WS), and one day after watering was resumed to allow recovery (R). In panel (B), the survival rate of the plants shown in A is presented. Panel (C) shows the water loss rate measured in detached rosette leaves taken from transgenic and WT control plants. Panels (D) and (E) show the calculated “Aspect Ratio” parameter employed to quantify stomata aperture, and the regions of interest (ROI) cropped from *A*. *thaliana* leaves, respectively, in WT and transgenic (line EL25) plants in optimal (Op) or water-deficit stress (WS) conditions. Bars and error bars indicate mean values and ES, respectively (n = 25). Asterisks (in C) and different letters over the bars (in B and D) represent statistically significant differences at *P* ≤ 0.05 (Tukey Kramer test). The results shown are those obtained from a representative experiment that was repeated thrice with similar results.

**Fig 9:**
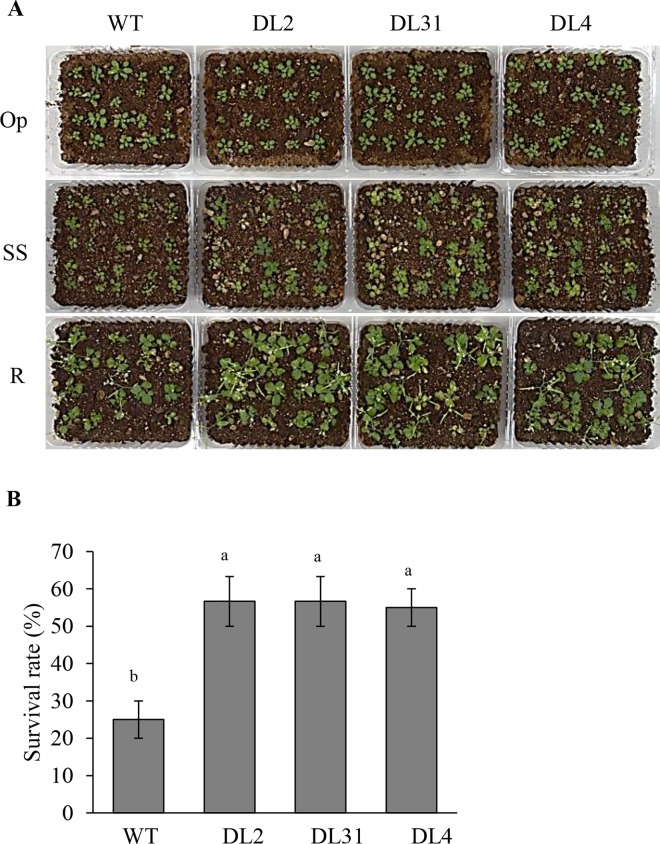
The overexpression of the *AhDOF-AI* gene confers acute salt stress tolerance in transgenic *Arabidopsis* plants. Panel (A) shows the aspect of WT control plants and of three lines of *AhDof-AI* overexpressing transgenic Arabidopsis plants (DL2, DL31, and DL4) in optimal conditions (Op), at the end of acute salt stress (SS), and 2 weeks after watering was resumed to allow recovery (R). In panel (B), the survival rate of the plants shown in (A) is presented. Bars and error bars indicate mean values and ES, respectively (n = 25). Different letters over the bars represent statistically significant differences at *P* ≤ 0.05 (Tukey Kramer test). The results shown are those obtained from a representative experiment that was repeated thrice with similar results.

Tolerance to WS was significantly increased in all three OE*-AhERF-VII* transgenic lines tested, with respect to WT plants. The aspect of the plants in Op conditions, and in the WS and R phases of the experiment is shown (Fig 8A). The survival rate scored in 3-week-old WT plants was only around 45%. In contrast, more than 80% of OE-*AhERF-VII* transgenic plants tested, survived (Fig 8B). WS tolerance, in line EL25, was consistent with a significantly slower rate of water loss in detached rosette leaves as compared with leaves of WT plants (Fig 8C). This was in agreement with the significantly reduced stomatal opening measured on the surface of leaves of transgenic plants undergoing water-deficit stress (Fig 8D and 8E). Conversely, SS tolerance was increased in all OE-*AhDof-AI* transgenic lines tested. The aspect of the plants in Op conditions, and in the SS and R phases, the latter two weeks after watering was restored, is shown (Fig 9A). The recovery rate scored in 3-week-old WT plants was only around 25%. In contrast, more than 50% of the OE-*AhDof-AI* transgenic plants tested, were able to recover and reach the flowering stage (Fig 9B). No increased tolerance to SS or WS was detected in OE-*AhERF-VII* and OE-*AhDof-AI* transgenic plants, respectively (results not shown). Apart from an increased tolerance to certain abiotic stresses, OE-*AhERF-VII* and OE-*AhDof-AI* plants showed normal vegetative and reproductive development (S4 Fig).

The results derived from the biochemical, metabolic and microarray analyses, described below, were performed with the transgenic lines expressing the highest levels of either the *AhERF-VII* (i.e., line EL25) or the *AhDof-AI* (i.e., line DL2) genes (S3 Fig).

### Biochemical Analysis of *OE-AhERF-VII* and *OE-AhDof-AI* Transgenic Arabidopsis Plants under Acute Water-Deprivation or Salt Stress

#### Antioxidant enzymatic activity

**Fig 10:**
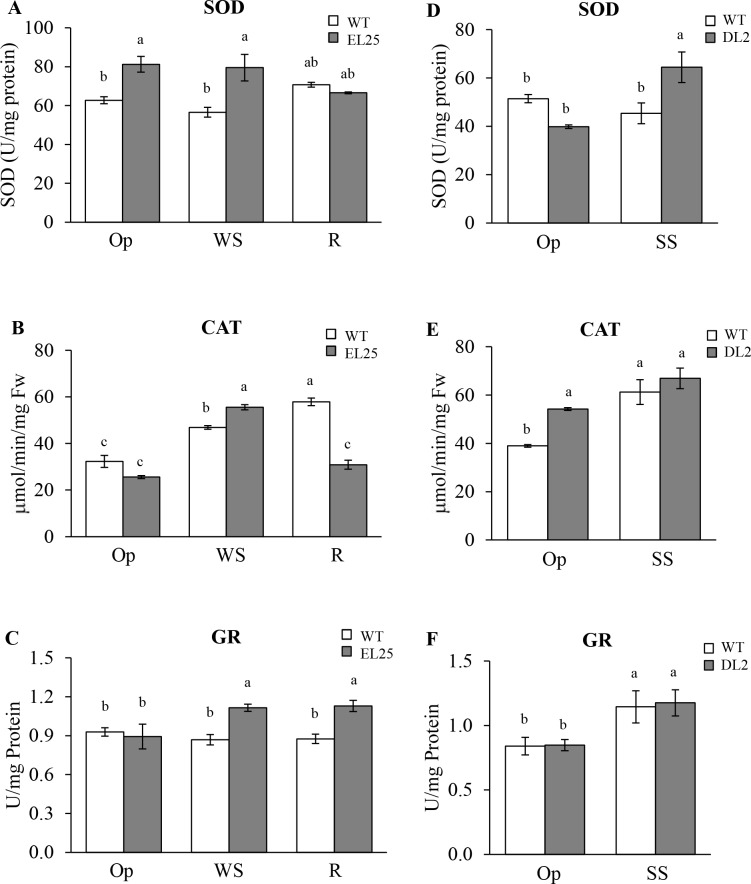
Reactive oxygen species scavenging enzyme activity in transgenic OE-*AhERF-VII* and OE-*AhDof-AI* plants. Activity levels of three reactive oxygen species scavenging enzymes (superoxide dismutase [SOD], catalase [CAT], and glutathione reductase [GR]) and were quantified *in vitro* in leaf extracts of transgenic OE-*AhERF-VII* (line EL25) Arabidopsis plants (panels A-C) growing in optimal conditions (Op), 6 days after water-deficit stress (WS) and 1 day after normal watering was restored (R). Antioxidant enzyme activity was also determined in leaves of transgenic OE-*AhDof-AI* (line DL2) Arabidopsis plants (panels D-F) maintained under optimal growing conditions (Op) or at the end of the acute salt stress (SS) treatment. In each case, gray and empty bars represent transgenic and WT plants, respectively. Different letters over the bars represent statistically significant differences at *P* ≤ 0.05 (Tukey Kramer test). Bars and error bars indicate mean values and ES, respectively (n = 20). The results shown are those obtained from a representative experiment that was repeated thrice with similar results.

SOD activity levels were significantly higher in OE-*AhERF-VII* transgenic plants tested under Op and WS conditions, compared to those recorded in WT plants. In contrast, SOD activity levels recorded in WT and transgenic plants during R did not vary from those detected under optimal conditions (Fig 10A). WS clearly induced CAT activity levels in WT plants, but the inductive effect during WS was significantly stronger in the OE-*AhERF-VII* transgenic plants (Fig 10B). However, inversely to the SOD behavior observed during R conditions, CAT activity levels increased further, in WT plants, and decreased to levels detected under optimal growing conditions, in OE-*AhERF-VII* transgenic plants. GR activity levels did not vary in WT plants during the course of the WS experiments, whereas WS and R conditions led to significantly higher GR activities in OE-*AhERF-VII* transgenic plants (Fig 10C). SOD activity levels were also significantly increased in SS conditions in OE-*AhDof-AI* plants (Fig 10D), whereas both CAT and GR activities reached similar levels in response to SS in both WT and transgenic plants (Fig 10E and 10F). However, CAT activity was significantly higher than WT controls in OE-*AhDof-AI* plants maintained under Op growing conditions (Fig 10E).

#### Proline and soluble NSC accumulation

**Fig 11:**
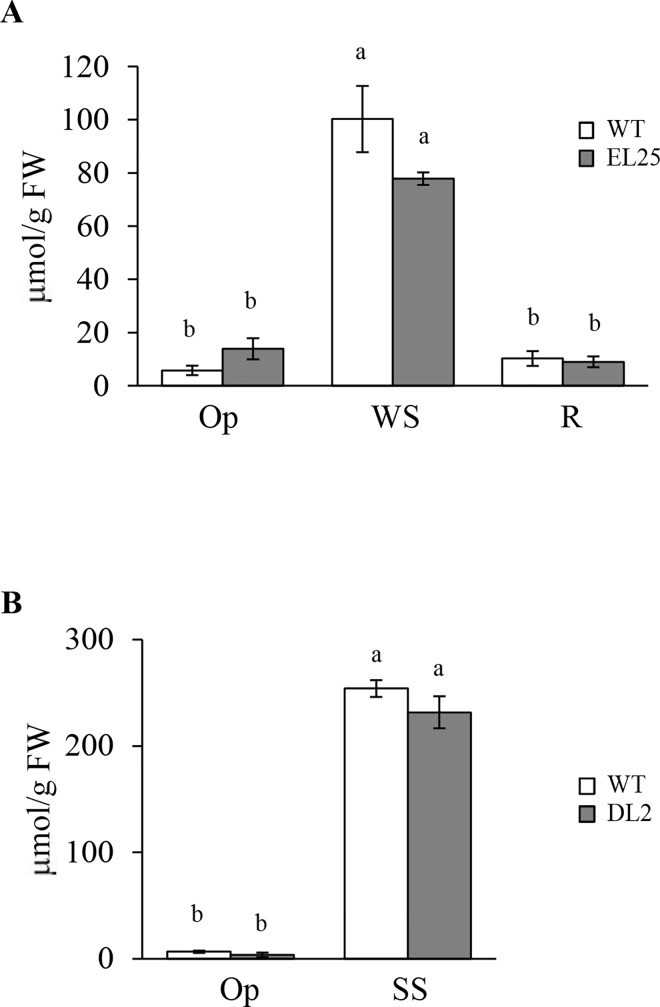
Proline content in transgenic OE-*AhERF-VII* and OE-*AhDof-AI* plants. Proline accumulation levels were quantified *in vitro* in leaf extracts of transgenic *OE-AhERF-VII* (line EL25) Arabidopsis plants (panel A) growing in optimal conditions (Op), 6 days after water-deficit stress (WS) and 1 day after normal watering was restored (R). Proline levels were also determined in leaves of transgenic *OE-AhDof-AI* (line DL2) Arabidopsis plants (panel B) maintained under optimal growing conditions (Op) or at the end of the acute salt stress (SS) treatment. In each case, gray and empty bars represent transgenic and WT plants, respectively. Different letters over the bars represent statistically significant differences at *P* ≤ 0.05 (Tukey Kramer test). Bars and error bars indicate mean values and ES, respectively (n = 20). The results shown are those obtained from a representative experiment that was repeated thrice with similar results.

A highly significant accumulation of proline was detected in response to both WS and SS (Fig 11A and 11B). However, the levels detected in both transgenic plant lines tested did not differ from those detected in WT plants. A similar pattern was observed for most NSCs analyzed, which were also significantly increased under stress conditions to comparable levels, in both WT and transgenic plants (S5 Fig). The only difference observed involved glucose (Glu) levels, which were significantly higher, under Op and WS conditions, in WT compared OE-*AhERF-VII* transgenic plants (S5A Fig). Glu levels were also lower than WT in OE-*AhDof-AI* plants maintained under Op growing conditions. However, in contrast to the above, SS induced a significantly higher Glu accumulation in OE-*AhDof-AI* plants (S5D Fig). Also relevant was the finding that all soluble NSCs levels in OE-*AhERF-VII* transgenic plants decreased to Op conditions levels during the R stage (S5A–S5C Fig).

### Gene Expression Profiles of OE-*AhERF-VII* and OE-*AhDof-AI* Transgenic Arabidopsis Plants

Microarray gene expression profiles were analyzed in WT and transgenic OE-*AhERF-VII* and OE-*AhDof-AI* plants sampled in all stages of the stress experiments performed: i.e., in Op growing conditions, under acute WS or SS, and during R. This was performed in order to discern the possible mechanisms responsible for the increased tolerance to the particular stress condition applied. The fold change analysis (FC = 2.0) showed that the 1237 genes were altered in one or more of the conditions analyzed, representing approximately 5% of the genes on the array having an AGI annotation. Different degrees of overlap between differentially expressed genes were observed in Arabidopsis plants across all treatments and genotypes. The percentage of overlapping genes in all combinations analyzed is shown in S6 Fig. The highest overlap in gene expression patterns in OE-*AhERF-VII* plants was observed between the WS and R conditions (S6C Fig), whereas R and Op conditions were the most dissimilar (S6B Fig). In the OE-*AhDof-AI* plants, shared gene expression in plants under SS and Op conditions was higher than that observed between in OE-*AhERF-VII* plants (S6A and S6D Fig). The overexpression of the *AhERF-VII* and *AhDof-AI* transgenes in Arabidopsis led to similar percentages of shared up- down-regulated genes under both Op and stress conditions (S6E and S6F Fig). A selection of genes whose modified expression was shared in more than one of the conditions tested in this study is shown in Table 1. Also shown is a comparison of genes whose expression was up- or down-regulated under Op growing conditions (S2 and S3 Tables) and under WS (S4 Table) or SS (S5 Table) in OE-*AhERF-VII* and OE-*AhDof-AI* plants, respectively.

**Table 1:** Highly expressed genes detected in both OE-*AhERF-VII* and OE-*AhDOF-AI* Arabidopsis transgenic plants under optimal, stress and recovery conditions.

Gene description	Z score	Gene function	Conditions where induced^1^
***Putative calcium-transporting ATPase (Ca***^***2+***^***ATPases)***	3.7	Ca^2+^ATPases have diverse roles as regulators of many stress signaling pathways, leading to plant growth, development and **salt stress and drought tolerance**.	**OE, D, R, S**
***Nucleotide-sugar transporter family protein***	3.6	Anion transmembrane transport. NAD^+^/ NADH redox control.	**OE, D, R, OD, S**
***N-acetylglucosamine-1-phosphate uridylyltransferase 1*, *GlcNA*.*1UT1***	3.6	This is an essential precursor for **glycolipid and glycoprotein synthesis** and is also used for regulatory protein modification in signaling pathways	**D, S**
***NAC transcription factor family protein (NAC036)***	3.4	NAC proteins enhance drought and salt resistance in transgenic plants. Are also involved in leaf and inflorescence stem morphogenesis.	**D, OD, S**
***Cation/ H***^***+***^ ***antiporter 16 (CHX16)***	3.4	Na^+^/ H^+^ and K^+^/ H^+^ antiporters are involved in intracellular ion and pH regulation in plants. AtNHX1is an important **salt tolerance determinant**; suggested to favor Na^+^ accumulation in vacuoles.	**OE, D, OD**
***Calcium-dependent protein kinase 1 (CDPK1)***	3.1	Over-expression of ginger *CDPK1* gene in tobacco conferred tolerance to **salinity and drought stress**. Positive regulators controlling stress signal transduction in plants.	**OE, D, S**
***Lactoylglutathione lyase*, *GLY1***	3.1	Overexpression of GLY1 confered **salt tolerance in transgenic tobacco plants**.	**D, R, S**
***Responsive to dessication 29A (RD29A)***	3.1	Marker of response to **abiotic stress**.	**D, OD, S**
***Heavy metal transport/detoxification domain-containing protein (K23F3*.*1)***^**2**^	3.0	Involved in heavy metal homeostasis and detoxification mechanisms, especially those involved in cadmium tolerance and transcriptional responses to **cold and drought.**	**OE, D, OD, S**
***Myb-domain transcription factor LOF1 (MYB117)***	3.0	MYB transcription factors are involved in plant development, secondary metabolism, hormone signal transduction, disease resistance and **abiotic stress tolerance.**	**OE, D, OD, S**
***Sugar transport protein 5***	2.9	Cation transmembrane transport. Carbohydrate transmembrane transport activity.	**OE, D, R, OD, S**

^**1**^Optimal conditions, *OE-AhERF-VII* (OE); Water-deficit stress, *OE-AhERF-VII* (D); Recovery (both genes) (R); Optimal *OE-AhDOF-AI* (OD); Salt stress (S), *OE-AhDOF-AI*.

^**2**^de Abreu-Neto JB, Turchetto-Zolet AC, de Oliveira LF, Zanettini MH, Margis-Pinheiro M (2013) Heavy metal-associated isoprenylated plant protein (HIPP): characterization of a family of proteins exclusive to plants. FEBS J. 280:1604–1616.

A gene ontology (GO) analysis of the microarray data obtained from both transgenic plants was also performed (S6 and S7 Tables). Data in S6 Table shows that several biological processes and molecular functions were significantly modified in OE*-AhERF-VII* plants. Highly represented were categories involved in ribonucleobase, and ribonucleobase monophosphate biosynthesis and metabolism, which were mostly downregulated in all conditions tested. Several categories related to development and morphogenesis were enriched under Op and R conditions, but not under WS. Included in Op conditions were those involved in plastid and thylakoid membrane organization, trichome morphogenesis, hair cell differentiation, and root and lateral root development, whereas cell proliferation, differentiation and morphogenesis categories were enriched during R. An evident enrichment in categories involved in reproductive processes was also observed during R. Conversely, leaf and organ senescence were enriched under both Op and WS conditions. WS also led to an enrichment of various categories involved in cell wall and phloem/ xylem biosynthesis and modification. This effect coincided with a reduction of the cell wall macromolecule catabolic process category. In contrast, cell wall-related categories were significantly reduced under Op conditions, and were not significantly represented in R.

An enrichment of categories related to gene silencing and epigenetic control of gene expression, in addition to other categories related to DNA conformational changes, replication and metabolism occurred in Op conditions. Apart from a few exceptions, the above categories ceased to be significantly modified under WS and R.

Various categories involving secondary metabolism were altered in Op conditions. Categories representing proteolytic/ endopeptidase activity, as well as macromolecule catabolic processes were also downregulated in Op conditions. In contrast, only the vitamin biosynthesis and proteolytic activity categories remained downregulated under WS, in addition to those related to fatty acid biosynthesis and oxidative metabolism. Conversely, several categories involving carbohydrate, aldehyde and other catabolic processes of were enriched under WS and R. Additionally, WS led to an enrichment of categories associated with lactate metabolism and protein modification. Flavone, flavonol, cytokinin, branched-chain aa metabolism, primary amine oxidase and chitinase biosynthetic, metabolic and/ or activity processes were exclusively enriched in R. Curiously, transmembrane transporter/ water channel activity categories were only enriched in Op conditions. This contrasted with the downregulation of several other transport related categories involving, among others, vesicle-mediated transport, cation-transporting activity and movement of proteins oligopeptides and peptides, organic acids, and purine nucleobases. Many of these transport-related categories remained significantly reduced under WS and R. Conversely, WS and R were particularly enriched in ATPase activity coupled to transmembrane movement of substances and calcium-transporting ATPase activity categories, respectively. Signaling-related categories that were significantly enriched in Op conditions included many associated with biotic stress responses, including SA biosynthesis and metabolism categories. On the other hand, categories involving inositol monophosphate biosynthesis and metabolism were reduced. These categories remained downregulated under WS, in addition to many other inositol-related categories. WS was further characterized by an enrichment of protein kinase categories, particularly of calmodulin-dependent protein kinases. Also enriched were categories representing methylglyoxal (MG) metabolic and catabolic processes, D-lactate and auxin conjugate metabolic processes, response to monosaccharide stimulus and maintenance of cellular di-, tri-valent inorganic cation homeostasis. In contrast, important stress-associated categories were downregulated under WS, including those involving oxylipin and JA biosynthesis and metabolism, responses to herbivore or to ethylene and polyol biosynthetic processes. Downregulation of the oxylipin and JA categories was also detected during R, although it led to an enrichment of the SA biosynthesis category. The inorganic cation homeostasis category, including iron, also remained enriched during R, in addition to those related to the regulation of hormone levels. In contrast, the R stage became impoverished in important categories related to red light responses and signaling, hydrogen peroxide, and auxin and hormone transport.

Several biological processes and molecular functions were also significantly modified in OE-*AhDof-AI* plants (S7 Table). Similar to OE*-AhERF-VII* plants, an extensive down-regulation of several categories involved in ribonucleotide, ribonucleoside, and ribonucleoside monophosphate biosynthesis was observed under Op conditions, although the tendency decreased noticeably during SS. Likewise, many transport-related categories that were mostly downregulated in Op conditions, had a reduced representation during SS. Included were ion transport categories expected to be increased under SS. In addition, many other categories normally associated with high salinity responses (see below), were also downregulated under SS. Cell wall-related categories were not significantly altered in these plants, except for categories involved in cell wall lignification, which were enriched under Op and SS. A striking effect was the enrichment of several categories related to vegetative and reproductive development under SS. These conditions were also characterized by the singular enrichment of categories associated with posttranscriptional gene silencing, including gene silencing by miRNA. Numerous metabolism-related categories particularly pertaining lipids and steroids, were similarly reduced under both Op and SS conditions, but predominantly in the former conditions. Included were may related to phosphatidylinositol biosynthetic and metabolic processes. Metabolism-related categories that were negatively affected exclusively during SS involved several sugar and branched-chain aa biosynthetic and/or metabolic processes. Conversely, several categories connected to SA response and signaling, systemic acquired resistance, ABA response and signaling, response to 1-aminocyclopropane-1-carboxylic acid and ethylene, and response to and metabolism of reactive oxygen species (ROS) were enriched in Op conditions. In contrast, several categories representing responses to radiation and light, pigment accumulation, stomatal complex morphogenesis, gibberellin catabolism and IAA-aa conjugate hydrolase activities, and responses to temperature and metal ions were under-represented. A striking tendency observed in SS plants was that important (a)biotic-stress related categories, including many directly associated with salt and osmotic stress, such as polyol biosynthetic processes, were downregulated. Another surprising finding was that the stress-related categories enriched during SS included various associated with response to biotic stress, flooding and the concomitant regulation of nitric oxide (NO) metabolism. Of relevance, as well, was that several energy-generation-related categories, which underwent both enrichment (e.g., mitochondrial respiratory chain complex assembly and pentose pathway) or depletion (e.g., carbon fixation, electron transport chain, ATP biosynthetic process, and Calvin cycle) under optimal conditions, ceased to be significant under SS.

### Changes in the Metabolic Profile of OE-*AhERF-VII* and OE-*AhDof-AI* Transgenic Arabidopsis Plants under Drought and Salt Stress

**Fig 12:**
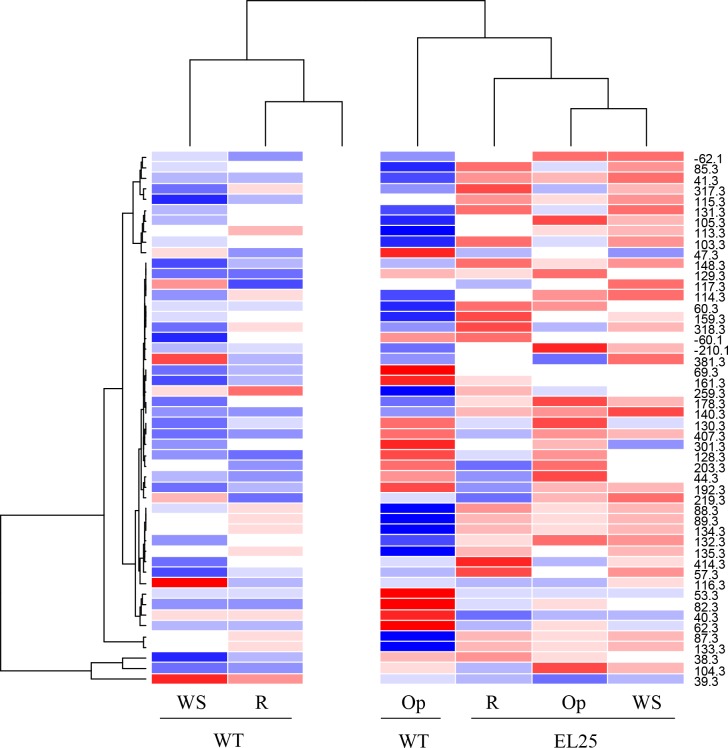
The overexpression of *AhERF-VII* in Arabidopsis modifies its metabolic pattern in optimal growing conditions and in both water-deficit stress and recovery conditions. Metabolic heat map obtained from acidified methanol extracts obtained from leaves collected from wild type (WT) and OE-*AhERF-VII Arabidopsis* plants (line EL25) grown in optimal conditions (Op), or subjected to water stress for 6 d (WS), or allowed to recover from stress, 1 d after normal watering was reestablished (R). The 50 most abundant ionizable metabolites were selected to obtain the metabolic heat-map within a 80–1300 m/z range. The results shown are those obtained from a representative experiment that was repeated thrice with similar results.

The analysis of DIESI-MS metabolic profiles yielded a clear separation of OE-*AhERF-VII* and WT control plants into two distinctive clades (Fig 12), which showed that metabolism was more strongly influenced by the plantʼs genotype than by the experimental conditions. However, *OE-AhERF-VII* clade also included the WT metabolic profile under Op conditions. Nevertheless, a higher ion intensity was detected in the metabolic profiles of OE-*AhERF-VII* transgenic plants compared to those of WT control plants.

**Fig 13:**
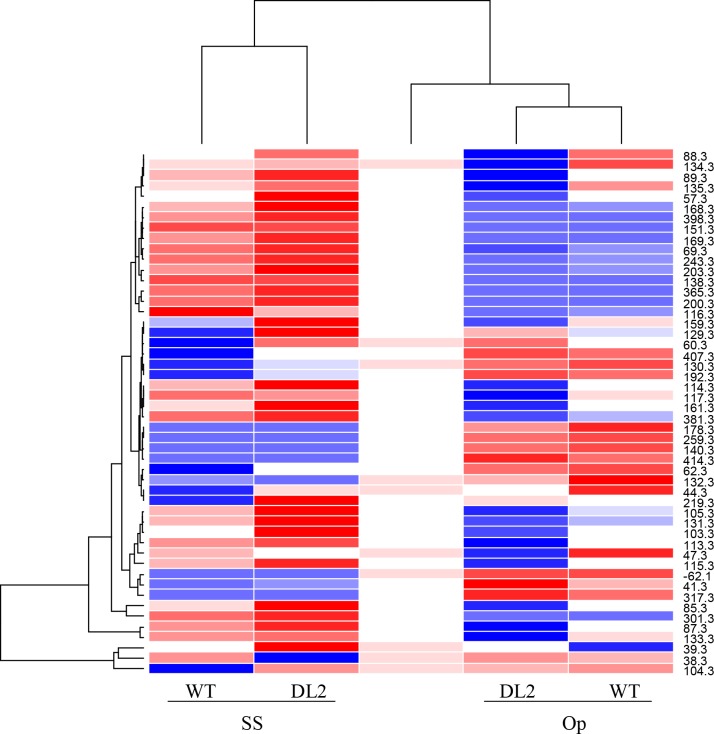
The overexpression of *AhDof-AI* in Arabidopsis modifies its metabolic pattern in optimal growing conditions and under acute salt stress conditions. Metabolic heat map obtained from acidified methanol extracts obtained from leaves collected from wild type (WT) and OE-*AhDof-AI Arabidopsis* plants (line DL2) grown in optimal conditions (Op), or subjected to acute salt stress for 3 d (SS). The 50 most abundant ionizable metabolites were selected to obtain the metabolic heat-map within a 80–1300 m/z range. The results shown are those obtained from a representative experiment that was repeated thrice with similar results.

The metabolic profiles derived from OE-*AhDof-AI* plants also clustered into two well-defined clades (Fig 13). Contrary to the above, clustering in these plants was more influenced by the experimental conditions than by genotype. Ion intensity was also higher in the extracts obtained from transgenic plants subjected to SS.

**Fig 14:**
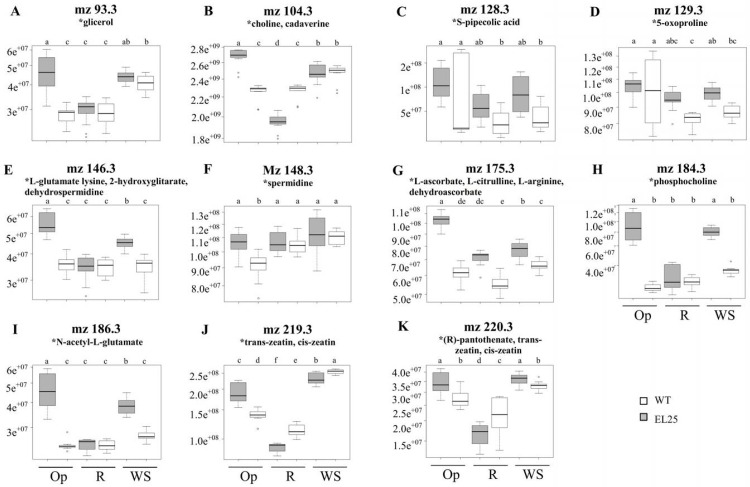
Differential accumulation of water stress-related metabolites in WT and OE-*AhERF-VII* Arabidopsis plants in well-watered conditions and in both water-deficit stress and recovery conditions. The box-plots show the levels (represented as different intensities of the respective m/z ion peaks) of putative (represented as an asterisk) Arabidopsis stress-related secondary metabolites that differentially accumulated in leaves of wild type (WT; empty bars) and transgenic OE-*AhERF-VII* (line EL25; light gray bars) plants. The plants were maintained in optimal conditions (Op), subjected to water-deficit stress for 6 d (WS), or in recovery from stress, 1 d after normal watering was reestablished (R). Each box plot graphically represents groups of numerical data (in this case 10 scans per biological replicate, i.e., n = 30), through their quartiles. The lines extending vertically from the boxes indicate variability outside the upper and lower quartiles, whereas outliers, or atypical values, are plotted as individual points. The upper and lower limits of the box represent 75% and 25% of the data, respectively, while the horizontal bar is the median, representing 50% of the data. Different letters over the bars represent statistically significant differences at *P* ≤ 0.05 (Tukey Kramer test).

**Fig 15:**
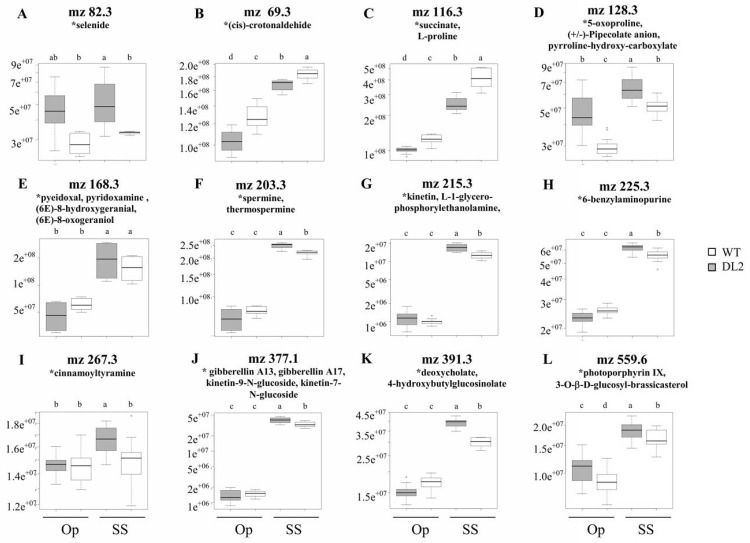
Differential accumulation of water stress-related metabolites in WT and OE-*AhDof-AI* Arabidopsis plants in well-watered conditions and in both water-deficit stress and recovery conditions. The box-plots show the levels (represented as different intensities of the respective m/z ion peaks) of putative (represented as an asterisk) Arabidopsis stress-related secondary metabolites that differentially accumulated in leaves of wild type (WT; empty bars) and transgenic OE-*AhDof-AI* (line DL2; light gray bars) plants. The plants were maintained in optimal conditions (Op), or subjected to acute salt stress for 3 d (SS). Different letters over the bars represent statistically significant differences at *P* ≤ 0.05 (Tukey Kramer test).

An analysis of the most abundant ions showing a significant change in abundance was subsequently performed. The presumed identity of these ions was biased towards those previously reported to have a relation with stress responses (Figs 14 and 15). Notwithstanding its tentative nature, this exercise nevertheless suggested possible metabolic alteration scenarios occurring in the transgenic plants under stress and/ or recovery. Thus, WS caused changes in the abundance of putative metabolites reported to contribute to stress amelioration in other plants, such as glycerol (Fig 14A). Precursors, intermediaries or final products involving betaines (e.g., pipecolic acid, choline; Fig 14B and 14C), proline (e. g., lysine, glutamate, pyrroline-hydroxy-carboxylate, and (S)-2-hydroxyglutarate; Fig 14E) and polyamines (e. g., cadaverine, spermidine, dehydrospermidine, and cadaverine; Fig 14B and 14F) which conform a common biochemical response to stress in plants, were found to be already significantly higher in OE-*AhERF-VII* plants prior WS and/ or R conditions. This tendency was also detected for known antioxidants (e. g., L-ascorbate, monodehydroascorbate or dehydroascorbate; Fig 14G), stress-adaptive phospholipids (e.g., phosphocholine; Fig 14H), and for biochemical markers of diverse abiotic stresses such as citrulline (Fig 14G), 5-oxoproline (Fig 14D), and N-acetyl-L-glutamate (Fig 14I). Moreover, citrulline and arginine are involved in the synthesis of NO, a ubiquitous stress-signal molecule in plants. Some of the above also accumulated in response to SS in OE-*AhDof-AI* plants (Fig 15). In addition, phytohormones or phytohormone-like compounds, such as cytokinins (cis-, trans-zeatin; Fig 14J and 14K; kinetin; kinetin glycosides, 6-benzylamino purine; Fig 15G, 15H and 15J), gibberellins (Fig 15J) and 3-O-β-D-glucosyl-brassicasterol (Fig 15L) were also found to be significantly modified in OE-*AhERF-VII* and/ or OE-*AhDof-AI* plants. This pattern was in agreement with several reports describing their participation in the modulation of several abiotic stress responses. Other metabolites whose levels were exclusively modified in salt-stressed OE-*AhDof-AI* plants were (cis)-crotonaldehyde and proline (both reduced; Fig 15B and 15C). The former is a common decomposition product of lipid hydroperoxides, whereas the latter coincided with the reduced proline levels detected *in vitro* during SS (Fig 11B). In contrast, an increased abundance of putative selenide (Fig 15A), pyridoxal/ pyridoxamine/ (6E)-8-hydroxygeranial/ (6E)-8-oxogeraniol/ (3S)-3-hydroxycyclocitral (Fig 15E), cinnamoyltyramine (Fig 15I), 4-hydroxybutylglucosinolate (Fig 15K) and protoporphyrin IX (Fig 15L), was detected in salt-stressed OE-*AhDof-AI* plants. This tendency was in agreement with the reported influence that cell wall lignification levels have on plant responses to abiotic stresses, as well as certain tetrapyrroles, glucosinolates, water-soluble vitamins, terpenes and essential metals. The relevance of the metabolic changes suggested here is further discussed below.

## Discussion

This report is part of an ongoing effort to characterize grain amaranth genes responsible for their tolerance to adverse environmental conditions. The two genes studied belong to extensive TF families known to regulate stress responses, among other functions, in plants. The AhERF-VII protein was similar to group VII ERF TFs [21]. In Arabidopsis, this group of TFs is involved in the NO-dependent adaptation to low-oxygen stress, although they are also known to be important regulators of other (a)biotic stress responses that involve ethylene, which is a unifying characteristic shared by this large TF family [18,46]. In agreement with the above, *AhERF-VII* was found to be induced under WS conditions in amaranth and to confer WS tolerance to OE-*AhERF-VII* transgenic Arabidopsis plants. It was also induced by exposure to JA, defoliation or avirulent bacterial pathogens in amaranth, which suggests that *AhERF-VII* might be also able to regulate plant responses to wounding and/ or biotic stressors. This possibility is supported, in part, by microarray data showing the up-regulated expression of several defense-related genes and by the observed enrichment of GO categories associated with biotic stress in OE-*AhERF-VII* plants. Many of these are involved in SA-mediated disease resistance (e. g., an eukaryotic aspartyl protease family gene), innate immune response and response to wounding and/ or ethylene (e. g., *THIONIN 2*.*1* and *THAUMATIN-LIKE PROTEIN 1*), positive regulation of plant-type hypersensitive response (e. g., *RPM1 INTERACTING PROTEIN 3*), and suppression of virus induced gene silencing (e. g., *ARGONAUTE 5*). This response is in agreement with ethylene’s capacity to mediate the activation of either biotic or abiotic stress response genes through ERF TFs [18]. Apart from the increased WS tolerance conferred by this transgene, no obvious effect on vegetative or reproductive development was detected in OE-*AhERF-VII* plants. This occurred despite the substantial number of development-related genes and GO categories that were significantly modified in these plants. Relevant changes were the specific downregulation of Dof and NAC TF genes and the upregulation of *ATMYB117* (*LOF1*) and *GATA 23* and other TF genes that control various developmental processes [47–49]. Relevant to the latter is the suggested role of ATDof5.8 TF in the regulation of a network of abiotic stress responses via the downstream expression of *ANAC069* [31].

Other up-regulated genes that coincided with the enrichment of vegetative and reproductive development GO categories observed in OE-*AhERF-VII* plants, included the *E2F TF 1*, *SIMILAR TO RCD 1*, *FZO-LIKE*, *C-TERMINALLY ENCODED PEPTIDE 1*, *LATERAL ROOT PRIMORDIUM 1* and *SPIKE1* genes. The latter code for phytohormone-sensitive proteins that participate in several vegetative and/ or reproductive development processes, including organization of the thylakoid network and trichome formation (S2 Table).

WS tolerance in OE-*AhERF-VII* plants was related to increased antioxidant enzyme activity levels during stress conditions (Fig 10), and to reduced water loss rates in detached leaves (Fig 8C), the latter of which coincided with the significantly increased tendency toward stomatal closure detected in detached leaves and in leaves sampled under water-deficit stress conditions (Fig 8D and 8E). It is pertinent to add that no negative effect on seed yield was observed in OE-*AhERF-VII* plants (S4 Fig) despite the higher tendency toward stomatal closure measured in in these plants under optimal growing conditions, as well. This apparent contradiction may be explained taking into consideration previous reports indicating that cell growth, and indirectly seed yield, is generally more susceptible to water stress than to reduced CO_2_ assimilation [50]. Other factors known to affect CO_2_ assimilation and stress-yield relations but not measured in this study, such leaf area index (i. e., leaf area per unit land area [51], could have also explained the lack of a deleterious effect on seed yield observed in OE-*AhERF-VII* plants with smaller stomatal aperture and, most probably, lower CO_2_ assimilation rates. These possibilities remain to be further explored.

Contrary to the increased antioxidant enzyme activity levels observed during stress, proline accumulated to similar levels in response to both WS and SS, in both WT and transgenic plants (Fig 11). The recorded response was in accordance to the commonly observed accumulation of this aa in plants exposed to diverse abiotic stresses, given proline´s proposed role in several responses that contribute to ameliorate their noxious effects via the stabilization of sub-cellular structures, free radicals scavenging, and the regulation of cellular redox potential, among others. The fall in proline during R (Fig 11A) was also in conformity with its proposed catabolism, which is needed to provide nitrogen and reducing power to plants recovering from stress [52–54]. Nevertheless, the lack of a significant stress-activated proline accumulation suggested it did not represent a contributing factor to the increased WS or SS tolerance observed in the transgenic plants under study. This outcome was in agreement with data questioning proline´s function in stress tolerance, at least in Arabidopsis and carrot [55–57]. It also emphasized the largely unclear circumstances that affect proline accumulation, such as *de novo* synthesis, decreased degradation, altered transport between cells and cellular compartments (e. g., vacuoles, mitochondria or cytoplasm), and modified rates of utilization and/ or protein hydrolysis [52, 53, 56 58, 59]. Moreover, the results were likewise indicative of the many signaling networks known to influence ABA-mediated control of proline metabolism under stress conditions, which involve NO, hydrogen peroxide- phospholipase D and C, and SA [54].

WS conditions also led to an enrichment of GO categories associated with cell wall organization and modification. Other relevant enriched categories were those related to MG and D-lactate metabolism. This contrasted was the underrepresentation of GO categories associated, intriguingly, with ethylene, and JA responses.

Microarray data (S4 Table) indicated that the up-regulation of genes belonging to the glutaredoxin family and metallo-hydrolase/oxidoreductase superfamily proteins may have complemented WS induced antioxidant enzyme activity detected in these plants under WS. This may also hold true for *ferretin 1*, which encodes a NO-sensitive ferritin chloroplast protein that has been associated with acclimatization to excessive iron or drought conditions in tolerant bean cultivars [60]. Enrichment in cell wall organization and modification GO categories and related genes was in accordance with the plant’s need to modify cell wall synthesis and flexibility under water stress, in order to maintain root growth, at the expense of shoot growth cessation [61,62]. This concept coincided with the up-regulated expression of the salt-responsive *COBRA-like* gene, representing a family member of extracellular glycosyl-phosphatidyl inositol-anchored proteins, proposed to be involved in extracellular matrix remodeling via cellulose microfibril organization and signaling [63], and to participate in the hydraulic adaptation to salinity in *Populus* [64]. Likewise, the induction of *wall associated kinase 5* (*WAK5*) agreed with WAK’s participation in cell elongation and development during stress [65]. Also pertinent, was the up-regulation of the *EMBRYO DEFECTIVE 30* gene, encoding a small G-protein of the RAF class that positively regulates cell adhesion, cell wall organization, lateral root formation, root hair cell differentiation and phloem/ xylem histogenesis [66]. WS also led to the differential up-regulation of O-glycosyl hydrolase genes belonging to families 5 and 10, which include cellulases and xylanases known to participate in the loosening of the cell wall and subsequent plant growth, under stress [61]. Stress-related modifications of plant´s cell wall was also consistent with the down-regulation of *TRICHOME BIREFRINGENCE-LIKE 36* and *VANGUARD 1 HOMOLOG 2*. These genes encode proteins presumably involved in the synthesis and deposition of secondary wall cellulose [67] and for a putative pectin methyl esterase involved in cell wall modification and pectin catabolic processes [68], respectively. Besides, the observed underrepresentation of hydroxyproline-rich O-glycoprotein family genes which include proline-rich extensin-like and arabinogalactan protein genes, may have indicated a need to reduce cell wall rigidity under WS conditions [69].

WS induced the expression of a gene coding for a member of the lactoylglutathione lyase/ glyoxalase I (GlyI) protein family. These proteins are part of the GSH-dependent glyoxalase system employed for the detoxification of MG, a toxic metabolite known to reach growth-inhibiting levels in plants under (a)biotic stress conditions [70,71]. Additionally, manipulation of plant glyoxalases has been shown to contribute to stress tolerance. This has been particularly germane for GlyI, whose induction in several plant species or ectopic overexpression in various plant models was associated with increased abiotic stress tolerance [72]. Moreover, the previous identification of GlyI as a target of abiotic stress-related SnRK2 kinases [70] was in concordance with the up-regulated expression of *SNF1-related protein kinase 2*.*1* in response to WS.

The increased stomatal closure detected in WS plants appeared to be ABA-independent. First, because it correlated with the up-regulated expression of the *H*^*+*^*-ATPase 1* gene, coding for a negatively ABA-regulated plasma membrane proton ATPase known to participate in guard cell stomatal closure in response to drought [73]. Second, it coincided with the down-regulated expression of an unidentified At1g80180.1 gene coding for a downstream component of the ABA-independent MPK3/ 6 signaling cascade which represses stomatal biogenesis [74,75]. And, third, it differed with the down-regulation of three genes coding for well-known ABA-dependent positive regulators of stomatal closure during WS. These were the *ZINC INDUCED FACILITATOR-LIKE 1*, *GATED OUTWARDLY-RECTIFYING K+ CHANNEL*, and the *RING ZINC-FINGER PROTEIN34* or *RZFP34/CHYR1* genes, coding for a Major Facilitator Superfamily Transporter [76], a guard cell outward potassium channel [73], and a ubiquitin E3 ligase [77], respectively. Moreover, the down-regulation of *the inositol-pentakisphosphate 2-kinase 1* gene, which is involved in the biosynthesis of phytic acid, a demonstrated mediator of ABA-induced guard cell closure [78], was also detected. On the other hand, increased SOD and CAT activity levels detected in WS OE-*AhERF-VII* plants were in agreement with studies suggesting a role for ROS in the regulation of stomatal closure [79], whereas the additional participation of signaling pathways resembling those activated in response to biotic stressors, remains a possibility [80].

ABA-independent WS tolerance mechanisms was also supported by the underrepresentation of the GO inositol phosphate biosynthesis and metabolism category, which agreed with the downregulation of the *ALTERED EXPRESSION OF APX2 8* gene, encoding a bi-functional protein having inositol polyphosphate 1-phosphatase activity that negatively regulates drought tolerance and ABA signaling [81].

However the induction of a *SpRing* zinc finger gene, codifying for another small ubiquitin-like modifier (SUMO) E3 ligase that positively regulates drought and freezing tolerance via both ABA-dependent and ABA-independent pathways [82], suggested the participation of both pathways in the enhancement of WS tolerance observed in OE-*AhERF-VII* plants. Other changes, such as the induction of a diacylglycerol kinase gene (*DAG4*), *CBL-interacting protein kinase 25*, *calcium-dependent protein kinases 1*, *13*, and *31*, and *casein kinase II*, *alpha chain 2* argued in favor of the operation of ABA-dependent WS tolerance mechanisms in OE-*AhERF-VII* plants. *DAG4* is involved in phosphatidic acid biosynthesis, a lipid stress signal involved in ABA-signaling, [83], whereas the up-regulation of the calcium-dependent kinase genes coincided with the important role played by Ca^2+^ in ABA-dependent abiotic stress signaling and plant stress tolerance [84,85].

WS also modified TF gene expression, mostly on those belonging to the NAC and MYB TF families. For instance, certain NAC TF genes were exclusively repressed under Op conditions, but became induced under WS. On the other hand, WS had a predominantly negative effect on the expression levels of MYB TF genes. Curiously, among the latter were *MYB-like 102*, *MYB 16* and *LHY-CCA1-LIKE5*, a circadian clock regulator, variously involved in responses to ABA, ethylene, JA, SA and/ or salt-osmotic stress. On the other hand, the induction of NAC TF genes during WS coincided with large-scale genomic and transcriptomic studies reporting significant abiotic-stress-induced changes in the expression of a sizeable proportion of the NAC TF gene families in Arabdopsis, soybean, and rice [86]. Likewise pertinent was the up-regulation of *basic transcription factor 3*, known to be necessary for WS tolerance in wheat, partly due to its regulation of water loss rates during stress [87,88]. Regarding other ERF genes, the overexpression of the *AhERF-VII* in Arabidopsis positively affected the expression of *RAP2*.*2*, an endogenous group VII ERF TF activated by NO, an important plant signal that regulates many developmental processes, including water stress responses such as stomatal aperture and leaf senescence [46]. It also modified the expression of two group II and one group VIII ERF TF genes, only one of which (i.e., RAP2.1) was up-regulated. The latter was curiously shown to be a negative regulator of cold and drought stress in Arabidopsis [89], similarly to other group VIII TFs, which can be negative regulators of ethylene-, JA-, and ABA-responsive gene expression [90]. Further down-regulation of *ETHYLENE INSENSITIVE 4*, coding for an ethylene receptor that negatively regulates the ethylene-activated signaling pathway [18], and of *TARGET OF EARLY ACTIVATION TAGGED 2*, involved in ethylene-activated development programs [91], was observed. These results suggest that the overexpression of the *AhERF-VII* transgene affected endogenous ethylene-related stress signaling in Arabidopsis.

The abundance and type of miRNA´s genes in OE-*AhERF-VII* plants was clearly influenced by the experimental conditions applied (S8 Table) and were, at times, contradictory. For instance, miR164c’s expression was induced under WS and R, which disagreed with its reported role as a negative regulator of drought tolerance in rice [92].

The differential expression pattern of repeat domain gene families observed in WS plants, including Armadillo and Pentatricopeptide (mostly down-regulated) and WD40, Tetratricopeptide, ankyrin/ BTB/ POZ domain and Leucine rich repeats (mostly up-regulated) was in agreement with the ability of repeat proteins to integrate protein complexes and influence plant stress responses and hormone signaling. Representative examples include catalase stabilization during oxidative stress, transpiration or adaptation to water deprivation and oxidative stress through ROS homeostasis and positive/ negative regulation of ethylene and ABA signaling [93]. Finally, an over-representation of ABC/ pleiotropic drug transporter genes during WS could be connected with the tolerance observed in OE-*AHERF-VII* plants. Although none of the above include known ABA transporters, their participation in drought stress amelioration through ABA transport cannot be discarded, considering their plastic nature, and the fact that many of them remain to be functionally analyzed [94].

It stems from the above discussion, that the numerous factors contributed to the WS tolerance observed in OE *AhERF-VII* Arabidopsis plants, and that this tolerance occurred via both ABA-dependent and ABA-independent mechanisms. An intriguing question that remains to be answered is why so many obviously WS tolerance-related genes that were up-regulated in OE-*AhERF-VII* plants in Op conditions (e.g., water channels, dehydration responsive proteins and key ABA biosynthesis or response genes), ceased to be significantly represented under WS and/ or R conditions.

An inverse effect resulted from the overexpression of the *Ah-Dof-AI* gene in Arabidopsis, which tolerated SS but not WS. Otherwise, OE-*AhDof-AI* plants kept in Op conditions were no different from WT plants. This, despite the almost 3-fold difference between the number of under- and up-regulated genes yielded by the microarray assay, many of which involved development-related genes. Similarly, a significant under-representation of GO categories associated with radiation and light responses, usually related to vegetative and reproductive development programs was observed (S7 Table). Another noteworthy GO category modification was the reduced representation of numerous transport related categories in Op conditions. This tendency was maintained in SS plants, despite the fact that many genes related to the latter (e.g., *sodium hydrogen exchanger 4*, *cation exchangers 5* and *6*, *vacuolar ATP synthase subunit C*, *vacuolar proton ATPase A2* involved in vacuolar sequestration of NaCl and other ions), are usually enriched under stress [95]. Nevertheless, two member genes of the Major Facilitator Superfamily of membrane transporters (or MATE) were among the most highly induced genes in SS plants. These transporters participate in cellular detoxification processes in plants, via the elimination of toxic compounds from the cell, through extrusion or sequestration in the vacuole [96]. Pertinent to this property was a reported transcriptional profile of salt-stressed Arabidopsis roots that revealed the enrichment MATE transporters [97]. In addition, putative salt responsive MATE genes were identified in Arabidopsis, rice, and chickpea [98] whereas an Arabidopsis Golgi-localized MATE transporter was found to be induced by excessive iron and osmotic stress conditions [99]. In like manner, the up-regulation of a cyclic nucleotide gated *Ca*^*2+*^*-permeable cation channel 5* gene in SS plants coincided with the proposed role for a similar channel in the conferral of salt tolerance to Arabidopsis [100]. Mention must be made, however, that similar transporter genes were also down-regulated during SS in these plants. These included the cyclic nucleotide-gated channel *CNGC 17* gene, identified as a growth regulator in Arabidopsis [101], and three additional MATE efflux family protein genes. In addition, SS led to the exclusive down-regulation of three ATP-BINDING CASSETTE genes (see below), and other transporter genes involved in phosphate and nitrate transport.

Similar to OE-*AhERF-VII* plants, biochemical analysis of OE-*Ah-Dof-AI* plants revealed that SS tolerance was associated with significantly higher SOD activity levels and Glu accumulation. However, SS tolerance in these plants did not involve modified stomatal closure, as supported by a down-regulation of GO categories related to stomatal complex morphogenesis and development. Likewise, genes associated with stomata movement were mostly down-regulated, as well. The latter code for ARABIDOPSIS THALIANA ATP-BINDING CASSETTE C5, a high-affinity inositol hexakisphosphate transporter, ABA DEFICIENT 3, implicated in ABA biosynthesis and RESPIRATORY BURST OXIDASE HOMOLOG F, linked to ABA-activated signaling pathways [73]. Conversely, the GO analysis indicated that SS tolerance in OE-*Ah-Dof-AI* plants coincided with an enrichment of categories associated with response to xenobiotic/ biotic stimulus and to flooding and related nitric oxide metabolic process. The latter result was surprising, more expected of plants transformed with the *AhERF-VII* transgene, similar to ERF TFs which are known to regulate NO-dependent low-oxygen stress responses triggered during flooding [46]. Moreover, none of the five ERF genes that were up-regulated in SS plants corresponded to this group. On the other hand, the above concurred with the up-regulation of GO categories (i.e. photorespiration and energy reserve metabolic processes) and genes associated with a lower energy status due to anoxia. These included genes coding for a methionine aminopeptidase 2A, catalyzing the regulatory N-terminal methionine cleavage required for ERF-VII TFs degradation [102] and for glutamate: glyoxylate aminotransferase 1, involved in the peroxisomal metabolic salvage pathway induced in response to hypoxia [103]. Similarly up-regulated were genes activated for nutrient mobilization or triggered in response to starvation (e.g., *CA*^*2+*^*-ACTIVATED RELA/ SPOT HOMOLOG*, *N-terminal asparagine amidohydrolase*, and *pyruvate orthophosphate dikinase*) and coding for sucrose synthase 1, preferentially employed for the enzymatic breakdown of sucrose in ATP-limiting conditions [104–106]. Also pertinent with a response to anoxia was the down-regulation several GO categories and genes involved in the regulation of C: N metabolism, incorporating fatty acid oxidation, the TCA cycle and of many other plant catabolic processes [107]. GO analysis also showed an impoverishment of a many categories associated with abiotic stress tolerance involving ABA, ethylene, ROS, MAPKs, response to salt and osmotic stress, and polyol biosynthetic processes. This outcome coincided with the down-regulation of ABA biosynthetic genes (e.g. *ABA DEFICIENT 3*), and the concurrent induction of *ATAF1*, *a* NAC TF that attenuates ABA signaling and synthesis [108]. In addition, several kinase genes (e.g., *MAP kinase 9*, *11*, and *19*; *MAPK homolog 2*; *shaggy-like kinase 42*) and numerous others associated to SS and oxidative stress responses were found to be repressed. These included *RGA-LIKE 1*, a positive regulator of SOD transcription, *glutamate dehydrogenase 2* and *isopropylmalate dehydrogenase 3*, both associated with regulating C: N metabolism under stress, *dehydrin xero 1*, and a salt-stress induced TRAF-like family protein gene [109–112]. Nonetheless, the microarray data also revealed the up-regulated expression of numerous genes, including TFs, similarly related to SS responses. Illustrative examples include *CER26* required for wax biosynthesis, ABA-, ethylene, and/ or salt/ osmotic stress-responsive TF genes such as *MYB38*, *ABRE BINDING FACTOR 4*, *BASIC LEUCINE-ZIPPER 1*, and *RAP2*.*1*, *2*.*3*, *2*.*17*, and *TINY* [113]. Other induced emblematic genes were those coding for the following: desiccation-responsive protein 29A, glycine-rich protein 8, responsive to dehydration 21b, indole-3-acetic acid 7, six different late embryogenesis abundant proteins, and the SNF1-related 2.9 and 3.10 protein kinases, in addition to the membrane-associated kinase regulator 6 [114]. Similarly indicative was the down-regulation, also detected in OE-*AhERF-VII* plants under WS, of the *APX2 8* gene, involved in the negative regulation of ABA-dependent drought and salt tolerance [115].

Additional microarray data suggest that SS tolerance in OE-*AhDof-AI* plants was associated with chromatin remodeling and histone modifications, in accordance with previous reports [116]. It also evinced a rather counterintuitive SS-up-regulation of numerous development-related GO categories and genes, predominantly involved in reproductive functions, which merits further research. Similarities with WS induced responses in OE-*AhERF-VII* plants were observed, as well. Included were the up-regulation of participant genes in the MG detoxification cycle and in cell wall modifications (Table 1). Despite this coincidence, several genes involved in cytoskeleton organization were found to be induced only in response SS plants. It has been suggested that some of these gene products might mediate cytoskeleton-cell wall communication via specialized adhesion domains controls, in order to regulate cell growth, differentiation and cell-to-cell communication in plants [117]. In addition, genes coding for expansin A25, member of α-expansin gene family that participate in cell wall loosening [61], and for mucilage-related 10, a galactomannan-1, 6-galactosyltransferase required for the maintenance of cellulose structure, mucilage density, as well as the adherence of pectin [118], were induced exclusively in SS plants. Loosening of plant cell walls in response to stress was also in agreement with the down-regulation of genes associated with lignification, such as those coding for caffeoyl shikimate esterase and hydroxycinnamoyl-CoA shikimate [119], in addition to *arabinan deficient 1*, possibly involved in the determining the mechanical properties of the cell wall [120].

Other genes that, in like manner, were affected in both stressed transgenic plants (Table 1) are involved in Ca^2+^ transport and signaling which plays a crucial role in the regulation of stress responses [121]. The shared expression of nucleotide sugar transporters genes supports the proposed participation of nucleotide sugar inter-conversion enzymes in the regulation of glycosylation patterns that control carbon flux between primary metabolism, cell wall formation and/ or the biosynthesis of stress-related osmolytes, such as galactinol, that occurs in response to stress-related perturbations [122]. A shared up-regulation of an *UDP-N-acetylglucosamine pyrophosphorylase* gene suggests a common stress-activated leaf senescence program, as reported in rice [123], whereas the up-regulated expression of the *ANAC 36* gene suggests a novel role for this TF in the positive regulation of salt and water-deficit stresses [124]. A similar novel function for *MYB 58* and *MYB 117* (or LOF1) TF genes in abiotic stress regulation could be inferred by their shared expression in WS and SS. This result complements the previously reported role for these TFs in the regulation of pigment and lignin biosynthesis in fibers and vessels [47]. Likewise, the shared induced expression of the *Responsive to desiccation 29A* and *sugar transport 1* genes was also congruent with their reported induction by desiccation, cold and high-salinity conditions, [125], and the need to cope with the increased synthesis of sugar and polyol compounds under stress [94,114].

The metabolite analysis, although hypothetical, permitted the proposal of additional stress-amelioration mechanisms that partly complemented those discussed previously. For example, many putative hormone-like metabolites appeared to accumulate in response to stress. Such possibility is congruent with the finding that auxins, cytokinins, gibberellins and brassinosteroids are important mediators of abiotic stress tolerance [126–131]. Among the proposed mechanisms for their stress-protective effect are modulation of the antioxidant system and energy status of the plant, as well as amelioration of the adverse effects of stress and the restoration of normal growth and development. On the other hand, citrulline, identified as a marker of both WS and SS in melon [132], and a by-product of the biosynthesis of the ubiquitous stress-associated NO signal [133], is also proposed to contribute to stress tolerance by dint of its effective scavenging of hydroxyl radicals and strong antioxidant activity. Likewise, Mg-protoporphyrin IX and vitamin B6 have been implicated in protection against cellular oxidative stress. The former, due to its ability to associate with several proteins associated with oxidative stress responses [134], and the latter, to its capacity to quench ROS [135]. Transgenic-mediated accumulation of N-acetyl-L-glutamate and consequent abiotic stress tolerance correlated with high levels of ornithine, the precursor of proline and polyamine biosynthesis [136]. Further, geraniol induced transcript accumulation of glutathione S-transferase and stress-associated ERBP and WRKY TFs genes in *Matricaria chamomilla* [137], whereas geraniol and citral, increased substantially in response to WS in lemongrasses [138]. In like manner, experimental evidence indicates a possible crosstalk between stress-associated physiological processes and glucosinolate metabolism [139]. The detection of putative glycerol agreed with the observed correlation between glycerol accumulation and salt stress tolerance in Arabidopsis and in highly salt-tolerant ice plants [140,141]. The critical role of polyamines in stress tolerance has been amply demonstrated [142], whereas selenium, a beneficial nutrient for plants, has been implicated in various mechanisms associated with abiotic stress amelioration. These include the regulation of ROS and antioxidants, an inhibition of heavy metal uptake and translocation, and a role in the recovery of damaged cell membrane and chloroplast structures, including the photosynthetic system [143].

To conclude, this study shows that the OE of two grain amaranth TF genes increase the tolerance to specific abiotic stresses in transgenic Arabidopsis plants. The mechanisms by means of which these genes confer stress tolerance appear to be multifactorial and to involve both ABA- dependent and ABA-independent regulatory pathways.

## Supporting Information

S1 Fig Predicted properties of the AhERF-VII and AhDOF-AI transcription factors. (DOC)

S2 Fig Expression patterns of the *AhERF-VII* gene in *A*. *hypochondriacus* infected with two different bacterial pathogens. (DOCX)

S3 Fig Levels of expression, relative to background expression in WT plants, in four homozygous transgenic *A*. *thaliana* T_2_ lines overexpressing the *AhERF-VII* or the *AhDOF*-*AI* genes. (DOCX)

S4 Fig The overexpression of the *AhERF-VII* or *AhDof-AI* genes in transgenic *Arabidopsis* plants had no negative effects on vegetative or reproductive growth. (DOCX)

S5 Fig Soluble nonstructural carbohydrates (NSCs) quantification in transgenic OE-*AhERF-VII* (line EL25) and OE-*AhDof-AI* (line DL2) *Arabidopsis* plants subjected to water-deficit stress (WS) or acute salt stress (SS). (DOCX)

S6 Fig Number of genes differentially expressed in transgenic *OE-AhERF-VII* or *OE-AhDOF-AI* transgenic Arabidopsis plants under optimal conditions (Op), subjected to water-deficit (WS) or salt (SS) stress conditions, or in recovery after WS (R), and their proportional overlap. (DOCX)

S1 Table Sequence of the primers employed for PCR amplification in this study. (DOCX)

S2 Table List of genes with altered expression detected in transgenic *AhERF-VII* overexpressing Arabidopsis plants under optimal conditions. (DOCX)

S3 Table List of genes with altered expression detected in transgenic *AhDOF-IA* overexpressing Arabidopsis plants under optimal conditions. (DOCX)

S4 Table List of representative genes with altered expression detected in transgenic *AhERF-VII* overexpressing Arabidopsis plants under water-deficit stress. (DOCX)

S5 Table List of genes with altered expression detected in transgenic *AhDOF-IA* overexpressing Arabidopsis plants under salt-stress conditions. (DOCX)

S6 Table GO categories found to be significantly modified in *AhERF-VII* overexpressing transgenic Arabidopsis plants in optimal conditions, under water-deficit stress (WS), or in recovery, after stress (R). (DOCX)

S7 Table GO categories found to be significantly modified in *AhDof-AI* overexpressing transgenic Arabidopsis plants in optimal conditions and under salt stress (SS). (DOCX)

S8 Table Modified microRNA gene expression. (DOCX)
