# Supplementary material for: Overexpression of Grain Amaranth (Amaranthus hypochondriacus) AhERF or AhDOF Transcription Factors in Arabidopsis thaliana Increases Water Deficit- and Salt-Stress Tolerance, Respectively, via Contrasting Stress-Amelioration Mechanisms
Source: PLoS One. 2016 Oct 17;11(10):e0164280. doi: 10.1371/journal.pone.0164280 (PMC5066980; doi:10.1371/journal.pone.0164280)
Supplement: S1 Fig — (DOC) [file pone.0164280.s001.doc]

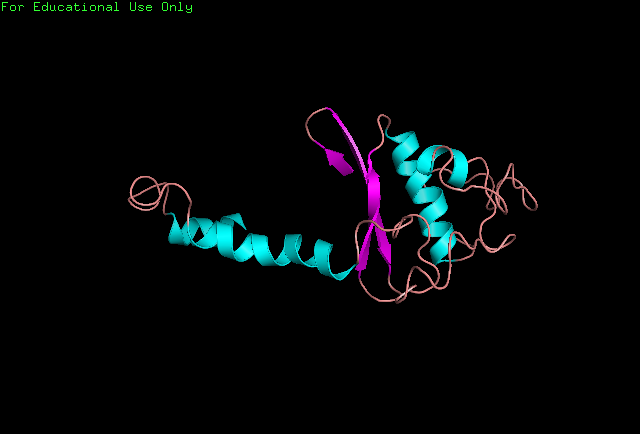

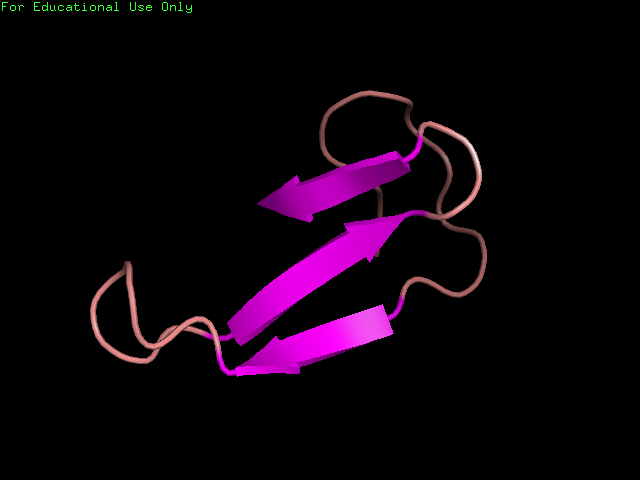


**C**

**D**

**A**


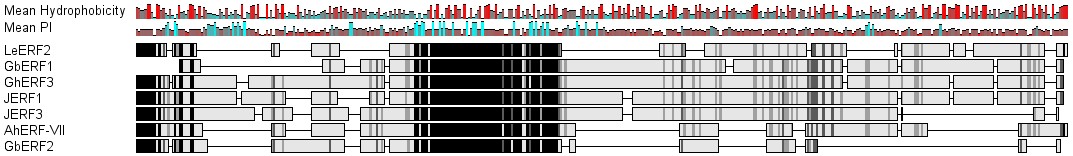

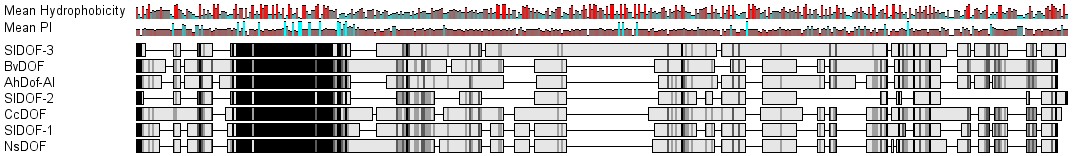


**B**

`

**S1 Fig. Predicted characteristics of the AhERF-VII and AhDOF-AI transcription factors.** Predicted consensus level, mean hydripobicity and mean isolectric point in AhERF-VII (panel A) and AhDOF-AI (panel B) transcription factors (TFs) with other TF family members from selected plant species. The predicted secondary structure of AhERF-VII and AhDOF-AI proteins is shown in panels **C** and **D**, respectively. The DNA binding domains are outlined in a yellow oval. Extended strands are colored in purple, alpha helices in cyan, and random coils in brown.
