# Supplementary material for: Overexpression of Grain Amaranth (Amaranthus hypochondriacus) AhERF or AhDOF Transcription Factors in Arabidopsis thaliana Increases Water Deficit- and Salt-Stress Tolerance, Respectively, via Contrasting Stress-Amelioration Mechanisms
Source: PLoS One. 2016 Oct 17;11(10):e0164280. doi: 10.1371/journal.pone.0164280 (PMC5066980; doi:10.1371/journal.pone.0164280)
Supplement: S2 Fig — (DOCX) [file pone.0164280.s002.docx]

# AhERF-VII


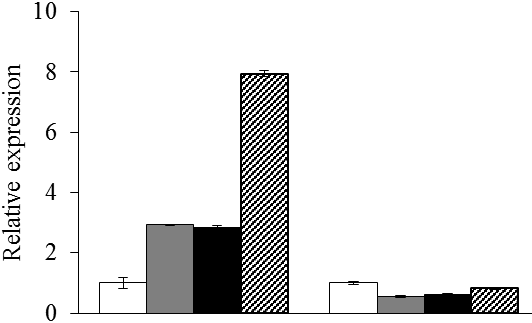


Avr Vir

Ctl

4 days

6 days

8 days

**S2 Fig.** **Expression patterns of the *AhERF-VII* gene in *A. hypochondriacus* infected with two different bacterial pathogens.** The figure shows that the expression of the *AhERF-VII* gene gradually increased in a time-dependent manner in leaf tissues infected with the avirulent *Pseudomonas syringae* pv. *syringae* (*Pss*) bacterial pathogen. On the other hand, the expression of this gene was not modified by the infection with the virulent *P. syringae* pv. *tabaci* (*Pst*) bacterial pathogen. Plants treated with phosphate buffer solution only were used as controls (Ctl). Bars and error bars indicate mean values and ES, respectively (n = 6).
