# Supplementary material for: Overexpression of Grain Amaranth (Amaranthus hypochondriacus) AhERF or AhDOF Transcription Factors in Arabidopsis thaliana Increases Water Deficit- and Salt-Stress Tolerance, Respectively, via Contrasting Stress-Amelioration Mechanisms
Source: PLoS One. 2016 Oct 17;11(10):e0164280. doi: 10.1371/journal.pone.0164280 (PMC5066980; doi:10.1371/journal.pone.0164280)
Supplement: S3 Fig — (DOCX) [file pone.0164280.s003.docx]

**A**

**B**

EL25 EL2 EL9 EL15

Transgenic line


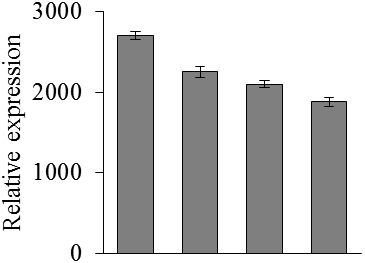


*****

*****

*****

*****

*****

*****


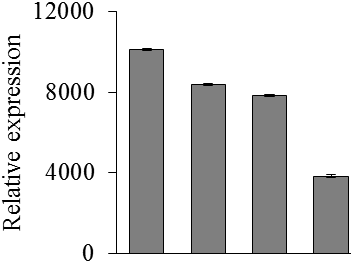


DL2 DL31 DL28 DL4

Transgenic line

(1.4×)

(1.2×)

(1.1×)

(1.0×)

(2.6×)

(2.2×)

(2.0×)

(1.0×)

**S3 Fig.** **Transgene gene expression.** Levels of expression, relative to background expression in WT plants, in four homozygous transgenic *A. thaliana* T_2_ lines overexpressing the *AhERF-VII* (panel A) or the *AhDOF*-*AI* (panel B) genes. In each panel, the three lines marked with an asterisk, having high, medium, and low gene expression levels, were chosen for further study. Bar and errors indicate mean values and SE, respectively (n = 30). The numbers over the bars represent the fold-change in expression levels relative to the transgenic lines expressing the lowest values, which was set at 1.0
