# Supplementary material for: Overexpression of Grain Amaranth (Amaranthus hypochondriacus) AhERF or AhDOF Transcription Factors in Arabidopsis thaliana Increases Water Deficit- and Salt-Stress Tolerance, Respectively, via Contrasting Stress-Amelioration Mechanisms
Source: PLoS One. 2016 Oct 17;11(10):e0164280. doi: 10.1371/journal.pone.0164280 (PMC5066980; doi:10.1371/journal.pone.0164280)
Supplement: S4 Fig — (DOCX) [file pone.0164280.s004.docx]

# A

Weight (mg)


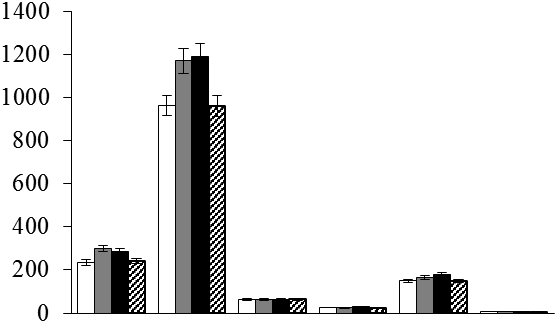


Rt In R Rt In R

Fw Dw

WT

EL25

EL2

EL15

# C


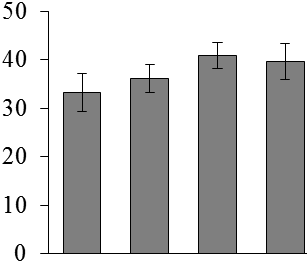


Wt EL25 EL2 EL15

Seed yield (mg/plant)

**D**


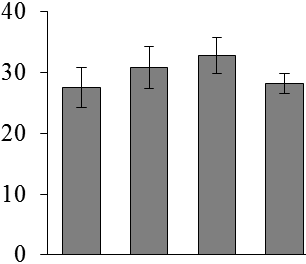


Wt DL2 DL31 DL4

Seed yield (mg/plant)

# B


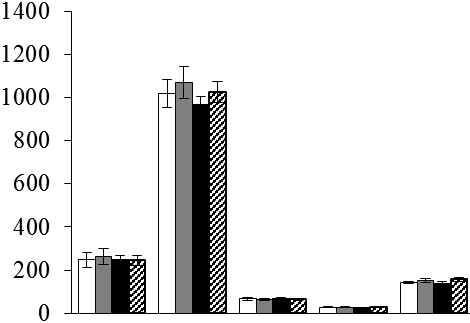


Rt In R Rt In R

Fw Dw

Weight (mg)

WT

DL2

DL31

DL4

**S4 Fig.** **The overexpression of the *AhERF-VII* or *AhDof-AI* genes in transgenic *Arabidopsis* plants had no negative effects on vegetative or reproductive growth.** Panels A and B show the weight (FW) and dry (DW) weights of the rosettes (Rt), inflorescences (In), and roots (R) of mature Arabidopsis transgenic plants. The plants examined were OE-*AhERF-VII* (lines EL25, gray bars; EL2, black bars, and L15, stripled bars), OE-*AhDOF-AI* (lines DL2, gray bars; DL31, black bars, and DL4, stripled bars) and WT control plants (empty bars). Panels C and D show that OE-*AhERF-VII*, OE-*AhDOF-AI* and WT control plants produced similar seed yields. Bars and error bars indicate mean value and SE, respectively of three independent experiments (n = 10).
