# Supplementary material for: Overexpression of Grain Amaranth (Amaranthus hypochondriacus) AhERF or AhDOF Transcription Factors in Arabidopsis thaliana Increases Water Deficit- and Salt-Stress Tolerance, Respectively, via Contrasting Stress-Amelioration Mechanisms
Source: PLoS One. 2016 Oct 17;11(10):e0164280. doi: 10.1371/journal.pone.0164280 (PMC5066980; doi:10.1371/journal.pone.0164280)
Supplement: S5 Fig — (DOCX) [file pone.0164280.s005.docx]

ab

b

c

a


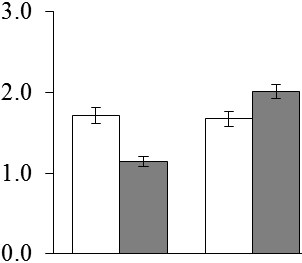


Umol /g FW

Op SS

## D

WT

DL2

**A**

WT

EL25


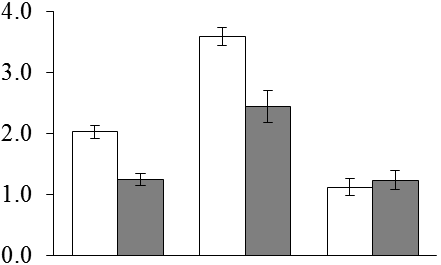


Umol /g FW

Op WS R

b

a

c

b

c

c

**Glu**

**B**

WT

EL25


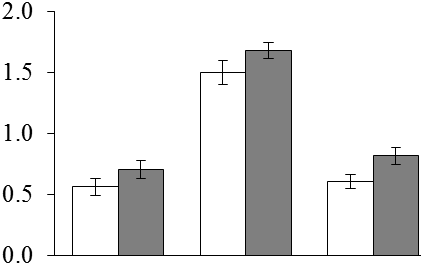


Umol /g FW

Op WS R

b

b

b

b

a

a

**E**

WT

DL2


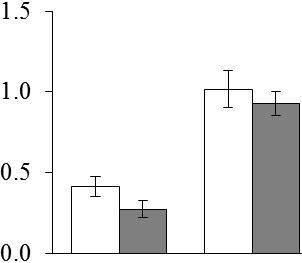


Umol /g FW

Op SS

b

b

a

a

**Fru**

**C**

WT

EL25


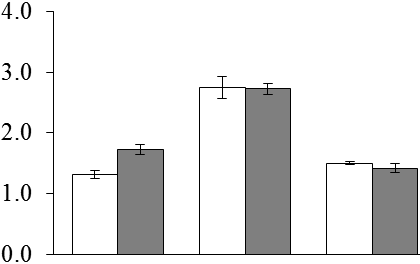


Op WS R

Umol /g FW

b

b

b

b

a

a

**F**

WT

DL2


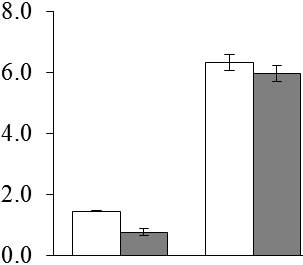


Op SS

Umol /g FW

b

b

a

a

**Suc**

**S5 Fig.** **Soluble nonstructural carbohydrates (NSCs) quantification in transgenic OE-*AhERF-VII* (line EL25) and OE-*AhDof-AI* (line DL2) *Arabidopsis* plants subjected to water-deficit stress (WS) or acute salt stress (SS).** NSCs quantification in transgenic OE-*AhERF-VII* (line EL25) (panels A-C) and OE-*AhDof-AI* (line DL2) (panels D-F) *Arabidopsis* plants subjected to water-deficit stress (WS) or acute salt stress (SS). Changes in glucose (A and D), fructose (B and E) and sucrose (C and F) levels are shown for all plants maintained under optimal conditions (Op, empty bars). Gray bars represent the NSC levels in transgenic plants subjected to water-deficit stress (WS) and subsequent recovery (R) or to acute salt stress (SS). Bars and error bars indicate mean values and ES, respectively (n = 20). Different letters over the bars represent statistically significant differences at P ≤ 0.05 (Tukey Kramer test). The results shown are those obtained from a representative experiment that was repeated thrice with similar results.
