# Supplementary material for: Overexpression of Grain Amaranth (Amaranthus hypochondriacus) AhERF or AhDOF Transcription Factors in Arabidopsis thaliana Increases Water Deficit- and Salt-Stress Tolerance, Respectively, via Contrasting Stress-Amelioration Mechanisms
Source: PLoS One. 2016 Oct 17;11(10):e0164280. doi: 10.1371/journal.pone.0164280 (PMC5066980; doi:10.1371/journal.pone.0164280)
Supplement: S6 Fig — (DOCX) [file pone.0164280.s006.docx]

**A**

**B**

**C**

**D**

**E**

**F**

524

713

91- 9%

121-8%

525

768

Op *vs.* WS

*AhERF-VII*

615

650

75- 7%

81-6%

525

768

Op *vs.*  R

*AhERF-VII*

615

650

151- 13%

174-13%

524

713

WS *vs.* R

*AhERF-VII*

460

886

117- 14%

382-21%

387

927

Op *vs.* SS

*AhDof-AI*

387

927

89- 10%

219-13%

525

768

*Ah-ERF* *vs.*  *AhDof-AI*

Op

460

886

104- 11%

131-8%

524

713

*Ah-ERF* *vs.* *AhDof-AI*

WS SS

**S6 Fig**. **Number of genes differentially expressed in transgenic *OE-AhERF-VII* or *OE-AhDOF-AI* transgenic Arabidopsis plants under optimal conditions (Op), subjected to water-deficit (WS) or salt (SS) stress conditions, or in recovery after WS (R), and their proportional overlap.** Panels A-C and panel D show the differential gene overlap in all treatment combinations analyzed in *OE-AhERF-VII* and *OE-AhDOF-AI* transgenic Arabidopsis plants, respectively. Panels E and F show the differential gene overlap between the *OE-AhERF-VII* and *OE-AhDOF-AI* plants under optimal and stress conditions, respectively. Upward pointing arrows indicate upregulated genes, whereas downward pointing arrows indicate downregulated genes.
