# Supplementary material for: Overexpression of Grain Amaranth (Amaranthus hypochondriacus) AhERF or AhDOF Transcription Factors in Arabidopsis thaliana Increases Water Deficit- and Salt-Stress Tolerance, Respectively, via Contrasting Stress-Amelioration Mechanisms
Source: PLoS One. 2016 Oct 17;11(10):e0164280. doi: 10.1371/journal.pone.0164280 (PMC5066980; doi:10.1371/journal.pone.0164280)
Supplement: S1 Table — (DOCX) [file pone.0164280.s007.docx]

| **S1 Table.** **Sequence of the primers employed for PCR amplification in this study.** The table includes oligonucleotide sequences employed for diverse purposes, including qRT-PCR, RACE, endogenous expression levels in grain amaranth (*Ah*) and confirmation of heterologous expression in Arabidopsis. | | | |
| --- | --- | --- | --- |
| **Primer**  **nomenclature** | **5’-3’ oligonucleotide sequence** | **Primer**  **nomenclature** | **5’-3’ oligonucleotide sequence** |
| **Expression levels in *Ah* by qRT-PCR** | | **Complete *ERF-VII and Dof-AI* cDNA sequences (RACE) in *Ah*** | |
| qERF-F | TGATTCTCTTCCTCCACCAT | Race5'-ERF | *GGTGGTGATGGTGGAGGAAGAGAAT* |
| qERF-R | CTGAAACAACATTATCCATTAGG | Race3'-ERF | *ATTCTCTTCCTCCACCATCACCACC* |
| qDOF-F | TGAAGTGGACACGCAAAAT | Race5'-DOF | *ACTCAATCCGAAATTCTCCGTTTGC* |
| qDOF-R | CCTAAATACCCCAACTATCCC | Race3'-DOF | *GCAAACGGAGAATTTCGGATTGAGT* |
| qACT-F | CGTGACCTGACTGATTACCTTA |  | |
| qACT-R | GCCATTGAGAAGAACTACGAGC |  |  |
| qTUB-F | TCTCAGCAGTATGTCTCCCTCA |  |  |
| qTUB-R | AAGATGAGCACCAAAGAAGTAGA |  |  |
|  |  |  |  |
| **Cloning of *AhERF-VII* and *AhDOF-AI* in Arabidopsis** | | **Cloning of *AhERF-VII-GFP* and *AhDOF-AI-GFP* fusion in Arabidopsis** | |
| ERF pB7-F | *CCCTTGTTGATAGTGAATGTTG* | ERFGFP-F | *ATGTGCGGTGGTGCAATTATT* |
| ERF pB7-R | *TACTATTAAAAAGGCAAAGCATCA* | ERFGFP-R | *TCAAAATAAGAAATTATCATTGAGGTCAT* |
| DOF pB7-F | *CAAATGCAAGACGTACAACAAATTA* | DOFGFP-F | *ATGCAAGACGTACAACAAATTAATG* |
| DOF pB7-R | *AAAATATCAAGGAAGAAAGAGTGGAAG* | DOFGFP-R | *TCAAGGAAGAAAGAGTGGAAGAT* |
|  |  |  |  |
