# Supplementary material for: Overexpression of Grain Amaranth (Amaranthus hypochondriacus) AhERF or AhDOF Transcription Factors in Arabidopsis thaliana Increases Water Deficit- and Salt-Stress Tolerance, Respectively, via Contrasting Stress-Amelioration Mechanisms
Source: PLoS One. 2016 Oct 17;11(10):e0164280. doi: 10.1371/journal.pone.0164280 (PMC5066980; doi:10.1371/journal.pone.0164280)
Supplement: S2 Table — (DOCX) [file pone.0164280.s008.docx]

**S2 Table.** **List of genes with altered expression detected in transgenic *AhERF-VII* overexpressing Arabidopsis plants under optimal conditions.**

| **DISEASE RESISTANCE** | |
| --- | --- |
| **INDUCED** | **REPRESSED** |
| **Disease resistance protein (TIR-NBS-LRR class) family; VICTR (VARIATION IN COMPOUND TRIGGERED ROOT growth response) encodes a TIR-NB-LRR (for Toll-Interleukin1 Receptor-nucleotide binding-Leucine-rich repeat) protein**. VICTR is necessary for DFPM-induced root growth arrest and **inhibition of abscisic acid-induced stomatal closing** (DFPM is [5-(3,4-dichlorophenyl)furan-2-yl]-piperidine-1-ylmethanethione)(PMID:21620700). DFPM-mediated root growth arrest is accession-specific and depends on EDS1 and PAD4; Col-0 has a functional copy of VICTR. Induction of the VICTR gene by DFPM treatment requires functional VICTR (Col). A close homolog to VICTR, named VICTL (At5g46510) lies in tandem with VICTR. The mRNA is cell-to-cell mobile. | Disease resistance protein (**TIR-NBS-LRR class**) |
| Disease resistance protein (**TIR-NBS-LRR class**) family; Disease resistance protein (TIR-NBS-LRR class) family | Disease resistance protein (**TIR-NBS-LRR class**) family. |
|  | Disease resistance protein (**TIR-NBS-LRR class**) family. |
|  | Disease resistance protein (**TIR-NBS-LRR class**) family |
|  | Disease resistance protein (**TIR-NBS-LRR class**) |
|  | Disease resistance family protein / **LRR family protein** |
|  |  |
| Disease resistance-responsive (**dirigent-like protein**) family protein. |  |
|  |  |
| **Receptor like protein 53**; expressed in guard cell; INVOLVED IN: signal transduction, defense response.  Disease resistance-responsive (**dirigent-like protein**) family protein; INVOLVED IN: lignan biosynthetic process, defense response.  Disease resistance protein (**NBS-LRR class**) family; Disease resistance protein (TIR-NBS-LRR class) family; INVOLVED IN: apoptosis, defense response | ALTERED SEED GERMINATION 6, ASG6, CRK2, **CYSTEINE-RICH RLK (RECEPTOR-LIKE PROTEIN KINASE) 2**; Encodes a cysteine-rich receptor-like protein kinase**. Defense response**, protein autophosphorylation, response to ozone.  CRK33, CYSTEINE-RICH RLK (RECEPTOR-LIKE PROTEIN KINASE) 33  Encodes a cysteine-rich receptor-like protein kinase. Defense response to bacterium.  ATRLP46**, RECEPTOR LIKE PROTEIN 46**, RLP46; **Leucine-rich repeat**-containing N-terminal domain, type 2. |
|  |  |
| **THI2.1, THI2.1.1, THIONIN 2.1; Encodes a thionin** which is a cysteine rich protein having antimicrobial properties. **Thi2.1 is expressed in response to a variety of pathogens and induced by ethylene and jasmonic acid**. Belongs to the plant thionin (PR-13) family. Defense response, jasmonic acid mediated signaling pathway. | **Thionin 2.2** |
| Encodes a **defensin-like (DEFL) family protein**; defense response to fungus.  THAUMATIN-LIKE PROTEIN 1; TTLP1, THAUMATIN-LIKE PROTEIN 1, TLP1. Root-specific expression activated in response to rhizobacteria and **ACC. Role in induced systemic resistance; response to ethylene.** |  |
| **CAP (Cysteine-rich secretory proteins**, Antigen 5, and Pathogenesis-related 1 protein) superfamily protein; **basic pathogenesis-related protein 1.** |  |
| Pathogenesis-related **thaumatin s**uperfamily protein |  |
| Pathogenesis-related **thaumatin** superfamily protein |  |
| Pathogenesis-related **thaumatin** superfamily protein |  |
|  |  |
| ATLFG3, LFG3, **LIFEGUARD 3; Bax inhibitor-1 family protein**; CONTAINS InterPro DOMAIN/s: Inhibitor of apoptosis-promoting Bax1 related. | **Bax inhibitor-1** family protein. |
|  | **BAX inhibitor 1** |
|  |  |
| 2-CYSTEINE PEROXIREDOXIN B: **defense response to bacterium**, oxidation-reduction process, response to cold |  |
| **Eukaryotic aspartyl protease family protein**; Encodes a protein with aspartic protease activity (also known as aspartate-type endopeptidase activity). Overexpression of the gene was shown to lead to **salicylic acid (SA)-mediated disease resistance** upon exposure to the pathogen Pseudomonas syringae. Moreover, overexpression of this gene led to the upregulation of two pathogenesis-related genes PR1 and PR2. |  |
| **Phytosulfokin receptor 1; Innate immune response**, protein phosphorylation, **regulation of defense response**, **response to wounding**, transmembrane receptor protein tyrosine kinase signaling pathway |  |
|  |  |
| **Glycoside hydrolase, family 18**; Glycosyl hydrolase family protein **with chitinase insertion domain.** |  |
| **Glycoside hydrolase, family 18**; chitinase class II group which includes **chitinase,** **chitodextrinase**. |  |
|  |  |
| RPM1 interacting protein 3; RIN3, **RPM1 INTERACTING PROTEIN 3**, positive regulation of **plant-type hypersensitive response**, proteasome-mediated ubiquitin-dependent protein catabolic process, protein polyubiquitination, protein ubiquitination involved in ubiquitin-dependent protein catabolic process. | **RPM1 interacting protein 13**, a resistance protein interactor shown to **positively enhance resistance function of RPM1**. |
|  | **NIM1-INTERACTING 3**, NIMIN-3 encodes a kinase that physically interacts with NPR1/NIM1; **regulation of systemic acquired resistance.** |
|  |  |
| **Lectin protein kinase family protein**; CALLUS EXPRESSION OF RBCS 101, CES101, **RESISTANCE TO FUSARIUM OXYSPORUM 3**, RFO3. |  |
| **Hydroperoxide lyase 1.** |  |
|  |  |
|  | **Defender against death (**DAD family) protein. |
|  |  |
|  | **RESTRICTED TEV MOVEMENT 3** |
|  |  |
|  | ATTLP7, TLP7, **TUBBY LIKE PROTEIN 7**; **Response to fungus** |
|  |  |
|  | **Serpin 3** |
|  |  |
| **CELL WALL SYNTHESIS/ MODIFICATION-CYTOSKELETON** | |
| **INDUCED** | **REPRESSED** |
|  | **LORELEI-LIKE-GPI-ANCHORED PROTEIN 1** |
|  | **LORELEI-LIKE-GPI ANCHORED PROTEIN 2** |
|  | **COBRA-like extracellular glycosyl-phosphatidyl inositol-anchored protein family** |
|  | **COBRA-like protein** |
|  |  |
| **Glycosyl hydrolase family 38** protein (alpha-mannosidase (EC 3.2.1.24)) | Glycosyl hydrolase superfamily protein |
| **Cellulase (glycosyl hydrolase family 5) protein** | **O-Glycosyl hydrolases family 17 protein** |
| O-Glycosyl hydrolases family 17 protein (endo-1,3-beta-glucosidase (EC 3.2.1.39); **lichenase** (EC 3.2.1.73); **exo-1,3-glucanase** (EC 3.2.1.58)). | **beta-1,3-glucanase**_putative |
| Glycosyl hydrolase family 10 protein (xylanase (EC 3.2.1.8); endo-1,3-beta-xylanase (EC 3.2.1.32); cellobiohydrolase (EC 3.2.1.91)). | **cellulase 3** |
| Glycosyl hydrolase superfamily protein (glycosyl hydrolase family protein 5 / cellulase family protein / (1-4)-beta-mannan endohydrolase) |  |
| **Glycosyl hydrolase family 81** protein (**beta-1,3-glucanases** EC 3.2.1.39)  The structure of a glycoside hydrolase family 81 endo-[beta]-1,3-glucanase  Endo-[beta]-1,3-glucanases catalyze the hydrolysis of [beta]-1,3-glycosidic linkages in glucans. They are also responsible for rather diverse physiological functions such as carbon utilization, **cell-wall organization** and pathogen defense. Glycoside hydrolase (GH) family 81 mainly consists of [beta]-1,3-glucanases from fungi, higher plants and bacteria. |  |
| Glycosyl hydrolase 9B13; endoglucanase (EC 3.2.1.4) and cellobiohydrolase (EC 3.2.1.91); **cellulase family E. Cellulases.** |  |
| Glycosyl hydrolase family protein; Glycoside hydrolase, family 3 (beta-glucosidase (EC 3.2.1.21); beta-xylosidase (EC 3.2.1.37); **N-acetyl beta-glucosaminidase** (EC 3.2.1.52); glucan beta-1,3-glucosidase (EC 3.2.1.58); **cellodextrinase** (EC 3.2.1.74); **exo-1,3-1,4-glucanase** (EC 3.2.1). |  |
| beta glucosidase 16 (13); **beta glucosidase 16** (BGLU16); FUNCTIONS IN: cation binding, hydrolase activity, hydrolyzing O-glycosyl compounds, |  |
|  |  |
|  | **cellulose synthase like G2** |
|  | **cellulose synthase like E1** |
|  | **cellulose synthase 1** |
|  |  |
| **Subtilase family protein**; FUNCTIONS IN: identical protein binding, serine-type endopeptidase activity; INVOLVED IN: response to cadmium ion, proteolysis; **located in cell wall**, extracellular region. |  |
| Subtilisin-like serine endopeptidase family protein; **located in cell wall, extracellular region** |  |
|  |  |
| **Pectin lyase-like** superfamily protein; cell wall organization | **Pectinacetylesterase** family protein |
| **Pectin lyase-like superfamily protein;** cell wall organization | **Plant invertase/pectin methylesterase inhibitor** superfamily protein |
| Plant **invertase**/**pectin methylesterase** inhibitor superfamily; **PECTIN METHYLESTERASE 39**, PME39; INVOLVED IN**: cell wall modification** | **Pectin lyase-like** superfamily protein. |
| **Plant invertase/pectin methylesterase** inhibitor superfamily protein **(embryo sac development arrest 24 (EDA24)** | **Plant invertase/pectin methylesterase** inhibitor superfamily protein |
| **Pectate lyase** family protein | **Plant invertase/pectin methylesterase** inhibitor superfamily protein |
|  | **Pectin lyase-like** superfamily protein |
|  |  |
| Encodes a Golgi apparatus-localized protein whose expression of the CGR3 gene is correlated with that of **several cell wall biosynthetic genes** **and that may be involved in pectin modifications.** | **VANGUARD 1 HOMOLOG 2, VGDH2; VANGUARD 1 homolog 2 (VGDH2); enzyme inhibitor activity, pectinesterase activity; cell wall modification, pectin catabolic process** |
|  | TBL38, **TRICHOME BIREFRINGENCE-LIKE 38**; Encodes a member of the TBL (TRICHOME BIREFRINGENCE-LIKE) gene family containing a plant-specific DUF231 (domain of unknown function) domain. TBL gene family has 46 members, two of which (TBR/AT5G06700 and TBL3/AT5G01360) have been shown to be involved in the **synthesis and deposition of secondary wall cellulose, presumably by influencing the esterification state of pectic polymers.** |
|  | **L-fucokinase/GDP-L-fucose pyrophosphorylase**; Encodes a bifunctional enzyme that has both L-fucokinase and GDP-L-fucose pyrophosphorylase activities. It catalyzes the two steps of the L-fucose salvage pathway for the generation of activated GDP-L-fucose. **This pathway seems to be of minor importance for cell wall polysaccharide biosynthesis** compared to the de novo GDP-L-fucose biosynthesis pathway in Arabidopsis. |
|  | **fucosyltransferase 7** |
|  |  |
| UDP-XYL synthase 6; **UDP-D-glucuronate carboxy-lyase-related** | **UDP-D-glucuronate 4-epimerase 2** |
| **Rhamnogalacturonate lyase** family protein | **Galactosyltransferase family protein** |
| **O-fucosyltransferase** family protein; located in Golgi apparatus |  |
|  |  |
| **purple acid phosphatase 10** |  |
|  |  |
| ATCWINV6, **CWINV6** |  |
|  |  |
| **Bifunctional inhibitor/lipid-transfer protein/seed storage 2S albumin superfamily protein** | **Bifunctional inhibitor/lipid-transfer protein/seed storage 2S albumin superfamily protein** |
| **Bifunctional inhibitor/lipid-transfer protein/seed storage 2S albumin superfamily protein** | **Bifunctional inhibitor/lipid-transfer protein/seed storage 2S albumin superfamily protein** |
| **Bifunctional inhibitor/lipid-transfer protein/seed storage 2S albumin superfamily protein.** |  |
|  |  |
| **Pollen Ole e 1 allergen and extensin family protein** | **Proline-rich extensin-**like family protein |
|  | **Pollen Ole e 1 allergen and extensin** family protein |
|  | **Proline-rich** family protein |
|  | **similar to Proline-rich extensin-**like family protein [Arabidopsis thaliana] |
|  | **Proline-rich extensin**-like family protein |
|  | **Proline-rich extensin-like** family protein |
|  | **ATPERK1, PERK1, PROLINE-RICH EXTENSIN-LIKE RECEPTOR KINASE 1**; Encodes a member of the proline-rich extensin-like receptor kinase (PERK) family. |
|  |  |
|  | **Glycine-rich** protein |
|  | **Glycine-rich** protein. |
|  |  |
|  | **hydroxyproline-rich** glycoprotein family protein |
|  | **hydroxyproline-rich** glycoprotein family protein |
|  | **hydroxyproline-rich** glycoprotein family protein |
|  |  |
|  | **beta-amylase 3** |
|  | **isoamylase 1** |
|  |  |
| **PDLP6, PLASMODESMATA-LOCATED PROTEIN 6**; Encodes a plasmodesmal protein that may be involved in the intercellular movement of molecules through the plasmodesmata. The protein has two DUF26 domains and a single transmembrane domain. |  |
| **PDLP4, PLASMODESMATA-LOCATED PROTEIN 4**; Encodes a plasmodesmal protein that may be involved in the intercellular movement of molecules through the plasmodesmata. The protein has two DUF26 domains and a single transmembrane domain. |  |
| **ATREM4.1, REM4.1, REMORIN GROUP 4 1**; Remorin family protein; CONTAINS InterPro DOMAIN/s: Remorin, C-terminal (InterPro:IPR005516); BEST Arabidopsis thaliana protein match is: Remorin family protein (TAIR:AT2G41870.1). |  |
|  |  |
| Peroxidase superfamily protein; PEROXIDASE 71; Encodes a cell wall bound peroxidase that is induced by hypo-osmolarity and is involved in the **lignification of cell walls.** |  |
| **Laccase 14** |  |
| 4-coumarate-CoA ligase activity; AMP-dependent synthetase and ligase family protein |  |
| ATCAD3, CAD3, CINNAMYL ALCOHOL DEHYDROGENASE HOMOLOG 3; **lignin biosynthetic process, o**xidation-reduction process |  |
| AR2, ATR2, P450 REDUCTASE 2; Encodes NADPH-cytochrome P450 reductase that catalyzes the first oxidative step of the **phenylpropanoid general pathway**. The mRNA is cell-to-cell mobile. |  |
| UDP-GLUCOSYL TRANSFERASE 73C6, UGT73C6; The At2g36790 gene encodes a **UDP-glucose:flavonol-3-O-glycoside-7-O-glucosyltransferase** (UGT73C6) attaching a glucosyl residue to the 7-O-position of the **flavonols kaempferol, quercetin** and their 3-O-glycoside derivatives. |  |
| ARABIDOPSIS THALIANA CHORISMATE MUTASE 1, ATCM1, CHORISMATE MUTASE 1, CM1; L-ascorbate peroxidase; **aromatic amino acid family biosynthetic process.** |  |
|  |  |
| **Receptor like protein 40**; **expressed in guard cell** | ATBCAT-2, BCAT-2, BCAT2, **BRANCHED-CHAIN AMINO ACID TRANSAMINASE 2,** BRANCHED-CHAIN AMINO ACID TRANSFERASE 2; Encodes a chloroplast branched-chain amino acid aminotransferase. Complements the yeast leu/iso-leu/val auxotrophy mutant. **Involved in cell wall development. plant-type cell wall organization or biogenesis** |
| **Wall associated kinase-like 4**; Encodes a cell-wall associated kinase like protein of the receptor-like kinase (RLK) superfamily. Likely involved in Arabidopsis root mineral responses to Zn2+, Cu2+, K+, Na+ and Ni+ |  |
| **Wall associated kinase-like 1** |  |
| **Wall associated kinase-like 7** |  |
| **Arabinose kinase** |  |
|  |  |
| TUB9, **TUBULIN BETA-9 CHAIN**; **microtubule-based process**, protein polymerization, response to cadmium ion. | **Actin depolymerizing factor 8** |
| TCAP1, CAP 1, CAP1, CYCLASE ASSOCIATED PROTEIN 1; CYCLASE ASSOCIATED PROTEIN; **actin cytoskeleton organization**, **unidimensional cell growth**. | **Actin-related protein C2B** |
| **Actin-binding FH2/DRF autoregulatory protein**; FUNCTIONS IN: actin binding; INVOLVED IN: cellular component organization, **actin cytoskeleton organization.** | **Myosin heavy chain-related** |
| **Myosin heavy chain-related** protein | **Tubulin beta-1 chain** |
| **Myosin heavy chain-related** protein | **Phragmoplast-associated kinesin-related protein**, putative |
| **Myosin heavy chain-related** protein |  |
| **Actin-binding FH2/DRF autoregulatory protein** |  |
| **Kinesin like protein for actin based chloroplast movement 1** |  |
| **Dynamin related** protein |  |
| **Cyclase associated protein 1; actin cytoskeleton organization, unidimensional cell growth** |  |
|  |  |
| **PHYTOHORMONE-RELATED-DEVELOPMENT-ABIOTIC STRESS** | |
| **ABA-STOMATAL OPENING** | |
| **INDUCED** | **REPRESSED** |
| **Golgi snare 12**; ER to Golgi vesicle-mediated transport, intra-Golgi vesicle-mediated transport, regulation of vesicle targeting, to, from or within Golgi, **response to abscisic acid**, vesicle fusion. | **Flowering time control protein-related** / FCA gamma-related, RNA binding; **abscisic acid binding.** |
| **SLAC1 HOMOLOGUE 3**, SLAH3; Encodes a protein with ten predicted transmembrane helices. The SLAH3 protein has similarity to the SLAC1 protein **involved in ion homeostasis in guard cells.** Although it is not expressed in guard cells, it can complement an slac1-2 mutant suggesting that it performs a similar function. SLAH3:GFP localizes to the plasma membrane. | ATLPP2, ATPAP2, LIPID PHOSPHATE PHOSPHATASE 2, LPP2, **PHOSPHATIDIC ACID PHOSPHATASE 2**; Encodes phosphatidic acid phosphatase. **Involved in ABA signaling.** Functions as a negative regulator upstream of ABI4. Expressed during germination and seed development. Expressed overall in young seedlings, in roots, hypocotyls, and vascular cells of cotyledons and leaves of 10 day-old seedlings, in flower filaments and stem elongation zones. Not expressed in anthers, pollen nor petals**. Abscisic acid-activated signaling pathway,** phospholipid dephosphorylation, phospholipid metabolic process. |
| **ABI FIVE BINDING PROTEIN 2,** AFP2; Encodes a member of a small plant-specific gene family whose members interact with ABI5 and appear to be involved in mediating stress responses. AFP2 mutants affect a number of ABA mediated processes such as germination and response to osmotic and sugar stress. AFP2 nuclear localization is stress dependent. **Response to abscisic acid, response to water deprivation, signal transduction.** |  |
|  |  |
| **DEHYDRATION-SALT-OSMOTIC-OXIDATIVE-STRESS-INDUCED PROTEINS** | |
| **Late embryogenesis abundant** protein**, group 2.** | **CBL-INTERACTING PROTEIN KINASE 19**, CIPK19, SNF1-RELATED PROTEIN KINASE 3.5, SNRK3.5; Encodes a member of the **SNF1-related kinase (SnRK) gene family (SnRK3.5),** which has also been reported as a member of the CBL-interacting protein kinases (CIPK19). |
| **Late embryogenesis abundant (LEA) hydroxyproline-rich glycoprotein family; group 2; defence response; antimicrobial peptide activity; defense/immunity protein activity; physiological defense response.** | **SOS3-interacting protein 4**; CBL-INTERACTING PROTEIN KINASE 11, CIPK11, PKS5, PROTEIN KINASE SOS2-LIKE 5, SIP4, **SNF1-RELATED PROTEIN KINASE 3.22**, SNRK3.22, SOS3-INTERACTING PROTEIN 4; Encodes a SOS2-like protein kinase that is a member of the CBL-interacting protein kinase family. **Loss of function mutants show a decrease in sensitivity to high pH.** Phosphorylates AHA2, a plasma membrane H+ ATPase.This phosphorylation appears to regulate the activity of the proton transporter. |
| **Late embryogenesis abundant (LEA) hydroxyproline-rich glycoprotein family; group 2**; Arabidopsis thaliana gene AT5G21130, encoding **harpin-induced 1 family** member. | **CYANASE**, CYN; Encodes a cyanase that catalyzes the bicarbonate-dependent breakdown of cyanate to ammonia and bicarbonate. CYN forms a hexadecamer and is believed to be a cytosolic protein. **Long-term exposure to NaCl increases CYN transcript levels.** It is also expressed at higher levels in flowers relative to stems, roots, and seedlings. **response to salt stress.** |
| **Late embryogenesis abundant (LEA)** **hydroxyproline-rich glycoprotein family** (TAIR:AT4G01410.1); expressed **in guard cell**. | **Isoflavone reductase, putative**, oxidation-reduction process, response to cadmium ion, **response to oxidative stress.** |
| **SITE-1 protease**; S1P appears to function as **a Golgi-localized subtilase and to help protect seedlings against salt and osmotic stress**. The roots of s1p-3 mutants **are hypersensitive to NaCl, KCl, LiCl, and mannitol**. Several salt-stress responsive genes show weaker induction in an s1P-3 mutant background. The proteolytic cleavage of the bZIP17 transcription factor depends on S1P in vitro. And there is evidence that S1P **can cleave bZIP17 *in vitro***. | CLT2, **CRT (CHLOROQUINE-RESISTANCE TRANSPORTER)-LIKE TRANSPORTER 2**; Encodes one of the CRT-Like transporters (CLT1/AT5G19380, CLT2/AT4G24460, CLT3/AT5G12170). **Required for glutathione homeostasis and stress responses**. Mutants lacking these transporters are heavy metal-sensitive, glutathione(GSH)-deficient, and **hypersensitive to *Phytophthora* infection.** |
| **HEXOKINASE-LIKE 3, HKL3**; carbohydrate phosphorylation, cellular glucose homeostasis, cellular response to DNA damage stimulus, glycolytic process, response to UV-B, response to cold, response to heat, response to osmotic stress, response to oxidative stress, response to salt stress, **response to water deprivation;** **is downregulated by response to hypoxia.** | **Early-responsive to dehydration stress protein (ERD4)** |
| **Diacylglycerol kinase 4** (DGK4); FUNCTIONS IN: diacylglycerol kinase activity; **INVOLVED IN: activation of protein kinase C activity by G-protein coupled receptor protein signaling pathway.** | **CBL-INTERACTING PROTEIN KINASE 17**, CIPK17, **SNF1-RELATED PROTEIN KINASE 3.21**, SNRK3.21; Encodes a member of the SNF1-related kinase (SnRK) gene family (SnRK3.21), which has also been reported as a member of the **CBL-interacting protein kinases (CIPK17).** |
| **Drought-responsive family protein**; Drought-responsive family protein; INVOLVED IN: response to water deprivation. | ATPCR2, PCR2**, PLANT CADMIUM RESISTANCE 2**; PCR2 encodes a membrane protein involved **in zinc transport and detoxification**. Response to oxidative stress. |
| PRP38 family protein; ATSRL1; encodes a putative splicing factor. Over-expression in yeast and Arabidopsis **result in increased tolerance to high salt.** | **NMRA-like negative transcriptional regulator** family protein; **isoflavone reductase. Involved in response to oxidative stress.** The mRNA is cell-to-cell mobile. |
| SIMILAR TO RCD ONE 1, SRO1; **embryo development, lateral root morphogenesis, photoperiodism, flowering, response to osmotic stress, response to oxidative stress, response to salt stress.** | **Putative lysine decarboxylase family protein** |
| ATLACS7, LACS7, LONG-CHAIN ACYL-COA SYNTHETASE 7; **Encode peroxisomal long-chain acyl-CoA synthetase**. Activates fatty acids for further metabolism. Interacts with PEX5. Fatty acid metabolic process, long-chain fatty acid metabolic process, **response to ozone, response to salt stress.** |  |
|  |  |
| **Glucose-methanol-choline (GMC) oxidoreductase family protein**; FUNCTIONS IN: aldehyde-lyase activity, oxidoreductase activity, acting on CH-OH group of donors, FAD binding; **INVOLVED IN: response to salt stress.** |  |
| ATMSH1, **CHLOROPLAST MUTATOR**, CHM, CHM1, MSH1, MUTL PROTEIN HOMOLOG 1; **response to UV, response to heat.** |  |
| **FAR1-RELATED SEQUENCE 1**, FRS1; FAR1-related sequence 1 (FRS1); FUNCTIONS IN: zinc ion binding; INVOLVED IN: **response to red or far red light**. |  |
| **Methionine aminopeptidase 2A** |  |
| ATLACS7, LACS7, **LONG-CHAIN ACYL-COA SYNTHETASE 7**; Encode **peroxisomal long-chain acyl-CoA synthetase**. Activates fatty acids for further metabolism. Interacts with PEX5. Fatty acid metabolic process, **long-chain fatty acid metabolic process, response to ozone, response to salt stress.** |  |
| Putative **lysine decarboxylase family protein** |  |
| **Copper amine oxidase** family protein |  |
| **Blue-copper-binding protein** |  |
| **LOW-TEMPERATURE-INDUCED 65**, LTI65, RD29B, RESPONSIVE TO DESICCATION 29B; **Encodes a protein that is induced in expression in response to water deprivation such as cold, high-salt, and desiccation. The response appears to be via abscisic acid.** The promoter region contains two ABA-responsive elements (ABREs) that are required for the dehydration-responsive expression of rd29B as cis-acting elements. Protein is a member of a gene family with other members found plants, animals and fungi. **Abscisic acid-activated signaling pathway, leaf senescence, response to abscisic acid, response to cold, response to salt stress, response to water deprivation.** |  |
|  |  |
| **AUXIN** | |
| ARF23, **AUXIN RESPONSE FACTOR 23**; auxin response factor 23 (ARF23); **auxin-activated signaling pathway.** | Encodes LAX2 (**LIKE AUXIN RESISTANT**), a member of the **AUX1 LAX family of auxin influx carriers.** Required for the establishment of embryonic root cell organization. Amino acid transport, **auxin-activated signaling pathway**, cotyledon vascular tissue pattern formation, response to nematode, root cap development. |
| **SAUR12, SMALL AUXIN UPREGULATED RNA 12**; **SAUR-like auxin-responsive** protein family. | **Auxin efflux carrier family protein.** |
| ATNCED2, NCED2, **NINE-CIS-EPOXYCAROTENOID DIOXYGENASE 2**; Encodes 9-cis-epoxycarotenoid dioxygenase, **a key enzyme in the biosynthesis of abscisic acid.** The expression of this gene declines during the first 12h of imbibition. | **SAUR-like auxin-responsive protein family.** |
| ARF9, **AUXIN RESPONSE FACTOR 9**; **auxin-activated signaling pathway**. | **Auxin-responsive GH3 family protein** |
| **LATERAL ROOT PRIMORDIUM 1, LRP1**; A member of SHI gene family. Arabidopsis thaliana has ten members that encode proteins with a RING finger-like zinc finger motif. Despite being highly divergent in sequence, many of the SHI-related genes are partially redundant in function and synergistically promote gynoecium, **stamen and leaf development in Arabidopsis**. Expressed in lateral root primordia **and induced by auxin**. SWP1 is involved in the repression of LRP1 via histone deacetylation. **Auxin biosynthetic process, auxin-activated signaling pathway, multicellular organismal development, response to auxin, root development.** | **Indole-3-acetic acid inducible 34** |
|  | **SAUR-like auxin-responsive protein family** |
|  |  |
|  |  |
|  |  |
|  |  |
| **BRASSINOSTEROIDS- GIBBERELLINS** | |
| ABCC13, ATABCC13, ATMRP11, **ATP-BINDING CASSETTE C13**, MRP11, MULTIDRUG RESISTANCE-ASSOCIATED PROTEIN 11; Encodes ABCC13/MRP11, a member of the multidrug resistance associated protein MRP/ABCC subfamily. **Its expression is induced by gibberellic acid and downregulated by naphthalene acetic acid, abscisic acid, and zeatin.** |  |
| **CYTOCHROME P450, FAMILY 716**, SUBFAMILY A, POLYPEPTIDE 1", CYP716A1; brassinosteroid biosynthetic process, **brassinosteroid homeostasis**, multicellular organismal development, sterol metabolic process. |  |
| **BRASSINOSTEROID-SIGNALING KINASE 2**, BSK2; Encodes BR-signaling kinase 2 (BSK2), one of the three homologous BR-signaling kinases (BSK1, AT4G35230; BSK2, AT5G46570; BSK3, AT4G00710). Mediates signal transduction from receptor kinase BRI1 by functioning as the substrate of BRI1. Plasma membrane localized**; brassinosteroid mediated signaling pathway.** |  |
|  |  |
|  |  |
|  |  |
| **CYTOKININS** | |
| AHP4, **HPT PHOSPHOTRANSMITTER 4**; Encodes AHP4, a histidine-containing phosphotransmitter involved in Histidine (His)-to-Aspartate (Asp) phosphorelay signal transduction. AHP4 is one of the six Arabidopsis thaliana histidine phosphotransfer proteins (AHPs). AHPs function as redundant **positive regulators of cytokinin signaling**. | **Cytokinin response factor 2.** |
| ATIPT2, IPPT, IPT2, TRNA, **ISOPENTENYLTRANSFERASE 2**; **cytokinin biosynthetic** process, tRNA modification. | **ARABIDOPSIS THALIANA SULFOTRANSFERASE 4B,** ATST4B, ST4B, SULFOTRANSFERASE 4B; Encodes a sulfotransferase. Unlike the related ST4A protein (At2g14920), in vitro experiements show that this enzyme does not act as brassinosteroids. ST4B is expressed in the roots and **transcript levels rise in response to cytokinin treatment**. |
|  | ATLOG1, LOG1, **LONELY GUY 1**; LONELY GUY 1 (LOG1); CONTAINS InterPro DOMAIN/s: Conserved hypothetical protein CHP00730 (InterPro:IPR005269); BEST Arabidopsis thaliana protein match is: **lysine decarboxylase family** protein; **cytokinin biosynthetic process.** |
|  | **Response regulator 7**; Encodes a member of the Arabidopsis response regulator (ARR) family, most closely related to ARR15. A two-component response regulator protein containing a phosphate-accepting domain in the receiver domain but lacking a DNA binding domain in the output domain. Involved in **response to cytokinin** and meristem stem cell maintenance. **Arr7 protein is stabilized by cytokinin**. |
|  | **Ureide permease 5** |
|  | **Purine permease 9** |
|  |  |
| **ETHYLENE-JASMONIC ACID** | |
| **Ethylene-dependent gravitropism-deficient and yellow-green-like 2**; S2P-like **putative metalloprotease**, also contain transmembrane helices near their C-termini and many of them, five of seven, contain a conserved zinc-binding motif HEXXH**. Homolog of EGY1. Each of the EGY1 and EGY-like proteins share** two additional highly conserved motifs, the previously reported NPDG motif (aa 442–454 in EGY1, Rudner et al., 1999) and a newly defined GNLR motif (aa 171–179 in EGY1). The GNLR motif is a novel signature motif unique to EGY1 and EGY-like proteins as well as other EGY1 orthologs found in cyanobacteria. | **Fatty acid desaturase 3; Endoplasmic reticulum enzyme responsible for the synthesis of 18:3 fatty acids from phospholipids. Uses cytochrome b5 as electron donor.** |
| ATS3, **SEED GENE 3**; Lipase/ l**ipooxygenase**. |  |
|  |  |
| **GROWTH-DEVELOPMENT-CELL DIVISION** | |
| **CITRATE SYNTHASE 2,** CSY2; Encodes a peroxisomal citrate synthase that is expressed throughout seedling and shoot development. | IMK3, **MERISTEMATIC RECEPTOR-LIKE KINASE**, MRLK  Protein kinase expressed in meristematic cells. Phosphorylates AGL24. |
| ATTLP6, TLP6, TUBBY LIKE PROTEIN 6; **phosphatidylinositol binding**, phosphoric diester hydrolase activity, **pollen development**, protein localization to cilium, regulation of transcription. | VAL3, **VP1/ABI3-LIKE 3**; **leaf development.** |
| FZL, FZO-LIKE; A new plant-specific member of the dynamin superfamily; defines a new protein class within the dynamin superfamily of **membrane remodeling GTPases** that regulates organization of the **thylakoid network in plants.** Targeted to chloroplasts and associated with thylakoid and envelope membranes as punctate structures. Knockout mutants have abnormalities in chloroplast and thylakoid morphology, including disorganized grana stacks and alterations in the relative proportions of grana and stroma thylakoids. Overexpression of FZL-GFP also conferred **defects in thylakoid organization.** | DEL1, DP-E2F-LIKE 1, E2F-LIKE 3, E2FE, E2L3; **E2F-like protein, an inhibitor of the endocycle,** **preserves the mitotic state of proliferating cells** by suppressing transcription of genes that are required for cells to enter the DNA endoreduplication cycle |
| ATTSO1, CHINESE FOR 'UGLY', TSO1**; floral organ morphogenesis**, regulation of cell division, regulation of meristem structural organization. | **Root hair specific 3** |
| **NO POLLEN GERMINATION RELATED 2**, NPGR2; **encodes a calmodulin-binding protein that is expressed in pollen, suspension culture cells, flowers, and fruits.** | **cyclin-dependent kinase D1; 3.** |
| MATERNAL EFFECT EMBRYO ARREST 38, **embryo development ending in seed dormancy.** | **Maternal effect embryo arrest 59** |
| ATWEE1, WEE1, **WEE1 KINASE HOMOLOG**; Protein kinase that negatively **regulates the entry into mitosis**. | **FAR1-related sequence 3** |
| **C-TERMINALLY ENCODED PEPTIDE 1**, CEP1; **Encodes CEP1, a 15-amino-acid peptide**, which is mainly expressed in the **lateral root primordia**. When overexpressed or externally applied, CEP1 arrests root growth. **CEP1 is a candidate for a novel peptide plant hormone.** | **Kinesin 5** |
| GAS41, GLIOMAS 41, HOMOLOG OF YEAST YAF9 A, TAF14B, TBP-ASSOCIATED FACTOR 14B, YAF9A; The GSA41 human homolog is expressed in nuclei and binds NuMA, a component of the nuclear matrix in interphase nuclei. **It negatively regulates flowering by controlling the H4 acetylation levels in the FLC and FT** **chromatin.** Histone H4-K5 acetylation, regulation of histone H4 acetylation, **regulation of photoperiodism, flowering, regulation of timing of transition from vegetative to reproductive phase, regulation of transcription**, DNA-templated. |  |
| CLE-LIKE 6, CLEL 6, GLV1, GOLVEN 1, RGF6, **ROOT MERISTEM GROWTH FACTOR 6**; Encodes a root meristem growth factor (RGF). Belongs to a family of functionally redundant homologous peptides that are secreted, tyrosine-sulfated, and expressed mainly in the stem cell area and the innermost layer of central columella cells. **RGFs are required for maintenance of the root stem cell niche and transit amplifying cell proliferation**. Members of this family include: At5g60810 (RGF1), At1g13620 (RGF2), At2g04025 (RGF3), At3g30350 (RGF4), At5g51451 (RGF5), At4g16515 (RGF6), At3g02240 (RGF7), At2g03830 (RGF8) and At5g64770 (RGF9). |  |
| ELF4-L1**, ELF4-LIKE 1**; **positive regulation of circadian rhythm, rhythmic process** |  |
| **SPIKE1, SPK1**; Encodes SPIKE1 (SPK1), the lone DOCK family guanine nucleotide exchange factor (GEF) in Arabidopsis. SPK1 is a peripheral membrane protein that accumulates at, and promotes the formation of, a specialized domain of the endoplasmic reticulum (ER) termed the ER exit site (ERES). **SPK1 promotes polarized growth and cell-cell adhesion in the leaf epidermis.** Mutant has seedling lethal; cotyledon, leaf-shape, **trichome defects**. **Auxin-activated signaling pathway,** multicellular organismal development, positive gravitropism, positive regulation of GTPase activity, regulation of actin polymerization or depolymerization, regulation of auxin mediated signaling pathway, **regulation of cell shape, regulation of cytoskeleton organization, vesicle-mediated transport.** |  |
|  |  |
| **SPX (SYG1/Pho81/XPR1) domain-containing protein** / zinc finger (C3HC4-type RING finger) protein-related; FUNCTIONS IN: zinc ion binding; **EXPRESSED IN: male gametophyte, flower, pollen tube.** |  |
| **Cyclin family** protein. |  |
| **Seed gene 3** |  |
| **3-KETOACYL-ACYL CARRIER PROTEIN SYNTHASE I**, KAS1, KASI, KETOACYL-ACP SYNTHASE 1; Encodes beta-ketoacyl-[acyl carrier protein] synthase I (KASI). Crucial for fatty acid synthesis**. Plays a role in chloroplast division and embryo development.** |  |
| **Maternal effect embryo arrest 38** |  |
| **Cyclin T1;1** |  |
| **Cell differentiation, Rcd1-like protein** |  |
| **ELF4-like 1: positive regulation of circadian rhythm**, rhythmic process |  |
|  |  |
|  |  |
| **TRANSCRIPTION FACTORS AND RELATED GENES** | |
| **INDUCED** | **REPRESSED** |
| Encodes **homeobox protein HAT9**. Regulation of transcription, DNA-templated, transcription, DNA-templated. | Homeobox-leucine zipper protein family; **Encodes homeobox protein HAT22**, member of the HD-Zip II family. The mRNA is cell-to-cell mobile; response to cytokinin |
| ATML1, MERISTEM LAYER 1; **Homeobox-leucine zipper family protein** / lipid-binding START domain-containing protein; | Homeobox-leucine zipper protein 4 (HB-4) / HD-ZIP protein |
| **Homeobox 12**, HB-12, multicellular organismal development, positive regulation of transcription, DNA-templated, regulation of transcription, DNA-templated, response to **abscisic acid, response to osmotic stress, response to salt stress, response to virus, response to water deprivation**, transcription, DNA-templated | **Homeodomain GLABROUS 12** |
| ARABIDOPSIS THALIANA INHIBITOR OF GROWTH 1, ATING1, ING1, INHIBITOR OF GROWTH 1; ING1 encodes a member of the Inhibitor of Growth family of nuclear-localized PhD domain containing **homeodomain** proteins. Binds to H3K4 di or trimethylated DNA. | **Homeodomain GLABROUS 8** |
|  | **Basic helix-loop-helix** (bHLH) DNA-binding family protein |
|  | **Basic helix-loop-helix** (bHLH) DNA-binding superfamily protein |
|  | **Basic helix-loop-helix** (bHLH) DNA-binding superfamily protein |
|  | AL2, **ALFIN-LIKE 2**; Encodes a member of the Alfin1-like family of nuclear-localized PHD (**plant homeodomain**) domain containing protein |
|  | **Basic helix-loop-helix, Nulp1-type** |
|  |  |
| **GATA TRANSCRIPTION FACTOR 23;** cell differentiation, lateral root development, response to light stimulus | **GATA** transcription factor 3 |
|  | **GATA type** zinc finger transcription factor family protein |
|  | **GATA type** zinc finger transcription factor family protein; **GATA transcription factor 17**; cell differentiation |
|  |  |
| **Myb domain protein 58**; Member of the R2R3 factor gene family. **Lignin biosynthetic process,** positive regulation of transcription, DNA-templated, **regulation of secondary cell wall biogenesis**, | **Myb domain protein 3r-4** |
| ATMYB117, **LATERAL ORGAN FUSION 1**, LOF1, MYB DOMAIN PROTEIN 117, **MYB117;** Encodes LOF1 (LATERAL ORGAN FUSION1), a MYB-domain transcription factor expressed in organ boundaries. Functions in boundary specification, meristem initiation and maintenance, and organ patterning | Arabidopsis thaliana **myb** **family transcription factor** |
| **Myb** family transcription factor; BEST Arabidopsis thaliana protein match is: Duplicated homeodomain-like superfamily protein | **Myb** **domain protein 92**; **response to jasmonic acid, response to salicylic acid** |
| ARABIDOPSIS THALIANA **MYB DOMAIN PROTEIN 80**, ATMYB103, ATMYB80, MALE STERILE 188, MS188, MYB DOMAIN PROTEIN 103, MYB103, MYB80; **Encodes a member of the R2R3 MYB transcription factor gene family** that is required for anther development by regulation tapetum development, callose dissolution and exine formation. It acts upstream of MS2 | **Myb-like** HTH transcriptional regulator family protein; Homeodomain-like superfamily protein |
| HTH transcriptional regulator, **Myb-type**, DNA-binding (InterPro:IPR017930); BEST Arabidopsis thaliana protein match is: Duplicated homeodomain-like superfamily protein. | **Myb domain protein 90** |
| **ATMYB99**, ATMYBCU15, **MYB DOMAIN PROTEIN 99**, MYB99 | **Myb domain protein 18; LAF1 is a R2R3-MYB transcription factor and positive regulator of the phyA photoresponse. Interaction of LAF1 with HFR1 stabilize the proteins against ubiquitination by COP1** |
| **Myb-like HTH transcriptional regulator family protein;** FUNCTIONS IN: DNA binding; INVOLVED IN: regulation of transcription; EXPRESSED IN: **guard cell;** TRF-like 5 |  |
|  |  |
| **Encodes WRKY** **transcription factor 2,** a zinc-finger protein. In wrky2 mutants, egg cells polarize normally but zygotes fail to reestablish polar organelle positioning from a transient symmetric state, resulting in equal cell division and distorted embryo development; establishment of cell polarity, longitudinal axis specification, pollen development, | **WRKY** **DNA-binding protein 27; ; nitric oxide mediated signal transduction.** |
| **ATWRKY59**, WRKY DNA-BINDING PROTEIN 59, WRKY59 | **WRKY DNA-binding protein 50; Involved in jasmonic acid inducible defense responses.** |
| WRKY DNA-BINDING PROTEIN 3, **WRKY3** | **WRKY DNA-binding protein 46; Encodes a WRKY transcription factor that contributes to the feed-forward inhibition of osmotic/ salt stress-dependent LR inhibition via regulation of ABA signaling and auxin homeostasis.** |
| **WRKY family transcription factor** | **WRKY DNA-binding protein 57; Activation of WRKY57 confers drought tolerance.** |
|  |  |
|  | **NAC** (No Apical Meristem) domain transcriptional regulator superfamily protein |
|  | **NAC domain containing protein 60** |
|  | **NAC domain containing protein 17** |
|  | **NAC domain containing protein 11** |
|  | **NAC domain protein 66** |
|  | **NAC domain containing protein 102** |
|  | **NAC domain containing protein 12** |
|  |  |
| **AGAMOUS-LIKE 61, AGL61, DIA, DIANA;** Encodes a member of the Agamous-like family of transcription factors. Localized to the nucleus in the central cell and endosperm of the female gametophyte. Loss of function mutations show reduced female fertility. | **AGAMOUS-like 23** |
| **AGAMOUS-like 89 (AGL89);** FUNCTIONS IN: sequence-specific DNA binding transcription factor activity; INVOLVED IN: regulation of transcription, DNA-dependent; LOCATED IN: nucleus; EXPRESSED IN: female gametophyte | MADS-box transcription factor family protein **AGAMOUS-like 80** |
| **AGAMOUS-LIKE 76, AGL76** | MADS-box transcription factor family protein |
|  |  |
|  | **Dof-type** zinc finger domain-containing protein |
|  | **Dof-type** zinc finger DNA-binding family protein |
|  |  |
| **NUCLEAR FACTOR Y**, **SUBUNIT A9**, NF-YA9; embryo development, microgametogenesis, negative regulation of transcription, DNA-templated, pollen development, regulation of transcription, DNA-templated, seed development, somatic embryogenesis, | **Nuclear factor Y, subunit C3** |
| **NUCLEAR FACTOR Y**, **SUBUNIT C13**, NF-YC13 | **Nuclear factor Y, subunit B2** |
|  |  |
| **ATE2FB, E2F TRANSCRIPTION FACTOR 1, E2F1, E2FB; Member of the E2F transcription factors,** (cell cycle genes), key components of the cyclin D/retinoblastoma/E2F pathway. Binds DPA and RBR1 proteins. Expressed throughout the cell cycle. Abundance increased by auxin through stabilization of the protein. Elevates CDK levels and activity, even under hormone-free conditions. Promotes cell division and shortens cell doubling time, inhibits cell growth. Transgenic plants overexpressing AtE2Fa contained an increased level of AtE2Fb transcripts that is paralleled by an increase in the amount of the AtE2Fb protein, suggesting that AtE2Fb expression might actually be up-regulated by the AtE2Fa transcription factor. |  |
|  |  |
| Encodes NIN Like Protein 7 (NLP7). **Modulates nitrate sensing and metabolism**. Mutants of NLP7 show features of nitrogen-starved plants and are tolerant to drought stress. Localized in the nucleus and f**unctions as a putative transcription factor**. The mRNA is cell-to-cell mobile. |  |
| RING/U-box protein with **C6HC-type zinc finger** |  |
| **BSD domain (BTF2-like transcription factors**, Synapse-associated proteins and DOS2-like proteins) |  |
|  | **General transcription factor 2**-related zinc finger protein TTF-type zinc finger protein with HAT dimerisation domain |
| **Transcription factor IIS** family protein | **General transcription factor 2-related** zinc finger protein |
| **NmrA-like negative transcriptional regulator** family protein | **GLOBAL TRANSCRIPTION FACTOR GROUP E2, GTE2** |
|  |  |
|  | **Transcriptional factor B3 family protein** |
|  | Transcription factor DP |
|  | **GRAS family transcription factor** |
|  |  |
|  | **NGA3, NGATHA3; AP2/B3**-like transcriptional factor family protein; flower development, leaf development. |
|  |  |
|  | ARID1, AT-RICH INTERACTING DOMAIN 1; Encodes a transcriptional activator that is involved in pollen development. ARID1 is expressed in nuclear bodies of microspore, **vegetative and generative cells**, and binds to and activates DUO during microgametogenesis. |
|  |  |
|  | **Heat shock transcription factor A4A**; Encodes a member of Heat Stress Transcription Factor(Hsf) family that is **a substrate of the MPK3/MPK6 signaling** and regulates stress responses. |
|  |  |
|  | **CONSTANS-like 2** |
|  |  |
|  | **HMG (high mobility group) box protein** with ARID/BRIGHT DNA-binding domain |
|  | **Sequence-specific DNA binding transcription factors** |
|  |  |
|  | **TSPL6, SPL6, SQUAMOSA PROMOTER BINDING PROTEIN (SBP)-DOMAIN** **TRANSCRIPTION FACTOR 6**; Encodes SPL6. Required for the resistance mediated by the TIR-NB-LRR RPS4 against *Pseudomonas syringae* carrying the avrRps4 effector. Transcriptome analysis indicates that SPL6 positively regulates a subset of defense genes. |
|  | **Squamosa promoter-binding protein-like (SBP domain) transcription factor** family |
|  |  |
|  | **Winged helix-turn-helix transcription repressor DNA-binding** |
|  | **DNA-binding storekeeper protein-related transcriptional regulator** |
|  | **Bromodomain transcription factor** |
|  |  |
|  |  |
| **TRANSPORT** | |
| **INDUCED** | **REPRESSED** |
| **Sugar transporter 1;** carbohydrate transmembrane transporter activity, sugar: **proton symporter activity; expressed in carpel, flower, flower pedicel, guard cell.** | **Sugar transporter 14** |
| **ATSWEET7, SWEET7;** Nodulin MtN3 family protein; LOCATED IN: endomembrane system, integral to membrane, membrane; EXPRESSED IN: 9 plant structures; expressed during: 4 anthesis, C globular stage, petal differentiation and expansion stage, E expanded cotyledon stage: Nodulin MtN3 family protein; **carbohydrate transport.** |  |
|  |  |
| **Major facilitator** superfamily protein**; expressed in guard cell.** | **Major facilitator** superfamily protein; functions in: carbohydrate transmembrane transporter activity, **sugar: hydrogen symporter activity** |
| **Major facilitator** superfamily protein**; carbohydrate transmembrane transporter activity, transporter activity, sugar: hydrogen symporter activity.** | **Major facilitator** superfamily protein; involved in: **oligopeptide transport**. |
| **Major facilitator** superfamily protein; **oligopeptide transport; expressed in guard cell.** | **Major facilitator** superfamily protein; involved in **nitrate assimilation, oligopeptide transport, transport** |
| **Major facilitator** superfamily protein; **oligopeptide transport** | **Major facilitator** superfamily protein; involved in **dipeptide transport, nitrate transport, oligopeptide transport.** |
|  |  |
| **Plasma membrane intrinsic protein 2E (PIP2E)**: **water channel** activity; INVOLVED **IN: transport, response to nematode.** |  |
| **PIP1;4, PIP1E, PLASMA MEMBRANE INTRINSIC PROTEIN 1;4, PLASMA MEMBRANE INTRINSIC PROTEIN 1E, TMP-C, TRANSMEMBRANE PROTEIN C**; plasma membrane intrinsic protein 1E;4; response to water deprivation, transport, **water transport.** |  |
| NIP7;1, NLM6, NLM8, **NOD26-LIKE INTRINSIC PROTEIN 7;1**, NOD26-LIKE MIP 6, NOD26-LIKE MIP 8; Encodes NIP7;1, an anther-**specific boric acid transporter** of **the aquaporin superfamily regulated by an unusual tyrosine in helix 2 of the transport pore; water channel activity.** |  |
| **Plasma membrane intrinsic protein 2E** |  |
| **Plasma membrane intrinsic protein 1; 4** |  |
|  |  |
| **MATE efflux** family protein; antiporter activity. | **MATE efflux** family protein; **antiporter activity**, **response to nematode.** |
|  | **MATE efflux** family protein; **antiporter activity, guard cell.** |
|  | **MATE efflux** family protein; FERRIC REDUCTASE DEFECTIVE 3; FRD3 is likely to function in **root xylem loading of an iron chelator** or other factor necessary for efficient iron uptake out of the xylem or apoplastic space and into leaf cells; cellular response to **ethylene stimulus**, **iron ion**, **nitric oxide**, and **abscisic acid.** |
|  | **MATE efflux** family protein; antiporter activity. |
|  |  |
|  | **Multidrug resistance-associated protein 7** |
|  | **Drug/metabolite transporter** superfamily protein |
|  |  |
| **Mitochondrial substrate carrier** family protein. | **Mitochondrial import inner membrane translocase subunit Tim17/Tim22/Tim23** family protein. |
|  | **Mitochondrial substrate carrier** family protein |
|  | **Mitochondrial acyl carrier protein 2** |
|  | **Translocase of the outer mitochondrial membrane 40** |
|  |  |
| **Heavy metal transport/**detoxification superfamily protein**.** | **Magnesium transporter CorA-like family protein** |
| **Vacuolar iron transporter (VIT) family** protein | **ZIP metal ion transporter** family. |
| **Cobalt ion transmembrane transporters** | **Magnesium (Mg) transporter 10** |
|  | **High-affinity nickel-transport family protein** |
|  | **Zinc transporter 5** precursor |
|  | **Aluminium-activated malate transporter** family protein. |
|  | **IRON-REGULATED PROTEIN 1, ATIREG1, FERROPORTIN 1, FPN1, IREG1, IRON REGULATED 1; cobalt and iron ion transmembrane transport** |
|  |  |
|  | **ABC-2 and Plant PDR ABC-type transporter** family protein |
|  |  |
|  | **Nodulin MtN21 /EamA-like transporter** family protein. |
|  | **Nodulin-like / Major Facilitator** Superfamily protein |
|  |  |
| **Autoinhibited Ca2+ -ATPase**, isoform 8; **calcium-transporting ATPase**, putative. | **Solute: sodium symporters; urea transmembrane transporters.** |
| **ATCHX16, CATION/ H+ EXCHANGER 16, CHX16**; **cation transport, hydrogen ion transmembrane transpor**t, regulation of pH, **sodium ion transmembrane** transport. | **Cation exchanger 5** |
| ATCLC-B, **CHLORIDE CHANNEL B**, CLC-B; member of Anion channel protein family The mRNA is cell-to-cell mobile; anion transmembrane transport, chloride transmembrane transport, chloride transport, hydrogen ion transmembrane transport**, regulation of anion transmembrane transport.** | **Cation exchanger 11** |
| **Calcium-transporting ATPase**, putative |  |
|  |  |
| **Nucleotide-sugar** transporter family protein | **Nucleotide-diphospho-sugar transferases** superfamily protein. |
|  | **Nucleotide-diphospho-sugar transferases** superfamily protein |
|  | **Nucleotide-diphospho-sugar transferases** superfamily protein |
|  |  |
| **Nitrate transporter 2.2** | **Transmembrane amino acid transporter** family protein. |
| **Peptide transporter 3** | **Transmembrane amino acid transporter** family protein |
|  | **Transmembrane amino acid transporter** family protein |
|  | **Transmembrane amino acid transporter** family protein |
|  | LHT1, **LYSINE HISTIDINE TRANSPORTER 1**; Encodes LHT1 (lysine histidine transporter), a high-affinity transporter for **cellular amino acid uptake** in both root epidermis and leaf mesophyll. |
|  | **Lysine histidine transporter 2; amino acid, ACCO transport.** |
|  | **Oligopeptide transporter 1** |
|  |  |
|  | **Mechanosensitive channel of small conductance-like 4** |
|  |  |
| **Sec14p-like phosphatidylinositol transfer** family protein. | **Sec14p-like phosphatidylinositol** transfer family protein. |
| **Sec14p-like phosphatidylinositol transfer** family protein. | **Sec14p-like phosphatidylinositol** transfer family protein |
| **Sec14p-like phosphatidylinositol transfer** family protein | **Arv1-like protein; intracellular sterol transport, regulation of plasma membrane sterol distribution, sphingolipid and sterol metabolic process.** |
|  |  |
| ORP3C, **OSBP(OXYSTEROL BINDING PROTEIN)-RELATED PROTEIN 3C**; **lipid transport**, steroid metabolic process | **Polyketide cyclase/dehydrase and lipid transport** superfamily protein. |
| **Polyketide cyclase/dehydrase and lipid transport** superfamily protein. | **Polyketide cyclase/dehydrase and lipid transport** superfamily protein |
| **Polyketide cyclase/dehydrase and lipid transport** superfamily protein |  |
|  |  |
| **COP1-interacting protein-related**. | **ASAR1, ATSAR2, ATSARA1C, SAR2, SECRETION-ASSOCIATED RAS SUPER FAMILY 2; A member of ARF-like GTPase family.** *A thaliana* has 21 members, in two subfamilies, **ARF and ARF-like (ARL) GTPases; intracellular protein transport, vesicle-mediated transport.** |
| **ER lumen protein retaining receptor** family protein. | **Sec23/ sec24 transport family protein** |
| **Vesicle associated protein** | **Sec1/mnc18-like** (SM) proteins superfamily. |
| **Target SNARE coiled-coil domain protein** | **Golgi transport complex protein**-related |
| **Membrane trafficking VPS53** family protein | **Importin alpha isoform 5** |
| **ROP interactive partner 2** | **SecY protein transport family protein.** |
| **ER lumen protein retaining receptor** family protein | **Coatomer gamma-2 subunit, putative** / gamma-2 coat protein, putative / gamma-2 COP, putative. |
|  | **Bacterial sec-independent translocation protein mttA/Hcf106**  subunit |
|  | **Secretion-associated RAS super family 2** |
|  | **Adaptor protein complex AP-1**, gamma |
|  |  |
| **Vacuolar protein sorting-associated protein VPS28 family protein** | **Vacuolar protein sorting 41** |
|  | **Vacuolar sorting protein 9 (VPS9) domain** |
|  |  |
|  | Encodes a component of the **TIC** (translocon at the inner envelope membrane of chloroplasts) **protein translocation machinery mediating the protein translocation across the inner envelope of plastids**. The Arabidopsis genome encodes four Tic20 homologous proteins, AT1G04940(Tic20-I), AT2G47840(Tic20-II), AT4G03320(Tic20-IV) and AT5G55710(Tic20-V). |
|  |  |
|  | **Nuclear transport factor 2 (NTF2) family protein** |
|  |  |
| **Sulfite exporter TauE/SafE** family protein | **S-adenosylmethionine carrier 2** |
|  |  |
|  | **ADP/ATP carrier 3** |
|  |  |
|  |  |
| **SIGNAL TRANSDUCTION** | |
| **INDUCED** | **REPRESSED** |
| **Calcium-dependent lipid-binding (CaLB domain)** family protein. | **Calcium-binding EF-hand family protein** |
| **Calmodulin-binding protein-related** | **Calcium-binding EF hand family protein** |
|  | **Calcium-binding EF-hand family protein** |
|  |  |
| SKM1**, STERILITY-REGULATING KINASE MEMBER** 1; **Leucine-rich receptor-like protein kinase** family protein. | **Leucine-rich repeat transmembrane protein kinase** |
| **NSP-interacting kinase 1,** **Leucine-rich receptor-like Protein kinase** family protein. | **Leucine-rich repeat protein kinase** family protein |
| **Leucine-rich repeat transmembrane protein kinase protein** | **Leucine-rich repeat protein kinase** family protein |
| **Leucine-rich repeat protein kinase** family protein | **Leucine-rich repeat protein kinase** family protein |
| **Leucine-rich repeat (LRR) family protein** | **Leucine-rich repeat protein kinase** family protein |
| **Leucine-rich repeat 2** | **Leucine-rich repeat protein kinase** family protein |
| **Leucine-rich repeat protein kinase family protein** | **Leucine-rich repeat transmembrane protein kinase** |
|  |  |
|  | **Receptor protein kinase-related.** |
|  | **Receptor like protein 2** |
|  |  |
| **FBD / Leucine Rich Repeat domains** containing protein; F-box/RNI-like/FBD-like domains-containing protein. | **FBD, F-box and Leucine Rich Repeat domains** containing protein. |
| **F-box/RNI-like/FBD-like domains-containing protein**; CONTAINS InterPro DOMAIN/s: **FBD, F-box domain, cyclin-like, FBD-like, F-box domain, Skp2-like.** |  |
|  |  |
|  | **Mannose-binding lectin** superfamily protein. |
|  | **Mannose-binding lectin superfamily protein** |
|  | **Mannose-binding lectin superfamily protein** |
|  |  |
| **Serinc-domain containing** serine and **sphingolipid biosynthesis protein** | **Serinc-domain containing serine and sphingolipid biosynthesis protein** |
| **SPla/RYanodine receptor (SPRY) domain**-containing protein. | **ARABIDOPSIS INOSITOL PHOSPHORYLCERAMIDE SYNTHASE 3**, ATIPCS3; Inositol phosphorylceramide synthase; **sphingolipid biosynthetic process.** |
| **SPla/RYanodine receptor (SPRY)** domain-containing protein. | Arabidopsis **Inositol phosphorylceramide synthase 3** |
|  |  |
| **UDP-Glycosyltransferase superfamily protein; glycosyltransferase family protein**; FUNCTIONS IN: transferase activity, **transferring glycosyl groups**; INVOLVED IN: metabolic process; LOCATED IN: endomembrane system; EXPRESSED IN: 18 plant structures. | **UDP-Glycosyltransferase** superfamily protein |
| SQD1, **SULFOQUINOVOSYLDIACYLGLYCEROL 1**; **involved in sulfolipid biosynthesis** The mRNA is cell-to-cell mobile. **Glycolipid biosynthetic process**. | **Galactosyltransferase** family protein |
| **Core-2/I-branching beta-1,6-N-acetylglucosaminyltransferase family** protein; CONTAINS InterPro DOMAIN/s: Core-2/I-Branching enzyme (InterPro:IPR021141); BEST Arabidopsis thaliana protein match is: **Core-2/I-branching beta-1,6-N-acetylglucosaminyltransferase** family protein. | **UDP-Glycosyltransferase** superfamily protein |
| **UDP-Glcnac-adolichol phosphate glcnac-1-p-transferase** | **UDP-Glycosyltransferase** superfamily protein |
| **Beta-1,3-N-Acetylglucosaminyltransferase** family protein | **UDP-glucosyl transferase 78D2** |
|  | **UDP-glucosyl transferase 84B1** |
|  |  |
| **Protein kinase family** protein | **Protein kinase** superfamily protein |
| **Protein kinase superfamily** protein | **Protein kinase** superfamily protein |
| **Protein kinase superfamily** protein | **Protein kinase** superfamily protein |
| **Protein kinase superfamily** protein | **Protein kinase** superfamily protein |
| **Protein kinase superfamily** protein | **Protein kinase** superfamily protein |
| **Protein kinase superfamily** protein | **Protein kinase** superfamily protein |
| **Protein kinase superfamily** protein | **Protein kinase** superfamily protein |
|  | **Protein kinase** superfamily protein |
|  | **Protein kinase** superfamily protein |
|  | **Protein kinase** superfamily protein |
|  | **Protein kinase** superfamily protein |
|  | **Protein kinase** superfamily protein |
|  | **Protein kinase** superfamily protein |
|  | **Protein kinase** superfamily protein |
|  | **Protein kinase** superfamily protein |
|  |  |
| **U-box domain-containing protein kinase** family protein  phosphofructokinase 1 | **Interleukin-1 receptor-associated kinase 4** protein |
| **Lectin protein kinase** family protein | **Calmodulin-domain protein kinase 7** |
| **Calcium-dependent protein kinase 1** | **Calcium-dependent protein kinase 6** |
|  | **S-locus lectin protein kinase** family protein |
|  | **Mitogen-activated protein kinase homolog** |
|  | **MAP kinase substrate 1** |
|  | **MAP kinase kinase 7** |
|  | **MAK10** homologue |
|  | **Uridine kinase-like 3** |
|  |  |
| ATPFA-DSP2, **PFA-DSP2**, PLANT AND FUNGI **ATYPICAL DUAL-SPECIﬁCITY PHOSPHATASE 2**; Encodes an atypical dual-speciﬁcity phosphatase. | **Histidine acid phosphatase** family protein |
| **Dual specificity protein phosphatase (DsPTP1)** family protein | **Phosphatidic acid phosphatase (PAP2)** family protein |
| **Phosphatidic acid phosphatase (PAP2)** family protein. | **Protein phosphatase 4** |
| **HAD superfamily, subfamily IIIB acid phosphatase.** | **3-phosphoserine phosphatase** |
| **Protein phosphatase 2C** family protein | **Lipid phosphate phosphatase 2** |
| **Protein phosphatase 2C** family protein | **PP2C induced by avrRPM1** |
| **Phosphotyrosine protein phosphatases** superfamily protein | **Metal-dependent phosphohydrolase** |
|  | **Type one serine/threonine protein phosphatase 3** |
|  |  |
|  | **Copine (Calcium-dependent phospholipid-binding protein)** family |
|  |  |
|  | **Plant self-incompatibility protein S1** family |
|  |  |
|  | **FK506- and rapamycin-binding protein 15 kD-2** |
|  | **Calcium-dependent lipid-binding (CaLB domain) plant phosphoribosyltransferase family protein** |
|  |  |
| GNL2**, GNOM-LIKE 2**; GNOM-like 2 (GNL2); FUNCTIONS IN: **ARF guanyl-nucleotide exchange factor activity.** | **Prenylated RAB acceptor 1.G1** |
| ATNUDT9, ATNUDX9, **NUDIX HYDROLASE HOMOLOG 9**, NUDT9, NUDX9; **Encodes a GDP-d-mannose pyrophosphohydrolase that is involved in the regulation of GDP-d-Man levels affecting ammonium sensitivity via modulation of protein N-glycosylation in the roots. Cellular response to ammonium ion** | **RAC-like GTP binding protein 5** |
| **AMP-dependent synthetase and ligase** family protein | **RAB GTPase homolog A1I** |
| **cAMP-regulated phosphoprotein 19-related** protein | **AMP-dependent synthetase and ligase** family protein Type one serine/threonine |
| **Guanyl-nucleotide exchange factors; GTPase binding;GTP binding** | **GTP cyclohydrolase I** |
|  | **AGC** (**cAMP-dependent, cGMP-dependent and protein kinase C**) kinase family protein  calmodulin binding;purine nucleotide binding. |
|  | **GTP cyclohydrolase I** |
|  | **GTP-binding family** protein |
|  | **GTP-binding protein-related** |
|  | **PRA1 (Prenylated rab acceptor**) family protein |
|  | **Guanylate kinase** |
|  | **Rho GTPase activating protein with PAK-box/P21-Rho-binding domain**  **GTP-binding family protein.** |
|  | **GPCR-type G protein 1** |
|  |  |
|  | **TRAM, LAG1 and CLN8** (TLC) **lipid-sensing domain containing protein** |
|  | **PEBP (phosphatidylethanolamine-binding protein)** family protein |
|  |  |
|  |  |
| **PROTEIN/ LIPASES/ HYDROLASES/ CHAPERONES** | |
| **INDUCED** | **REPRESSSED** |
| **RING/U-box** superfamily protein | **RING/U-box** superfamily protein |
| **RING/U-box** superfamily protein | **RING/U-box** superfamily protein |
| **RING/U-box** superfamily protein | **RING/U-box** superfamily protein |
| **RING/U-box** superfamily protein | **RING/U-box** superfamily protein |
| **RING/U-box** superfamily protein | **RING/U-box** superfamily protein |
|  | **RING/U-box** superfamily protein |
|  | **RING/U-box** superfamily protein |
|  | **RING/U-box** superfamily protein |
|  |  |
| **RING/U-box protein with C6HC-type zinc finger** | **RING/U-box protein with C6HC-type zinc finger** |
|  |  |
|  | **RING-H2 finger protein 2B** |
|  | **RING-H2 finger A1A** |
| **Eukaryotic aspartyl protease** family protein | **Eukaryotic aspartyl protease** family protein |
|  | **Eukaryotic aspartyl protease** family protein |
|  | **Eukaryotic aspartyl protease** family protein |
|  |  |
|  | **Metal-dependent protein hydrolase** |
|  |  |
| **Subtilase family** protein | **Subtilisin-like serine protease 3** |
| **Subtilisin-like serine endopeptidase** family protein |  |
|  |  |
|  | **Cysteine proteinases superfamily** protein |
|  | **Calpain-type cysteine protease family** |
|  |  |
| **Peptidase S24/S26A/S26B/S26C family protein**; proteolysis, **signal peptide** **processing**. | **Serine carboxypeptidase-like 31** |
| **Prolyl oligopeptidase family protein**; FUNCTIONS IN: serine-type peptidase activity, **serine-type endopeptidase activity**; INVOLVED IN: proteolysis. | **Peptidase S24**/S26A/S26B/S26C family protein |
| **Peptidase S24/S26A/S26B/S26C** family protein | **Serine carboxypeptidase-like 18** |
| **Prolyl oligopeptidase** family protein | **Aspartate aminotransferase 4** |
| **Prolyl oligopeptidase** family protein | **ATP-dependent Clp protease** |
| **Gamma-glutamyl transpeptidase 4** | **Proteolysis 6** |
| **Amidase family** protein | **Gamma-glutamyl hydrolase 2** |
| **Tyrosine transaminase** family protein | Similar to **Ulp1 protease family protein** [Arabidopsis thaliana] |
| **SITE-1 protease** | **Rhomboid-related intramembrane serine protease** family protein |
|  | **RHOMBOID-like 1** |
|  | **RHOMBOID-like protein 4** |
|  |  |
|  | **20S proteasome alpha subunit C1** |
|  |  |
| **Ubiquitin-like superfamily protein; autophagy, cellular response to nitrogen starvation**, protein transport. | **Ubiquitin-conjugating enzyme 35,** Ubiquitin carboxyl-terminal hydrolase family protein |
| **Ubiquitin-like superfamily** protein | **Ubiquitin-specific protease 27** |
| **Ubiquitin-like superfamily** protein | **Ubiquitin carboxyl-terminal hydrolase** family protein |
| **Ubiquitin-protein ligase 2** | **Ubiquitin-conjugating enzyme 6** |
|  | **Ubiquitin-specific protease 14** |
|  | **Ubiquitin carboxyl-terminal hydrolase**-related protein |
|  | **Ubiquitin family** protein |
|  | **Ovarian tumour, otubain**, **Ubiquitin thioesterase Otubain**, **Peptidase C65**, otubain. |
|  |  |
| **Ribosomal protein S28** | **Ribosomal protein L1p**/L10e family |
| **Ribosomal protein L34** | **Ribosomal protein S5 domain 2**-like superfamily protein |
| **Ribosomal L18p/L5e** | **Ribosomal protein L24e** family protein |
| **Ribosomal protein S30** family protein | **Ribosomal protein L7Ae/L30e/S12e/Gadd45** family protein |
| **Ribosomal protein L18e/L15** superfamily protein | **Ribosomal protein S7e** family protein |
| **Mitochondrial 28S ribosomal protein S29**-related | **Ribosomal protein L6** family protein |
|  | **Ribosomal protein S8** family protein |
|  | **Ribosomal protein L41** family |
|  | **Ribosomal L5P** family protein |
|  | **Ribosomal protein S27a** / Ubiquitin family protein |
|  | **Ribosomal protein L34e** superfamily protein |
|  | **Ribosomal protein L22p/L17e** family protein |
|  |  |
| **Eukaryotic translation initiation factor 3G2** | **Mitochondrial transcription termination** factor family protein |
| **Ribosome associated membrane protein RAMP4** | **Mitochondrial transcription termination factor** |
| **tRNA synthetase class** I (I, L, M and V) family protein | **Mitochondrial transcription termination** factor family protein |
| **Transcription elongation factor (TFIIS**) family protein | **Eukaryotic translation initiation factor 3A** |
| **tRNA isopentenyltransferase 2** | **GTP binding Elongation factor Tu** family protein |
|  | ATP binding; l**eucine-tRNA ligases;aminoacyl-tRNA ligases**; nucleotide binding. |
|  | **ATP binding; aminoacyl-tRNA ligases** |
|  | **Elongator protein 6** |
|  | Small **nuclear ribonucleoprotein** family protein |
|  | **Peptidyl-tRNA hydrolase II (PTH2) family protein** |
|  | **Translation elongation factor EFG/EF2 protein** |
|  | **Eukaryotic translation initiation factor 4G** |
|  | **Ribosomal protein L25**/**Gln-tRNA synthetase, anti-codon-binding domain** |
|  | **Alanine-tRNA ligases;nucleic acid binding;ligases, forming aminoacyl-tRNA and related compounds; nucleotide binding ;ATP binding** |
|  |  |
| **Heat shock protein DnaJ** **with tetratricopeptide repeat** | **Chaperone DnaJ-domain** superfamily protein |
| **DNAJ heat shock** family protein | **TCP-1/cpn60 chaperonin** family protein |
| **Chaperone DnaJ-domain** superfamily protein | **HSP20-like chaperone** |
| **HSP20-like chaperones** superfamily protein | **HSP20-like chaperones** superfamily protein |
| Putative **thiol-disulphide oxidoreductase** DCC | **Chaperone DnaJ-domain** superfamily protein |
|  | **HSP20-like chaperone** |
|  | **Cyclophilin-like peptidyl-prolyl cis-trans isomerase family protein** |
|  | Molecular **chaperone, heat shock protein, Hsp40, DnaJ**. |
|  |  |
| **GDSL-like Lipase/Acylhydrolase** superfamily protein | **GDSL-like Lipase/Acylhydrolase** superfamily protein |
| **GDSL-like Lipase/Acylhydrolase** superfamily protein | **GDSL-like Lipase/Acylhydrolase** superfamily protein |
|  |  |
|  | **SGNH hydrolase-type esterase superfamily protein** |
|  |  |
| **Alpha/beta-Hydrolases** superfamily protein. | **Alpha/beta-Hydrolases** superfamily protein |
|  | **Alpha/beta-Hydrolases** superfamily protein |
|  | **Alpha/beta-Hydrolases** superfamily protein |
|  | **Alpha/beta-Hydrolases** superfamily protein |
|  | **Alpha/beta-Hydrolases** superfamily protein |
|  | **Alpha/beta-Hydrolases** superfamily protein |
|  |  |
|  |  |
| **REDOX** | |
| **INDUCED** | **REPRESSED** |
| **Glutaredoxin family protein; N-terminal protein myristoylation, cell redox homeostasis.** |  |
| **Glutaredoxin** family protein |  |
|  |  |
| **Peroxidase** superfamily protein | **Peroxidase superfamily** protein |
|  | **Peroxidase** superfamily protein |
|  | Arabidopsis thaliana **PEROXYGENASE 2** |
|  |  |
| **Monodehydroascorbate reductase 4** | **monodehydroascorbate reductase 1** |
|  |  |
| **Oxoglutarate/iron-dependent oxigenase** | 2**-oxoglutarate (2OG) and Fe(II)-dependent oxygenase** superfamily protein |
| **2-oxoglutarate (2OG) and Fe(II)-dependent oxygenase** superfamily protein | **2-oxoglutarate (2OG) and Fe(II)-dependent oxygenase** superfamily protein |
| **2-oxoglutarate (2OG) and Fe(II)-dependent oxygenase** superfamily protein |  |
|  |  |
| **NAD(P)H dehydrogenase 18** | **FAD/NAD(P)-binding oxidoreductase** family protein |
| **FAD/NAD(P)-binding oxidoreductase** family protein | Root FNR 2; Encodes a root-type **ferredoxin:NADP(H) oxidoreductase**. |
|  |  |
| **Cytochrome P450** superfamily protein | **CYTOCHROME C-1** |
| **P450 reductase 2** | **Cytochrome P450, family 89**, subfamily A, polypeptide 5 |
|  | **Cytochrome P450, family 71**, subfamily B, polypeptide 9 |
|  | **Cytochrome P450, family 71**, subfamily B, polypeptide 32 |
|  | **cytochrome p450 79f1** |
|  |  |
|  | **L Thioredoxin** superfamily protein |
|  | **Thioredoxin superfamily** protein |
|  | **Thioredoxin z** |
|  |  |
|  | **Glutathione S-transferase TAU 18** |
|  | **glutathione S-transferase (class zeta) 2** |
|  |  |
|  | **Metallothionein 2** |
|  | **Acetate/malate dehydrogenase** family protein |
|  | **Ferric reduction oxidase 1** |
|  | **Germin-like protein 8** |
|  |  |
| **Methionine sulfoxide reductase** (MSS4-like) family protein |  |
|  |  |
|  |  |
| **PRIMARY-SECONDARY METABOLISM** | |
| **INDUCED** | **REPRESSED** |
| **Terpenoid cyclases/Protein prenyltransferases** superfamily protein | **Terpenoid cyclases family protein** |
| **Terpene synthase-like sequence-1,8-**cineole | **Terpenoid cyclases/Protein prenyltransferases superfamily protein** |
|  | Putative **pentacyclic triterpene synthase 3** |
|  | **Beta-carotene hydroxylase 2** |
|  |  |
| **LACS8, LONG-CHAIN ACYL-COA SYNTHETASE 8; AMP-binding, conserved site.** | **Saposin B domain-containing protein; lipid metabolic process** |
| **FatA acyl-ACP thioesterase**; fatty acid biosynthetic process |  |
| **Long-chain acyl-CoA synthetase 7** |  |
|  |  |
| **Glutamate decarboxylase 5** |  |
| Class I **glutamine amidotransferase-like** superfamily protein |  |
|  |  |
| **6-1-fructan exohydrolase** | **Dihydroxyacetone kinase** |
| **PfkB-like carbohydrate kinase** family protein | **Carbohydrate-binding X8** domain superfamily protein |
| **Aldolase-type TIM barrel** family protein | **Carbohydrate-binding X8 domain** superfamily protein |
| **Glucose-methanol-choline (GMC) oxidoreductase** family protein | **Galactose mutarotase-like** superfamily protein |
|  | **Glucose-6-phosphate dehydrogenase** |
|  | **Mannose-6-phosphate isomerase, type I** |
|  | **6-phosphogluconate dehydrogenase** family protein |
|  |  |
| **Cinnamyl alcohol dehydrogenase homolog 3** |  |
| **Chorismate mutase** 1 |  |
|  |  |
| **S-adenosyl-L-methionine-dependent methyltransferases** superfamily protein | **S-adenosyl-L-methionine-dependent methyltransferases** superfamily protein |
| **Methyltransferases** | **S-adenosyl-L-methionine-dependent methyltransferases** superfamily protein |
|  | **S-adenosyl-L-methionine-dependent methyltransferases** superfamily protein |
|  | **S-adenosyl-L-methionine-dependent methyltransferases** superfamily protein |
|  | **S-adenosyl-L-methionine-dependent methyltransferases** superfamily protein  **S-adenosyl-L-methionine-dependent methyltransferases** superfamily protein |
|  | **S-adenosyl-L-methionine-dependent methyltransferases** superfamily protein |
|  | **S-adenosyl-L-methionine-dependent methyltransferases** superfamily protein |
|  | **Putative methyltransferase** family protein |
|  | **Methyltransferase FkbM** |
|  | **Methyl esterase 19** |
|  | **O-methyltransferase** family protein |
|  | **Methyl esterase 5** |
|  |  |
| **HXXXD-type acyl-transferase** family protein | **HXXXD-type acyl-transferase** family protein |
| **Acyl-activating enzyme 15** | **Acyl-activating enzyme 18** |
|  | **Acyl-CoA N-acyltransferases (NAT)** superfamily protein |
|  | **Transferases**, transferring acyl groups |
|  | **O-acyltransferase (WSD1-like**) family protein |
|  | **Acyl-CoA N-acyltransferase with RING/FYVE/PHD-type zinc finger** domain |
|  |  |
|  | **Pyridoxine biosynthesis 1.2** |
|  | **Pyridoxamine 5'-phosphate oxidase** family protein |
|  | **Pyridoxal phosphate (PLP)-dependent transferases superfamily protein; carbon-sulfur lyase activity** |
|  | **Formyltetrahydrofolate deformylase**, putative |
|  |  |
|  | **Cyanase** |
|  | **Nitrilase/cyanide hydratase and apolipoprotein N-acyltransferase** family protein |
|  | **Nitrile specifier protein 2; glucosinolate catabolic process, nitrile biosynthetic process** |
|  | **Nitrile specifier protein 5; glucosinolate catabolic process, nitrile biosynthetic process** |
|  |  |
| **Dihydropterin pyrophosphokinase** / Dihydropteroate synthase | **Cytidine/deoxycytidylate deaminase** family protein |
| **4-hydroxy-3-methylbut-2-enyl diphosphate synthase** | **ACT domain-containing small subunit of acetolactate synthase** protein |
|  | **Arogenate dehydratase 1** |
|  | **Phosphoribosylaminoimidazole carboxylase**, putative / AIR carboxylase, putative |
|  |  |
|  |  |
| **CHLOROPLAST/ MITOCHONDRIA-PHOTOSYNTHESIS-ENERGY** | |
| **GENES NECESSARY FOR THE ACHIEVEMENT OF RUBISCO ACCUMULATION 5**, NARA5; Encodes a **phosphofructokinase B-type** **carbohydrate kinase family** protein, NARA5. **Regulates photosynthetic gene expression.** |  |
|  |  |
| **ATP synthase protein I** –related | **ATPase, V1 complex**, subunit B protein |
|  | **ATPase, V0 complex, subunit E** |
|  | **Vacuolar ATP synthase subunit C (VATC)** / V-ATPase C subunit / **vacuolar proton pump C subunit (DET3)** |
|  | **Vacuolar proton ATPase A2** |
|  | **Gamma subunit of Mt ATP synthase** |
|  | **Autoinhibited Ca2+-ATPase 11** |
|  | **ATP-citrate lyase A-2** |
|  |  |
| **Photosystem II reaction center PsbP family protein** |  |
| **Photosystem I subunit F** |  |
| **Photosystem I P subunit** |  |
| **PsbQ-like 1;** photosynthesis, light reaction, photosynthetic electron transport chain. |  |
|  | **High chlorophyll fluorescence 153** |
|  | **High chlorophyll fluorescent 109** |
|  | **Phytochelatin synthase 1 (PCS1)** |
|  |  |
|  | **Beta carbonic anhydrase 6** |
|  | **Cytochrome c oxidase 19-1** |
|  |  |
|  |  |
| **DNA REPAIR-HISTONE MODIFICATIONS-RNA MODIFICATIONS** | |
| **INDUCED** | **REPRESSED** |
| **AGO5, ARGONAUTE 5, ATAGO5; defense response, incompatible interaction, response to virus, viral gene silencing in virus induced gene silencing.** | **Rad23 UV excision repair** protein family |
| ATUPF3, **UPF3**; **Involved in mRNA surveillance**, detects exported mRNAs with truncated open reading frames and initiates nonsense-mediated mRNA decay (NMD); **Defense response to bacterium**, **mRNA transport**, **nuclear-transcribed catabolic process,** nonsense-mediated decay. | **Rad23 UV excision repair** protein family |
| CEN2, **CENTRIN 2**; Encodes a member of the Centrin family. Mutants are **hypersensitive to UV and prone to UV induced DNA damage**. Based on sequence similarity and mutant phenotype CEN2 is thought to be involved in nucelotide excision repair/**DNA repair**. | **DNA repair metallo-beta-lactamase** family protein |
| **Argonaute** family protein |  |
|  |  |
| Putative endonuclease or glycosyl hydrolase with C2H2-type zinc finger domain  **DEAD-like helicase**. | **RECQ helicase L2** |
| **RNA helicase**, **ATP-dependent, SK12/DOB1** protein |  |
| **Helicase/SANT-associated, DNA binding protein** |  |
|  |  |
| **Polynucleotidyl transferase**, **ribonuclease H-like** superfamily protein | **RNase H** family protein |
| similar to **RNase H domain-containing** protein | **Ribonuclease H-like** superfamily protein |
| **Polynucleotidyl transferase,** **ribonuclease H-like superfamily** protein | **Ribonuclease PH45A** |
| **Polynucleotidyl transferase**, **ribonuclease H-like** superfamily protein | **Polynucleotidyl transferase, ribonuclease H-like** superfamily protein |
|  | **HNH endonuclease** |
|  | **Ribonuclease 3** |
|  |  |
|  | **Histone superfamily** protein |
|  | **Histone superfamily** protein |
|  | **Histone superfamily** protein |
|  | **Histone H3 K4-specific methyltransferase SET7/9** family protein |
|  | **Methyltransferases; nucleic acid binding** |
|  |  |
|  |  |
| **Regulator of chromosome condensation (RCC1)** family protein |  |
| **Regulator of chromosome condensation (RCC1)** family protein |  |
|  |  |
| **MIR164/MIR164B; miRNA** | **MIR395B; miRNA** |
|  | **MIR395A; miRNA** |
|  |  |
|  | **Ribonucleotide reductase 1** |
|  | **Ribonucleotide reductase 2A** |
|  |  |
| **RNA-binding (RRM/RBD/RNP motifs)** family protein | **RNA-binding (RRM/RBD/RNP motifs)** family protein |
| **RNA-binding (RRM/RBD/RNP motifs**) family protein | **RNA-binding (RRM/RBD/RNP motifs)** family protein |
| **RNA-binding (RRM/RBD/RNP motifs)** family protein | **RNA-binding (RRM/RBD/RNP motifs)** family protein |
| **RNA-binding (RRM/RBD/RNP motifs**) family protein | **RNA-binding (RRM/RBD/RNP motifs)** family protein |
| **RNA-binding (RRM/RBD/RNP motifs)** family protein | **RNA binding** |
|  |  |
| **Pre-mRNA-processing-splicing factor** | **pre-mRNA-processing protein 40A** |
|  | **Mitochondrial RNA-editing factor 1** |
|  | **Mitochondrial editing factor 20; Encodes a pentatricopeptide repeat protein (PPR) protein involved in mitochondrial mRNA editing.** |
|  | Ortholog of maize **chloroplast splicing factor CRS1** |
|  |  |
| **RNA polymerase II, Rpb4,** core protein | **RNA polymerase Rpb7 N-terminal domain**-containing protein |
| **DNA polymerase V** family |  |
|  |  |
| **RNA 2'-phosphotransferase**, **Tpt1 / KptA family** | **Phosphoribosyltransferase** family protein |
|  | **Nucleotidyltransferase family** protein |
|  |  |
| **P-loop containing nucleoside triphosphate hydrolases** superfamily protein | **P-loop containing nucleoside triphosphate hydrolases** superfamily protein |
|  | **P-loop containing nucleoside triphosphate hydrolases** superfamily protein |
|  |  |
|  | **RNA binding; nucleic acid binding** |
|  | **Integrase-type DNA-binding** superfamily protein |
|  | **Integrase-type DNA-binding** superfamily protein |
|  |  |
| putative **endonuclease** or glycosyl hydrolase with C2H2-type zinc finger domain |  |
|  |  |
|  |  |
| **OTHERS** | |
| **INDUCED** | **REPRESSED** |
| Immunoglobulin E-set superfamily protein | Lung seven transmembrane receptor family protein |
| EXS (ERD1/XPR1/SYG1) family protein | Emp24/gp25L/p24 family/**GOLD family** protein |
| Ran BP2/NZF zinc finger-like superfamily protein | Similar to DNA binding |
| Delta-adaptin | Telomere repeat binding factor 1 |
| NIN like protein 7 | DPP6 N-terminal domain-like protein |
| CBS domain-containing protein with a domain of unknown function (DUF21) | YGGT family protein |
| PRP38 family protein | Bromo-adjacent homology (BAH) domain-containing protein |
| Nudix hydrolase homolog 9 | Sporulation 11-2 |
| MUTL protein homolog 1 | Anaphase-promoting complex/cyclosome 11 (TAIR:AT3G05870.2) |
| similar to RCD one 1 | Neurochondrin family protein |
| LisH and RanBPM domains containing protein | NC domain-containing protein-related |
| PAR1 protein | VIRB2-interacting protein 2 |
| DHHC-type zinc finger family protein | MMS ZWEI homologue 1 |
| Gag-Pol-related retrotransposon family protein | Homolog of yeast ergosterol28 |
| U5 small nuclear ribonucleoprotein helicase | AAR2 protein family |
| Centrin 2 | Mini zinc finger 1 |
| Josephin family protein | Tubby like protein 7 |
| Crooked neck protein, putative / cell cycle protein, putative | SMAD/FHA domain-containing protein |
| IBR domain-containing protein | HOPW1-1-interacting 1 |
| AZA-guanine resistant1 | Reticulon family protein |
| CAP160 protein | Transmembrane proteins 14C |
| HCP-like superfamily protein with MYND-type zinc finger | Reticulon family protein |
| Smg-4/UPF3 family protein | Caleosin-related family protein |
| D111/G-patch domain-containing protein | GINS complex protein |
| YEATS family protein | AWPM-19-like family protein |
| MuDR family transposase | Paralog of ARC6 |
| Lumazine-binding family protein | **Timeless family protein** |
| RGPR-related | Tic22-like family protein |
| C-terminal cysteine residue is changed to a serine 2 | Evolutionarily conserved C-terminal region 8 |
| Telomerase activating protein Est1 | regulatory particle non-ATPase 12A |
| CCT motif family protein | **EXORDIUM like 4** |
| **Remorin** family protein | **Cullin** family protein |
| INO80 ortholog | MIF4G domain-containing protein |
| Emp24/gp25L/p24 family/GOLD family protein | **Yippee family** putative **zinc-binding protein** |
| Formin homologue 4 | MALE GAMETOPHYTE DEFECTIVE 2 |
| RAD3-like DNA-binding helicase protein | ArfGap/RecO-like **zinc finger domain**-containing protein |
| F-box family protein | Protein with RNI-like/FBD-like domains |
| VQ motif-containing protein | PYR1-like 10 |
| like COV 2 | MIF4G domain-containing protein / MA3 domain-containing protein |
| Caleosin-related family protein | TGACG motif-binding factor 6 |
| Phox (PX) domain-containing protein | Exocyst subunit exo70 family protein C1 |
| Paired amphipathic helix (PAH2) superfamily protein | Sas10/Utp3/C1D family |
| Beige/BEACH domain ;WD domain, G-beta repeat protein | Pumilio 17 |
| **Zinc finger, C3HC4 type (RING finger)** family protein | U2 small nuclear ribonucleoprotein A |
| NHL domain-containing protein | TRF-like 3 |
| Prohibitin 3 | BSD domain-containing protein |
| SPX (SYG1/Pho81/XPR1) domain-containing protein / zinc finger (**C3HC4-type RING finger**) protein-related | VIRB2-interacting protein 1 |
| TLD-domain containing nucleolar protein | PLAC8 family protein |
| **Haloacid dehalogenase-like hydrolase (HAD)** superfamily protein | TOXICOS EN LEVADURA 63 |
|  | DHHC-type zinc finger family protein |
|  | Las1-like family protein |
|  | Sirohydrochlorin ferrochelatase B |
|  | **Zinc finger (C2H2 type)** family protein |
|  | ARID/BRIGHT DNA-binding domain;ELM2 domain protein |
|  | Serine/arginine-rich 22 |
|  | Exocyst subunit exo70 family protein H5 |
|  | **B-box type zinc finger protein** with CCT domain |
|  | Survival protein SurE-like phosphatase/nucleotidase |
|  | **Nudix hydrolase homolog 4** |
|  | IQ-domain 27 |
|  | ENTH/VHS family protein |
|  | ALA-interacting subunit 5 |
|  | Exostosin family protein |
|  | TBP-associated factor II 15 |
|  | Homolog of yeast autophagy 18 (ATG18) H |
|  | KAR-UP F-box 1 |
|  | SKU5 similar 2 |
|  | Zinc finger (CCCH-type) family protein |
|  | General control non-repressible 3 |
|  | HIT zinc finger ;PAPA-1-like conserved region |
|  | DP-E2F-like 1 |
|  | BRCT domain-containing DNA repair protein |
|  | EXS (ERD1/XPR1/SYG1) family protein |
|  | BREVIS RADIX-like 1 |
|  | Alfin-like 2 |
|  | Pyrimidine 1 |
|  | Sterile alpha motif (SAM) domain-containing protein |
|  | Outer membrane OMP85 family protein. |
|  | SIN3-like 6 |
|  | Breast basic conserved 1 |
|  | Dentin sialophosphoprotein-related |
|  | PYR1-like 13 |
|  | Similar to nucleic acid binding / zinc ion binding [Arabidopsis thaliana] |
|  | Reticulon family protein |
|  | **AT hook motif DNA-binding family** protein |
|  | **Ovate family protein 1** |
|  | **HMG (high mobility group) box protein** with ARID/BRIGHT DNA-binding domain |
|  | **Haloacid dehalogenase-like hydrolase** **(HA**D) superfamily protein |
|  |  |
| **Ankyrin repeat** family protein | **Ankyrin repeat** family protein |
|  | **Ankyrin repeat** family protein |
|  |  |
| **ARM repeat** superfamily protein | **ARM repeat superfamily** protein |
|  | **ARM repeat** superfamily protein |
|  | **ARM repeat** superfamily protein |
|  | **ARM repeat** superfamily protein |
|  | **CCCH-type zinc finger protein with ARM repeat** domain |
|  |  |
|  | **BTB and TAZ domain** protein 2 |
|  | **BTB/POZ domain-containin**g protein |
|  | **BTB/POZ domain with WD40/YVTN repeat**-like protein |
|  | **TRAF-like family** protein |
|  |  |
| **Cysteine/Histidine-rich C1 domain** family protein | **Cysteine/Histidine-rich C1 domain** family protein |
| **Cysteine/Histidine-rich C1 domain** family protein | **Cysteine/Histidine-rich C1 domain** family protein |
|  | **Cysteine/Histidine-rich C1 domain** family protein |
|  | **Cysteine/Histidine-rich C1 domain** family protein |
|  | **Cysteine/Histidine-rich C1 domain** family protein |
|  | **Cysteine/Histidine-rich C1 domain** family protein |
|  | **Cysteine/Histidine-rich C1 domain** family protein |
|  | **Cysteine/Histidine-rich C1 domain** family protein |
|  |  |
| **F-box** and associated interaction domains-containing protein | **F-box** and associated interaction domains-containing protein |
| **F-box** and associated interaction domains-containing protein | **F-box** family protein |
| **F-box** and associated interaction domains-containing protein | **F-box** and associated interaction domains-containing protein |
| **F-box** and associated interaction domains-containing protein | **F-box** and associated interaction domains-containing protein |
| **F-box** and associated interaction domains-containing protein | **F-box** and associated interaction domains-containing protein |
| **F-box** family protein | **F-box** and associated interaction domains-containing protein |
| **F-box** family protein | **F-box/RNI-like** superfamily protein |
|  | **F-box family** protein |
|  |  |
| **Galactose oxidase/kelch repeat** superfamily protein | **Galactose oxidase/kelch repeat** superfamily protein |
| **Galactose oxidase/kelch repeat** superfamily protein | **Galactose oxidase/kelch repeat** superfamily protein |
| **Galactose oxidase/kelch repeat** superfamily protein |  |
|  |  |
| **NAD(P)-binding Rossmann-fold** superfamily protein | **NAD(P)-binding Rossmann-fold** superfamily protein |
|  | **NAD(P)-binding Rossmann-fold** superfamily protein |
|  |  |
|  |  |
|  | **Nucleic acid-binding, OB-fold-like** protein |
|  | **Nucleic acid-binding, OB-fold-like** protein |
|  | **Nucleic acid-binding, OB-fold-like** protein |
|  |  |
| **Pentatricopeptide repeat (PPR)** superfamily protein | **Pentatricopeptide repeat (PPR)** superfamily protein |
| **Pentatricopeptide repeat (PPR)** superfamily protein | **Pentatricopeptide repeat (PPR)** superfamily protein |
| **Pentatricopeptide repeat (PPR-like)** superfamily protein | **Pentatricopeptide repeat (PPR**) superfamily protein |
| **Pentatricopeptide repeat (PPR-like)** superfamily protein | **Pentatricopeptide repeat (PPR)** superfamily protein |
|  | **Pentatricopeptide repeat (PPR)** superfamily protein |
|  |  |
| **RING/FYVE/PHD zinc finger** superfamily protein | **RING/FYVE/PHD zinc finger** superfamily protein |
|  | **RING/FYVE/PHD zinc finger** superfamily protein |
|  | **RING/FYVE/PHD zinc finger** superfamily protein |
|  | **RING/FYVE/PHD zinc finger superfamily** protein |
|  | **Zinc finger C-x8-C-x5-C-x3-H type** |
|  | **CHY-type/CTCHY-type/RING-type Zinc finger** protein |
|  | **Zinc finger (CCCH-type)** family protein |
|  | **Zinc finger, C3HC4 type** (RING finger) family protein |
|  |  |
|  | **RNI-like** superfamily protein |
|  | **RNI-like superfamily** protein |
|  |  |
| **Tetratricopeptide repeat (TPR)-like** superfamily protein | **Tetratricopeptide repeat (TPR)-like** superfamily protein |
| **Tetratricopeptide repeat (TPR)-like** superfamily protein | **Tetratricopeptide repeat (TPR)-like** superfamily protein |
| **Tetratricopeptide repeat (TPR)-like** superfamily protein | **Tetratricopeptide repeat (TPR)-containing** protein |
|  | **Tetratricopeptide repeat (TPR)-like** superfamily protein |
|  | **Tetratricopeptide repeat (TPR)-like** superfamily protein |
|  | **Tetratricopeptide repeat (TPR)-like** superfamily protein |
|  | **Tetratricopeptide repeat (TPR)-like** superfamily protein |
|  |  |
| **Transducin/WD40 repeat-like** superfamily protein | **Transducin/WD40 repeat-like** superfamily protein |
| **Transducin/WD40 repeat-like** superfamily protein | **Transducin/WD40 repeat-like** superfamily protein |
|  | **Transducin/WD40 repeat-like** superfamily protein |
|  | **Transducin/WD40 repeat-like** superfamily protein |
|  | **Transducin/WD40 repeat-like s**uperfamily protein |
|  |  |
|  |  |
