# Supplementary material for: Overexpression of Grain Amaranth (Amaranthus hypochondriacus) AhERF or AhDOF Transcription Factors in Arabidopsis thaliana Increases Water Deficit- and Salt-Stress Tolerance, Respectively, via Contrasting Stress-Amelioration Mechanisms
Source: PLoS One. 2016 Oct 17;11(10):e0164280. doi: 10.1371/journal.pone.0164280 (PMC5066980; doi:10.1371/journal.pone.0164280)
Supplement: S3 Table — (DOCX) [file pone.0164280.s009.docx]

**S3 Table.** **List of genes with altered expression detected in transgenic *AhDOF-AI* overexpressing Arabidopsis plants under optimal conditions.**

| **DISEASE RESISTANCE** | |
| --- | --- |
| **INDUCED** | **REPRESSED** |
| **3.225966634** **Disease resistance-responsive (dirigent-like protein)** family protein | -6.275330869 **Pathogenesis-related gene 1** |
| **2.891396688** **Disease resistance protein** (**TIR-NBS-LRR class**) family | -4.077367543 Disease resistance protein (**CC-NBS-LRR class**) family |
| 2.880115401 **Cysteine/Histidine-rich C1 domain** family protein | -3.4420274 **Seven transmembrane MLO family protein** |
| 2.704250468 **Cysteine/Histidine-rich C1 domain** family protein | -3.2763523 **Cysteine/Histidine-rich C1** domain family protein |
| 2.66993171 Arabidopsis **defensin-like** protein | -2.961924007 **Receptor protein kinase-related** |
| 2.401987136 **Avirulence induced gene (AIG1)** family protein | -2.753196575 **Leucine-rich repeat transmembrane protein kinase** |
| 2.420088412 **Bax inhibitor-1** family protein | -2.616430345 **cysteine-rich RLK (RECEPTOR-like protein kinase) 2** |
| 2.348386718 **Cystatin B** | -2.545378111 **Receptor like protein 21** |
| 2.289707138 **Disease resistance-responsive (dirigent-like protein)** family protein | -2.491140646 **Cysteine/Histidine-rich C1 domain family protein** |
| 2.264777257 **Cysteine-rich RLK (RECEPTOR-like protein kinase) 31** | -2.474532212 **RPM1 interacting protein 2** |
| 2.101379625 **Disease resistance protein (CC-NBS-LRR class) family** | -2.442310333 **NIMA-related serine/threonine kinase 1** |
| 2.075024501 **Receptor-like protein kinase-related family** protein | -2.394414525 **mto 1 responding down 1** |
| 2.017770813 **Disease resistance protein (TIR-NBS-LRR class)** family | -2.340211336 **NIM1-interacting 3** |
|  | -2.318524685 **Toll-Interleukin-Resistance (TIR) domain** family protein |
|  | -2.280932016 **BAX inhibitor 1** |
|  | -2.272023086 **Cysteine/Histidine-rich C1 domain** family protein |
|  | -2.26994383 **Cysteine-rich RLK (RECEPTOR-like protein kinase) 32** |
|  | -2.165433725 **GRIM-19 protein** |
|  | -2.148877129 **Toll-Interleukin-Resistance (TIR)** domain family protein |
|  | -2.146638536 **Receptor-like protein kinase-related** |
|  | -2.097486677 **Cysteine/Histidine-rich C1 domain** family protein |
|  | -2.075092207 **Leucine-rich repeat protein kinase** family protein |
|  | -2.059113498 **Defender against death (DAD family)** protein |
|  | -2.026354948 **Disease resistance protein (TIR-NBS-LRR clas**s) |
|  | -2.012280216 **Disease resistance protein (TIR-NBS-LRR class) family** |
|  |  |
|  |  |

| **CELL WALL RELATED-SUGAR MODIFICATION-SYNTHESIS** | |
| --- | --- |
| **INDUCED** | **REPRESSED** |
| 3.069422751 **Glycosyl hydrolase superfamily** protein | -5.820590933 **TRICHOME BIREFRINGENCE-LIKE 38** |
| 3.058453223 **Sinapoylglucose 1** | -3.660244647 **Phloem protein 2 A5** |
| 2.868089618 **Bifunctional inhibitor/lipid-transfer protein/seed storage 2S albumin** superfamily protein | -3.277639602 **Cellulose synthase-like D5** |
| 2.81836919 **Leucine-rich repeat receptor-like protein kinase** family protein | -3.192741916 **Core-2/I-branching beta-1,6-N-acetylglucosaminyltransferase family protein** |
| 2.784502476 **Annexin 3** | -3.191574432 **Carbohydrate-binding X8 domain** superfamily protein |
| 2.778905387 **Leucine-rich receptor-like protein kinase** family protein | -3.178865933 **Plant invertase/pectin methylesterase inhibitor** superfamily |
| 2.745843486 **Fibrillarin family** protein | -3.113646959 **Extensin 3** |
| 2.741704373 **Plant invertase/pectin methylesterase inhibitor** superfamily protein | -3.040116889 **Carbohydrate-binding X8 domain** superfamily protein |
| 2.639707843 **Myosin heavy chain-related** protein | -2.875985363 **Hydroxyproline-rich glycoprotein** family protein |
| 2.600612683 **Beta-galactosidase 4** | -2.875096354 **Glycosyl hydrolase** superfamily protein |
| 2.549147522 **Protease inhibitor/seed storage/lipid transfer protein (LTP)** family protein | -2.855532645 **UDP-Glycosyltransferase** superfamily protein |
| 2.527129853 **Microtubule-associated proteins 70-2** | -2.8381087 **O-Glycosyl hydrolases family 17 protein** |
| 2.478926866 **Tubulin beta-9 chain** | -2.791478294 **Glycine-rich protein** |
| 2.363449437 **Core-2/I-branching beta-1,6-N-acetylglucosaminyltransferase** family protein | -2.771108689 **Extensin-like protein** |
| 2.321819016 **UDP-glucosyl transferase 74D1** | -2.738965067 **Hydroxyproline-rich** glycoprotein family protein |
| 2.323499805 **Actin binding Calponin homology (CH) domain-containing protein** | -2.718545504 **Bifunctional inhibitor/lipid-transfer protein/seed storage 2S albumin** superfamily protein |
| 2.313670858 **Leucine-rich repeat (LRR)** family protein | -2.533851707 **FBD / Leucine Rich Repeat domains** containing protein |
| 2.288196608 **Myosin heavy chain-related** | -2.48560205 **Myosin heavy chain-related** |
| 2.272175116 **Glucan synthase-like 8** | -2.45372334 **Actin-related protein 4** |
| 2.271579058 **Delta-adaptin** | -2.436294457 **Galactose-binding** protein |
| 2.242001804 **Plant invertase/pectin methylesterase inhibitor** superfamily protein | -2.401400951 **Pectin lyase-like** superfamily protein |
| 2.235664429 **Wall associated kinase-like 1** | -2.40508249 **FAD-binding Berberine** family protein |
| 2.213922213 **Glycine-rich protein** | -2.367739255 **Bifunctional inhibitor/lipid-transfer protein/seed storage 2S albumin** superfamily protein |
| 2.168139087 **Beta glucosidase 4** | -2.361722357 **COBRA-like protein 1 precursor** |
| 2.166346835 **Cellulase (glycosyl hydrolase family 5) protein** | -2.345684813 **Pectin lyase-like superfamily** protein |
| 2.15174813 **Hydroxyproline-rich glycoprotein** family protein | -2.342328752 **Leucine-rich repeat (LRR) family** protein |
| 2.118746939 **FASCICLIN-like arabinogalactan protein 18** precursor | -2.330655256 **Leucine-rich repeat protein kinase** family protein |
| 2.101342339 **UDP-Glycosyltransferase** superfamily protein | -2.306979345 **Beta-1,4-N-acetylglucosaminyltransferase family protein** |
| 2.092363475 **Exostosin family** protein | -2.281550577 **Glycine/proline-rich protein** |
| 2.090320226 **UDP-Glycosyltransferase** superfamily protein | -2.271854683 **Hydroxyproline-rich glycoprotein** family protein |
| 2.086522539 **Leucine-rich repeat protein kinase** family protein | -2.25487539 **FAD-binding Berberine** family protein |
| 2.086108133 **Actin 4** | -2.194548231 **Profilin 2** ; Encodes profilin2, a **low-molecular weight, actin monomer-binding protein that regulates the organization of actin cytoskeleton.** |
| 2.075993368 **Leucine-rich repeat (LRR) family** protein | -2.180535094 **Arabinogalactan protein 17** |
| 2.056937582 **Phloem protein 2-B8** | -2.156253234 **Bifunctional inhibitor/lipid-transfer protein/seed storage 2S albumin** superfamily protein |
| 2.042291282 **Pectin lyase-like** superfamily protein | -2.149834555 **Leucine-rich repeat protein kinase** family protein |
| 2.007878249 **KDO transferase A; structures of the pectic polysaccharide rhamnogalacturonan II (RG-II) pectin constituent.** | -2.133550291 **Arabinogalactan protein 21** |
|  | -2.113775132 **Core-2/I-branching beta-1,6-N-acetylglucosaminyltransferase** family protein |
|  | -2.055628578 **Glycine-rich protein** |
|  | -2.046840969 **Arabinogalactan protein 15** |
|  | -2.044934404 **Hydroxyproline-rich glycoprotein** family protein |
|  | -2.042507769 **Wall-associated kinase** family protein |
|  | -2.032780282 **Cellulose synthase 1** |
|  | -2.032054548 **Serinc-domain containing serine and sphingolipid biosynthesis** protein |
|  | -2.004121355 **Bifunctional inhibitor/lipid-transfer protein/seed storage 2S albumin** superfamily protein |
|  |  |
|  |  |

| **TRANSCRIPTION FACTORS** | |
| --- | --- |
| **INDUCED** | **REPRESSED** |
| 3.48394968 **NAC domain containing protein 36** | -3.118828065 **NAC domain containing protein 1** |
| 2.117500576 **NAC domain containing protein 41** | -2.154282533 **NAC domain containing protein 11** |
| 2.030754953 **NAC domain containing protein 100** |  |
|  |  |
| 2.009165774 **LEAFY COTYLEDON 2, AP2/B3-like transcriptional factor family protein**; embryo development, positive regulation of auxin biosynthetic process | -4.121362562 **AP2/B3-like transcriptional factor family protein** |
| 2.004428913 **RAP2.6**, encodes a member of the **ERF (ethylene response factor) subfamily B-4 of ERF/AP2 transcription factor** family**, ethylene-activated signaling pathway**, response to **abscisic acid, to cold, to jasmonic acid, to osmotic stress, to salicylic acid, to salt stress, to water deprivation, to wounding**. | -3.087547636 **AP2/B3-like transcriptional factor family protein**; NGATHA3, flower development, leaf development. |
|  | -2.603643556 **AP2 domain-containing transcription factor family** protein; **CYTOKININ RESPONSE FACTOR 12**, **ethylene-activated signaling pathway**, pollen development. |
|  |  |
| 3.194095461 **Nuclear factor Y, subunit C10** | -2.089412108 **Nuclear factor Y, subunit C6** |
| 2.311765907 **Nuclear factor Y, subunit B3** | -2.08500176 **Nuclear factor Y, subunit B8** |
| 2.250156268 **Nuclear factor Y, subunit C11** |  |
|  |  |
| 2.700876807 **WRKY DNA-binding protein 59** | -4.017484259 **WRKY DNA-binding protein 10** |
| 2.235852168 **WRKY DNA-binding protein 60** | -2.984373581 **WRKY DNA-binding protein 54** |
| 2.117084223 **WRKY DNA-binding protein 55** |  |
|  |  |
| 3.224708355 **Myb domain protein 117** | -2.652458819 **Myb domain protein 21** |
| 2.483093149 **Myb domain protein 58** | -2.291095903 **Myb domain protein 28** |
| 2.080588571 **Myb-like HTH transcriptional regulator family protein** | -2.188766389 **Myb domain protein 16** |
|  | -2.161907991 **Myb domain protein 27** |
|  | -2.129086264 **Myb domain protein 107** |
|  |  |
| 2.959733221 **Basic helix-loop-helix (bHLH) DNA-binding** superfamily protein | -3.816147048 **Basic helix-loop-helix (bHLH) DNA-binding** superfamily protein |
| 2.648895624 **Basic helix-loop-helix (bHLH) DNA-binding** superfamily protein | -3.361048327 **Basic helix-loop-helix (bHLH) DNA-binding** superfamily protein |
| 2.201403851 **Basic helix-loop-helix (bHLH) DNA-binding** superfamily protein | -2.48770086 **Basic helix-loop-helix 32** |
| 2.176465881 **Basic helix-loop-helix (bHLH) DNA-binding** superfamily protein | -2.433643788 **Basic helix-loop-helix (bHLH) DNA-binding** superfamily protein |
| 2.072647741Encodes a atypical member **of the bHLH (basic helix-loop-helix) family transcriptional factors**. | -2.587884861 **Basic helix-loop-helix (bHLH) DNA-binding** superfamily protein |
| 2.056236192 **Basic helix-loop-helix (bHLH) DNA-binding** superfamily protein | -2.567926614 **Winged helix-turn-helix transcription repressor** DNA-binding |
|  | -2.343926793 **Basic helix-loop-helix (bHLH) DNA-binding** superfamily protein |
|  | -2.148876622 **Basic helix-loop-helix (bHLH) DNA-binding** family protein |
|  |  |
| 3.160726259 **Homeodomain-like** superfamily protein | -3.164302659 **Homeodomain-like/winged-helix DNA-binding** family protein |
| 2.181971355 **Homeodomain-like** superfamily protein | -2.815897159 **Duplicated homeodomain-like** superfamily protein |
|  | -2.638504866 **Homeobox-leucine zipper protein** family |
|  | -2.195686574 **Homeodomain-like** superfamily protein |
|  | -2.113800632 **Homeodomain-like** superfamily protein |
|  | -2.032945818 **Homeodomain GLABROUS 12** |
|  | -2.009997625 **Homeobox-7** |
|  | -2.008421974 **Homeodomain-like** superfamily protein |
|  |  |
| 3.135163756 **C2H2-like zinc finger** protein | -2.361870455 **Zinc finger (C2H2 type)** family protein |
| 2.205802197 **C2H2 and C2HC zinc fingers** superfamily protein | -2.239616697 **C2H2-type zinc finger** family protein |
| 2.183946415 **C2H2-like zinc finger** protein |  |
| 2.074102587 **C2H2 and C2HC zinc fingers** superfamily protein |  |
|  |  |
| 2.712425572 **Basic leucine-zipper 58** | -2.402217404 **bZIP transcription factor** family protein |
|  | -2.129701579 **Basic leucine-zipper 52** |
|  |  |
|  | -2.520017214 **AGAMOUS-like 48** |
|  | -2.165249538 **AGAMOUS-like 13** |
|  | -2.107315262 **AGAMOUS-like 38** |
|  | -2.102525883 Encodes a subunit of the mediator complex that affects flowering time and floral organ formation through **FLOWERING LOCUS C (FLC) and AGAMOUS (AG).** |
|  | -2.045162997 **AGAMOUS-like 39** |
|  |  |
| 2.44092218 **Squamosa promoter binding protein-like 7** | -2.01038991 **Squamosa promoter binding protein-like 4** |
|  |  |
|  | -2.404266165 **SCARECROW-like 14** |
|  | -2.249872584 **GRAS family transcription factor** |
|  |  |
|  | -2.22634038 **MADS-box transcription factor** family protein |
|  | -2.210765381 **K-box region and MADS-box transcription factor** family protein |
|  |  |
|  | -2.10586903 **TEOSINTE BRANCHED 1**, cycloidea, and PCF family 24 |
|  | -2.037741492 **TCP family transcription factor** |
|  | -2.036618175 plant-specific transcription factor **YABBY family protein** |
|  |  |
|  | -2.000271129 **Dof-type zinc finger DNA-binding family protein;** **DOF AFFECTING GERMINATION 1**, response to red or far red light, seed germination. |
|  |  |
| 2.493279692 **Transcriptional factor B3** family protein | -3.211538786 **Heat shock transcription factor A4A** |
| 2.044651091 **Zinc ion binding; nucleic acid binding** | -2.553118294 **Transcriptional factor B3 family protein** |
|  | -2.724630855 **Transcription factor IIA, alpha/beta subunit** |
|  | -2.409348194 **Basal transcription factor complex subunit-related** |
|  |  |
|  |  |

| **PHYTOHORMONE-DEVELOPMENT-ABIOTIC STRESS** | |
| --- | --- |
| **INDUCED** | **REPRESSED** |
| **ABA-STOMATAL OPENING** | |
|  |  |
|  |  |
| **SALT/ DEHYDRATION-, STRESS-INDUCED PROTEINS** | |
| 3.197196349 **Low-temperature-responsive protein 78 (LTI78) / desiccation-responsive protein 29A (RD29A)** | -3.537704419 **Early-responsive to dehydration protein-related** / ERD protein-related |
| 2.228887222 CBL-interacting protein kinase 7; **SNF1-RELATED PROTEIN KINASE 3.10, response to cold** | -3.058742256 **Cold-regulated 47** |
| 2.089808625 **ERD (early-responsive to dehydration stress)** family protein | -2.489483057 **SNF1 kinase homolog 11** |
|  | -2.334828237 **Galactinol synthase 2** |
|  | -2.185418548 **Dehydrin Xero** 1 |
|  | -2.178988488 **ERD (early response to dehydration) six-like 1** |
|  | -2.171238811 **CBL-interacting protein kinase 19** |
|  | -2.146889324 **Dehydrin family protein** |
|  | -2.027449154 **Dehydrin family protein** |
|  |  |
|  |  |
| **AUXIN** | |
| 3.48894868 **SAUR-like auxin-responsive** protein family | -4.666166059 **Auxin efflux carrier** family protein |
| 2.687855156 **SAUR-like auxin-responsive** protein family | -3.104410066 **SAUR-like auxin-responsive** protein family |
| 2.027687141 **Auxin-responsive GH3** family protein | -2-2.936047419 **like AUXIN RESISTANT 2** |
|  | -2.918518969 **SAUR-like auxin-responsive** protein family |
|  | -2.888448187 **Late embryogenesis abundant (LEA) hydroxyproline-rich glycoprotein** family |
|  | -2.261004433 **Auxin efflux carrier** family protein |
|  | -2.139458788 **Auxin-responsive family** protein |
|  | -2.137247443 **Indole-3-acetic acid 6** |
|  | -2.105296156 **IAA-leucine-resistant (ILR1)-like 3** |
|  | -2.053005768 **Auxin-responsive family protein** |
|  | -2.019797923 **IAA-leucine resistant (ILR)-like gene 6** |
|  | -2.000284911 **Auxin-responsive GH3 family protein** |
|  |  |
|  |  |
| **BRASSINOSTEROIDS- GIBBERELLINS** | |
| 2.238174932 **PHYTOCHROME RAPIDLY REGULATED2 (PAR2), an atypical basic helix-loop-helix (bHLP) protein.** Up regulated after simulated **shade perception**. Acts in the nucleus to control plant development **and as a negative regulator of shade avoidance response. Brassinosteroid mediated signaling pathway, circadian rhythm.** | -3.055029204 **Gibberellin 3-oxidase 1** |
|  | -2.793073597 **BR enhanced expression 1** |
|  | -2.520441965 **BRI1 suppressor 1 (BSU1)-like 3** |
|  | -2.411438103 **Gibberellin 3-oxidase 2** |
|  |  |
|  |  |
| **CYTOKININS** | |
|  | -3.916135261 **Cytokinin response factor 2** |
|  | -3.244646146 **Purine permease 4** |
|  | -2.417752299 **Purine permease 6** |
|  |  |
|  |  |
| **ETHYLENE-JASMONIC ACID-WOUNDING** | |
| 2.743321963 **Ethylene-responsive nuclear protein -related** | -3.23012749 **Ethylene-dependent gravitropism-deficient and yellow-green-like 2** |
| 2.317562085 **Ethylene responsive element binding factor 2** | -2.537094 **Wound-responsive family protein** |
| 2.073041404 **Ethylene responsive element binding factor 5** |  |
|  |  |
|  |  |
| **GROWTH-DEVELOPMENT-CELL DIVISION** | |
| 2.503955224 **Lateral root primordium (LRP) protein-related** | -4.180564917 **Root hair specific 2** |
| 2.389048156 **Seed gene 3** | -3.324832434 **Cyclin b3;1** |
| 2.326831927 **Gamete-expressed 3** | -2.930972942 **CLAVATA3/ESR-RELATED 6** |
| 2.246302185 **Seed storage albumin 5** | -2.774105087 **Cell division control, Cdc6** |
| 2.145165528 **Embryo sac development arrest 7** | -2.650553422 **Rhodanese/Cell cycle control phosphatase** superfamily protein |
| 2.086306147 **Eukaryotic release factor 1-1; EUKARYOTIC; encodes a eukaryotic release factor 1 homolog.** Co-suppression of the gene's expression results affects **cell elongation of the inflorescence stem, specifically the internodes, and radial cell division.** Expression of the protein is primarily observed in the **vascular system and in actively growing and elongating zones.** | -2.605063607 **Integral membrane TerC** family protein**,** **PIGMENT DEFECTIVE 149 (PDE149); seedling development, thylakoid membrane organization.** |
| 2.007466992 **Somatic embryogenesis receptor-like kinase 1** | -2.394306382 **Lateral organ boundaries (LOB) domain family** protein |
|  | -2.388061505 **Somatic embryogenesis receptor-like kinase 5** |
|  | -2.21540437 **Cyclin A1;1** |
|  | -2.214614164 **Cyclin p4;3** |
|  | -2.159571553 **LOB domain-containing protein 23** |
|  | -2.151603841 **Embryonic flower 1 (EMF1)** |
|  | -2.122304161 **Kinesin 5** |
|  | -2.106137839 **root FNR 2** |
|  | -2.084126757 **Growth-regulating factor 5** |
|  | -2.081765625 **Hypoxia-responsive family protein / zinc finger (C3HC4-type RING finger)** family protein |
|  | -2.077430499 **LOB domain-containing protein 32** |
|  | -2.04341978 **LOB domain-containing protein 28** |
|  |  |

| **TRANSPORT** | |
| --- | --- |
| **INDUCED** | **REPRESSED** |
| 3.337520543 **COP1-interactive protein 1** | -3.292952923 **Coatomer gamma-2 subunit, putative / gamma-2 coat protein, putative / gamma-2 COP, putative** |
| 3.438727477 **Cation/H+ exchanger 16** | -3.144054581 **Mitochondrial substrate carrier** family protein |
| 3.210586276 **Heavy metal transport/detoxification** superfamily protein | -3.141783987 **Vesicle transport V-snare 13** |
| 2.900441224 **Rer1 family protein**; Encodes AtRER1C1, a Golgi membrane protein involved in returning the molecules that are exported from the endoplasmic reticulum (ER) to the Golgi apparatus back to the ER (a mechanism known as retrieval). | -3.135501477 **MATE efflux** family protein |
| 2.724915482 **Mitochondrial substrate carrier** family protein | -3.071152667 **Nodulin-like / Major Facilitator** Superfamily protein |
| 2.541788253 **Major facilitator superfamily** protein | -3.044389338 **High affinity nitrate transporter 2.7** |
| 2.541311437 **Nucleotide-sugar transporter** family protein | -2.988397412 Nodulin MtN21 /EamA-like transporter family protein |
| 2.474420914 **Major facilitator** superfamily protein |  |
| 2.3073185 0 **Rer1 family protein** | -2.985778979 Secretory 1A |
| 2.235156378 **Nodulin MtN21 /EamA-like transporter** family protein | -2.945622747 **Transmembrane amino acid transporter** family protein 1 |
| 2.207334575 **Plasma membrane intrinsic protein 1;5** | -2.938088244 **Rer1** family protein |
| 2.179825186 **Major facilitator** superfamily protein | -2.86520588 **ADP/ATP carrier 3** |
| 2.174471616 **COP1-interacting protein-related** | -2.84412557 **Lysine histidine transporter 1** |
| 2.106039641 **Coatomer, beta' subunit** | -2.810997571 **Polyketide cyclase/dehydrase and lipid transport** superfamily protein |
| 2.094768645 **Amino acid permease 6** | -2.808993185 **Polyketide cyclase/dehydrase and lipid transport** superfamily protein |
| 2.083672598 **Polyketide cyclase/dehydrase and lipid transport** superfamily protein |  |
| 2.055310065 **Translocon at the outer envelope membrane of chloroplasts 75-III** | -**2.804740943 Nuclear pore complex protein-related** |
| 2.028376774 **Signal recognition particle-related / SRP-related** | -2.80394131 **Mitochondrial substrate carrier** family protein |
| 2.040548027 **Ca^2+^ activated outward rectifying K+ channel 3** | -2.777361623 **Phosphate transporter 4;3** |
|  | -2.766134049 **Mitochondrial import inner membrane**  **translocase subunit Tim17/Tim22/Tim23** family protein |
|  | -2.705437909 **Aquaporin-like** superfamily protein |
|  | -2.684517066 **Nodulin MtN3** family protein |
|  | -2.655829928 **Major facilitator** superfamily protein |
|  | -2.65375398 **Polyketide cyclase/dehydrase and lipid transport** superfamily protein |
|  | -2.638321971 **Solute: sodium symporters; urea transmembrane** transporters |
|  | -2.624319449 **Divalent ion symporter** |
|  | -2.583636083 **ZIP metal ion transporter** family |
|  | -2.569780014 **Phosphate transporter 1;9** |
|  | -2.56504781 **Clathrin adaptor complexes medium subunit** family protein |
|  | -2.564143435 **CRT (chloroquine-resistance transporter)-like transporter 2** |
|  | -2.56116973 **Cyclic nucleotide-gated channel 17** |
|  | -2.556795288 **MATE efflux** family protein |
|  | -2.550092527 **EamA-like transporter** family |
|  | -2.494512846 **Lipid transporters** |
|  | -2.470516731 **Acyl carrier protein 2** |
|  | -2.467352753 **Multidrug resistance-associated protein 5** |
|  | -2.433212078 **Inositol transporter 3** |
|  | -2.419812424 **Heavy metal transport/detoxification** superfamily protein |
|  | -2.414242565 **MATE efflux** family protein |
|  | -2.397298 **Aluminium activated malate transporter** family protein |
|  | -2.376838221 **ATP-binding cassette 14** |
|  | -2.345157814 **Target SNARE coiled-coil domain protein** |
|  | -2.332180532 **Remorin** family protein |
|  | -2.325292454 **Copper-exporting ATPase / responsive-to-antagonist 1 / copper-transporting ATPase (RAN1)** |
|  | -2.324857427 **Gamma-adaptin 1** |
|  | -2.323959388 **Amino acid permease** family protein |
|  | -2.306316767 **Vacuolar glucose transporter 1** |
|  | -2.262459752 **Nodulin MtN3** family protein |
|  | -2.258764847 **Multidrug resistance-associated protein 7** |
|  | -2.225107703 **Got1/Sft2-like vescicle transport protein** family |
|  | -2.223484584 **Vacuolar protein sorting 26B** |
|  | -2.220479014 **Vacuolar protein sorting-associated protein 28 homolog 1** |
|  | -2.216343072 **Mitochondrial import inner membrane translocase subunit Tim17/Tim22/Tim23** family protein |
|  | -2.211883267 **Lipid transfer protein 1** |
|  | -2.211817609 **Cyclic nucleotide-gated channel 13** |
|  | -2.21023624 **Secretory carrier membrane protein (SCAMP)** family protein |
|  | -2.201399194 **Ureide permease 5** |
|  | -2.200571582 **Glucose 6-phosphate/phosphate translocator 1** |
|  | -2.198523372 **Nodulin MtN21 /EamA-like transporter** family protein |
|  | -2.184847639 **Zinc transporter 5 precursor** |
|  | -2.180009808 **Ca^2+^ activated outward rectifying K+ channel 5** |
|  | -2.169414472 **Plasma membrane intrinsic protein 1C** |
|  | -2.15985926 **Xanthine/uracil permease** family protein |
|  | -2.138871507 **Magnesium transporter 2** |
|  | -2.132881924 **Glutamine dumper 6** |
|  | -2.130287539 **Polyketide cyclase/dehydrase and lipid transport** superfamily protein |
|  | -2.122721863 **Ureide permease 2** |
|  | -2.111379753 **Nodulin MtN21 /EamA-like transporter** family protein |
|  | -2.102317983 **Bacterial sec-independent translocation protein mttA/Hcf106** |
|  | -2.07880778 **Nodulin MtN21 /EamA-like transporter** family protein |
|  | -2.074091009 **SecY protein transport** family protein |
|  | -2.066240099 **Major facilitator** superfamily protein |
|  | -2.065229268 **Cation exchanger 5** |
|  | -2.062633027 non-intrinsic **ABC protein 11** |
|  | -2.055908777 **Copper transporter 1** |
|  | -2.054536524 **S-adenosylmethionine carrier 2** |
|  | -2.051809692 **Major facilitator** superfamily protein |
|  | -2.04680807 **Nucleotide-sugar transporter** family protein |
|  | -2.046747547 **Cadmium tolerance 1** |
|  | -2.036266952 **Sodium hydrogen exchanger 4** |
|  | -2.036063407 **UDP-galactose transporter 3** |
|  | -2.033629504 **Phosphate transporter 4;1** |
|  | -2.026981793 **Aluminium activated malate transporter** family protein |
|  | -2.023931168 **MATE efflux** family protein |
|  | -2.014167423 **Drug/metabolite transporter** superfamily protein |
|  | -2.000009534 **Major facilitator** superfamily protein |
|  |  |
|  |  |

| **PROTEIN TRANSLATION, MODIFICATION, PROTEOLYSIS-PROTEIN DEGRADATION and others** | |
| --- | --- |
| **INDUCED** | **REPRESSED** |
| 3.570075493 **RING/U-box** superfamily protein | -5.634387782 **RING-H2 finger A1A** |
| 3.522032962 **RING/U-box** superfamily protein | -4.671172687 **Ubiquitin-conjugating enzyme 25** |
| 3.439380261 **50S ribosomal protein**-related |  |
| 3.428803329 **Ubiquitin domain-containing protein** | -4.114416993 **Calpain-type cysteine protease** family |
| 2.**945755543 F-box associated ubiquitination effector** family protein | -3.655028004 **RING/U-box** superfamily protein |
| 2.670355108 **Ubiquitin-like** superfamily protein | -3.655028004 **RING/U-box** superfamily protein |
| 2.651011732 **Ubiquitin-specific protease 15** | -3.541492431 **Peptidase C65, otubain** |
| 2.619729395 **Mannosyltransferase family** protein | -3.519740097 **Ubiquitin-like** superfamily protein |
| 2.417058286 **Serine carboxypeptidase-like 9** | -3.487488615 **RING/U-box** superfamily protein |
| 2.370731192 **RING/U-box** superfamily protein | -3.46261661 60S **Acidic ribosomal protein** family |
| 2.329201582 **SWAP (Suppressor-of-White-APricot)/surp domain-containing protein / ubiquitin family** protein | -3.366929414 **RING-H2 finger A1B** |
| 2.458851637 **Galactosyl transferase GMA12/MNN10** family protein | -3.352187802 **Ubiquitin-like** superfamily protein |
| 2.410983637 **Ribosome associated membrane protein RAMP4** | -3.343919799 **Thioesterase s**uperfamily protein |
| 2.383538773 **tRNA synthetase class I** (I, L, M and V) family protein | -3.342391402 **Ribosomal protein L13e family protein** |
| 2.220414733 **RING/U-box** superfamily protein | -3.319070949 **Ribosomal protein L18ae** family |
| 2.215114631 **Methionine aminopeptidase 2A** | -3.268391682 **Eukaryotic translation initiation factor 4G** |
| 2.201576704 **Ubiquitin-like** superfamily protein | -3.199544237 **Aminoacyl-tRNA ligases; nucleotide binding;ATP binding** |
| 2.186963228 **Peptide-N4-(N-acetyl-beta-glucosaminyl) asparagine amidase A protein** | -3.177367314 **Ribosomal protein L6** family protein |
| 2.171422719 **Proteophosphoglycan-related** | -3.125661053 **60S acidic ribosomal protein** family |
| 2.169005548 **Ubiquitin-like** superfamily protein | -3.098913847 **Ubiquitin 4** |
| 2.155202292 **26S proteasome regulatory complex**, non-ATPase subcomplex, **Rpn2/Psmd1 subunit** | -3.02049818 transposable_element_gene **similar to Ulp1 protease family protein** |
| 2.151045628 similar to **Ulp1 protease family protein** | -2.958266959 **RING/U-box** superfamily protein |
| 2.117111146 **Peptidase S24/S26A/S26B/S26C** family protein | -2.949800217 **RHOMBOID-like 1** |
| 2.113452629 **Subtilisin-like serine endopeptidase** family protein | -2.858075337 **Ubiquitin-conjugating enzyme 6** |
| 2.110539685 **Ubiquitin-specific protease family C19-related protein** | -2.836166526 **RING/U-box** superfamily protein |
| 2.10871544 **F-box associated ubiquitination** effector family protein | -2.800861837 **20S proteasome beta subunit G1** |
| 2.105912747 **RING/U-box** superfamily protein | -2.770657793 **Translation initiation factor 3 subunit H1** |
| 2.103983296 **60S acidic ribosomal** protein family | -2.689861245 **20S proteasome alpha subunit F2** |
| 2.093271454 **F-box associated ubiquitination** effector family protein | -2.643919903 **Ubiquitin-specific protease family C19-related** protein |
| 2.086036956 **RING/U-box** superfamily protein | -2.600196382 **N-terminal nucleophile aminohydrolases (Ntn hydrolases)** superfamily protein |
| 2.07035649 **RING/U-box** superfamily protein | -2.580775372 **RING/U-box** superfamily protein |
| 2.067628618 **FTSH protease 9** | -2.551586256 **Ubiquitin-like** superfamily protein |
| 2.065798707 **Mitochondrial ribosomal protein L11** | -2.533867647 **Ubiquitin-conjugating enzyme 35** |
| 2.041609233 **PPPDE putative thiol peptidase** family protein | -2.533502302 **N-terminal nucleophile aminohydrolases (Ntn hydrolases)** superfamily protein |
|  | -2.522993069 **RING/U-box** superfamily protein |
|  | -2.480475641 **RING/U-box** superfamily protein |
|  | -2.480063597 **Translation initiation factor 3 (IF-3) family** protein |
|  | -2.455369188 **Ribosomal protein L35Ae** family protein |
|  | -2.453884428 **Serine carboxypeptidase-like 15** |
|  | -2.452667665 **Ribosomal S17** family protein |
|  | -2.440046865 **Cysteine proteinases** superfamily protein |
|  | -2.423807738 **RING/U-box** superfamily protein |
|  | -2.419731835 **Peptidyl-tRNA hydrolase II (PTH2)** family protein |
|  | -2.371811197 **F-box associated ubiquitination effector** family protein |
|  | -2.365721323 **Glycoprotein membrane precursor GPI-anchored** |
|  | -2.328805773 **Serine carboxypeptidase-like 29** |
|  | -2.311419321 **Proteasome Inhibitor PI31** |
|  | -2.305569577 **tRNA synthetase class II** (G, H, P and S) family protein |
|  | -2.295133641 **RING/U-box** superfamily protein |
|  | -2.294032842 **Translation elongation factor EFG/EF2 protein** |
|  | -2.267465349 F**-box associated ubiquitination effector** family protein |
|  | -2.257718332 **Eukaryotic aspartyl protease** family protein |
|  | -2.218061762 **RING/U-box** superfamily protein |
|  | -2.194066565 **Ribosomal protein L7Ae/L30e/S12e/Gadd45** family protein |
|  | -2.190890601 **Serine protease inhibitor, potato inhibitor I-type** family protein |
|  | -2.188118455 **RHOMBOID-like protein 4** |
|  | -2.174754335 **Eukaryotic aspartyl protease** family protein |
|  | -2.16488597 **RING/U-box** superfamily protein |
|  | -2.162438917 **Serine protease inhibitor (SERPIN)** family protein |
|  | -2.146754778 **Cysteine proteinases** superfamily protein |
|  | -2.145489914 **Subtilase** family protein |
|  | -2.140958978 **Ribosomal protein S15A** |
|  | -2.115594136 **L-Aspartase-like** family protein |
|  | -2.103342923 **F-box associated ubiquitination effector** family protein |
|  | -2.092442961 **Ribosomal protein L12/ ATP-dependent Clp protease adaptor protein ClpS family protein** |
|  | -2.082786715 **60S acidic ribosomal** protein family |
|  | -2.071692496 **Papain family cysteine protease** |
|  | -2.052310812 **UDP-Glycosyltransferase** superfamily protein |
|  | -2.047884743 **RING/U-box** superfamily protein |
|  | -2.032447338 **Ribosomal protein L10** family protein |
|  | -2.030122253 **Ribosomal protein S10p/S20e** family protein |
|  | -2.009145648 **Ubiquitin carboxyl-terminal hydrolase** family protein |
|  |  |
|  |  |
| **SECONDARY METABOLISM** | |
| **INDUCED** | **REPRESSED** |
| 2.81795717 **Terpenoid synthases** superfamily protein | -3.563705985 **GDSL-like Lipase/Acylhydrolase** superfamily protein |
| 2.560175424 S**-adenosyl-L-methionine-dependent methyltransferases** superfamily protein | -3.438901482 **S-adenosyl-L-methionine-dependent methyltransferases** superfamily protein |
| 2.511572013 **Alpha/beta-Hydrolases** superfamily protein | -3.253079845 **HXXXD-type acyl-transferase** family protein |
| 2.469224433 **Acyl-CoA N-acyltransferase with RING/FYVE/PHD-type zinc finger domain** | -3.178657056 **OPC-8:0 CoA ligase1** |
| 2.457968456 **Fatty acid desaturase 2** | -3.141200776 **S-adenosyl-L-methionine-dependent methyltransferases** superfamily protein |
| 2.443974301 1**-deoxy-D-xylulose 5-phosphate synthase 1** | -3.119690729 **Acyl-CoA N-acyltransferases (NAT)** superfamily protein |
| 2.28098289 **SGNH hydrolase-type esterase** superfamily protein | -2.774167687 S**-adenosyl-L-methionine-dependent methyltransferases** superfamily protein |
| 2.232715876 **HXXXD-type acyl-transferase** family protein | -2.749424809 **S-adenosyl-L-methionine-dependent methyltransferases** superfamily protein |
| 2.228358144 **Adenine nucleotide alpha hydrolases-like** superfamily protein | -2.715424153 **Thiamin pyrophosphokinase 2** |
| 2.226510427 **GDSL-like Lipase/Acylhydrolase** superfamily protein | -2.715221332 **Pseudouridine synthase** family protein |
| 2.26216713 **Acyl-CoA N-acyltransferases (NAT)** superfamily protein | -2.701317262 **Putative methyltransferase** family protein |
| 2.225202251 **Alpha/beta-Hydrolases** superfamily protein | -2.685992253 **Alpha/beta-Hydrolases** superfamily protein |
| 2.195066419 **Alpha/beta-Hydrolases** superfamily protein | -2.68459629 **Acyl-activating enzyme 18** |
| 2.154612981 **Hydroxysteroid dehydrogenase 1** | -2.641776181 S-adenosyl-L-methionine-dependent methyltransferases superfamily protein |
| 2.142328195 **Prenylyltransferase** superfamily protein | -2.639443766 **Alpha/beta-Hydrolases** superfamily protein |
| 2.115580088 **NagB/RpiA/CoA transferase-like** superfamily protein | -2.623175342 **Hydrolase-like** protein family |
| 2.104473487 **S-adenosyl-L-methionine-dependent methyltransferases** superfamily protein | -2.581446216 **Geranylgeranyl pyrophosphate synthase 6** |
| 2.040905985 **Squalene synthase 1** | -2.566642949 **GDSL-like Lipase/Acylhydrolase** superfamily protein |
| 2.03028358 **Terpenoid cyclases/Protein prenyltransferases** superfamily protein | -2.559956481 **GDSL-like Lipase/Acylhydrolase** superfamily protein |
| 2.013630566 **Camelliol C synthase 1** | -2.554195831 **Isoprenylcysteine carboxyl methyltransferase** (ICMT) family |
|  | -2.508803155 **Alpha/beta-Hydrolases** superfamily protein |
|  | -2.47970178 **Nucleotidylyl transferase** superfamily protein |
|  | -2.425682494 **Terpenoid cyclases/Protein prenyltransferases** superfamily protein |
|  | -2.396861174 **Phospholipid/glycerol acyltransferase** family protein |
|  | -2.388336131 putative **Pentacyclic triterpene synthase 3** |
|  | -2.369162709 **Alpha/beta-Hydrolases** superfamily protein |
|  | -2.364210258 **Ureidoglycolate amidohydrolase** |
|  | -2.337739262 **Oleosin 2** |
|  | -2.335092873 **ACT domain-containing small subunit of acetolactate synthase** protein |
|  | -2.296359983 **Putative methyltransferase** family protein |
|  | -2.291170268 **3-oxo-5-alpha-steroid 4-dehydrogenase** family protein |
|  | -2.246447891 **Class-II DAHP synthetase** family protein |
|  | -2.228311191 **5-formyltetrahydrofolate cycloligase** |
|  | -2.188590084 **Methyltransferases** |
|  | -2.188517477 **Shikimate kinase 1** |
|  | -2.180658748 **GDSL-like Lipase/Acylhydrolase** superfamily protein |
|  | -2.173607273 **Mevalonate kinase** |
|  | -2.143507312 **Diphthamide synthesis DPH2** family protein |
|  | -2.135539212 **S-adenosyl-L-methionine-dependent methyltransferases** superfamily protein |
|  | -2.129102463 **Phosphoribosylaminoimidazole carboxylase, putative / AIR carboxylase,** putative |
|  | -2.1285538 **Acyl-CoA N-acyltransferase with RING/FYVE/PHD-type** zinc finger protein |
|  | -2.139452176 **Amino acid dehydrogenase** family protein |
|  | -2.110911934 **Acyl-activating enzyme 14** |
|  | -2.107824441 **Pyridoxine biosynthesis 1.2** |
|  | -2.103620681 **S-adenosyl-L-methionine-dependent methyltransferases** superfamily protein |
|  | -2.096612063 **Pyridoxal phosphate (PLP)-dependent transferases s**uperfamily protein |
|  | -2.087126557 **Zinc ion binding; nucleic acid binding; hydrolases, acting on acid anhydrides, in phosphorus-containing anhydrides** |
|  | -2.051800586 **S-adenosyl-L-methionine-dependent methyltransferases** superfamily protein |
|  | -2.049189409 **Alpha/beta-Hydrolases** superfamily protein |
|  | -2.04916121 3**-oxo-5-alpha-steroid 4-dehydrogenase** family protein |
|  | -2.048740412 **3-deoxy-d-arabino-heptulosonate 7-phosphate** synthase |
|  | -2.04104778 **Dihydrolipoamide acetyltransferase**, long form protein |
|  | -2.040265388 **Acyl-CoA oxidase 1** |
|  | -2.038834456 **Putative methyltransferase** family protein |
|  | -2.030378982 **Leucine carboxyl methyltransferase** |
|  | -2.029252782 **GLN phosphoribosyl pyrophosphate amidotransferase 3** |
|  | -2.006648466 **O-acyltransferase (WSD1-like)** family protein |
|  |  |
|  |  |

| **REDOX** | |
| --- | --- |
| **INDUCED** | **REPRESSED** |
| 2.871340103 **NAD(P)-binding Rossmann-fold** superfamily protein | -4.57871935 ***Arabidopsis thaliana* PEROXYGENASE 2** |
| 2.400582297 **NAD(P)-binding Rossmann-fold** superfamily protein | -4.397141823 **NAD(P)-binding Rossmann-fold** superfamily protein |
| 2.302812573 2**-oxoglutarate (2OG) and Fe(II)-dependent oxygenase** superfamily protein | -4.339771429 **2-oxoglutarate (2OG) and Fe(II)-dependent oxygenase** superfamily protein |
| 2.238316675 **Acyl-CoA oxidases;oxidoreductases, acting on the CH-CH group of donors**; FAD binding;oxidoreductases; **acyl-CoA oxidases** | -3.937106147 **Plant EC metallothionein-like protein, family 15** |
| 2.139479964 **2-oxoglutarate (2OG) and Fe(II)-dependent oxygenase** superfamily protein | -3.240450402 **FAD/NAD(P)-binding oxidoreductase family protein** |
| 2.115794817 **Thioredoxin superfamily** protein | -3.178049637 **Cytochrome P450, family 705, subfamily A, polypeptide 19** |
| 2.090316335 **Thioredoxin superfamily** protein | -3.080034056 **NAD(P)-binding Rossmann-fold** superfamily protein |
| 2.065538086 **Cytochrome P450, family 702, subfamily A, polypeptide 1** | -2.937706725 **Cytochrome P450, family 94, subfamily B, polypeptide 1** |
|  | -2.905873139 **Germin-like protein 8** |
|  | -2.812539771 **FAD-linked oxidases** family protein |
|  | -2.779040713 **2-oxoglutarate (2OG) and Fe(II)-dependent oxygenase** superfamily protein |
|  | -2.77164704 **2-oxoglutarate (2OG) and Fe(II)-dependent oxygenase** superfamily protein |
|  | -2.642022936 **Glutathione S-transferase zeta 1** |
|  | -2.610753785 **2-oxoglutarate (2OG) and Fe(II)-dependent oxygenase** superfamily protein |
|  | -2.601577331 **Thioredoxin superfamily** protein |
|  | -2.576780081 **Cytochrome p450 79f1** |
|  | -2.574244462 **2-oxoglutarate (2OG) and Fe(II)-dependent oxygenase** superfamily protein |
|  | -2.476695317 **Cytochrome P450, family 94, subfamily D, polypeptide 1** |
|  | -2.426914067 **Zinc-binding dehydrogenase** family protein |
|  | -2.408719359 **FAD/NAD(P)-binding oxidoreductase** family protein |
|  | -2.370870271 **2-oxoglutarate (2OG) and Fe(II)-dependent oxygenase** superfamily protein |
|  | -2.362415311 **Methionine sulfoxide reductase B 1** |
|  | -2.325440559 **Cupredoxin s**uperfamily protein |
|  | -2.323960138 **Peroxidase superfamily** protein |
|  | -2.305005082 **2-oxoglutarate (2OG) and Fe(II)-dependent oxygenase** superfamily protein |
|  | -2.312686898 **Flavin-dependent monooxygenase 1** |
|  | -2.198398966 **Metallothionein 2B** |
|  | -2.193114058 **Cytochrome P450, family 71, subfamily B, polypeptide 16** |
|  | -2.190179855 **NAD(P)-binding Rossmann-fold** superfamily protein |
|  | -2.174022507 **NAD(P)H dehydrogenase C1** |
|  | -2.168883862 **Cytochrome P450, family 71, subfamily A, polypeptide 15** |
|  | -2.167049321 **NAD(P)-linked oxidoreductase** superfamily protein |
|  | -2.134994488 **Cytochrome P450, family 705, subfamily A, polypeptide 13** |
|  | -2.130708174 **NAD(P)-binding Rossmann-fold** superfamily protein |
|  | -2.080186403 **Acid phosphatase/vanadium-dependent haloperoxidase-related protein** |
|  | -2.06590173 **Thioredoxin superfamily** protein |
|  | -2.064554945 **SKU5 similar 2**; oxidation-reduction process |
|  | -2.058112223 **NAD(P)-binding Rossmann-fold** superfamily protein |
|  | -2.037014497 **Peroxisomal NAD-malate dehydrogenase 2** |
|  | -2.036238794 **Cytochrome P450, family 77, subfamily A, polypeptide 4** |
|  |  |
|  |  |

| **CHAPERONES** | |
| --- | --- |
| **INDUCED** | **REPRESSED** |
| 2.451098796 **TCP-1/cpn60 chaperonin** family protein | -3.488531686 **Chaperone DnaJ-domain** superfamily protein |
| 2.115672726 **Cyclophilin-like peptidyl-prolyl cis-trans isomerase** family protein | -3.180938114 **HSP20-like chaperones** superfamily protein |
|  | -3.106322646 **Cyclophilin-like peptidyl-prolyl cis-trans isomerase** family protein |
|  | -2.860328995 **Chaperonin-60 alpha** |
|  | -2.73172347 **HSP20-like chaperone** |
|  | -2.690957745 **Chaperone DnaJ-domain** superfamily protein |
|  | -2.428324034 **TCP-1/cpn60 chaperonin** family protein |
|  | -2.344428067 **Chaperone protein htpG** family protein |
|  | -2.183868362 **Copper chaperone for SOD1** |
|  | -2.153084544 **HSP20-like chaperones** superfamily protein |
|  | -2.059802015 **TCP-1/cpn60 chaperonin** family protein |
|  | -2.066053155 **FKBP-like peptidyl-prolyl cis-trans isomerase** family protein |
|  | -2.032047719 **Chaperone DnaJ-domain** superfamily protein |
|  | -2.023966411 **HSP20-like chaperone** |
|  |  |
|  |  |

| **SIGNAL TRANSDUCTION** | |
| --- | --- |
| **INDUCED** | **REPRESSED** |
| 3.432191763 **HCP-like superfamily protein with MYND-type zinc finger** | -6.648797934 **F-box domain, Skp2-like, FBD-like, Leucine-rich repeat 2** |
| 3.390560873 **Kinase interacting (KIP1-like)** family protein | -4.048244992 **Calcium-binding EF-hand** family protein |
| 3.372357191 **Calcineurin-like metallo-phosphoesterase** superfamily protein | -3.964729045 **Type one serine/threonine protein phosphatase 4** |
| 2.343405088 **Alkaline-phosphatase-like** family protein | -3.648435114 FBD, F**-box and Leucine Rich Repeat domains containing protein** |
| 3.302261356 **Protein kinase superfamily** protein | -3.628726042 **Nucleotide-diphospho-sugar transferases** superfamily protein |
| 2.874912728 **Protein phosphatase 2A regulatory B subunit** family protein | -3.594069122 **Phosphatidic acid phosphatase (PAP2)** family protein |
| 2.852037626 **Vacuolar calcium-binding protein-related** | -3.519036077 **Nucleotide-diphospho-sugar transferases** superfamily protein |
| 2.70795215 **Calcium-dependent protein kinase 22** | -3.510946204 **WAG 1** |
| 2.631471123 **Protein kinase superfamily** protein | -3.472628799 **Protein kinase superfamily** protein |
| 2.500768472 **Protein phosphatase 2C** family protein | -3.265664663 **F-box family protein**-related |
| 2.420648236 **Lumazine-binding** family protein | -3.228892336 **F-box and associated interaction domains-containing protein** |
| 2.416113504 **Concanavalin A-like lectin** family protein | -3.195388115 **MAK10 homologue** |
| 2.4106184 **mirna MIR166/ MIR166D**; miRNA | -3.181393335 **AMP-dependent synthetase and ligase** family protein |
| 2.406156952 **Rho GTPase activation protein (RhoGAP) with PH domain** | -3.16552595 **Uridine kinase-like 3** |
| 2.349959565 **OSBP(oxysterol binding protein)-related protein 3C** | -3.087602378 **Nucleotide-diphospho-sugar transferases** superfamily protein |
| 2.3361895 0 **ROP-interactive CRIB motif-containing protein 7** |  |
| 2.324196842 **FAR1-related sequence 1;** response to red or far-red light. | -3.073615861 **Calcium-dependent lipid-binding (CaLB domain) family protein** |
| 2.291272892 **Calcium-dependent protein kinase 1** |  |
| 2.320991038 **Phospholipase D gamma 1** | -3.066971095 **MAP kinase 9** |
| 2.28811428 **Sec14p-like phosphatidylinositol transfer family protein** | -2.999856479 **Ran BP2/NZF zinc finger-like** superfamily protein |
| 2.259216818 **Diacylglycerol kinase 4** | -2.995196718 **Nucleotide-diphospho-sugar transferases** superfamily protein |
| 2.256056 **SNF2 domain-containing protein / helicase domain-containing protein / zinc finger protein-related** | -2.980241297 **Calcium-binding EF-hand** family protein |
| 2.247902204 **Ralf-like 33** | -2.932348036 **Protein kinase superfamily** protein |
| 2.212706231 **OSBP(oxysterol binding protein)-related** protein 2B | -2.909077861 **Mannose-binding lectin** superfamily protein |
| 2.169415742 **Cyclophilin** | -2.905609944 **MAP kinase 11** |
| 2.106471356 **Curculin-like (mannose-binding) lectin family** protein | -2.877717482 **Copine (Calcium-dependent phospholipid-binding protein)** family |
| 2.075517858 **RAN GTPase activating protein 1** | -2.853710056 **Lipase/lipooxygenase, PLAT/LH2** family protein |
| 2.066963803 **Protein phosphatase 2C** family protein | -2.78375265 **Sec14p-like phosphatidylinositol transfer** family protein |
| 2.06303275 **Mitogen activated protein kinase kinase kinase-related** | -2.783544524 **Lactoylglutathione lyase / glyoxalase I family protein** |
| 2.058882222 **Calcium-binding EF-hand** family protein | -2.77727578 **Mannose-binding lectin** superfamily protein |
| 2.0522824 **mirna MIR398C**; miRNA | -2.76874554 **Ralf-like 24** |
| 2.051890729 **Kinase interacting (KIP1-like) family protein** | -2.768071595 **Calcium-dependent protein kinase 6** |
| 2.048808697 **Inositol 1,3,4-trisphosphate 5/6-kinase** family protein | -2.748236505 **Calcium-binding EF hand** family protein |
| 2.051083917 **FBD, F-box, Skp2-like and Leucine Rich Repeat** domains containing protein | -2.722628021 **MAP kinase 19** |
| 2.049272812 **Nucleic acid-binding, OB-fold-like** protein | -2.711234221 **G-protein-coupled receptor 1** |
| 2.03133052 **3-phosphoinositide-dependent protein kinase** | -2.690338079 **CTC-interacting domain 4** |
| 2.022448129 **Protein kinase superfamily** protein | -2.654249669 **Sec14p-like phosphatidylinositol transfer** family protein |
| 2.01941523 **5'-AMP-activated protein kinase beta-2 subunit protein** | -2.642858603 **Fatty acid desaturase family protein** |
| 2.013197621 **Phosphotyrosine protein phosphatases** superfamily protein | -2.61502818 **MAP kinase kinase 7** |
| 2.012752542 **Protein kinase superfamily** protein | -2.593040292 **TRAM, LAG1 and CLN8 (TLC) lipid-sensing domain containing protein** |
| 2.009919666 **Mitogen-activated protein kinase kinase kinase 15** | -2.578130735 **Protein kinase superfamily** protein |
|  | -2.576261322 **Protein kinase superfamily** protein |
|  | -2.551112487 **Protein kinase superfamily** protein |
|  | -2.512607578 **Calcineurin-like metallo-phosphoesterase** superfamily protein |
|  | -2.488215467 **Early nodulin-like protein 15** |
|  | -2.482000089 **Protein kinase superfamily** protein |
|  | -2.46661409 **Protein phosphatase 2C** family protein |
|  | -2.456081659 Arabidopsis **Inositol phosphorylceramide synthase 3** |
|  | -2.420911851 **Sphingoid base hydroxylase 2** |
|  | -2.40357938 **mirna MIR395B; miRNA** |
|  | -2.39458016 **Phototropic-responsive NPH3 family protein** |
|  | -2.380657847 **GTP-binding protein-related** |
|  | -2.37998604 **Phototropic-responsive NPH3 family protein** |
|  | -2.368866054 **Protein kinase superfamily** protein |
|  | -2.340364219 **Protein kinase superfamily** protein |
|  | -2.339525092 **Ralf-like 4** |
|  | -2.337466871 **Calcium-binding EF hand** family protein |
|  | -2.333923362 **Mitogen-activated protein kinase kinase kinase 3** |
|  | -2.321469032 **Cullin family protein** |
|  | -2.31986441 **Response regulator 3** |
|  | -2.315514361 **Calmodulin-binding protein** |
|  | -2.31203087 **Prenylated RAB acceptor 1.G1** |
|  | -2.289948407 **Protein kinase superfamily** protein |
|  | -2.275938148 **Nucleotide-diphospho-sugar transferase** family protein |
|  | -2.267933717 **Calcineurin-like metallo-phosphoesterase** superfamily protein |
|  | -2.26731419 **ATP binding; protein kinases; protein serine/threonine kinases** |
|  | -2.263088025 **RAB GTPase homolog A4A** |
|  | -2.247162886 **Protein kinase superfamily** protein |
|  | -2.245095844 **UDP-Glycosyltransferase** superfamily protein |
|  | -2.217662484 **C2 calcium/lipid-binding plant** protein |
|  | -2.202483836 **Protein kinase superfamily** protein |
|  | -2.198934287 **ADP-ribosylation factor-like A1D** |
|  | -2.175396921 **GHMP kinase** family protein |
|  | -2.170457564 **Purple acid phosphatase 8** |
|  | -2.170190466 **Protein phosphatase 2C** family protein |
|  | -2.138186059 **Lipid phosphate phosphatase 2** |
|  | -2.126884932 **S-locus lectin protein kinase** family protein |
|  | -2.126517688 **Protein kinase family** protein |
|  | -2.099940965 **Ras-related small GTP-binding family protein** |
|  | -2.087184497 **Nucleic acid binding**; zinc ion binding |
|  | -2.070420957 **mirna MIR395E**; miRNA |
|  | -2.068357693 **FK506- and rapamycin-binding protein 15 kD-2** |
|  | -2.063190425 **GPCR-type G protein 1** |
|  | -2.061527474 **MIR168/MIR168B**; miRNA |
|  | -2.060235976 **TRAM, LAG1 and CLN8 (TLC) lipid-sensing domain containing protein** |
|  | -2.054338904 **Purple acid phosphatase 18** |
|  | -2.029869262 **Inositol-pentakisphosphate 2-kinase family protein** |
|  | -2.027044323 **SKP1 interacting partner 3** |
|  | -2.007422629 **CTC-interacting domain 10** |
|  | -2.007303184 **Ralf-like 8** |
|  |  |
|  |  |

| **DUF PROTEINS** | |
| --- | --- |
| **INDUCED** | **REPRESSED** |
| 2.938357229 Protein of Unknown Function (DUF239) | -3.940970845 Protein of unknown function (DUF677) |
| 2.830742535 Protein of unknown function (DUF616) | -3.146101614 Protein of unknown function (DUF300) |
| 2.73584138 Domain of unknown function DUF1794 | -3.086762154 Protein of unknown function (DUF1666) |
| 2.715425175 Protein of unknown function (DUF761) | -3.056223135 Protein of unknown function (DUF1639) |
| 2.70467372 Plant protein of unknown function (DUF828) | -3.026006826 Protein of unknown function (DUF679) |
| 2.674411194 Protein of unknown function (DUF1313) | -3.02592249 Protein of unknown function (DUF1163) |
| 2.490593361 Domain of unknown function (DUF1726) | -2.995402778 Protein of Unknown Function (DUF239) |
| 2.364103936 Protein of unknown function, DUF538 | -2.968786038 Plant protein of unknown function (DUF828) |
| 2.311491887 Protein of unknown function (DUF3133) | -2.966727505 Protein of unknown function (DUF295) |
| 2.265751966 Protein of unknown function (DUF1639) | -2.902303055 Plant protein of unknown function (DUF639) |
| 2.263151576 Protein of unknown function (DUF1624) | -2.857313426 Protein of unknown function, DUF593 |
| 2.239068595 Plant protein of unknown function (DUF868) | -2.849172545 Protein of unknown function (DUF640) |
| 2.233929771 Protein of unknown function (DUF1685) | -2.79284488 Protein of unknown function (DUF3411) |
| 2.216364874 Protein of unknown function, DUF647 | -2.777004685 Protein of unknown function (DUF581) |
| 2.173973005 Plant protein of unknown function (DUF827) | -2.707480329 Protein of unknown function (DUF295) |
| 2.148916555 Protein of unknown function (DUF579) | -2.701163446 Protein of unknown function (DUF1184) |
| 2.077635789 Protein of unknown function (DUF1012) | -2.658027365 Protein of unknown function (DUF3527) |
| 2.064328407 Protein of unknown function (DUF1997) | -2.612938166 Protein of unknown function (DUF810) |
| 2.0454337 Protein of unknown function (DUF581) | -2.602146633 Protein of unknown function DUF688 |
| 2.023478377 Protein of unknown function (DUF803) | -2.600416625 Protein of unknown function (DUF1218) |
| 2.005436789 DOMAIN OF UNKNOWN FUNCTION 724 6 | -2.60014182 Protein of unknown function (DUF784) |
|  | -2.55449941 Domain of unknown function (DUF966) |
|  | -2.530023971 Protein of unknown function (DUF607) |
|  | -2.475610888 Protein of unknown function DUF482, Acyl-CoA N-acyltransferase |
|  | -2.463849504 Protein of unknown function (DUF1223) |
|  | -2.39817241 Protein of Unknown Function (DUF239) |
|  | -2.34882083 Protein of unknown function (DUF300) |
|  | -2.34243934 Protein of unknown function DUF455 |
|  | -2.325439683 Protein of unknown function (DUF2930) |
|  | -2.311740749 Protein of unknown function (DUF784) |
|  | -2.267755074 Plant protein 1589 of unknown function |
|  | -2.217452846 Protein of unknown function, DUF617 |
|  | -2.211418769 Plant protein of unknown function (DUF247) |
|  | -2.207336371 Protein of unknown function (DUF630 and DUF632) |
|  | -2.190168889 Protein of unknown function (DUF1645) |
|  | -2.190069393 Protein of unknown function (DUF1295) |
|  | -2.167606137 Family of unknown function (DUF577) |
|  | -2.157150842 Arabidopsis protein of unknown function (DUF241) |
|  | -2.114069401 Protein of unknown function (DUF761) |
|  | -2.103960892 Protein of unknown function (DUF295) |
|  | -2.097905606 Protein of unknown function (DUF295) |
|  | -2.096143924 Uncharacterized protein family (UPF0016) |
|  | -2.086773353 Protein of unknown function (DUF 3339) |
|  | -2.068982187 Protein of unknown function DUF985 |
|  | -2.012905997 Uncharacterised protein family (UPF0497) |
|  | -2.001793868 Protein of unknown function (DUF668) |
|  | -2.000356988 Protein of unknown function (DUF668) |
|  |  |
|  |  |

| **CHLOROPLAST-PHOTOSYNTHESIS-ENERGY-PHOTORESPIRATION** | |
| --- | --- |
| **INDUCED** | **REPRESSED** |
| 3.231266317 **Plastid-lipid associated protein PAP / fibrillin** family protein | -3.056494729 **ATPase, V0 complex, subunit E** |
| 3.015514824 **Pyrophosphate--fructose-6-phosphate 1-phosphotransferase-related** / pyrophosphate-dependent 6-phosphofructose-1-kinase-related | -3.046958955 **Gamma subunit of Mt ATP synthase** |
| 2.858555461 **Phosphofructokinase 1** | -2.989846789 **Phosphofructokinase** family protein |
| 2.792997087 **Regulatory particle triple-A ATPase 6A** | -2.9183434 **P-type ATPase of Arabidopsis 2** |
| 2.471106727 **Plastid transcriptionally active 6** | -2.83898905 **NADH-ubiquinone oxidoreductase 24 kDa subunit, putative** |
| 2.504119832 **ADP glucose pyrophosphorylase large subunit 1** | -2.770247389 **Sedoheptulose-bisphosphatase** |
| 2.189069175 **Photosystem II assembly, regulation of chlorophyll biosynthetic process, photosystem I assembly, thylakoid membrane organization**, RNA modification | -2.737482669 **Vacuolar proton ATPase A2** |
| 2.21775299 **Mitochondrial editing factor 22** | -2.702972266 **Ribulose bisphosphate carboxylase small chain 1A** |
| 2.072210241 **Alanine: glyoxylate aminotransferase** | -2.64008033 **2Fe-2S ferredoxin-like** superfamily protein |
| 2.00835034 **Photosystem II subunit R** | -2.622390159 **Coproporphyrinogen III oxidase** |
|  | -2.615178863 **Phosphoglucomutase/phosphomannomutase** family protein |
|  | -2.599716901 **Homolog of yeast autophagy 18 (ATG18) H** |
|  | -2.593170254 **Cytochrome c oxidase 19-1** |
|  | -2.565221025 **Pyruvate kinase** family protein |
|  | -2.535377401 **Vacuolar ATPase assembly integral membrane protein VMA21-like domain** |
|  | -2.511728413 **Phosphoribulokinase** |
|  | -2.502081701 **Aldolase-type TIM barrel** family protein |
|  | -2.458260284 **AUTOPHAGY 6** |
|  | -2.446866766 **Ferredoxin 1** |
|  | -2.390214504 **Plastid transcriptionally active 14** |
|  | -2.342902101 **Ribulose bisphosphate carboxylase (small chain) family protein** |
|  | -2.338481796 **Urophorphyrin methylase 1** |
|  | -2.249121346 **P-type ATPase 1** |
|  | -2.247591954 **Chlorophyll A-B binding family protein** |
|  | -2.246904037 **Sucrose-6F-phosphate phosphohydrolase** family protein |
|  | -2.234149361 **Glycine decarboxylase complex H** |
|  | -2.230552538 **Ubiquinol-cytochrome C reductase iron-sulfur subunit** |
|  | -2.224451545 **Ribosome recycling factor, chloroplast precursor** |
|  | -2.214452657 **Glucose-6-phosphate dehydrogenase 2** |
|  | -2.18183745 **ATP-citrate lyase A-1** |
|  | -2.174121436 **INVOLVED IN: photosynthesis** |
|  | -2.119566834 **ATPase, F0/V0 complex, subunit C protein** |
|  | -2.096414721 **Induced by phosphate starvation1** |
|  | -2.084260783 **P-type ATPase 1** |
|  | -2.055899861 **ATP phosphoribosyl transferase 2** |
|  | -2.053795016 **High chlorophyll fluorescence 153** |
|  | -2.052535534 **Beta-amylase 3** |
|  | -2.039984748 **Electron transport SCO1/SenC family protein** |
|  | -2.037092615 **Chlorophyll A/B binding protein 3** |
|  | -2.011538925 **Chlorophyll A/B-binding protein 2** |
|  |  |
|  |  |

| **DNA REPAIR-HISTONE MODIFICATIONS-RNA MODIFICATIONS** | |
| --- | --- |
| **INDUCED** | **REPRESSED** |
| 3.011925433 **Histone acetyltransferase of the TAFII250 family 2** | -4.569454616 **Decapping 5** |
| 3.**006229147 Transposable_element_gene similar to RNase H domain-containing protein** | -4.045881021 **P-loop containing nucleoside triphosphate hydrolases** superfamily protein |
| 2.875027084 **RNA helicase** family protein | -3.828403962 **GDA1/CD39 nucleoside phosphatase** family protein |
| 2.74674037 **DEAD/DEAH box RNA helicase** family protein | -3.442763688 **DNA directed RNA polymerase, 7 kDa subunit** |
| 2.732589977 **DNA methyltransferase 2** | -3.383179417 **P-loop containing nucleoside triphosphate hydrolases** superfamily protein |
| 2.651850411 **DNAse I-like** superfamily protein | -3.322216826 **Regulator of chromosome condensation (RCC1)** family protein |
| 2.650449248 **RNA-binding CRS1 / YhbY (CRM) domain** protein | -3.320163791 **Mitochondrial transcription termination factor** family protein |
| 2.578990445 **RNA splicing factor-related** | -3.2676532 **dsRNA-binding domain-like** superfamily protein |
| 2.577463152 **Nucleic acid-binding, OB-fold-like** protein | -3.249528825 similar to **RNase H domain-containing** protein |
| 2.549020528 **Regulator of chromosome condensation (RCC1)** family protein | -3.199791248 **RNA-DIRECTED DNA METHYLATION 1** |
| 2.547519862 **DNA LIGASE 6** | -3.149109606 **Arabidopsis retrotransposon ORF-1 protein** |
| 2.522797247 **DNA-directed RNA polymerase, subunit M, archaeal** | -3.**141653244 P-loop containing nucleoside triphosphate hydrolases** superfamily protein |
| 2.511251485 **DEAD box RNA helicase** family protein | -3.027193532 **RNA-binding KH domain-containing protein** |
| 2.504520862 **Polynucleotidyl transferase, ribonuclease H-like** superfamily protein | -3.023824693 **GDA1/CD39 nucleoside phosphatase** family protein |
| 2.4638092 0.0 **DNA-binding bromodomain-containing protein** | -2.827060627 **Ribosomal RNA adenine dimethylase family protein** |
|  | -2.819614066 **P-loop containing nucleoside triphosphate hydrolases** superfamily protein |
| 2.433411846 **mRNA splicing factor, Cwf18** | -2.771854875 **Integrase-type DNA-binding** superfamily protein |
| 2.352885029 **DNA primase, large subunit family** | -2.744350212 **RNA polymerase II transcription mediators** |
| 2.332005224 **Minichromosome maintenance (MCM2/3/5)** family protein | -2.702912984 **Ribonuclease II/R** family protein |
| 2.317204039 **RNA-binding (RRM/RBD/RNP motifs)** family protein | -2.640207782 **RNA-binding (RRM/RBD/RNP motifs)** family protein |
| 2.30032307 **rRNA processing protein-related** | -2.603095829 **RNA ligase/cyclic nucleotide phosphodiesterase** family protein |
| 2.285268159 **pre-mRNA-processing protein 40B** | -2.525546228 **DEA(D/H)-box RNA helicase** family protein |
| 2.262911863 **Exonuclease family protein** | -2.525038459 **A20/AN1-like zinc finger** family protein |
| 2.153713054 **Minichromosome maintenance (MCM2/3/5)** family protein | -2.507746726 **ATP-dependent peptidases**; **nucleotide binding**; serine-type endopeptidases; **DNA helicases**; **ATP binding;** **damaged DNA binding**; **nucleoside-triphosphatases** |
| 2.124182261 **RNA polymerase Rpb6** | -2.48097305 P**-loop containing nucleoside triphosphate hydrolases** superfamily protein |
| 2.06785599 **P-loop containing nucleoside triphosphate hydrolases** superfamily protein | -2.456435024 **ATP-dependent helicase** family protein |
| 2.062530966 **RNA-directed DNA polymerase (reverse transcriptase)**-related family protein | -2.423541731 **P-loop containing nucleoside triphosphate hydrolases** superfamily protein |
| 2.060072402 **Phosphorylated adapter RNA export protein, RNA-binding domain** | -2.361740435 **Minichromosome maintenance (MCM2/3/5)** family protein |
| 2.055812478 **DNA/RNA polymerases** superfamily protein | -2.350997952 **RNAse II-like 1** |
| 2.021408725 **RNA recognition motif (RRM)-containing protein** | -2.340202072 **RNase H family** protein |
| 2.003686164 **RNA-binding protein 45A** | -2.328375524 **RNA polymerase Rpb7 N-terminal domain-containing protein** |
|  | -2.328375375 **P-loop containing nucleoside triphosphate hydrolases** superfamily protein |
|  | -2.309508807 **P-loop containing nucleoside triphosphate hydrolases** superfamily protein |
|  | -2.304587964 **Integrase-type DNA-binding** superfamily protein |
|  | -2.298361266 **Ribonuclease H-like** superfamily protein |
|  | -2.298078629 **RNA-binding (RRM/RBD/RNP motifs)** family protein |
|  | -2.248782916 **RNA-binding (RRM/RBD/RNP motifs)** family protein |
|  | -2.246007586 **Polynucleotidyl transferase, ribonuclease H-like** superfamily protein |
|  | -2.238441936 **DNA ligase 1** |
|  | -2.234816612 **Telomere repeat binding factor 1** |
|  | -2.215551703 **Polynucleotide adenylyltransferase** family protein |
|  | -2.185613919 **mRNA splicing factor, thioredoxin-like U5 snRNP** |
|  | -2.168838533 **Male-gamete-specific histone H3** |
|  | -2.087191451 **Splicing factor, putative** |
|  | -2.084040477 **Small nuclear RNA activating complex (SNAPc), subunit SNAP43 protein** |
|  | -2.067047837 **RECQ helicase L4B** |
|  | -2.056969077 **RECQ helicase L2** |
|  | -2.052019117 **Poly(A) binding protein 1** |
|  | -2.040902878 **RNA-binding (RRM/RBD/RNP motifs)** family protein |
|  |  |
|  |  |

| **OTHER** | |
| --- | --- |
| **INDUCED** | **REPRESSED** |
| 3.491679267 **Beige/BEACH domain** ;WD domain, G-beta repeat protein | -5.621560661 **ARM repeat** superfamily protein |
| 3.055719745 **Homolog of yeast ADA2 2B** | -3.740871394 F**-box family** protein |
| 2.90437742 **Tetratricopeptide repeat (TPR)-like** superfamily protein | -3.666718506 **BTB/POZ domain-containing** protein |
| 2.860754152 **Pumilio 13** | -3.563926425 **Rubredoxin-like** superfamily protein |
| 2.84032925 **SGF29 tudor-like domain** | -3.505429252 **Reticulon** family protein |
| 2.795120235 **Galactose oxidase/kelch repeat** superfamily protein | -3.431203181 **Cryptdin protein**-related |
| 2.662943282 **Nudix hydrolase homolog 26** | -3.360455677 **ARM repeat** superfamily protein |
| 2.61435705 **RING/FYVE/PHD-type zinc finger** family protein | -3.315079974 **Pentatricopeptide repeat (PPR)** superfamily protein |
| 2.576815779 **Zinc knuckle (CCHC-type)** family protein | -3.310746004 **DHHC-type zinc finger** family protein |
| 2.566751132 **Ca^2+-^activated RelA/spot homolog** | -3.263165648 **Evolutionarily conserved C-terminal region 11** |
| 2.521910401 **Tubby like protein 6** | 3.039079977 **Tetratricopeptide repeat (TPR)-like** superfamily protein |
| 2.515050652 **Pentatricopeptide repeat (PPR-like)** superfamily protein | -3.03234728 **Tetratricopeptide repeat (TPR)-like** superfamily protein |
| 2.492443292 **YEATS family** protein | -3.015481652 **RmlC-like cupins** superfamily protein |
| 2.483875817 **Prohibitin 3** | -3.006518714 **RNI-like superfamily** protein |
| 2.468952687 **SLAC1 homologue 3** | -2.957170934 **SMAD/FHA domain-containing** protein |
| 2.442056203 **Tetratricopeptide repeat (TPR)-like** superfamily protein | -2.921340555 **RNI-like** superfamily protein |
| 2.423909637 **Small and basic intrinsic protein 2;1** | -2.909890151 **D-alanine--D-alanine ligase** family |
| 2.375622708 **Receptor homology region transmembrane domain ring H2 motif protein 1** | -2.904047034 **F-box/RNI-like** superfamily protein |
| 2.341725851 **Single hybrid motif** superfamily protein | -2.903393155 **RING/FYVE/PHD zinc finger** superfamily protein |
| 2.334417238 **Evolutionarily conserved C-terminal region 10** | -2.896374947 **Transducin/WD40 repeat-like** superfamily protein |
| 2.32921039 **Pseudouridine synthase** family protein | -2.862208466 **ARM repeat** superfamily protein |
| 2.326358554 **IQ-domain 29** | -2.845600912 **Zinc finger protein**-related |
| 2.302949026 **RING/FYVE/PHD zinc finger** superfamily protein | -2.840568072 **TRF-like 3** |
| 2.298486062 **Long chain base2** | -2.808659796 **HOPW1-1-interacting 1** |
| 2.236347927 **Ankyrin repeat-containing 2B** | -2.807728754 **Transducin/WD40 repeat-like** superfamily protein |
| 2.236292746 **TRAF-like family protein** | -2.800226991 **Pumilio 8** |
| 2.224559096 **MUTL protein homolog 3** | -2.785989826 **MMS ZWEI homologue 3** |
| 2.192406 **YELLOW STRIPE like 1** | -2.783422067 **Rubber elongation factor protein (REF)** |
| 2.16102819 **Switch subunit 3** | -2.749227198 **Pentatricopeptide repeat (PPR)** superfamily protein |
| 2.151770041 **TRAF-like superfamily protein** | -2.741101156 **PHD finger protein-related** |
| 2.147975023 **Ypt/Rab-GAP domain of gyp1p** superfamily protein | -2.734823825 **Tetratricopeptide repeat (TPR)-like** superfamily protein |
| 2.1478944 **VQ motif-containing** protein | -2.72987633 **CCCH-type zinc finger protein with ARM repeat** domain |
| 2.147278946 **Surfeit locus protein 2 (SURF2)** | -2.729242925 **Galactose oxidase/kelch repeat** superfamily protein |
| 2.104630812 **SKP1/ASK1-interacting protein 2** | -2.712516536 **Putative adipose-regulatory protein (Seipin)** |
| 2.099851658 **Carbon/nitrogen insensitive 1** | -2.700036024 **Protein with RNI-like/FBD-like domains** |
| 2.096589824 **SNF7 family protein** | -2.694265422 **ENTH/VHS family protein** |
| 2.091426404 **Tetratricopeptide repeat (TPR)-like** superfamily protein | -2.692362008 **BREVIS RADIX-like 1** |
| 2.078774185 **MLP-like protein 168** | -2.686051238 **PYR1-like 4** |
| 2.072909017 **LSD1-like2** | -2.678125724 **Breast cancer susceptibility1** |
| 2.06030528 **Sterile alpha motif (SAM) domain-containing** protein | -2.67214628 **Paired amphipathic helix repeat-containing protein** |
| 2.036584726 **DNA-binding bromodomain-containing protein** | -2.661464717 **IQ-domain 18** |
| 2.035841667 **HIS triad family protein 3** | -2.649116831 **RING/FYVE/PHD zinc finger** superfamily protein |
| 2.018685961 **SPX (SYG1/Pho81/XPR1) domain-containing**  **protein / zinc finger (C3HC4-type RING finger)** protein-related | -2.626490046 **Pentatricopeptide repeat (PPR)** superfamily protein |
| 2.01667888 **DNA glycosylase** superfamily protein | -2.62524035 **F-box family** protein |
|  | -2.609204062 **ELMO/CED-12 family protein** |
|  | -2.578847342 **Plant self-incompatibility protein S1** family |
|  | -2.573398753 **Integral membrane HRF1** family protein |
|  | -2.575564404 **Nucleic acid-binding, OB-fold-like** protein |
|  | -2.567730037 **Nucleic acid-binding, OB-fold-like** protein |
|  | -2.539093939 **BREVIS RADIX-like 3** |
|  | -2.550523215 **Sas10/Utp3/C1D** family |
|  | -2.532098493 **Tetratricopeptide repeat (TPR)-like** superfamily protein |
|  | -2.529577777 **Zinc knuckle (CCHC-type)** family protein |
|  | -2.515559205 **Tetratricopeptide repeat (TPR)-like** superfamily protein |
|  | -2.511638075 **EXS (ERD1/XPR1/SYG1**) family protein |
|  | -2.485157397 **Plus-3 domain-containing protein** |
|  | -2.481614354 **Zinc finger C-x8-C-x5-C-x3-H type** family protein |
|  | -2.480819301 **SIN3-like 4** |
|  | -2.461380383 **Breast basic conserved 1** |
|  | -2.448295479 **MMS ZWEI homologue 1** |
|  | -2.447536232 **Surfeit locus protein 6** |
|  | -2.44567116 **Reticulata-related 1** |
|  | -2.444588913 **ArfGap/RecO-like zinc finger domain-containing** protein |
|  | -2.436192342 **Basic pentacysteine 3** |
|  | -2.435313375 **Nucleic acid-binding, OB-fold-like** protein |
|  | -2.428672468 **Associated molecule with the SH3 domain of STAM 1** |
|  | -2.426408576 **Pentatricopeptide repeat (PPR)** superfamily protein |
|  | -2.414440192 **F-box/RNI-like** superfamily protein |
|  | -2.390057595 **Uclacyanin 3** |
|  | -2.388817329 **Armadillo repeat kinesin 2** |
|  | -2.40672355 **F-box family** protein |
|  | -2.365130908 **Pumilio 17** |
|  | -2.352730719 **PapD-like** superfamily protein |
|  | -2.349126155 **Outer membrane OMP85** family protein |
|  | -2.347495264 **Emp24/gp25L/p24 family/GOLD** family protein |
|  | -2.337941803 **Transducin family protein / WD-40 repeat** family protein |
|  | -2.32587744 **Phosphorylase** superfamily protein |
|  | -2.32142522 **SET domain-containing** protein |
|  | -2.30285756 **BTB/POZ domain**-containing protein |
|  | -2.295371477 **Tetratricopeptide repeat (TPR)-like** superfamily protein |
|  | -2.290429556 **Tetratricopeptide repeat (TPR)-like** superfamily protein |
|  | -2.289417 **B12D protein** |
|  | -2.287964034 **Tetratricopeptide repeat (TPR)-like** superfamily protein |
|  | -2.286697249 **Bacterial hemolysin-related** |
|  | -2.283349482 **Pumilio 7** |
|  | -2.26995161 **Sporulation 11-2** |
|  | -2.26474563 **F-box and associated** interaction domains-containing protein |
|  | -2.260350323 **Galactose oxidase/kelch repeat** superfamily protein |
|  | -2.245609744 **Camphor resistance CrcB family protein** |
|  | -2.234617204 **F-box/RNI-like** superfamily protein |
|  | -2.234166 **Galactose oxidase/kelch repeat** superfamily protein |
|  | -2.233630155 **Vps52 / Sac2 family** |
|  | -2.209472914 **CHY-type/CTCHY-type/RING-type Zinc finger** protein |
|  | -2.203905814 **B-cell receptor-associated 31-like** |
|  | -2.203281553 **TRAF-like** family protein |
|  | -2.200661639 **Zinc finger C-x8-C-x5-C-x3-H type** family protein |
|  | -2.199231514 **Transducin/WD40 repeat-like** superfamily protein |
|  | -2.194569897 **Bromodomain and extraterminal domain protein 9** |
|  | -2.193653913 **BSD domain**-containing protein |
|  | -2.189212359 **RGA-like 1** |
|  | -2.184571183 **Transducin/WD40 repeat-like** superfamily protein |
|  | -2.178782298 **Cox19-like CHCH family** protein |
|  | -2.175252278 **APR-like 5** |
|  | -2.171239087 **Pentatricopeptide repeat (PPR-like)** superfamily protein |
|  | -2.167792856 **Prohibitin 2** |
|  | -2.158337092 **ELMO/CED-12 family protein** |
|  | -2.155806455 **XH domain-containing** protein |
|  | -2.147757768 **Sterile alpha motif (SAM) domain**-containing protein |
|  | -2.143709869 **Pentatricopeptide repeat (PPR)** superfamily protein |
|  | -2.13725494 **Tetratricopeptide repeat (TPR)-like** superfamily protein |
|  | -2.130875297 **Pentatricopeptide repeat (PPR)** superfamily protein |
|  | -2.130128833 **RING/FYVE/PHD zinc finger** superfamily protein |
|  | -2.121782005 **Arv1-like** protein |
|  | -2.120536151 **SET domain-containing** protein |
|  | -2.118202189 **DNA glycosylase** superfamily protein |
|  | -2.161227958 **RNI-like** superfamily protein |
|  | -2.117972747 **GCR2-like 2** |
|  | -2.115007431 **Transducin/WD40 repeat-like** superfamily protein |
|  | -2.105440919 **Survival protein SurE-like phosphatase/ nucleotidase** |
|  | -2.101239161 **DNA glycosylase** superfamily protein |
|  | -2.093149611 **Zinc knuckle (CCHC-type)** family protein |
|  | -2.090611491 **Pumilio 25** |
|  | -2.088500295 **Mov34/MPN/PAD-1 family protein** |
|  | -2.083042958 **AWPM-19-like** family protein |
|  | -2.081616035 **SET domain protein 38** |
|  | -2.078590774 **TRAF-like** family protein |
|  | -2.075663464 **Homolog of yeast ergosterol 28** |
|  | -2.071921157 **AAR2 protein family** |
|  | -2.056085918 **SWAP (Suppressor-of-White-APricot)/surp RNA-binding domain-containing** protein |
|  | -2.053929752 **RWD domain-containing** protein |
|  | -2.049992539 **Zinc finger protein 11** |
|  | -2.035958855 **Tubby-like protein 9** |
|  | -2.031215866 **Galactose oxidase/kelch repeat** superfamily protein |
|  | -2.02380979 **Tetratricopeptide repeat (TPR)-like** superfamily protein |
|  | -2.023032495 **Tetratricopeptide repeat (TPR)-like** superfamily protein |
|  | -2.02218367 **Reticulon** family protein |
|  | -2.01993517 **RNI-like** superfamily protein |
|  | -2.015950679 **Arginine/serine-rich protein**-related |
|  | -2.012039771 **VQ motif-containing** protein |
|  | -2.010351238 **F-box and associated interaction domains**-containing protein |
|  | -2.001212952 **Pseudo-response regulator 6** |
|  |  |
|  |  |
