# Supplementary material for: Overexpression of Grain Amaranth (Amaranthus hypochondriacus) AhERF or AhDOF Transcription Factors in Arabidopsis thaliana Increases Water Deficit- and Salt-Stress Tolerance, Respectively, via Contrasting Stress-Amelioration Mechanisms
Source: PLoS One. 2016 Oct 17;11(10):e0164280. doi: 10.1371/journal.pone.0164280 (PMC5066980; doi:10.1371/journal.pone.0164280)
Supplement: S4 Table — (DOCX) [file pone.0164280.s010.docx]

**S4 Table**. **List of representative genes with altered expression detected in transgenic *AhERF-VII* overexpressing Arabidopsis plants under water-deficit stress.**

| **DISEASE RESISTANCE** | |
| --- | --- |
| **INDUCED** | **REPRESSED** |
| **Disease resistance protein (TIR-NBS-LRR class**) family | **Disease resistance protein (TIR-NBS-LRR class),** putative |
| similar to **Disease resistance protein (TIR-NBS-LRR class)** | **Disease resistance protein (TIR-NBS-LRR class)** family |
| **Disease resistance-responsive** (**dirigent-like protein**) family protein | **Disease resistance protein (TIR-NBS-LRR class)**, putative |
| **Toll-Interleukin-Resistance (TIR) domain** family protein | **Disease resistance protein (CC-NBS-LRR class)** family |
|  | **Disease resistance protein (TIR-NBS-LRR class**) family |
| **Cysteine-rich RLK (RECEPTOR-like protein kinase) 11**; AT-RLK3, CRK11, CYSTEINE-RICH RLK (RECEPTOR-LIKE PROTEIN KINASE) 11, RECEPTOR LIKE PROTEIN KINASE 3; Encodes putative receptor-like protein kinase that is **induced by the soil-borne vascular bacteria, Ralstonia solanacearum.** | **Disease resistance protein (TIR-NBS-LRR class)** family |
| Receptor like protein 51; ATRLP51, **RECEPTOR LIKE PROTEIN 51**, RLP51, SNC2, SUPPRESSOR OF NPR1-1, CONSTITUTIVE 2 | **CRK27, CYSTEINE-RICH RLK (RECEPTOR-LIKE PROTEIN KINASE)** 27; **defense response**, protein phosphorylation |
|  |  |
|  | **Receptor-like protein kinase-related** family protein |
|  | **Receptor protein kinase**-related |
|  |  |
|  |  |
| **CAP (Cysteine-rich secretory proteins, Antigen 5**, and **Pathogenesis-related 1 protein**) superfamily protein | **CAP (Cysteine-rich secretory proteins, Antigen 5, and Pathogenesis-related 1 protein**) superfamily protein |
| **Pathogenesis-related thaumatin** superfamily protein | **Defensin-related** |
|  | LCR80**, LOW-MOLECULAR-WEIGHT CYSTEINE-RICH 80**  Encodes a member of a family of small, secreted, cysteine rich protein with sequence similarity to the PCP (pollen coat protein) gene family. Predicted to encode a PR (pathogenesis-related) protein. Belongs to the **plant defensin (PDF) family** |
|  |  |
| **Bax inhibitor-1** family protein; **inhibits the proapoptotic effect of Bax.** | **Defender against death (DAD family) protein**; Members of this family are thought to be integral membrane proteins. Some members of this family **have been shown to cause apoptosis if mutated.** These proteins are known as DAD for defender against death. The family also includes the epsilon subunit of the oligosaccharyltransferase that is involved in N-linked glycosylation. |
| **CYCLASE1**; Encodes a cyclase-family protein that is a **negative regulator of cell death that regulates pathogen-induced symptom development**. N**egative regulation of programmed cell death**, **response to salt stress,** tryptophan catabolic process to kynurenine. | ACA11, **AUTOINHIBITED CA^2+^-ATPASE 11;** anion homeostasis, **defense response to bacterium, negative regulation of programmed cell death** |
|  | **ARABIDOPSIS BAX INHIBITOR 1**, ATBI-1, ATBI1, BAX INHIBITOR 1, BI-1, BI1; Encodes BI-1, a homolog of mammalian Bax inhibitor 1. **Functions as an attenuator of biotic and abiotic types of cell death**. Bax-induced cell death can be downregulated by ectopically expressing AtBI in planta. ER overload response, **apoptotic process, negative regulation of apoptotic process, negative regulation of programmed cell death, response to water deprivation, very long-chain fatty acid metabolic process** |
|  |  |
| PLANT U-BOX 12; **Encodes a U-box E3 ubiquitin ligase** involved in ubiquitination of pattern recognition receptor **FLS2**. **defense response to bacterium** |  |
|  |  |
| **Glutamate receptor 3.6**; member of Putative ligand-gated ion channel subunit family; cellular calcium ion homeostasis, induced systemic resistance, jasmonic acid mediated signaling pathway, response to light stimulus, **response to wounding.** | **GERANYLLINALOOL SYNTHASE**, GES, **TERPENE SYNTHASE 04**, TERPENE SYNTHASE 4, TPS04, TPS4; Encodes a geranyllinalool synthase that produces a precursor to TMTT, a volatile plant defense C16-homoterpene. GES transcript levels rise in response to alamethicin, a fungal peptide mixture that damages membranes. **This transcriptional response is blocked in JA biosynthetic and JA signaling mutants**, but GES transcript levels still rise in response to alamethicin in mutants with salicylic acid and ethylene biosynthetic and/or signaling defects. **GES transcripts also accumulate in response to a larval infestation.** This enzyme does not localize to the plastids, and it may be present in the cytosol or endoplasmic reticulum. The mRNA is cell-to-cell mobile. |
| **Calcium-dependent phosphotriesterase** superfamily protein; FUNCTIONS IN: strictosidine synthase activity; INVOLVED IN: alkaloid biosynthetic process, biosynthetic process, response to **ethylene**, response to f**ungus**, response to j**asmonic acid, response to salicylic acid, response to virus, response to wounding** | ATTPS21, **TERPENE SYNTHASE 21**, TPS21; Encodes a sesquiterpene synthase involved in generating all of the group A sesquiterpenes found in the Arabidopsis floral volatile blend. Strongly expressed in the stigma. **response to herbivore,** sesquiterpene biosynthetic process, sesquiterpenoid biosynthetic process |
| **Ralf-like 33** | **Wound-responsive** family protein |
|  | Arabidopsis thaliana **WOUND-INDUCED PROTEIN 12**, ATWI-12, SAG20, **SENESCENCE ASSOCIATED GENE 20**, WI12, WOUND-INDUCED PROTEIN 12; A senescence-associated gene whose expression is induced in response to treatment with Nep1, a fungal protein that causes necrosis. The mRNA is cell-to-cell mobile. |
|  | **Serpin 3** |
|  | **Serine protease inhibitor (SERPIN)** family protein |
|  | **OPC-8:0 CoA ligase1** (OPCL1); FUNCTIONS IN: 4-coumarate-CoA ligase activity; INVOLVED IN: phenylpropanoid metabolic process, **jasmonic acid biosynthetic process, response to wounding** |
|  |  |
|  | **Nitrile specifier protein 2; glucosinolate catabolic process, nitrile biosynthetic process**: Intact glucosinolates are believed to be biologically inactive, **whereas degradation products after hydrolysis have multiple roles in growth regulation and defense.** |
|  | **2-ISOPROPYLMALATE SYNTHASE 2**, IMS2, MAM-L, MAM3, METHYLTHIOALKYMALATE SYNTHASE-LIKE; methylthioalkymalate synthase-like. Also known as 2-isopropylmalate synthase (IMS2). encodes a methylthioalkylmalate synthase **involved in the biosynthesis of aliphatic glucosinolates** which accepts all the omega-methylthio-2-oxoalkanoic acids needed to form the known C3 to C8 glucosinolates in Arabidopsis. The mRNA is cell-to-cell mobile |
|  |  |
|  | ATTLP9, TLP9, **TUBBY-LIKE PROTEIN 9**; Member of TLP family; regulation of transcription, DNA-templated, **response to fungus** |
|  | **ENHANCED DOWNY MILDEW 2** |
|  | **BETA GLUCOSIDASE 26**, BGLU26, PEN2, PENETRATION 2; Encodes a glycosyl hydrolase that localizes to peroxisomes and **acts as a component of an inducible preinvasion resistance mechanism**. **Required for mlo resistance**. The mRNA is cell-to-cell mobile. Carbohydrate metabolic process, **defense response by callose deposition in cell wall, defense response to bacterium, defense response to fungus, incompatible interaction, glucosinolate metabolic process, indole glucosinolate catabolic process, induced systemic resistance, response to bacterium.** |
|  | **RESTRICTED TEV MOVEMENT 3**, RTM3**; defense response to virus**, transport of virus in host, tissue to tissue. |
|  | **IAA-LEUCINE RESISTANT (ILR)-LIKE GENE 5**, ILL5 encodes a member of the six Arabidopsis IAA-amino acid conjugate hydrolase subfamily and conjugates, and is very similar to IAR3. metabolic process, proteolysis**, regulation of systemic acquired resistance.** |
|  | **Phloem protein 2 A5 (PP2-A5**); Functions in: carbohydrate binding; Involved in: **signal transduction, defense response, innate immune response.** |
|  | **CXC750, ECS1**; Encodes cell wall protein. ECS1 is not a Xcc750 resistance gene, but the genetic data indicate that ECS1 is **linked to a locus influencing resistance to Xcc750.** The mRNA is cell-to-cell mobile. |
|  |  |
|  | **Peptidoglycan-binding LysM domain**-containing protein |
|  | **Peptidoglycan-binding LysM domain**-containing protein |
|  | **Peptidoglycan-binding LysM domain**-containing protein |
|  |  |
|  |  |

| **CELL WALL RELATED-SUGAR MODIFICATION-SYNTHESIS** | |
| --- | --- |
| **INDUCED** | **REPRESSED** |
| **UDP-glucosyl transferase 73C6** | **O-fucosyltransferase** family protein |
| **UDP-glucosyl transferase 74D1** | **DOLICHOL PHOSPHATE MANNOSE SYNTHASE 2**, DPMS2; Encodes a subunit of the dolichol phosphate mannase synthase (DPMS) complex that may serve as membrane anchors for the catalytic core, DPMS1, or provide catalytic assistance. **It is localized in the ER and mediates isoprenyl-linked glycan biogenesis.** |
| **Galactosyl transferase GMA12/MNN10** family protein | **GALACTURONOSYLTRANSFERASE 7** |
| **N-acetylglucosamine-1-phosphate uridylyltransferase 1** | **ALG6, ALG8 glycosyltransferase** family |
|  | **UDP-GLUCOSYL TRANSFERASE 73C2** |
|  |  |
| **Cellulose synthase** family protein | cellulose synthase like; **CELLULOSE SYNTHASE LIKE A7** |
|  | **Cellulose synthase 10** |
|  |  |
| **COBRA-like extracellular glycosyl-phosphatidyl inositol-anchored protein** family; are likely to be involved in extracellular matrix remodeling and signaling; Involved in: **response to salt stress, multidimensional cell growth, cellulose microfibril organization.** | TBL36, **TRICHOME BIREFRINGENCE-LIKE 36**; Encodes a member of the TBL (TRICHOME BIREFRINGENCE-LIKE) gene family containing a plant-specific DUF231 (domain of unknown function) domain. TBL gene family has 46 members, two of which (TBR/AT5G06700 and TBL3/AT5G01360**) have been shown to be involved in the synthesis and deposition of secondary wall cellulose, presumably by influencing the esterification state of pectic polymers.** |
|  |  |
| **ADP glucose pyrophosphorylase large subunit 1** | **Isoamylase 1** |
| **Plant glycogenin-like starch initiation protein 4** |  |
| **Alpha 1,4-glycosyltransferase** family protein |  |
|  |  |
| **O-Glycosyl hydrolases family 17 protein** | Endonuclease or **glycosyl hydrolase** |
| **Cellulase (glycosyl hydrolase family 5)** protein | **Alpha-fucosidase 1** |
| **Glycosyl hydrolase family 10** protein | **Cell wall / vacuolar inhibitor of fructosidase 2** |
| **Glycosyl hydrolase** superfamily protein | **Cytosolic invertase 2** |
| **Glycosyl hydrolase** superfamily protein | **BETA GLUCOSIDASE 13**, BGLU13 |
| **Glycosyl hydrolase** superfamily protein | **O-Glycosyl hydrolases family 17** protein |
| **Beta-galactosidase 11** | **Beta-galactosidase-related** protein |
| **Beta-galactosidase related** protein |  |
| **Beta glucosidase 2** |  |
| **Beta galactosidase 1** |  |
|  |  |
| **Plant invertase/pectin methylesterase inhibitor** superfamily | **Plant invertase/pectin methylesterase inhibitor** superfamily |
| **Plant invertase/pectin methylesterase inhibitor** superfamily | **Plant invertase/pectin methylesterase inhibitor** superfamily |
| **Plant invertase/pectin methylesterase inhibitor** superfamily | **Plant invertase/pectin methylesterase inhibitor** superfamily |
| **Plant invertase/pectin methylesterase inhibitor** superfamily |  |
| **Plant invertase/pectin methylesterase inhibitor** superfamily |  |
| **Plant invertase/pectin methylesterase inhibitor** superfamily |  |
|  |  |
| **Pectin lyase-like** superfamily protein | **Pectin lyase-like** superfamily protein |
| **Pectin lyase-like** superfamily protein | **Pectin lyase-like** superfamily protein |
| **Pectin lyase-like** superfamily protein |  |
| **Pectin lyase-like** superfamily protein |  |
|  |  |
| **Pectinacetylesterase** family protein | **Pectinacetylesterase** family protein |
|  | **Pectinacetylesterase** family protein |
|  |  |
| **UDP-GLUCOSE DEHYDROGENASE 2**, UGD2; Encodes one of four UDP-glucose dehydrogenase UGD) genes. Mutation of this gene in combination with UGD3 leads to swollen plant cell walls and severe developmental defects associated with changes in pectic polysaccharides. **UDP-glucuronate biosynthetic process, carbohydrate metabolic process, cell wall pectin metabolic** process, glycosaminoglycan biosynthetic process, | **VANGUARD 1 HOMOLOG 2**, VGDH2; VANGUARD 1 homolog 2 (VGDH2); Functions in: enzyme inhibitor activity, **pectinesterase activity; cell wall modification, pectin catabolic process.** |
|  |  |
| **Bifunctional inhibitor/lipid-transfer protein/seed storage 2S albumin** superfamily protein | **Bifunctional inhibitor/lipid-transfer protein/seed storage 2S albumin** superfamily protein |
| **Bifunctional inhibitor/lipid-transfer protein/seed storage 2S albumin** superfamily protein |  |
| **Bifunctional inhibitor/lipid-transfer protein/seed storage 2S albumin** superfamily protein |  |
|  |  |
| **glycine-rich protein** | **glycine-rich** protein |
|  | **glycine/proline-rich** protein |
|  |  |
| **Proline-rich extensin-like** family protein | **Proline-rich extensin-like** family protein |
|  | **Proline-rich extensin-like** family protein |
|  | **Pollen Ole e 1 allergen and extensin** family protein |
|  | **Leucine-rich repeat/extensin 2** |
|  |  |
| **Hydroxyproline-rich glycoprotein** family protein | **Hydroxyproline-rich glycoprotein** family protein |
|  | **Hydroxyproline-rich glycoprotein** family protein |
|  | **Hydroxyproline-rich glycoprotein** family protein |
|  | **Hydroxyproline-rich glycoprotein** family protein |
|  | **Hydroxyproline-rich glycoprotein** family protein |
|  | **Hydroxyproline-rich glycoprotein** family protein |
|  | **Hydroxyproline-rich glycoprotein** family protein |
|  |  |
| **FASCICLIN-like arabinogalactan protein 17** precursor | **FASCICLIN-like arabinogalactan protein 5** precursor |
|  | **Arabinogalactan protein 21** |
|  | **Arabinogalactan protein 4** |
|  |  |
| **FAD-binding Berberine** family protein | **FAD-binding Berberine** family protein |
|  |  |
| **Cinnamyl alcohol dehydrogenase homolog 3** |  |
|  |  |
| **Tubulin beta-9 chain** | **Dynamin related protein 4A** |
| **Myosin heavy chain-related** | **Actin-binding FH2 (formin homology 2)** family protein |
| **Myosin heavy chain-related** | **Actin-11** |
| **Myosin heavy chain-related** | **Gamma-tubulin complex protein 2** |
| Myosin heavy chain-related (TAIR:AT5G32590.1) | PFD3, **PREFOLDIN 3; prefoldin 3** (PFD3); Functions in: unfolded protein binding**; microtubule-based process, protein folding, tubulin complex assembly.** |
| similar to **filament protein-related** [Arabidopsis thaliana]. | **ATP binding microtubule motor** family protein |
|  | **Myosin heavy chain-related** protein |
|  | **Tubulin alpha-4 chain** |
|  | **Microtubule end binding protein EB1A** |
|  |  |
| ATFH5, FH5, **FORMIN HOMOLOGY 5**, FORMIN HOMOLOGY5; **Encodes a protein with similarity to formins that is involved in cytokinesis**. Loss of function mutations exhibit delayed cellularization during endosperm development. FH5 is expressed in the endosperm and the protein localizes to the cell plate. FH5 was shown to be a maternally expressed imprinted gene. **Actin filament polymerization, actin nucleation,** **endosperm development, seed morphogenesis.** |  |
| **Q-DOMAIN 29**, IQD29; **Encodes a microtubule-associated protein.** |  |
|  |  |
| **Leucine-rich receptor-like protein kinase** family protein | **Leucine-rich repeat protein kinase** family protein |
| **Leucine-rich repeat protein kinase** family protein | **Leucine-rich repeat protein kinase** family protein |
| **Leucine-rich repeat transmembrane protein kinase** protein | **Leucine-rich receptor-like protein kinase** family protein |
| **Leucine-rich repeat protein kinase** family protein |  |
| **Leucine-rich repeat (LRR)** family protein |  |
| **Leucine-rich receptor-like protein kinase** family protein |  |
|  |  |
|  | **Plasmodesmata callose-binding protein 1** |
|  | **Carbohydrate-binding X8 domain superfamily** protein; **plasmodesmata callose-binding protein 3** |
|  |  |
|  |  |

| **TRANSCRIPTION FACTORS** | |
| --- | --- |
| **INDUCED** | **REPRESSED** |
| **C2H2-like zinc finger** protein | **C2H2 and C2HC zinc fingers** superfamily protein |
| **C2H2-type zinc finger** family protein | **C2H2-like zinc finger** protein |
| **C2H2 and C2HC zinc fingers** superfamily protein | **C2H2-like zinc finger** protein |
|  | SUF4, **SUPPRESSOR OF FRIGIDA 4**; Encodes SUF4 (SUPPRESSOR of FRI 4; **zinc finger (C2H2 type) family protein**), a putative zinc-finger-containing **transcription factor that is required for delayed flowering in winter-annual Arabidopsis. suf4 mutations strongly suppress the late-flowering phenotype of FRI (FRIGIDA) mutants. suf4 mutants also show reduced H3K4 trimethylation at FLC (FLOWERING LOCUS C), a floral inhibitor. SUF4 may act to specifically recruit a putative histone H3 methyltransferase EFS (EARLY FLOWERING IN SHORT DAYS) and the PAF1-like complex to the FLC locus. Cell** differentiation, flower development, negative regulation of flower development |
|  | **C2H2 and C2HC zinc fingers** superfamily protein |
|  | **C2H2 and C2HC zinc fingers** superfamily protein |
|  |  |
| **NAC domain containing protein 36** |  |
| **NAC-domain protein 101** |  |
| **NAC domain containing protein 75** |  |
| **NAC domain containing protein 47** |  |
|  |  |
| **Myb domain protein 58** | **Myb domain protein 76** |
| **Myb domain protein 65** | Homeodomain-like superfamily protein; LCL5, **LHY-CCA1-LIKE 5**, REVEILLE 8, RVE8; **Encodes a MYB-like transcription factor similar to CIRCADIAN CLOCK-ASSOCIATED1 (CCA1) and ELONGATED HYPOCOTYL (LHY). Involved in the regulation of circadian clock by modulating the pattern of histone 3 (H3) acetylation.** Response to **abscisic acid, response to auxin, response to cadmium ion, response to ethylene, response to gibberellin, response to jasmonic acid, response to salicylic acid**, response to salt stress, transcription, DNA-templated |
| **Myb domain protein 117** | **MYB-like 102**; Encodes a MYB transcription factor **involved in wounding and osmotic stress response**. Member of the R2R3 factor gene family. involved in regulation of transcription, DNA-templated, **response to abscisic acid, response to osmotic stress, response to salt stress, response to wounding** |
|  | Myb domain protein 16; response to **auxin, r**esponse to cadmium ion, response **to ethylene**, response to gibberellin, response to j**asmonic acid, response to salicylic acid, response to salt stress**. Member of the R2R3 factor gene family. |
|  |  |
|  | **KANADI 3 (KAN3)**; CONTAINS InterPro DOMAIN/s: Homeodomain-like (InterPro:IPR009057), **Myb, DNA-binding**, Myb-like DNA-binding domain, **SHAQKYF class** (InterPro, **Homeodomain-related,** Homeodomain-like superfamily protein. **cell differentiation, multicellular organismal development, regulation of transcription.** |
|  | ATMYB3R2, **C-MYB-LIKE TRANSCRIPTION FACTOR 3R-2**, MYB DOMAIN PROTEIN 3R2, MYB3R-2, MYB3R2, PC-MYB2**; Encodes a putative c-myb-like transcription factor.** Member of a class of domain proteins containing structural features of the vertebrate c-Myb proto-oncoprotein, including the presence of three Myb motifs (R1, R2, R3). |
|  | ATMYB82, **MYB DOMAIN PROTEIN 82**, MYB82; **trichome differentiation** |
|  | ATMYB67, ATY53, **MYB DOMAIN PROTEIN 67**, MYB67; Member of the R2R3 factor gene family. |
|  | **Myb domain protein 103** |
|  |  |
| **Homeodomain GLABROUS 7** | **Homeodomain GLABROUS 8** |
| **Homeodomain GLABROUS 1** | **Homeodomain-like superfamily** protein |
| **BEL1-like homeodomain 6** | **TRIPTYCHON**, TRY; **Homeodomain-like superfamily** protein; **multicellular organismal development.** |
| **Homeodomain-like superfamily** protein |  |
| **Homeodomain-like superfamily** protein |  |
| **Duplicated homeodomain-like superfamily** protein |  |
| **Duplicated homeodomain-like superfamily** protein |  |
|  |  |
| **WUSCHEL related homeobox 7** |  |
|  |  |
| **WRKY DNA-binding protein 59** | **WRKY DNA-binding protein 23** |
|  |  |
| **Nuclear factor Y, subunit B3** |  |
|  |  |
| **AGAMOUS-like 91**; Hasta ahora se distinguen 5 genes diferentes involucrados en la identidad floral de Arabidopsis: APETALA 1 (AP1), APETALA2 (AP2), APETALA3 (AP3), PISTILLATA (P1) Y **AGAMOUS (AG); MADS-domain proteins are found in plants, animals, and fungi and often play critical roles in the control of development** | **AGAMOUS-like 38** |
| **K-box region and MADS-box transcription factor** family protein; Muchas plantas presentan regiones conservadas, tales como la K-box, que posee de 237 a 259 pb y se cree que está involucrada en la dimerización de proteínas. Aquellos genes MADS-BOX de plantas que posean esa caja K se conocen como genes MIK o MIKC. La estructura MIKC se ha visto en musgos y en otras embriofitas.6 **La función mejor conocida de estos genes es la que da lugar al modelo ABC del desarrollo floral.** | **AGAMOUS-like 20** |
|  | **AGAMOUS-like 80**; **MADS-box transcription factor** family protein |
|  | **AGAMOUS-like 97** |
|  |  |
| related to **AP2 1; RAP2.1, RELATED TO AP2 1** encodes a member of the DREB subfamily A-5 of **ERF/AP2** transcription factor family (RAP2.1). **The protein contains one AP2 domain. There are 16 members in this subfamily including RAP2.9 and RAP2.10.** | encodes a member of the **ERF (ethylene response factor) subfamily B-1 of ERF/AP2 transcription factor** family. The protein contains one AP2 domain. There are 15 members in this subfamily including **ATERF-3, ATERF-4, ATERF-7, and leafy petiole.** |
| related to **AP2 2; encodes a member of the ERF (ethylene response factor) subfamily B-2 of ERF/AP2 transcription factor family (RAP2.2).** The protein contains one AP2 domain. **There are 5 members in this subfamily including RAP2.2 AND RAP2.12.** | encodes a member of the **DREB subfamily A-5 of ERF/AP2 transcription factor family**. The protein contains one AP2 domain. **There are 16 members in this subfamily including RAP2.1, RAP2.9 and RAP2.10.** |
| **Ethylene responsive element binding factor 5** | **Ethylene-responsive element binding factor 15** |
|  |  |
|  | **GATA TRANSCRIPTION FACTOR 13, GATA13** |
|  |  |
|  | **E2F/DP family winged-helix DNA-binding domain**; E2F was named because it is an adenovirus E2 gene promoter binding factor ([30](http://www.ncbi.nlm.nih.gov/pmc/articles/PMC3190776/#B30)). **E2F proteins play important roles in regulating cell cycle progression, cell proliferation, and differentiation.** |
|  |  |
|  | **Basic helix-loop-helix (bHLH) DNA-binding superfamily** protein; **MYC transcription factors are key transcriptional regulators in the expression of jasmonate (JA)-responsive genes, positively regulating wound resistance genes and acting as negative regulators during the expression of pathogen defense genes.** |
|  | **Basic helix-loop-helix (bHLH) DNA-binding superfamily** protein; **ELONGATED HYPOCOTYL 5**, HY5, REVERSAL OF THE DET PHENOTYPE 5, TED 5**. Gibberellic acid mediated signaling pathway. Photomorphogenesis, positive regulation of anthocyanin metabolic process, positive regulation of circadian rhythm, red or far-red light signaling pathway, red, far-red light phototransduction, regulation of photomorphogenesis. Regulation of transcription, DNA-templated, response to UV-B**, **response to abscisic acid, response to far red light, response to karrikin, response to red light.** |
|  | **Basic helix-loop-helix (bHLH) DNA-binding superfamily** protein; BHLH096, **BHLH96 (-2.44)** |
|  | **Basic helix-loop-helix (bHLH) DNA-binding** superfamily protein; **Encodes POPEYE (PYE), a bHLH transcription factor regulating response to iron deficiency** in Arabidopsis roots |
|  | **Basic helix-loop-helix 32**; **TARGET OF MONOPTEROS 5**, TMO5  Encodes a basic helix-loop-helix transcription factor **that is expressed in the hypophysis-adjacent embryo cells, and is required and partially sufficient for MP-dependent root initiation. Involved in response to phosphate starvation.** **Negative regulator of root hair development, anthocyanin formation and Pi content**. Its expression is responsive to both phosphate (Pi) and phosphite (Phi) in shoots. |
|  | **Basic helix-loop-helix (bHLH) DNA-binding superfamily** protein. |
|  | ***A. thaliana* INDUCER OF CBP EXPRESSION 1**, ATICE1, ICE1, INDUCER OF CBF EXPRESSION 1, SCREAM, SCRM; **Encodes a MYC-like bHLH transcriptional activator that binds specifically to the MYC recognition sequences in the CBF3 promoter. Mutants are defective in cold-regulated gene expression.** Cold stress triggers protein degradation of nuclear GFPICE1 protein, and the RING finger protein HOS1 is required. **Sumoylation of ICE1 controls CBF3/DREB1A expression and freezing tolerance**. |
|  | **Basic helix-loop-helix (bHLH) DNA-binding superfamily** protein; BHLH096, **BHLH96** |
|  | **TCP domain protein 17**; TCP genes encode plant-specific transcription factors **with a bHLH motif**. **The TCP gene family has >20 members in the eudicots. Control of cell proliferation in developing tissues.** |
|  |  |
|  | **Basic leucine-zipper 52** |
|  |  |
|  | **GATA transcription factor 13** |
|  |  |
|  | **Dof-type zinc finger DNA-binding family** protein |
|  | **Dof-type zinc finger DNA-binding family protein; ATDOF5.8** |
|  |  |
| **Basic transcription factor 3**; **regulates transcription**  **of pathogenesis-related genes during hypersensitive response upon Tobacco mosaic virus infection in Capsicum**; The b-subunit of NAC was originally identified as RNA polymerase B transcription factor 3 (Btf3), and was demonstrated to form a complex that is transcriptionally active and associated with apoptosis in mammalian cells. In Caenorhabditis elegans, the inhibitor of cell death-1 gene (ICD-1) that encodes the b-NAC functions in **preventing apoptosis. Furthermore, the BTF3-responsive protein interaction network revealed 20 key protein species, most of which are regulated by abscisic acid, ethane, or oxidative stress**; **TaBTF3-silenced wheat plants showed lower survival rates, free proline content, and relative water content and higher relative electrical conductivity and water loss rate.** | **Transcriptional factor B3** family |
|  |  |
| **HIGH-MOBILITY GROUP BOX 6** | **TARGET OF EARLY ACTIVATION TAGGED (EAT) 2**, TOE2, target of early activation tagged (EAT) 2 (TOE2); Functions in: DNA binding, sequence-specific DNA binding transcription factor activity; **ethylene-activated signaling pathway, multicellular organismal development.** |
|  |  |
|  | **Transcription factor IIS family protein; Transcription elongation factor.** |
|  |  |
|  |  |

| **PHYTOHORMONE-DEVELOPMENT-ABIOTIC STRESS** | |
| --- | --- |
| **INDUCED** | **REPRESSED** |
| **ABA-STOMATAL OPENING** | |
| AHA1, **H (+)-ATPASE 1, HA1, OPEN STOMATA 2**, OST2, PLASMA MEMBRANE PROTON ATPASE, PMA; Encodes a plasma membrane proton ATPase. **Mutants have a reduced ability to close their stomata** **in response to drought and are affected in stomatal but not seed responsiveness to ABA.** | ATRZPF34, **CHY ZINC-FINGER AND RING PROTEIN 1**, CHYR1, RING ZINC-FINGER PROTEIN 34, RZPF34; Encodes a protein with sequence similarity to RING, zinc finger proteins. **Loss of function mutations show reduced (15%) stomatal aperture under non stress conditions.** **Regulation of stomatal opening** |
|  | Regulatory component of **ABA receptor 1** |
|  | **HVA22-like protein H (**ATHVA22H) (HVA22H); Contains InterPro DOMAIN/s: TB2/DP1/HVA22 related protein**. Abscisic acid-responsive (TB2/DP1, HVA22) family protein.** |
|  | **ALDEHYDE DEHYDROGENASE 10A9**, ALDH10A9, BADH, **BETAINE ALDEHYDE DEHYDROGENASE**; ALDH10A9 encodes a protein that can function as a **betaine aldehyde dehydrogenase** in vitro. The C-terminal amino acids of this protein direct GFP to the peroxisome suggesting that ALDH10A9 accumulates in this organelle. ALDH10A9 transcript levels rise in response **to ABA, NaCl, chilling, methyl viologen, and dehydration stress.** **The enzyme can catalyze the formation of glycine betaine in vitro,** but there are still questions about whether Arabidopsis makes this protective compound under natural conditions. **This enzyme may be involved in oxidizing aminoaldehydes formed through polyamine metabolism. oxidation-reduction process, response to abscisic acid, response to water deprivation.** |
|  | **Inositol monophosphatase family** protein; **ALTERED EXPRESSION OF APX2 8**, ALX8, ATFRY1, ATSAL1, FIERY1, FRY1, HIGH EXPRESSION OF OSMOTICALLY RESPONSIVE GENES 2, HOS2, RON1, ROTUNDA 1, SAL1, SUPO1, SUPPRESSORS OF PIN1 OVEREXPRESSION 1**; Encodes a bifunctional protein that has 3'(2'),5'-bisphosphate nucleotidase and inositol polyphosphate 1-phosphatase activities and rescues sulfur assimilation mutants in yeast.** **It is involved in the response to cold, drought (negative regulator of drought tolerance), and ABA. Mutants in this gene exhibit enhanced induction of stress genes in response to cold, ABA, salt and dehydration due to higher accumulation of the second messenger, inositol (1,4,5)- triphosphate (IP(3)).** Involved in degradation of small mRNAs. Mutants also affect the accumulation of miRNA target cleavage products. Regulates light-dependent repression of hypocotyl elongation and flowering time via its 3'(2'),5'-bisphosphate nucleotidase activity. RNA catabolic process, **abscisic acid-activated signaling pathway**, miRNA catabolic process, negative regulation of signal transduction, negative regulation of transcription, DNA-templated, phosphatidylinositol-mediated signaling, photoperiodism, flowering, positive regulation of unidimensional cell growth, **regulation of jasmonic acid** **biosynthetic process, response to cation stress**, **response to cold, response to freezing, response to light stimulus, response to salt stress, response to water deprivation, sulfur compound metabolic process.** |
|  | **Encodes a substrate of the MAPK kinases**. Phenotypic analyses of Arabidopsis expressing **phosphorylation site mutant forms** of At1g80180.**1 showed clustered stomata and higher stomatal index in cotyledons expressing the phosphomimetic form of At1g80180.1**. **Stomatal complex patterning.** |
|  | ATSYP132, **SYNTAXIN OF PLANTS 132**, SYP132; exocytosis, intracellular protein transport, **response to abscisic acid,** **vesicle docking, vesicle fusion.** |
|  |  |
|  |  |
| **DEHYDRATION-, STRESS-INDUCED PROTEINS** | |
| **Dehydrin family** protein | **Dehydration-induced protein (ERD15)** |
| **Low-temperature-responsive protein 78 (LTI78) / desiccation-responsive protein 29A (RD29A)** | **STRESS-ASSOCIATED PROTEIN 7** |
| **Stress enhanced protein 2** | **Early-responsive to dehydration stress protein (ERD4)** |
| ATGRP8, CCR1, **COLD, CIRCADIAN RHYTHM, AND RNA BINDING 1**, **GLYCINE-RICH PROTEIN 8**, GLYCINE-RICH RNA-BINDING PROTEIN 8, GR-RBP8, GRP8, RBGA6, RNA-BINDING GLYCINE-RICH PROTEIN A6; **Encodes a glycine-rich protein with RNA binding domain at the N-terminus. Protein is structurally similar to proteins induced by stress in other plants**. **Gene expression is induced by cold.** Transcript undergoes circadian oscillations that is depressed by overexpression of AtGRP7. **A substrate of the type III effector HopU1 (mono-ADP-ribosyltransferase).** | **BTB and TAZ domain protein**. Acts reduntantly with BT1 and BT2 during female gametophyte development. Acts with BT2 during male gametophyte development**. Also in response to cold, response to ethylene, response to hydrogen peroxide, response to salicylic acid, response to salt stress.** |
| **Quinone reductase family** protein; **It is hypothesized that the auxin-inducible glutathione S-transferases and quinone reductases found in plants also act as detoxification enzymes, possibly to protect against auxin-induced oxidative stress.** | F3'H, F3H, **FLAVANONE 3-HYDROXYLASE**, **TRANSPARENT TESTA 6, TT6**; Encodes flavanone 3-hydroxylase that is coordinately expressed with chalcone synthase and chalcone isomerases. **Regulates flavonoid biosynthesis. Not responsive to auxin or ethylene stimulus (qRT-PCR). Flavonoid biosynthetic process, oxidation-reduction process, response to UV-B.** |
|  | **Galactinol synthase 7** |
|  | **DRE-binding protein 2A** |
|  | **Early-responsive to dehydration protein-related / ERD protein-related. Major facilitator superfamily protein**. |
|  | ATNLP1, CPA, **NITRILASE-LIKE PROTEIN 1**, NLP1; Encodes N-carbamoylputrescine amidohydrolase **that is involved in putrescine and polyamine biosynthesis.** |
|  | **Expression of the gene is downregulated in the presence of paraquat, an inducer of photoxidative stress. Response to oxidative stress.** |
|  | CBL-INTERACTING PROTEIN KINASE 11, CIPK11, PKS5, PROTEIN KINASE SOS2-LIKE 5, SIP4, **SNF1-RELATED PROTEIN KINASE 3.22**, SNRK3.22, SOS3-INTERACTING PROTEIN 4; **Encodes a SOS2-like protein kinase that is a member of the CBL-interacting protein kinase family.** Loss of function mutants show a decrease in sensitivity to high pH.Phosphorylates AHA2, a plasma membrane H+ ATPase. This phosphorylation appears to regulate the activity of the proton transporter. |
|  | BTI1, **RETICULAN LIKE PROTEIN B1**, RTNLB1, VIRB2-INTERACTING PROTEIN 1, endoplasmic reticulum tubular network organization: **Consistent with this observation, transgenic plants overexpressing soybean BiP have demonstrated to exhibit increased tolerance to ER (endoplasmic reticulum) stressors during seed germination and enhanced tolerance to water deficit during plant growth**. Chaperonins? |
|  |  |
|  | ZIFL1, **ZINC INDUCED FACILITATOR-LIKE 1; basipetal auxin transport, gravitropism, potassium ion transmembrane transport, regulation of stomatal closure, response to karrikin, response to water deprivation,** root development |
|  | ABCG13, **ATP-BINDING CASSETTE G13**; **cutin transport**, petal epidermis patterning, **response to salt stress** |
|  | **ABC2 homolog 4**; lipid transport; ABCG13, ATP-BINDING CASSETTE G13; ABC-2 type transporter family protein; **cutin transport, petal epidermis patterning, response to salt stress** |
|  | **TMPIT-like protein** (TMPIT (transmembrane protein inducible by TNF-α) family,); Real-time RT-PCR showed that the **TdicTMPIT1 gene is upregulated on drought stress in drought-tolerant wild emmer wheat, but not in a drought-sensitive accession or in cultivated durum wheat.** |
|  | **A20⁄AN1 zinc-finger containing stress-associated**  **proteins**; A variety of rice genes, belonging to diverse regulatory networks, have been assessed functionally **to impart abiotic**  **stress tolerance in plants to help protect against severe yield**  **loss**. **Among them, A20 ⁄AN1 zinc-finger domain**  **containing stress-associated proteins (SAPs) have emerged**  **as important candidates although their mechanism of action**  **is not yet known** |
|  | EXL1, **EXORDIUM LIKE 1,** PHI-1, PHOSPHATE-INDUCED 1  EXL1 is involved in the C-starvation response. growth, response to hypoxia; We hypothesize that EXL1 suppresses **brassinosteroid-dependent growth and controls C allocation in the cell.** However, experimental evidence in Arabidopsis  suggests **that adjustments to mild drought stress costs only a small fraction of the assimilated C**. Since growth is reduced more than photosynthesis, drought may lead **to a positive C balance** |
|  | **ALDEHYDE DEHYDROGENASE 10A9**, ALDH10A9, BADH, **BETAINE ALDEHYDE DEHYDROGENASE**; ALDH10A9 encodes a protein that can function as a betaine aldehyde dehydrogenase in vitro. ***ALDH10A9* transcript levels rise in response to ABA, NaCl, chilling, methyl viologen, and dehydration stress**. This enzyme may be involved in oxidizing aminoaldehydes formed **through polyamine metabolism.** |
|  |  |
|  |  |
| **AUXINS** | |
| **SAUR-like auxin-responsive protein** family | **YUCCA 9; Flavin-binding monooxygenase** family protein |
| **Auxin efflux carrier family** protein | **Auxin efflux carrier** family protein |
| **AUX/IAA transcriptional regulator** family protein | **SHI-RELATED SEQUENCE3**, SRS3; A member of SHI gene family. *Arabidopsis thaliana* has ten members that encode proteins with a RING finger-like zinc finger motif. Despite being highly divergent in sequence, many of the SHI-related genes are partially redundant in function and synergistically promote gynoecium, stamen and leaf development in Arabidopsis. **Auxin biosynthetic process, auxin-activated signaling pathway, multicellular organismal development** |
| **Late embryogenesis abundant 3 (LEA3)** family protein | **Auxin-responsive GH3** family protein |
| **Late embryogenesis abundant 3 (LEA3)** family protein | ABCG37, **ATP-BINDING CASSETTE G37**, ATPDR9, PDR9, PIS1, PLEIOTROPIC DRUG RESISTANCE 9, **POLAR AUXIN TRANSPORT INHIBITOR SENSITIVE 1**; **Negative regulator of auxin polar transport inhibitors. ABCG37 regulates auxin distribution and homeostasis in roots by excluding IBA from the root apex, but does not act directly in basipetal transport**. ABCG37 and ABCG36 act redundantly at outermost root plasma membranes and, transport IBA out of the cells. Also involved in root transmembrane secretion of fluorescent phenolics involved in Fe uptake. The mRNA is cell-to-cell mobile |
| **IAA-leucine resistant 2** | **Late embryogenesis abundant (LEA) hydroxyproline-rich glycoprotein** family |
| EMB30, **EMBRYO DEFECTIVE 30**, GN, **GNOM,** VAN7, VASCULAR NETWORK 7; **Encodes a GDP/GTP exchange factor for small G-proteins of the ADP ribosylation factor (RAF) class, and as regulator of intracellular trafficking**. **Basipetal auxin transport**, cell adhesion, cell wall organization, cytokinesis by cell plate formation, embryo development ending in seed dormancy, embryonic pattern specification, endocytosis, endosome transport via multivesicular body sorting pathway and establishment of planar polarity**. Lateral root formation, longitudinal axis specification, phloem or xylem histogenesis**, protein transport**, regulation of ARF protein signal transduction**, regulation of catalytic activity. **Regulation of vesicle targeting, to, from or within Golgi, root hair cell differentiation, unidimensional cell growth, vesicle-mediated transport.** | **SAUR-like auxin-responsive protein** family; **auxin-activated signaling pathway**, multicellular organismal development, regulation of growth, **response to auxin.** |
|  | **IBA-RESPONSE 3**, IBR3; Encodes a protein with similarity to acyl-CoA dehydrogenases. **Mutations in IBR3 render plants resistant to indole-3-butryic acid, a putative storage form of the biologically active auxin IAA** (indole-3-acetic acid). IBR3 is hypothesized to carry out the second step in a β-oxidation-like process of IBA metabolism in Arabidopsis. Fatty acid beta-oxidation using acyl-CoA dehydrogenase, lipid homeostasis, **root hair elongation.** |
|  | ATYUC2, YUC2, **YUCCA2**; Encodes YUC2**. Catalyzes conversion of IPA (indole-3-pyruvic acid) to IAA (indole-3-acetic acid) in auxin biosynthesis pathway.** |
|  |  |
|  |  |
| **BRASSINOSTEROIDS- GIBBERELLINS** | |
| **General regulatory factor 8**; **14-3-3 PROTEIN G-BOX FACTOR14 KAPPA**; member of 14-3-3 proteins. **This protein is reported to interact with the BZR1 transcription factor involved in brassinosteroid signaling** and may affect the nucleocytoplasmic shuttling of BZR1. | CDG1, **CONSTITUTIVE DIFFERENTIAL GROWTH 1**; Receptor-like cytoplasmic kinase, RLCKVII subfamily. Overexpression causes abnormal differential and elongation growth after organ differentiation. Growth, **positive regulation of brassinosteroid mediated signaling pathway.** |
|  | **ILI1 binding bHLH 1**; **brassinosteroid mediated signaling pathway, gibberellic acid mediated signaling pathway, regulation of growth.** |
|  |  |
|  |  |
| **CYTOKININS** | |
|  | **ARR3, RESPONSE REGULATOR 3**; Type A response regulator highly similar to bacterial two-component response regulators. **Rapidly induced by cytokinin**. **Involved in red-light signaling**. **Acts redundantly with ARR3 in the control of circadian period in a cytokinin-independent manner.** |
|  | **Cytokinin-responsive GATA factor 1** |
|  | ATSOFL1, **SOB FIVE-LIKE 1**, SOFL1; **AtSOFL1 acts redundantly with AtSOFL2 as positive regulator of cytokinin levels. cytokinin metabolic process,** developmental process. |
|  | **Purine permease 12** |
|  |  |
|  |  |
| **ETHYLENE-JASMONIC ACID** | |
| **Ethylene-responsive nuclear protein -related** | ***Arabidopsis thaliana*** **SULFOTRANSFERASE 2A**, ATST2A, ST2A, SULFOTRANSFERASE 2A; **Encodes a sulfotransferase that acts specifically on 11- and 12-hydroxyjasmonic acid.** Transcript levels for this enzyme are increased by treatments with **jasmonic acid (JA),** 12-hydroxyJA, JA-isoleucine, and 12-oxyphytodienoic acid (a JA precursor). **Jasmonic acid metabolic process, response to jasmonic acid. sulfotransferase family protein that is involved in the jasmonic acid metabolism,** ST2A is induced at the end of night under LD/22 °C and SD/28 °C conditions according to the external coincidence mechanism. The results of this study support the idea that the circadian clock orchestrates a variety of hormone-signaling pathways to regulate the photoperiod and temperature-dependent morphogenesis in A. thaliana |
| **Glutamate receptor 3.6**; cellular calcium ion homeostasis, induced systemic **resistance, jasmonic acid mediated signaling pathway, response to light stimulus, response to wounding.** | ACX1, **ACYL-COA OXIDASE 1**, ATACX1; Encodes a medium to long-chain acyl-CoA oxidase. **Catalyzes the first step of fatty acid beta-oxidation. Involved in jasmonate biosynthesis. Gene expression is induced by wounding, drought stress, abscisic acid, and jasmonate.** |
|  | **Senescence associated gene 20** |
|  | PDX1L4, **PUTATIVE PDX1-LIKE PROTEIN 4**; putative PDX1-like protein 4 (PDX1L4); Involved in: **response to ethylene stimulus, pyridoxal phosphate biosynthetic process, response to stress.** |
|  | EIN4, **ETHYLENE INSENSITIVE** **4**; **Ethylene receptor, subfamily 2**. Has serine kinase activity. **Negative regulation of ethylene**-activated signaling pathway, peptidyl-histidine phosphorylation |
|  | **TARGET OF EARLY ACTIVATION TAGGED (EAT) 2**, TOE2; **ethylene-activated signaling pathway**, multicellular organismal development, regulation of transcription |
|  | ATJAC1, **JACALIN-LECTIN LIKE 1**, JACALIN-RELATED LECTIN 35, JAL35, JASMONATE RESPONSIVE 1, JR1; **Encodes a JA-responsive gene that coordinates with GRP7 in shaping plant development through the regulation of RNA processing in Arabidopsis**. AtJAC1 interacts with RNA binding protein GRP7 specifically in the cytoplasm to regulate its nucleocytoplasmic distribution. **Response to cold,** **response to jasmonic acid, response to salt stress, response to wounding, vegetative to reproductive phase transition of meristem.** |
|  |  |
|  |  |
| **GROWTH-DEVELOPMENT-CELL DIVISION** | |
| **Lateral root primordium (LRP) protein-related** | **Encodes a root meristem growth factor (RGF).** Belongs to a family of **functionally redundant homologous peptides that are secreted, tyrosine-sulfated, and expressed mainly in the stem cell area and the innermost layer of central columella cells. RGFs are required for maintenance of the root stem cell niche and transit amplifying cell proliferation.** |
| **Rhodanese/Cell cycle control phosphatase superfamily** protein | **Arginine-rich cyclin 1** |
| **Encodes a root meristem growth factor (RGF**). Belongs to a family of functionally **redundant homologous peptides that are secreted, tyrosine-sulfated, and expressed mainly in the stem cell area and the innermost layer of central columella cells.** RGFs are required for maintenance of the root stem cell niche and transit amplifying cell proliferation. | DEL1, DP-E2F-LIKE 1, E2F-LIKE 3, E2FE, E2L3; **E2F-like protein**, **an inhibitor of the endocycle, preserves the mitotic state of proliferating cells by suppressing transcription of genes that are required for cells to enter the DNA endoreduplication cycle.** |
| **Meristematic receptor-like kinase; IMK3**, MERISTEMATIC RECEPTOR-LIKE KINASE, MRLK; **Protein kinase expressed in meristematic cells. Phosphorylates AGL24** | **CLAVATA 3**/ESR-RELATED 5; ATCLE5, CLAVATA3/ESR-RELATED 5, CLE5; **multicellular organismal development, signal transduction.** |
| **Cyclin T1;1** | **TCP DOMAIN PROTEIN 17**, TCP17, TCP **gene involved in heterochronic control of leaf differentiation.** |
| **Seed gene 3; Gene is expressed preferentially in the embryo** and encodes a unique protein of unknown function. | ATRKD2, RKD2, **RWP-RK DOMAIN CONTAINING 2**, UAS-TAGGED **ROOT PATTERNING 4**, URP4 |
| **KOKOPELLI**, KPL; Encodes KOKOPELLI (KPL). *kokopelli (kpl)* mutants display frequent single-fertilization events indicating that KPL is involved **in double fertilization. KPL and an inversely transcribed gene, ARIADNE14 (ARI14), which encodes a putative ubiquitin E3 ligase, generate a sperm-specific natural cis-antisense siRNA pai**r. In the absence of KPL, ARI14 RNA levels in sperm are increased and fertilization is impaired. , pollen development | **Arabidopsis thaliana NICOTIANAMINE SYNTHASE 3**; nicotianamine biosynthetic process, **phloem transport, pollen development, pollen tube growth.** |
| **NOZZLE, NZZ, SPL, SPOROCYTELESS**; Encodes a putative transcription factor that is required for **the initiation of both micro- and megagametogenesis and is expressed in the sporogenous tissue of the anther and the ovule.** Regulator of anther cell differenctiation. Interacts with TPL and TCP proteins. | **Encodes a root meristem growth factor (RGF).** Belongs to a family of functionally redundant homologous peptides that are secreted, tyrosine-sulfated, and expressed mainly in the stem cell area and the innermost layer of central columella cells. **RGFs are required for maintenance of the root stem cell niche and transit amplifying cell proliferation**. |
| **GEM-RELATED 5**, GER5; Encodes a protein with unknown function that is involved in **hormone-mediated regulation of seed germination/dormancy.** Embryo development ending in seed dormancy, **maintenance of seed dormancy by abscisic acid**, regulation of seed germination, seed germination | **Timeless family protein; regulation of circadian rhythm** |
| ATGEX1**, GAMETE EXPRESSED PROTEIN 1**, GEX1; Encodes a transmembrane domain containing protein that is expressed in pollen germ cells. **Embryo development ending in seed dormancy, embryo sac development, pollen development** | ATCOL2, **B-BOX DOMAIN PROTEIN 3**, BBX3, COL2, CONSTANS-LIKE 2; **homologous to the flowering-time gene CONSTANS (CO) encoding zinc-finger proteins.** |
|  | **Nicotianamine synthase 3**; nicotianamine biosynthetic process, phloem transport, **pollen development, pollen tube growth** |
|  |  |

| **TRANSPORT** | |
| --- | --- |
| **INDUCED** | **REPRESSED** |
| **Major facilitator superfamily** protein; **sugar transporter 1**; **sugar: hydrogen symporter activity.** | **Nodulin-like / Major Facilitator Superfamily** protein; Expressed in: sperm cell, male gametophyte; Expressed during: **L mature pollen stage, M germinated pollen stage** |
| **Major facilitator superfamily** protein; **sugar: hydrogen symporter activity.** |  |
| **Major facilitator superfamily** protein. |  |
|  |  |
| **MATE efflux family** protein | **MATE efflux** family protein |
|  | **MATE efflux family protein** |
|  | **MATE efflux** family protein |
|  | **MATE efflux** family protein |
|  |  |
| **Multidrug resistance-associated protein 11**; Encodes ABCC13/MRP11, a member of the multidrug resistance associated protein MRP/ABCC subfamily. **Its expression is induced by gibberellic acid and downregulated by naphthalene acetic acid, abscisic acid, and zeatin.** | **Multidrug resistance-associated protein 7** |
| **Multidrug resistance-associated protein 3**; encodes an ATP-dependent MRP-like ABC transporter **able to transport glutathione-conjugates as well as chlorophyll catabolites.** **The expression of this gene is upregulated by herbicide safeners such as benoxacor and fenclorim.** |  |
| **Pleiotropic drug resistance 6**; FUnctions in: **ATPase activity, coupled to transmembrane movement of substances.** |  |
| **Multidrug resistance-associated protein 10**; **Golgi apparatus, integral component of membrane**, **plant-type vacuole**, plasma membrane, vacuolar membrane, vacuole. |  |
| **ABC-2 type transporter family protein**; **Encodes a ATP-binding cassette transporter G26 (ABCG26) involved in tapetal cell and pollen development. Required for male fertility and pollen exine formation.** |  |
| **Peroxisomal ABC transporter 1;** . **The gene product promotes germination and represses embryo dormancy. ABI3, ABA1, FUS3 and LEC1 are epistatic to this gene.** Mutants accumulate fatty acyl CoA suggesting a defect in uptake of fatty acyl CoA into the peroxisome; fatty acid beta-oxidation, fatty-acyl-CoA transport, positive regulation of seed germination, |  |
|  |  |
| **Calcium-transporting ATPase**, putative | **Gated outwardly-rectifying K+ channel**; GATED OUTWARDLY-RECTIFYING K+ CHANNEL, GORK; **Encodes a guard cell outward potassium channel.** Belongs to the Shaker family K+ channel. This family includes five groups based on phylogenetic analysis; **Mutants have increased water consumption and limited stomatal closure in response to abscisic and jasmonic acids**. It forms a heteromeric K (out) channels with SKOR. The gene is expressed ubiquitously **in root and the vasculature and guard cells of leaves.** [**Response to cold**](https://www.arabidopsis.org/servlets/TairObject?type=keyword&id=5433)**,** [**response to jasmonic acid**](https://www.arabidopsis.org/servlets/TairObject?type=keyword&id=11419)**,** [**response to water deprivation**](https://www.arabidopsis.org/servlets/TairObject?type=keyword&id=5647)**.** |
| **Cation/H+ exchanger 16** | **Sodium Bile acid symporter family; sodium ion transport**, transport |
| **Potassium transporter 2** | **Mechanosensitive channel of small conductance-like 5**; [**anion transport**](https://www.arabidopsis.org/servlets/TairObject?type=keyword&id=5125)**,** [**detection of mechanical stimulus**](https://www.arabidopsis.org/servlets/TairObject?type=keyword&id=18334)**,** [transmembrane transport](https://www.arabidopsis.org/servlets/TairObject?type=keyword&id=28452) |
|  | **FH protein interacting protein FIP2**; **potassium channel tetramerisation domain-containing protein** / **pentapeptide repeat-containing protein.** |
|  | BOR2, **REQUIRES HIGH BORON 2**; **HCO3- transporter family; Functions in: anion exchanger activity;** [root development](https://www.arabidopsis.org/servlets/TairObject?type=keyword&id=18902) |
|  |  |
| **Nucleotide-sugar transporter** family protein |  |
|  |  |
|  | **Equilibrative nucleoside transporter 4** |
|  |  |
| **Mitochondrial substrate carrier** family protein | **Translocase of the inner mitochondrial membrane 13** |
| **Mitochondrial substrate carrier** family protein. **Encodes a predicted calcium-dependent S-adenosyl methionine carrier**. | **Mitochondrial import inner membrane translocase subunit Tim17/Tim22/Tim23** family protein. |
| **Mitochondrial substrate carrier** family protein; **Encodes a mitochondrial thiamin diphosphate carrier.** |  |
| ATOEP16-2, *ATOEP16-S*; **Mitochondrial import inner membrane translocase subunit Tim17/Tim22/Tim23 family protein**; Predominantly expressed in seed and is not inducible by cold treatment. AtOEP16-S gained an additional exon. **The promoter region of atOEP16-S (but not atOEP16-L) contains multiple G-box ABA-responsive elements.** The AtOEP16-S promoter conferred developmentally regulated seed- and pollen-specific GUS expression in tobacco |  |
|  |  |
| **Heavy metal transport/detoxification** superfamily protein | **Copper transport protein** family |
| **Vacuolar iron transporter (VIT)** family protein; The gene encodes a putative nodulin-like21 protein. | **Stabilizer of iron transporter SufD / Polynucleotidyl transferase** |
|  | **ZIP metal ion transporter** family |
|  | **Zinc transporter 5** precursor |
|  | **Heavy metal transport/detoxification** superfamily protein |
|  |  |
| **Transmembrane amino acid transporter** family protein | **Transmembrane amino acid transporter** family protein |
| **Oligopeptide transporter OPT** superfamily protein | AATL2, **AMINO ACID TRANSPORTER-LIKE PROTEIN 2**, ARABIDOPSIS LYSINE HISTIDINE TRANSPORTER 2, ATLHT2, LHT2, LYSINE HISTIDINE TRANSPORTER 2; **High-affinity transporter for neutral and acidic amino acids, expressed in tapetum tissue of anthers.** |
|  | **Amino acid permease** family protein |
|  | **Amino acid transporter 1** |
|  | **Transmembrane amino acid transporter** family protein |
|  | **Major facilitator superfamily** protein; INVOLVED IN: **oligopeptide transport**. |
|  |  |
| **Phosphate transporter 1;1** | **Phosphate transporter 4;3** |
| **Inorganic phosphate transporter** family protein |  |
|  |  |
| CLA-2, ACLA2, **ATP-CITRATE LYASE A-2**; One of the three genes encoding subunit A of the trimeric enzyme ATP Citrate lyase; acetyl-CoA biosynthetic process, lipid metabolic process. In plants, **ATP citrate lyase generates cytosolic acetyl-CoA precursor of thousands of specialized metabolites including waxes, sterols, and polyketides.** |  |
|  |  |
| **Polyketide cyclase / dehydrase and lipid transport** protein | **Polyketide cyclase/dehydrase and lipid transport** superfamily protein |
| **Polyketide cyclase/dehydrase and lipid transport** superfamily protein | **Phospholipid/glycerol acyltransferase** family protein |
| **Polyketide cyclase/dehydrase and lipid transport** superfamily protein |  |
| **Lecithin: cholesterol acyltransferase 3** |  |
|  |  |
| **Sec23/Sec24 protein transport** family protein | **ATRER1C1**; **Encodes AtRER1C1, a Golgi membrane protein involved in returning the molecules that are exported from the endoplasmic reticulum (ER) to the Golgi apparatus back to the ER (a mechanism known as retrieval).** There are two Arabidopsis homologues of AtRERC1: AtRER1A and AtRER1B. |
| **Syntaxin/t-SNARE family protein** | **COP9-signalosome 5B** |
| **EXOCYST COMPLEX COMPONENT SEC3B, SEC3B** | **ENTH/VHS/GAT family protein; clathrin coat assembly, endocytosis.** |
| **MBOAT (membrane bound O-acyl transferase) family protein**  **Sec23/Sec24 protein transport** family protein | **SNARE-like superfamily protein; ER to Golgi vesicle-mediated transport.** |
| **Delta-adaptin** | **Secretory carrier membrane protein (SCAMP)** family protein |
| **Signal recognition particle-related / SRP-related** | **ATG8-INTERACTING PROTEIN 2**, ATI2; **Encodes an Atg8-interacting protein that is partially associated with the ER during favorable growth conditions and becomes mainly associated with a spherical compartment that dynamically moves along the ER network.** |
| **Rer1 family protein**; retrieval of some endoplasmic reticulum membrane proteins from the early Golgi compartment. | **VPS35 HOMOLOG A**, VPS35A, ZIG SUPPRESSOR 3, ZIP3; **Encodes a protein with similarity to yeast VPS35 which encodes a component of the retromer involved in retrograde endosomal transport.** |
| **Rer1 family protein**; retrieval of some endoplasmic reticulum membrane proteins from the early Golgi compartment. | **Vesicle transport V-snare 13** |
| **Signal recognition particle, SRP54 subunit protein; SRP-dependent co-translational protein targeting to membrane.** | **PRA1.F2, PRA7, PRENYLATED RAB ACCEPTOR 1.F2; PRA7; Involved in: vesicle-mediated transport.** |
|  | **SNARE-like** superfamily protein |
|  | **Rer1 family protein**; **retrieval of some endoplasmic reticulum membrane proteins from the early Golgi compartment.** |
|  | VIRB2-interacting protein 1 |
|  | **Reticulon family protein**: Reticulons are proteins that have been found predominantly associated with the endoplasmic reticulum in yeast and mammalian cells. While their functions are still poorly understood, **recent findings suggest that they participate in the shaping of the tubular endoplamic reticulum (ER).** |
|  | **Reticulon family protein** |
|  | **Reticulon family protein** |
|  | **Exocyst subunit exo70 family protein C1**; **exocytosis, vesicle docking involved in exocytosis. It is important for polar growth and plant development** |
|  |  |
|  | ORP3A, OSBP, **OXYSTEROL BINDING PROTEIN)-RELATED PROTEIN 3A,** UNE18, UNFERTILIZED EMBRYO SAC 18; **lipid transport, single fertilization, steroid metabolic process**. |
|  |  |
| **EamA-like transporter family** protein | **EamA-like transporter** family |
|  | **Nodulin MtN21 /EamA-like** transporter family protein; **UMAMIT22**, *USUALLY MULTIPLE ACIDS MOVE IN AND OUT TRANSPORTERS 22*; nodulin MtN21-like transporter family protein. |
|  | **Nodulin MtN21 /EamA-like transporter** family protein; UMAMIT41, *USUALLY MULTIPLE ACIDS MOVE IN AND OUT TRANSPORTERS 41* |
|  |  |
|  | **Aquaporin-like superfamily protein** |
|  |  |
|  | **Nuclear transport factor 2 (NTF2) family protein with RNA binding (RRM-RBD-RNP motifs) domain** |
|  | **Nuclear transport factor 2 (NTF2) family protein with RNA binding (RRM-RBD-RNP motifs) domain** |
|  |  |
|  | **Sucrose transporter 2** |
|  |  |
|  |  |

| **PROTEOLYSIS-PROTEIN DEGRADATION and others** | |
| --- | --- |
| **INDUCED** | **REPRESSED** |
| **FTSH protease 4** | **FTSH protease 12** |
| **FTSH extracellular protease** family |  |
|  |  |
| **Ubiquitin-conjugating enzyme 36** | **Ubiquitin-like superfamily** protein |
| **Ubiquitin-specific protease 18** | RHC1A, **RING-H2 FINGER C1A**; **Encodes a putative RING-H2 finger protein RHC1a. Proteasome-mediated ubiquitin-dependent protein catabolic process, protein polyubiquitination, protein ubiquitination involved in ubiquitin-dependent protein catabolic process.** |
| **Peptidase C78, ubiquitin fold modifier-specific peptidase 1/ 2** | **Ubiquitin-conjugating enzyme 25** |
| **Ubiquitin-protein ligase 4** | **Ubiquitin 7** |
| **Proteasome component (PCI) domain protein** | **Ubiquitin carboxyl-terminal hydrolase** family protein**; response to oxidative stress.** |
| **Ubiquitin-conjugating enzyme 33** | **Ubiquitin carboxyl-terminal hydrolase** family protein |
| **SUMO-activating enzyme 1A** | **Ovarian tumour**, otubain, **Ubiquitin thioesterase Otubain, Peptidase C65, otubain. protein deubiquitination** |
| **Regulatory particle non-ATPase 12B; proteasome assembly, proteasome-mediated ubiquitin-dependent protein catabolic process, proteolysis** | **MIF4G domain-containing protein** / **MA3 domain-containing protein**; **U-box E3 ubiquitin ligases.** |
| **DNA-binding protein with MIZ/SP-RING zinc finger**, PHD-finger and SAP domain; ATSIZ1, SIZ1**; Encodes a plant small ubiquitin-like modifier (SUMO) E3 ligase that is a focal controller of Pi starvation-dependent responses**. **Also required for SA and PAD4-mediated R gene signalling,** which in turn confers innate immunity in Arabidopsis. **Also involved in the regulation of plant growth, drought responses and freezing tolerance. This latter effect is most likely due to SIZ1 dependent ABI5 sumoylation.** **Regulates leaf cell division and expansion through salicylic acid accumulation.** |  |
| **Plant U-box 54; plant U-box 54 (PUB54);** **Functions in: ubiquitin-protein ligase activity;** Involved in: **response to stress, protein ubiquitination**. |  |
| **ASK9, SK9, SKP1-LIKE 9**; **one of Arabidopsis SKP1 homologues ; protein ubiquitination, regulation of mitotic cell cycle, ubiquitin-dependent protein catabolic process.** |  |
| **Ubiquitin domain-containing protein** |  |
| **Peptidase C12, ubiquitin carboxyl-terminal hydrolase 1** |  |
|  |  |
| **Subtilisin-like serine endopeptidase** family protein | **Subtilase family protein** |
| **Subtilisin-like serine endopeptidase** family protein |  |
| **Subtilase** family protein |  |
|  |  |
| **Cysteine-type peptidases; cysteine-type peptidases** | **Cysteine proteinases** superfamily protein |
| **PPPDE putative thiol peptidase** family protein | **Calpain-type cysteine protease** family |
|  | **Papain family cysteine protease** |
|  |  |
| **Eukaryotic aspartyl protease** family protein | **Eukaryotic aspartyl protease** family protein |
| **Eukaryotic aspartyl protease** family protein | **Eukaryotic aspartyl protease** family protein |
|  | **Eukaryotic aspartyl protease** family protein |
|  |  |
| **Metalloendopeptidases**; **zinc ion binding** | **Amidase family protein**; amidase activity**, carbon-nitrogen ligase activity, with glutamine as amido-N-donor.** |
| **APPR-1-P processing enzyme** family protein | Similar to **Ulp1 protease** family protein |
| Similar to **Ulp1 protease** family protein | **RHOMBOID-like protein 4 (RBL4);** Functions in: **serine-type endopeptidase activity** |
|  |  |
|  | **ATP-dependent caseinolytic (Clp) protease**/crotonase family protein |
|  | **Peptide-N4-(N-acetyl-beta-glucosaminyl) asparagine amidase A protein** |
|  | **ASPARAGINASE B1**; **Encodes an asparaginase that catalyzes the degradation of L-asparagine to L-aspartic acid and ammonia.** |
|  |  |
| SCPL8, **SERINE CARBOXYPEPTIDASE-LIKE 8**, SINAPOYLGLUCOSE 1, SNG1; sinapoylglucose: malate sinapoyltransferase. **Catalyzes the formation of sinapoylmalate from sinapoylglucose**. **Mutants accumulate excess sinapoylglucose. phenylpropanoid metabolic process**, proteolysis |  |
|  |  |
| APG7, ATAPG7, ATATG7, ATG7, **AUTOPHAGY 7, AUTOPHAGY-RELATED 7**, PEROXISOME UNUSUAL POSITIONING 4, PEUP4; Component of autophagy conjugation pathway. **Required for proper senescence. Contributes to plant basal immunity towards fungal infection. cellular response to nitrogen starvation, defense response to fungus**, late nucleophagy, leaf senescence, **macroautophagy, mitophagy**, piecemeal microautophagy of nucleus, protein catabolic process, protein lipidation, protein modification by small protein conjugation, protein transport. | *ARABIDOPSIS THALIANA NEXT TO BRCA1 GENE 1*, ATNBR1, NBR1, NEXT TO BRCA1 GENE 1; **Encodes NBR1, a selective autophagy substrate.** |
|  |  |
|  | **GDSL-like Lipase/ acylhydrolase** superfamily protein |
|  | **GDSL-motif lipase/hydrolase 6** |
|  |  |
| **HXXXD-type acyl-transferase** family protein | **HXXXD-type acyl-transferase** family protein |
| **HXXXD-type acyl-transferase** family protein | **HXXXD-type acyl-transferase** family protein |
|  | **S-adenosyl-L-methionine-dependent methyltransferases** |
|  |  |
|  |  |

| **REDOX** | |
| --- | --- |
| **INDUCED** | **REPRESSED** |
| **CYP705A4** | **Cytochrome P450, family 76, subfamily C, polypeptide 1** |
| **Cytochrome P450** superfamily protein |  |
| **Cytochrome P450, family 705, subfamily A, polypeptide 15** |  |
| **Cytochrome P450, family 71, subfamily A, polypeptide 18** |  |
|  |  |
| **2-oxoglutarate (2OG) and Fe(II)-dependent oxygenase** superfamily protein | **2-oxoglutarate (2OG) and Fe(II)-dependent oxygenase** superfamily protein |
| **2-oxoglutarate (2OG) and Fe(II)-dependent oxygenase** superfamily protein |  |
| **2-oxoglutarate (2OG) and Fe(II)-dependent oxygenase** superfamily protein |  |
|  |  |
| **Glutaredoxin** family protein |  |
| **Glutaredoxin** family protein |  |
|  |  |
| **Thioredoxin** superfamily protein | **Thioredoxin** superfamily protein |
| **Thioredoxin** superfamily protein | **Thioredoxin** superfamily protein |
|  | **Thioredoxin** superfamily protein |
|  | **Thioredoxin** superfamily protein |
|  | **Thioredoxin X** |
|  |  |
| **Metallo-hydrolase/oxidoreductase** superfamily protein |  |
| **Metallo-hydrolase/oxidoreductase** superfamily protein |  |
|  |  |
| **Peroxidase** superfamily protein | **Peroxidase** superfamily protein; **hydrogen peroxide catabolic process, oxidation-reduction process, response to oxidative stress.** |
|  | ***Arabidopsis thaliana* PEROXYGENASE 2** |
|  |  |
| **Metallothionein 3** | **Metallothionein 2B** |
|  |  |
| **Lactoylglutathione lyase family protein / glyoxalase I family protein** | **Glutathione S-transferase zeta 1** |
|  |  |
| **Ferretin 1**; Encodes a ferretin protein that is targeted to the **chloroplast.** Member of a Ferritin gene family. **Gene expression is induced in response to iron overload and by nitric oxide.** Expression of the gene is downregulated in the presence of paraquat, an inducer of photoxidative stress. cellular iron ion homeostasis, flower development, iron ion homeostasis, iron ion transport, leaf development**, oxidation-reduction process**, photosynthesis, response to bacterium, response to cold, response to cytokinin, **response to hydrogen peroxide**, response to iron ion, **response to reactive oxygen species**, response to zinc ion. | **Alternative oxidase 1C** |
|  |  |
| **Selenium binding; cell redox homeostasis** | **Methionine sulfoxide reductase B9** |
|  |  |
|  | SKS13**, SKU5 SIMILAR 13**; **oxidoreductase activity**, copper ion binding; Involved in: oxidation reduction. |
|  | **FAD/NAD(P)-binding oxidoreductase** family protein |
|  | **NAD(P)-linked oxidoreductase** superfamily protein |
|  | **Thiamin diphosphate-binding fold (THDP-binding)** superfamily protein; **oxidation-reduction process.** |
|  |  |
|  |  |
| **NHL domain-containing protein**; For example, the repeat is found in a variety of enzymes of the copper type II, **ascorbate dependent monooxygenase** family which catalyse the C-terminus alpha-amidation of biological peptides. | **Glucose-1-phosphate adenylyltransferase family protein**; GDP-mannose biosynthetic process; **plant ascorbic acid (vitamin C) biosynthesis.** |
|  |  |
|  |  |

| **CHAPERONES** | |
| --- | --- |
| **INDUCED** | **REPRESSED** |
| **Heat shock protein 70 (Hsp 70**) family protein |  |
| **Heat shock protein 90.1** |  |
| Similar to **heat shock protein binding** [Arabidopsis thaliana] |  |
|  |  |
| **Chaperone DnaJ-domain** superfamily protein | **Chaperone DnaJ-domain** superfamily protein |
| **DnaJ/Hsp40 cysteine-rich domain** superfamily protein | **Chaperone DnaJ-domain** superfamily protein |
| **DnaJ/Hsp40 cysteine-rich domain** superfamily protein | **Chaperone DnaJ-domain** superfamily protein |
| **Heat shock protein DnaJ with tetratricopeptide repeat** | **DnaJ heat shock family** protein |
|  | **DnaJ heat shock N-terminal domain-containing protein** |
|  |  |
|  | **Cyclophilin-like peptidyl-prolyl cis-trans isomerase** family protein; Functions in: peptidyl-prolyl cis-trans isomerase activity; Involved in: **protein folding** |
|  |  |
| **GroES-like** family protein | **GroES-like zinc-binding dehydrogenase** family protein |
|  |  |
|  | **Endomembrane protein 70** protein family |
|  |  |
|  | **Aha1 domain-containing** protein |
|  |  |
|  |  |

| **SIGNAL TRANSDUCTION** | |
| --- | --- |
| **INDUCED** | **REPRESSED** |
| **Protein kinase superfamily** protein | **Protein** kinase superfamily protein |
| **Protein kinase superfamily** protein | **Protein kinase superfamily** protein |
| **Protein kinase superfamily** protein | **Protein kinase superfamily** protein |
| **Protein kinase superfamily** protein |  |
| **Protein kinase superfamily** protein |  |
| **Protein kinase superfamily** protein |  |
| **Protein kinase superfamily** protein |  |
| **Protein kinase superfamily** protein |  |
|  |  |
|  |  |
| ANP2, MAPKKK2, **MITOGEN-ACTIVATED PROTEIN KINASE KINASE KINASES 2**, NP2, NPK1-RELATED PROTEIN KINASE 2; member of MEKK subfamily | **MAP kinase 5** |
|  | **MAP kinase 9** |
|  | **Mitogen-activated protein kinase homolog 2** |
|  |  |
|  |  |
| **3'-phosphoinositide-dependent protein kinase 1** | **Phosphatidylinositol 3- and 4-kinase family** protein |
| **N-acetylglucosaminylphosphatidylinositol de-N-acetylase** family | **inositol-pentakisphosphate 2-kinase** 1; ATIPK1, INOSITOL-PENTAKISPHOSPHATE 2-KINASE 1, IPK1, M40H3; **Encodes an inositol tetra-/pentaphosphate 2-kinase, involved in the biosynthesis of phytic acid,** a regulator of intracellular signaling, a highly abundant animal antinutrient, and a phosphate and mineral storage compound in plant seeds. **Is also required for growth and modulates phosphate homeostasis at the transcriptional level.** |
|  | **Inositol 1,3,4-trisphosphate 5/6-kinase** family protein |
|  | **Inositol-pentakisphosphate 2-kinase 1** |
|  |  |
| **Neutral/ alkaline non-lysosomal ceramidase**; cellular response to oxidative stress, cellular **sphingolipid homeostasis.** | **ENTH/VHS/GAT family protein**; Functions in**: phospholipid binding, clathrin binding, binding, phosphatidylinositol binding.** |
| **ENTH/ANTH/VHS s**uperfamily protein; Functions in: **phospholipid binding**; endocytosis | **Cyclopropane-fatty-acyl-phospholipid synthase** |
| **Carotenoid cleavage dioxygenase 1** |  |
|  |  |
| **Diacylglycerol kinase 4** | Thymidine kinase; Encodes a thymidine kinase that salvages DNA precursors. |
| **CBL-interacting protein kinase 25** | AGC (cAMP-dependent, cGMP-dependent and protein kinase C) kinase family protein |
| **Wall associated kinase 5** | guanylate kinase |
| **SNF1-related protein kinase 2.1** | Encodes a member of the MAKR (**MEMBRANE-ASSOCIATED KINASE REGULATOR**) gene family. MAKRs have putative kinase interacting motifs and membrane localization signals. |
| **Casein kinase II, alpha chain 2** | **Cyclin-dependent kinase-activating kinase assembly factor-related / CDK-activating kinase assembly factor-related.** |
| **Shaggy-like protein kinase 41** | **Kinase interacting (KIP1-like)** family protein |
|  | **SERINE/THREONINE/TYROSINE KINASE 8**, STY8; ACT-like protein tyrosine kinase family protein |
|  | **ACT-like protein tyrosine kinase** family protein |
|  |  |
| **Calcium-dependent protein kinase 1** |  |
| **Calcium-dependent protein kinase 31** |  |
| **Calcium-dependent protein kinase 13** |  |
|  |  |
| **Calcium-binding EF-hand** family protein | **Calcium-binding EF-hand** family protein |
| **EF hand calcium-binding** protein family | **Calcium-binding EF-hand** family protein |
|  |  |
|  | **Calmodulin 4** |
|  | **Calmodulin 2** |
|  |  |
| **Leucine-rich receptor-like protein kinase** family protein | **Receptor like protein 13** |
| **Leucine-rich receptor-like protein kinase** family protein | **Receptor like protein 21** |
| **Receptor like protein 40** | **Leucine-rich receptor-like protein kinase** family protein |
|  | **Receptor protein kinase-related** (TAIR:AT3G46270.1) |
|  |  |
| **Phosphatidic acid phosphatase (PAP2)** family protein | **Protein phosphatase 2C** family protein |
| **Protein phosphatase 2C** family protein | **Protein phosphatase 2C** family protein |
| **Protein phosphatase 2C** family protein | **Protein phosphatase 2A regulatory B** subunit family protein |
| **Protein phosphatase 2C** family protein | **Protein phosphatase 2C** family protein |
| **Protein phosphatase 2C** family protein | **Type one serine/threonine protein phosphatase 4** |
| **Dual specificity protein phosphatase** family protein |  |
| **Type one serine/threonine protein phosphatase 2** |  |
|  |  |
| **Myotubularin-like phosphatases II** superfamily; FUNCTIONS IN: phosphatase activity, **protein tyrosine phosphatase activity; intracellular signal transduction**, **lipid metabolic process.** |  |
|  |  |
| **RAB GTPase homolog 8C** | **GTP-binding** family protein |
| **RAB geranylgeranyl transferase alpha subunit 1** | AGD10, **MATERNAL EFFECT EMBRYO ARREST 28**, MEE28, ROOT AND POLLEN ARFGAP, RPA; A member of ARF GAP domain (AGD), A thaliana has 15 members, grouped into four classes. **GTPase activator activity**, metal ion binding, zinc ion binding |
|  | **GTP cyclohydrolase I** |
|  | RIC2, **ROP-INTERACTIVE CRIB MOTIF-CONTAINING PROTEIN 2**; required for their specific interaction with **GTP-bound Rop1 (plant-specific Rho GTPase)**. |
|  |  |
| **Concanavalin A-like lectin protein kinase** family protein | **Mannose-binding lectin** superfamily protein |
| **S-locus lectin protein kinase** family protein | **S-locus lectin protein kinase** family protein |
|  | **L-TYPE LECTIN RECEPTOR KINASE VI.1**, LECRK-VI.1; **Concanavalin A-like lectin** protein kinase family protein; |
|  |  |
|  | **Nucleotide-diphospho-sugar transferases** superfamily protein |
|  | **Nucleotide-diphospho-sugar transferase** family protein |
|  | **Nucleotide-diphospho-sugar transferases** superfamily protein |
|  |  |
|  | **Phosphate-responsive 1** family protein |
|  |  |
|  | **Dihydrosphingosine phosphate lyase** |
|  | **GENERAL REGULATORY FACTOR 12**, GF14 IOTA, GRF12; 14-3-3 protein GF14iota (grf12). |
|  | **Copine** (**Calcium-dependent phospholipid-binding** protein) family |
|  | **Calcineurin-like metallo-phosphoesterase** superfamily protein |
|  |  |
|  | ***Arabidopsis thaliana* METHYL ESTERASE 15**, ATMES15, MES15, METHYL ESTERASE 15, RHS9, ROOT HAIR SPECIFIC 9; **Encodes a protein predicted to act as a carboxylesterase.** It has similarity to the **SABP2 methyl salicylate esterase from tobacco** but no enzymatic activity has been identified for this protein. |
|  |  |
|  |  |

| **DUF PROTEINS** | |
| --- | --- |
| **INDUCED** | **REPRESSED** |
| Plant protein of unknown function (DUF863) | Family of unknown function (DUF577) |
| Protein of unknown function (DUF1313) | Protein of unknown function, DUF584 |
| Protein of unknown function (DUF1644) | DOMAIN OF UNKNOWN FUNCTION 724 4 |
| Protein of unknown function (DUF1278) | Domain of unknown function (DUF313) |
| Protein of unknown function, DUF584 | Protein of unknown function (DUF295) |
| Protein of unknown function (DUF1677) | DUF679 domain membrane protein 2 |
| Protein of unknown function (DUF1216) | Protein of unknown function (DUF295) |
| Protein of unknown function (DUF581) | Protein of unknown function (DUF2930) |
| Protein of unknown function (DUF640) | Domain of unknown function (DUF543) |
| Protein of unknown function (DUF1664) | Eukaryotic protein of unknown function (DUF914) |
| Protein of unknown function (DUF581) | Protein of unknown function (DUF295) |
| Protein of unknown function, DUF617 | Protein of unknown function (DUF3133) |
| Domain of unknown function (DUF1985) | Protein of unknown function (DUF784) |
| Domain of unknown function (DUF313) | Uncharacterized protein family (UPF0497) |
| Protein of unknown function (DUF581) | Protein of unknown function (DUF3537) |
| Protein of unknown function DUF106, transmembrane | Plant protein 1589 of unknown function |
|  | Protein of unknown function (DUF740) |
|  | Protein of unknown function (DUF630 and DUF632) |
|  | Plant protein of unknown function (DUF828) |
|  | Protein of unknown function (DUF833) |
|  | Protein of unknown function (DUF626) |
|  | Kinase-related protein of unknown function (DUF1296) |
|  | Protein of unknown function, DUF599 |
|  | Arabidopsis protein of unknown function (DUF241) |
|  | Protein of unknown function, DUF617 |
|  | Protein of unknown function (DUF1184) |
|  | Protein of unknown function (DUF1278) |
|  | Family of unknown function (DUF566) |
|  | Protein of unknown function (DUF1223) |
|  | Protein of unknown function (DUF677) |
|  | Protein with domains of unknown function (DUF627 and DUF629) |
|  | Protein of unknown function (DUF498/DUF598) |
|  | Plant protein of unknown function (DUF247) |
|  |  |
|  |  |
|  |  |

| **CHLOROPLAST-PHOTOSYNTHESIS-ENERGY-PRIMARY/SECONDARY METABOLISM** | |
| --- | --- |
| **INDUCED** | **REPRESSED** |
| **Photosystem I subunit D-1** | **Photosystem II light harvesting complex gene 2.1** |
| **Chlorophyll A/B binding protein 3** | **PGR5-LIKE A**, Encodes PGRL1A, a transmembrane protein present in thylakoids. PGRL1A has a highly homologous isoform PGRL1B encoded by At4g11960. Plants lacking PGRL1 show **perturbation of cyclic electron flow,** similar to PGR5-deficient plants. PGRL1 and PGR5 interact physically and associate with PSI (photosystem I). |
| **Phytochelatin synthase 1 (PCS1)** | **PGR5-LIKE A**; Encodes PGRL1A, a transmembrane protein present in thylakoids. PGRL1A has a highly homologous isoform PGRL1B encoded by At4g11960. **Plants lacking PGRL1 show perturbation of cyclic electron flow,** similar to PGR5-deficient plants. |
| SE2, ATASE2, ATPURF2**, CHLOROPLAST IMPORT APPARATUS 1**, CIA1, **DIFFERENTIAL DEVELOPMENT OF VASCULAR ASSOCIATED CELLS, DOV1**, **GLN PHOSPHORIBOSYL PYROPHOSPHATE AMIDOTRANSFERASE** 2; Encodes glutamine 5-phosphoribosylpyrophosphate amidotransferase. **Mutants are deficient in leaf, but not cotyledon, plastid and palisade cell development. Mutants exhibit defective chloroplast development under non-low light, suggesting that the defect in chloroplast development is caused by photo-oxidative damage.** Plays role in differential development of vascular-associated cells. Demonstrates a cell-specific difference in chloroplast development. Mutant leaves are highly reticulate with a green vascular pattern. **'de novo' IMP biosynthetic process, chloroplast organization, glutamine metabolic process, leaf morphogenesis.** | **Pyridine nucleotide-disulphide oxidoreductase** family protein; Encodes for a multifunctional protein with **geranylgeranyl reductase activity shown to catalyze the reduction of prenylated geranylgeranyl-chlorophyll a to phytyl-chlorophyll a (chlorophyll a) and free geranylgeranyl pyrophosphate to phytyl pyrophosphate**. Vitamin E biosynthetic process |
|  | **Light harvesting complex photosystem II** |
|  |  |
| **GENES NECESSARY FOR THE ACHIEVEMENT OF RUBISCO ACCUMULATION 5**, NARA5; Encodes a **phosphofructokinase B-type carbohydrate kinase family protein, NARA5. Regulates photosynthetic gene expression.** |  |
| **Plastid transcriptionally active 17** | **PLASTID TRANSCRIPTIONALLY ACTIVE 14**, PTAC14, TAC14; plastid transcriptionally active 14 (PTAC14**): Rubisco methyltransferase family protein** (TAIR:AT1G24610.1) |
|  | **Cobalamin biosynthesis CobW-like** protein; **plastid transcriptionally active 17.** |
|  |  |
| **H(+)-ATPase 1** | **AFG1-like ATPase** family protein |
| **AAA-type ATPase** family protein | **Gamma subunit of Mt ATP synthase** |
|  | **AAA-type ATPase** family protein |
|  | **ATPase, F0/V0 complex, subunit C protein**; Involved in: **ATP synthesis** coupled proton transport. |
|  | **ATPase, V0 complex, subunit E** |
|  |  |
| **MITOCHONDRIAL PYRUVATE DEHYDROGENASE SUBUNIT 2-2**, MTE2-2; Encodes a subunit of the mitochondrial pyruvate dehydrogenase complex. | AT-E1 ALPHA, E1 ALPHA**, PYRUVATE DEHYDROGENASE COMPLEX E1** ALPHA subunit. |
| **Acyl-CoA oxidases; oxidoreductases**, acting on the CH-CH group of donors; FAD binding; oxidoreductases;acyl-CoA oxidases; **fatty acid oxidation.** | **PfkB-like carbohydrate kinase** family protein; **D-ribose metabolic process,** **acetate fermentation**, sucrose biosynthetic process, **sucrose catabolic process**, using beta-fructofuranosidase. |
| **Phosphofructokinase 4** | **Pyruvate kinase** family protein |
| **Citrate synthase** 2; Encodes a peroxisomal citrate synthase that is expressed throughout seedling and shoot development. **Fatty acid beta-oxidation**, tricarboxylic acid cycle. | Surfeit locus 1 cytochrome c oxidase biogenesis protein; respiratory chain complex IV assembly. |
| **Phosphofructokinase 1** | **Ubiquinol-cytochrome C reductase hinge protein**; Functions in: ubiquinol-cytochrome-c reductase activity; Involved in: **mitochondrial electron transport**, **ubiquinol to cytochrome.** |
| ATIVD, **ISOVALERYL-COA-DEHYDROGENASE**, IVD, IVDH; branched-chain amino acid catabolic process, **fatty acid beta-oxidation using acyl-CoA dehydrogenase**, leucine catabolic process, lipid homeostasis. | **2Fe-2S ferredoxin-like** superfamily protein; **electron transport.** |
|  | **Phosphoglycerate mutase** family protein; **glycolysis.** |
|  |  |
|  | **GRIM-19 protein**; Functions in: molecular_function unknown; Involved in: **photorespiration** |
|  | ATGWD2, GWD3, **PHOSPHOGLUCAN, WATER DIKINASE**, PWD, Pyruvate phosphate dikinase, PEP/pyruvate binding domain. |
|  | **Glycine decarboxylase P-protein 1** |
|  |  |
|  | **Plant stearoyl-acyl-carrier-protein desaturase** family protein; Functions in: acyl-[acyl-carrier-protein] desaturase activity, oxidoreductase activity, transition metal ion binding; Involved in: oxidation reduction, **fatty acid metabolic process**, fatty acid biosynthetic process. |
|  |  |
|  | **Haem oxygenase-like**, multi-helical; **thiamine biosynthetic process, thiamine diphosphate biosynthetic** process. |
|  |  |
|  | **Triacylglycerol lipase-like 1** |
|  | **POLYPRENOL REDUCTASE 3**, PPRD3; 3-oxo-5-alpha-steroid 4-dehydrogenase family protein |
|  | **3-oxo-5-alpha-steroid 4-dehydrogenase** family protein |
|  | **FARNESYL DIPHOSPHATE SYNTHASE 1**, FPS1; Encodes a protein with farnesyl diphosphate synthase activity; cholesterol biosynthetic process, **farnesyl diphosphate biosynthetic process, geranyl diphosphate biosynthetic process.** |
|  |  |
|  |  |

| **DNA REPAIR-HISTONE MODIFICATIONS-RNA MODIFICATIONS-PROTEIN SYNTHESIS** | |
| --- | --- |
| **INDUCED** | **REPRESSED** |
| **RNA-binding (RRM/RBD/RNP motifs**) family protein | **RNA-binding (RRM/RBD/RNP motifs**) family protein |
| **RNA-binding (RRM/RBD/RNP motifs)** family protein | **RNA-binding (RRM/RBD/RNP motifs)** family protein |
| **RNA-binding** protein | **RNA-binding (RRM/RBD/RNP motifs**) family protein |
|  | **RNA-binding protein 1** |
|  | **TERMINAL EAR1-like (TEL) genes** encode **putative RNA-binding proteins** only found in land plants. Previous studies suggested that they may regulate tissue and organ initiation in Poaceae. |
|  |  |
| **HNH endonuclease domain-containing** protein | **Endonuclease 2** |
| **Polynucleotidyl transferase, ribonuclease H-like** superfamily protein | **RNAse E/G-like** |
| **Polynucleotidyl transferase, ribonuclease H-like** superfamily protein | **Ribonuclease II/R** family protein |
| SBP (**S-ribonuclease binding** protein) family protein | **Polynucleotidyl transferase, ribonuclease H-like** superfamily protein |
| **HNH endonuclease domain-containing** protein | **RNAse II-like 1** |
|  | **Endonuclease III 2** |
|  |  |
| **Ribosome associated membrane protein RAMP4** | **Ribosomal protein L18ae** family |
| **Ribosomal protein S5 domain 2-like** superfamily protein | **Ribosomal protein L6** family protein |
| **Ribosomal protein L36e** family protein | **Ribosomal protein L7Ae/L30e/S12e/Gadd45** family protein |
| **Ribosomal protein L11** family protein | **Ribosomal protein L6** family protein |
|  | **60S ribosomal protein L18A-1** |
|  | **Ribosomal protein L34e** superfamily protein |
|  | **Ribosomal protein S11-beta** |
|  |  |
| **Nuclear poly(a) polymerase** | **Splicing factor**, putative |
| **FUS3-COMPLEMENTING GENE 2**; a LAMMER-type protein kinase that co-precipitates with serine/arginine-rich (SR) proteins in vitro, interaction modulated by phosphorylation of the proteins. **mRNA processing**. | APUM7, PUM7, **PUMILIO 7;** Encodes a member of the Arabidopsis Pumilio (APUM) proteins containing PUF domain (eight repeats of approximately 36 amino acids each). PUF proteins **regulate both mRNA stability and translation** through sequence-specific binding to the 3' UTR of target mRNA transcripts |
| SWAP (**Suppressor-of-White-APricot**)/surp domain-containing; **RNA processing; Pre-mRNA splicing factor** PRP21 like protein. | **Pre-mRNA-processing protein 40A** |
| CID5, **CTC-INTERACTING DOMAIN 5**, INCREASED POLYPLOIDY LEVEL IN DARKNESS 1, IPD1; Interacts with PAB (**poly A binding protein)** in yeast two hybrid experiments. Contains PAM2 motif, a PABC interacting domain. | AT-RSZ22, ATRSZ22, **RS-CONTAINING ZINC FINGER PROTEIN 22**, RSZ22, RSZP22, SERINE/ARGININE-RICH 22, SRZ-22, SRZ22.  Encodes a Serine/arginine-rich (SR) protein RSZp22. **SR proteins are splicing regulators** that share a modular structure consisting of one or two N-terminal RNA recognition motif domains and a C-terminal RS-rich domain. |
|  | **ARGININE/SERINE-RICH ZINC KNUCKLE-CONTAINING PROTEIN 33**; **RNA splicing, mRNA splicing, via spliceosome**, spliceosomal complex assembly. |
|  | **Poly(ADP-ribose) polymerase** |
|  | Polynucleotide adenylyltransferase family protein; **RNA processing** |
|  | APUM2, PUM2, **PUMILIO 2** |
|  |  |
| **Gag-Pol-related retrotransposon** family protein | **Transposable_element**_gene |
|  | **Transposable_element**_gene |
|  | **Putative harbinger transposase-derived nuclease** (InterPro:IPR006912) |
|  |  |
| **Histone deacetylase-related** / HD-related | **Histone-lysine N-methyltransferase ASHH3** |
| **Histone H4** | **Histone superfamily** protein |
| **Histone H2A protein 9** |  |
|  |  |
| **Transcription initiation factor TFIIE**, beta subunit | **Translation initiation factor SUI1** family protein |
| **tRNA synthetase class I** (I, L, M and V) family protein | **Eukaryotic translation initiation factor 3 subunit 7** (eIF-3) |
| Fibrillarin family protein; **tRNA processing**, **rRNA processing** | **Glutamyl-tRNA reductase** family protein |
| **MA3 domain-containing protein**; Initiation factor eIF-4 gamma | **Transcription elongation factor (TFIIS)** family protein |
| BAH domain ;TFIIS helical bundle-like domain; Functions in: **transcription regulator activity**. | **Translation elongation factor Ts (EF-Ts),** putative |
| **Translation initiation factor 1A** (eIF-1A) (**Nucleic acid-binding, OB-fold-like** protein) | **Transcription elongation factor (TFIIS)** family protein |
| Aspartate/glutamate/uridylate kinase family protein;  **regulation of transcription,** DNA-templated |  |
|  |  |
| **DNA-directed RNA polymerase II**-related | **Amidophosphoribosyltransferas**e (ATase: EC 2.4.2.14) is a key enzyme in the pathway of **purine nucleotide biosynthesis.** |
| homolog of human **DNA ligase IV-binding protein XRCC4** |  |
| PYRD, **PYRIMIDINE D**; dihydroorotate dehydrogenase, **catalyses fourth step of pyrimidine biosynthesis.** |  |
|  |  |
| **ARGONAUTE 3** |  |
| **Small RNA degrading nuclease 3** |  |
| Encodes **JASON***. jason* mutant produces diploid male gametes leading to triploid progeny. Diploid gametes in the jason mutant are generated by a defect in male meiosis II. |  |
|  |  |
| **Structural maintenance of chromosomes (SMC)** family protein |  |
| **Regulator of chromosome condensation (RCC1)** family protein |  |
| **Regulator of chromosome condensation (RCC1)** family protein |  |
| ADA2B, **HOMOLOG OF YEAST ADA2 2B**, PROPORZ1, PRZ1; Transcriptional co-activator. Essential for the developmental switch from cell proliferation to cell differentiation in response to variations in auxin and cytokinin concentrations. **Chromatin remodeling, cold acclimation, positive regulation of histone acetylation.** | **SU (VAR)3-9 HOMOLOG 1,** SUVH1; Encodes a SU(VAR)3-9 homolog, a SET domain protein. **Known SET domain proteins are involved in epigenetic control of gene expression and act as histone methyltransferases.** There are 10 SUVH genes in Arabidopsis and members of this subfamily of the SET proteins have an additional conserved SRA domain. |
| GAS41, **GLIOMAS 41, HOMOLOG OF YEAST YAF9 A**, TAF14B, TBP-ASSOCIATED FACTOR 14B, YAF9A; The GSA41 human homolog is expressed in nuclei and binds NuMA, a component of the nuclear matrix in interphase nuclei. **It negatively regulates flowering by controlling the H4 acetylation levels in the FLC and FT chromatin**. Flower development, **histone H4-K5 acetylation, regulation of histone H4 acetylation**, regulation of photoperiodism, flowering, regulation of timing of transition from vegetative to reproductive phase**.** |  |
| ATSGO2, SGO2**, SHUGOSHIN 2**; Encodes a protein that protects meiotic centromere cohesion. Maintenance of meiotic sister chromatid cohesion, meiotic chromosome segregation. |  |
|  |  |
| Putative mitochondrial **RNA helicase 1** | **Histidine kinase-, DNA gyrase B-, and HSP90-like ATPase** family protein |
| **Gamma response gene 1**; Induced in response to **ionizing radiation**, shows basal expression in mitotically active cells and high expression in endoreduplicating cells. **May be involved in DNA damage-induced growth arrest.** Protein sequence contains a PEST destruction box. DNA damage checkpoint, DNA repair, cellular response to DNA damage stimulus, gamete generation, meiotic DNA double-strand break processing. | **DNA photolyases** |
| **HOMOLOG OF RAD51 D**, RAD51D; DNA repair, meiotic DNA recombinase assembly, mitotic recombination, **positive regulation of defense response to bacterium**, reciprocal meiotic recombination, **response to ionizing radiation**, somatic cell DNA recombination, strand invasion. | **DNA photolyase** family protein |
| **HOMOLOG OF X-RAY REPAIR CROSS COMPLEMENTING 4**, XRCC4; DNA recombination**, double-strand break repair.** | **Rad23 UV excision repair** protein family |
|  | **Helicase protein with RING/U-box domain.** |
|  | **Serpin 2 (SRP2);** Functions in: serine-type endopeptidase inhibitor activity; Involved in: **DNA repair, response to DNA damage stimulus.** |
|  | **MMZ1/UEV1A** encodes a protein that may play a role in **DNA damage responses and error-free post-replicative DNA repair** by participating in lysine-63-based polyubiquitination reactions. |
|  | SMR (**Small MutS Related**) domain-containing protein;  MutS family proteins are widely distributed in almost all organisms from bacteria to human. They play central roles in various DNA transactions such as **DNA mismatch repair and recombinational events.** |
|  | **Histidine kinase-, DNA gyrase B-, and HSP90-like ATPase** family protein |
|  | **MMS ZWEI homologue** 1; **postreplication repair**, protein K63-linked ubiquitination. |
|  |  |
| Homolog of **RAD51 D** | **3XHIGH MOBILITY GROUP-BOX2**, 3XHMG-BOX2 |
| **Nucleic acid-binding, OB-fold-like protein** | **Phosphoribosyl pyrophosphate (PRPP) synthase 2** |
|  |  |
|  |  |

| **OTHER** | |
| --- | --- |
| **INDUCED** | **REPRESSED** |
| **Cysteine/Histidine-rich C1** domain family protein | **Cysteine/Histidine-rich C1** domain family protein |
| **Cysteine/Histidine-rich C1** domain family protein |  |
| **Cysteine/Histidine-rich C1** domain family protein |  |
|  |  |
| **NAD(P)-binding Rossmann-fold** superfamily protein | **NAD(P)-binding Rossmann-fold** superfamily protein |
| **NAD(P)-binding Rossmann-fold** superfamily protein | **NAD(P)-binding Rossmann-fold** superfamily protein |
| **NAD(P)-binding Rossmann-fold** superfamily protein | **NAD(P)-binding Rossmann-fold** superfamily protein |
|  | **NAD(P)-binding Rossmann-fold** superfamily protein |
|  |  |
|  |  |
| **F-box** and associated interaction domains-containing protein | **F-box** family protein |
| **F-box** family protein | **F-box** and associated interaction domains-containing protein |
|  | **F-box** family protein with a domain of unknown function (**DUF295**) |
|  | F-box and associated interaction domains-containing protein |
|  | **F-box domain, cyclin-like** (InterPro:IPR001810) |
|  | **F-box** family protein |
|  | **F-box** and associated interaction domains-containing protein |
|  | **F-box** and associated interaction domains-containing protein |
|  |  |
| **F-box/RNI-like** superfamily protein | **F-box/RNI-like/FBD-like** domains-containing protein |
| **F-box/RNI-like** superfamily protein | **F-box/RNI-like** superfamily protein |
|  | **F-box/RNI-like/FBD-like** domains-containing protein |
|  |  |
| **RING/U-box** superfamily protein | **RING/U-box** superfamily protein |
| **RING/U-box** superfamily protein | **RING/U-box** superfamily protein |
| **RING/U-box** superfamily protein |  |
| **RING/U-box** superfamily protein |  |
| **RING/U-box** superfamily protein |  |
| **RING/U-box** superfamily protein |  |
| **RING/U-box** superfamily protein |  |
| **RING/U-box** superfamily protein |  |
|  |  |
| **RING-H2 finger C2A** | **RING-H2 finger A1A** |
|  | **CHY-type/CTCHY-type/RING-type Zinc finger** protein |
|  | **RING/FYVE/PHD zinc finger** superfamily protein |
|  |  |
| **PRP38** family protein | **SMAD/FHA** domain-containing protein |
|  |  |
|  |  |
| **S-adenosyl-L-methionine-dependent methyltransferases** superfamily protein | **S-adenosyl-L-methionine-dependent methyltransferases** superfamily protein |
| **S-adenosyl-L-methionine-dependent methyltransferases** superfamily protein | **S-adenosyl-L-methionine-dependent methyltransferases** superfamily protein |
|  | **S-adenosyl-L-methionine-dependent methyltransferases** superfamily protein |
|  | **S-adenosyl-L-methionine-dependent methyltransferases** superfamily protein |
|  |  |
| **Galactose oxidase/kelch repeat superfamily protein** | **Galactose oxidase/kelch repeat** superfamily protein |
| **Galactose oxidase/kelch repeat** superfamily protein |  |
|  |  |
| **ARM repeat** superfamily protein | **ARM repeat** superfamily protein |
| **ARM repeat** superfamily protein | **ARM repeat** superfamily protein |
|  | **ARM repeat** superfamily protein |
|  | **ARM repeat** superfamily protein |
|  | **ARM repeat** superfamily protein |
|  |  |
| **Alpha/beta-Hydrolases** superfamily protein | **Alpha/beta-Hydrolases** superfamily protein |
| **Alpha/beta-Hydrolases** superfamily protein | **Alpha/beta-Hydrolases** superfamily protein |
| **Alpha/beta-Hydrolases** superfamily protein | **Alpha/beta-Hydrolases** superfamily protein |
|  |  |
| **Pentatricopeptide repeat (PPR**) superfamily protein | **Pentatricopeptide repeat (PPR)** superfamily protein |
| **Pentatricopeptide repeat (PPR)** superfamily protein | **Pentatricopeptide repeat (PPR)** superfamily protein |
| **Pentatricopeptide repeat (PPR)** superfamily protein |  |
|  |  |
| **P-loop containing nucleoside triphosphate hydrolases** superfamily protein | **P-loop containing nucleoside triphosphate hydrolases** superfamily protein |
|  | **P-loop containing nucleoside triphosphate hydrolases** superfamily protein |
|  | **P-loop containing nucleoside triphosphate hydrolases** superfamily protein |
|  | **P-loop containing nucleoside triphosphate hydrolases** superfamily protein |
|  |  |
| **Tetratricopeptide repeat (TPR)-like** superfamily protein |  |
| **Tetratricopeptide repeat (TPR)-like** superfamily protein |  |
| **Tetratricopeptide repeat (TPR)-like** superfamily protein |  |
| **Tetratricopeptide repeat (TPR)-like** superfamily protein |  |
| **Tetratricopeptide repeat (TPR)-like** superfamily protein |  |
| **Tetratricopeptide repeat (TPR)-like** superfamily protein |  |
| **Tetratricopeptide repeat (TPR)-like** superfamily protein |  |
|  |  |
| **Transducin family protein / WD-40 repeat** family protein | **Transducin family protein / WD-40 repeat** family protein |
| **Transducin/WD40 repeat-like** superfamily protein | **Transducin family protein / WD-40 repeat** family protein |
| **Transducin/WD40 repeat-like** superfamily protein |  |
| **Transducin/WD40 repeat-like** superfamily protein |  |
| **Transducin family protein / WD-40 repeat** family protein |  |
| **Transducin/WD40 repeat-like** superfamily protein; **homolog of yeast autophagy 18 (ATG18) B.** |  |
|  |  |
| **BTB/POZ domain-containing** protein |  |
| **BTB/POZ domain-containing** protein |  |
|  |  |
|  |  |
