# Supplementary material for: Overexpression of Grain Amaranth (Amaranthus hypochondriacus) AhERF or AhDOF Transcription Factors in Arabidopsis thaliana Increases Water Deficit- and Salt-Stress Tolerance, Respectively, via Contrasting Stress-Amelioration Mechanisms
Source: PLoS One. 2016 Oct 17;11(10):e0164280. doi: 10.1371/journal.pone.0164280 (PMC5066980; doi:10.1371/journal.pone.0164280)
Supplement: S5 Table — (DOCX) [file pone.0164280.s011.docx]

**S5 Table. List of genes with altered expression detected in transgenic *AhDOF-AI* overexpressing Arabidopsis plants under salt-stress conditions.**

| **CELL WALL COMPONENTS MODIFICATION LIGNIFICATION CHO METABOLISM** | |
| --- | --- |
| **INDUCED** | **REPRESSED** |
| **Glycosyl hydrolase** superfamily protein; Glycoside hydrolase, family 18, catalytic domain (InterPro:IPR001223), Chitinase II | **Glycosyl hydrolase 9B5** |
| **Glycosyl hydrolase family 10** protein; carbohydrate metabolic process | **Glycosyl hydrolase** superfamily protein; family 5 (CELLULASE) |
| **Cellulase (glycosyl hydrolase family 5)** protein |  |
| **Glycosyl hydrolase** superfamily protein |  |
|  |  |
|  | **Hydroxyproline-rich** glycoprotein family protein |
|  | **Hydroxyproline-rich** glycoprotein family protein |
|  | **Hydroxyproline-rich** glycoprotein family protein |
|  | **Hydroxyproline-rich** glycoprotein family protein |
|  | **Hydroxyproline-rich** glycoprotein family protein |
|  |  |
| **UDP-glucosyl transferase 73C6** | **UDP-Glycosyltransferase** superfamily protein |
| **UDP-Glycosyltransferase** superfamily protein | **UDP-Glycosyltransferase** superfamily protein |
| **UDP-glucosyl transferase 76E12** | **UDP-glucosyl transferase 74B1** |
|  | **UDP-Glycosyltransferase** superfamily protein |
|  | **UDP-glucosyl transferase 73C7** |
|  | **UDP-Glycosyltransferase** superfamily protein |
|  |  |
| **Bifunctional inhibitor/lipid-transfer protein/seed storage 2S albumin** superfamily protein | **Bifunctional inhibitor/lipid-transfer protein/seed storage 2S albumin** superfamily protein |
| **Bifunctional inhibitor/lipid-transfer protein/seed storage 2S albumin** superfamily protein | **Bifunctional inhibitor/lipid-transfer protein/seed storage 2S albumin** superfamily protein |
| **SEED STORAGE ALBUMIN 5**, SESA5; **Bifunctional inhibitor/plant lipid transfer protein/seed storage** (InterPro:IPR016140), Napin/ Bra allergen (InterPro:IPR000617), **Plant lipid transfer protein/seed storage/trypsin-alpha amylase inhibitor** (InterPro:IPR003612). |  |
| **Bifunctional inhibitor/lipid-transfer protein/seed storage 2S albumin** superfamily protein |  |
|  |  |
| **Galactosyl transferase** GMA12/MNN10 family protein; MUCI10, MUCILAGE-RELATED10; Encodes MUCI10, a galactomannan-1,6-galactosyltransferase. MUCI10 likely decorates glucomannan, synthesized by CSLA2, with galactose residues in vivo. The degree of galactosylation is essential for the synthesis of the GGM backbone, **the structure of cellulose, mucilage density, as well as the adherence of pectin.** | **Cellulose synthase-like D5** |
|  | **TRICHOME BIREFRINGENCE-LIKE 38**; involved in the synthesis and deposition of **secondary wall cellulose, presumably by influencing the esterification state of pectic polymers** |
|  | **Cellulose synthase-like A3** |
|  | **COBRA-like protein 1 precursor**; cell growth, cellulose microfibril organization |
|  |  |
| **Plant invertase/pectin methylesterase inhibitor** superfamily | **Plant invertase/pectin methylesterase inhibitor** superfamily |
| **Plant invertase/pectin methylesterase inhibitor** superfamily | **Plant invertase/pectin methylesterase inhibitor** superfamily |
| **Plant invertase/pectin methylesterase inhibitor** superfamily | **Plant invertase/pectin methylesterase inhibitor** superfamily |
|  | **6-FRUCTAN EXOHYDROLASE**, ATCWINV3, ATFRUCT5, BETA-FRUCTOFURANOSIDASE 5, FRUCT5; Encodes a protein with fructan exohydrolase (FEH) activity acting on levan-type fructans (6-FEH, levanase). **The enzyme does not have invertase activity.** |
|  | **Cytosolic invertase 2** |
|  |  |
| **Pectin lyase-like** superfamily protein | **Pectin lyase-like** superfamily protein |
| **Pectin lyase-like** superfamily protein | **Pectin lyase-like** superfamily protein |
| **Rhamnogalacturonate lyase** family protein | **Pectin lyase-like** superfamily protein |
| **Pectin lyase-like** superfamily protein | **Pectin lyase-like** superfamily protein |
|  | **Pectin lyase-like** superfamily protein |
|  | **Pectin lyase-like** superfamily protein |
|  | **Pectin lyase-like** superfamily protein |
|  |  |
|  | **Nucleotide-rhamnose synthase/epimerase-reductase** |
|  | **Pectinacetylesterase** family protein |
|  | **Exostosin** family protein **ARABINAN DEFICIENT 1,** ARAD1; Putative glycosyltransferase, similar to other CAZy Family 47 proteins. **Cell wall organization, pectin biosynthetic process** |
|  |  |
| **GDSL-like Lipase/Acylhydrolase** superfamily protein | **GDSL-like Lipase/Acylhydrolase** superfamily protein |
|  | **GDSL-like Lipase/Acylhydrolase** superfamily protein |
|  | **GDSL-like Lipase/Acylhydrolase** superfamily protein |
|  | **GDSL-like Lipase/Acylhydrolase** superfamily protein |
|  | **GDSL-like Lipase/Acylhydrolase** superfamily protein |
|  | **GDSL-like Lipase/Acylhydrolase** superfamily protein |
|  | **GDSL-like Lipase/Acylhydrolase** superfamily protein |
|  |  |
| **FAD-binding Berberine** family protein | **FAD-binding Berberine** family protein |
|  | **FAD-binding Berberine** family protein |
|  | **FAD-binding Berberine** family protein |
|  |  |
|  | **CAFFEOYL SHIKIMATE ESTERASE**, CSE, LYSOPHOSPHOLIPASE 2, LYSOPL2; Encodes caffeoyl shikimate esterase and is **involved in lignin biosynthesis.** CSE converts caffeoyl shikimate to caffiate. Loss of function mutations have reduced lignin content and collapsed vessel elements. It is also reported to function as a lysophospholipase 2 (LysoPL2) involved in tolerance to cadmium-induced oxidative stress. Binds Acyl-CoA-binding protein 2 (ACBP2). |
|  | **Hydroxycinnamoyl-CoA shikimate/quinate hydroxycinnamoyl transferase** |
|  |  |
| **Myosin heavy chain-related** |  |
| **Myosin heavy chain-related** protein |  |
| **Myosin heavy chain-related** |  |
| **Myosin heavy chain-related** protein |  |
| **Myosin heavy chain-related** |  |
| **Myosin heavy chain-related** |  |
|  |  |
| **Kinase interacting (KIP1-like) family protein NET4A**, NETWORKED 4A; **Encodes a member of the NET superfamily of proteins that potentially couples different membranes to the actin cytoskeleton in plant cells**. It co-localizes with filamentous actin and is localized to the tonoplast membrane**. It is expressed in the epidermis of the root meristem and the early expansion zone. colocalizes with actin filament.** | **Actin-related protein 4** |
| **Actin-related protein 9** | **Encodes profiling 2**, a low-molecular weight, **actin monomer-binding protein that regulates the organization of actin cytoskeleton.** Expressed in vegetative organs. Actin polymerization or depolymerization, cytoskeleton organization, inflorescence development, lateral root development, leaf development, sequestering of actin monomers |
| **Actin family** protein | **Kinesin motor** family protein |
| **Tubulin beta-9 chain** |  |
| **Dynamin related** protein |  |
|  |  |
| **Glycine-rich protein** | **Glycine-rich protein 20** |
|  | **Gycine-rich** protein |
|  | **Glycine-rich** protein |
|  |  |
| **Proline-rich** family protein | **Proline-rich extensin-like** family protein |
|  | **Extensin 3** |
|  | **Pollen Ole e 1 allergen and extensin** family protein |
|  |  |
| **Arabinogalactan protein 41** | **Arabinogalactan protein 17** |
| **Arabinogalactan protein 14** | **Fasciclin-like arabinogalactan** family protein |
|  | **Syntaxin of plants 31** |
|  |  |
|  | **Nucleotide-diphospho-sugar transferases** superfamily protein |
|  | **Nucleotide-diphospho-sugar transferases** superfamily protein |
|  | **Nucleotide-diphospho-sugar transferases** superfamily protein |
|  | **Nucleotide-diphospho-sugar transferase** family protein |
|  |  |
| **Expansin A25**, ATEXP25, ATEXPA25, ATHEXP ALPHA 1.18, EXP25, EXPA25, EXPANSIN 25, EXPANSIN A25, **member of Alpha-Expansin Gene Family plant-type** **cell wall loosening**, plant-type cell wall modification involved in multidimensional cell growth, unidimensional cell growth. |  |
|  |  |
|  | XXT4**, XYLOGLUCAN XYLOSYLTRANSFERASE 4**; Galactosyl transferase GMA12/MNN10 family protein; **xyloglucan metabolic process.** |
|  |  |
| **CER26**, ECERIFERUM 26; **HXXXD-type acyl-transferase family protein**; Functions in: transferase activity, transferring acyl groups other than amino-acyl groups, transferase activity; **cell wall organization, very long-chain fatty acid biosynthetic process, wax biosynthetic process.** | **MBOAT (membrane bound O-acyl transferase**) family protein; **lipid biosynthesis; wax.** |
| **KDO transferase A** Encodes a putative KDO (**3-deoxy-D-manno-octulosonate**) transferase; **lipopolysaccharide biosynthesis. Rhamnogalacturonan II biosynthesis.** |  |
|  |  |
| **Remorin family protein** | **Carbohydrate-binding X8 domain** superfamily protein |
|  | **Core-2/I-branching beta-1,6-N-acetylglucosaminyltransferase** family protein |
|  |  |
|  | **Purple acid phosphatase 3** |
|  |  |
|  |  |
| **INOSITOL TRANSPORT-METABOLISM-TRANSPORT-SIGNALING** | |
| **INDUCED** | **REPRESSED** |
|  | **Inositol monophosphatase** family protein |
|  | **Sec14p-like phosphatidylinositol transfer** family protein |
|  | **Sec14p-like phosphatidylinositol transfer** family protein |
|  | **Inositol transporter 3** |
|  |  |
|  | **ALTERED EXPRESSION OF APX2 8**, ALX8, ATFRY1, ATSAL1, FIERY1, FRY1, **HIGH EXPRESSION OF OSMOTICALLY RESPONSIVE GENES 2**, HOS2, RON1, ROTUNDA 1, SAL1, SUPO1, SUPPRESSORS OF PIN1 OVEREXPRESSION 1; **Encodes a bifunctional protein that has 3'(2'),5'-bisphosphate nucleotidase and inositol polyphosphate 1-phosphatase activities and rescues sulfur assimilation mutants in yeast.** It is involved in the **response to cold, drought (negative regulator of drought tolerance), and ABA**. **Mutants in this gene exhibit enhanced induction of stress genes in response to cold, ABA, salt and dehydration due to higher accumulation of the second messenger, inositol (1,4,5)- triphosphate (IP(3)).** Involved in degradation of small mRNAs. Mutants also affect the accumulation of miRNA target cleavage products. Regulates light-dependent repression of hypocotyl elongation and flowering time via its 3'(2'),5'-bisphosphate nucleotidase activity. |
|  |  |
|  |  |
|  | **RING/FYVE/PHD-type zinc** finger family protein  superfamily protein; **phosphatidylinositol binding**, zinc ion binding; **guard cell** |
|  |  |
| **Inositol-pentakisphosphate 2-kinase** family protein | **Phosphatidylinositol-4-phosphate 5-kinase** family protein |
| **Myo-inositol monophosphatase like 1** | **Inositol 1,3,4-trisphosphate 5/6-kinase** family protein |
| **Inositol monophosphatase** family protein |  |
| **Inositol monophosphatase** family protein; Involved in: sulfur metabolic process |  |
|  |  |

| **TRANSCRIPTION FACTORS** | |
| --- | --- |
| **INDUCED** | **REPRESSED** |
| **Myb domain protein 117** | **Myb domain protein 16** |
| **Myb domain protein 58** | **Myb domain protein 28** |
| myb family transcription factor | **Myb domain protein 50** |
| Duplicated homeodomain-like superfamily protein; **MAMYB, MEMBRANE ANCHORED MYB**; **The gene encodes a MYB transcription factor belons to R2R3-MYB family of transcription factors.** Knock-down mutant analysis indicates its role in root hair elongation. | **Duplicated homeodomain-like** superfamily protein |
| **Duplicated homeodomain-like** superfamily protein; **ATMYB38**, BIT1, BLUE INSENSITIVE TRAIT 1**, MYB DOMAIN PROTEIN 38**, MYB38, RAX2, REGULATOR OF AXILLARY MERISTEMS 2; "Putative homolog of the Blind gene in tomato. Together with RAX1 and RAX3 belong to the class R2R3 MYB genes; encoded by the Myb-like transcription factor MYB38, regulates axillary meristem formation. **Blue light signaling pathway**, multicellular organismal development, regulation of transcription, DNA-templated, **response to abscisic acid, response to gibberellin, response to jasmonic acid, response to salicylic acid.** | **Homeodomain-like/winged-helix DNA-binding** family protein |
| **Homeodomain-like** superfamily protein | **Homeodomain-like protein with RING/FYVE/PHD-type zinc** **finger** domain |
| **Alfin-like 4**, Encodes a member of the Alfin1-like family of nuclear-localized PHD (**plant homeodomain**) domain containing proteins. **All AL proteins except AL3 bind to di- or trimethylated histone H3 (H3K4me3/2).** | **Homeodomain-like superfamily** protein |
| ***Arabidopsis thaliana* INHIBITOR OF GROWTH 1**, ATING1, ING1, INHIBITOR OF GROWTH 1; ING1 encodes a member of the Inhibitor of Growth family of **nuclear-localized PhD domain containing homeodomain proteins**. **Binds to H3K4 di or trimethylated DNA. chromatin modification, regulation of growth, regulation of transcription**, (RING/FYVE/PHD zinc finger superfamily protein). | **Homeodomain-like** superfamily protein |
|  | **Homeodomain-like** superfamily protein |
|  |  |
| **NAC domain containing protein 36** | **NAC domain containing protein 11** |
| NAC (No Apical Meristem) domain transcriptional regulator superfamily protein; ANAC002, ANAC2, **ARABIDOPSIS NAC DOMAIN CONTAINING PROTEIN 2**, ATAF1; Belongs to a large family of putative transcriptional activators with NAC domain. **Transcript level increases in response to wounding and abscisic acid.** **ATAF1 attentuates ABA signaling and synthesis**. Multicellular organismal development, **negative regulation of abscisic acid-activated signaling pathway,** regulation of transcription, DNA-templated, **response to wounding.** | **NAC domain containing protein 1** |
| ANAC100, ATNAC5, **NAC DOMAIN CONTAINING PROTEIN 100**, NAC100, NAC domain containing protein 100**; multicellular organismal development.** | **NAC domain containing protein 3** |
|  |  |
| **Basic helix-loop-helix (bHLH) DNA-binding** superfamily protein | **Basic helix-loop-helix 32** |
| **Basic helix-loop-helix (bHLH) DNA-binding** superfamily protein | **Basic helix-loop-helix (bHLH) DNA-binding** family protein |
| Encodes a atypical member of the **bHLH (basic helix-loop-helix)** family transcriptional factors. | **Basic helix-loop-helix (bHLH) DNA-binding** superfamily protein |
| **Cryptochrome-interacting basic-helix-loop-helix 5**; CIB5, CRYPTOCHROME-INTERACTING BASIC-HELIX-LOOP-HELIX 5; Encodes CIB5 (cryptochrome-interacting basic-helix-loop-helix). Related to CIB1 (AT4G34530). **CIB5 interacts with CRY2 and forms heterodimer with CIB1 *in vitro***. Regulates flowering time redundantly with CIB1. positive regulation of flower development, regulation of growth | **Basic helix-loop-helix (bHLH) DNA-binding** superfamily protein |
|  | **Basic helix-loop-helix (bHLH) DNA-binding** superfamily protein |
|  |  |
| ABRE binding factor 4; **ABA-RESPONSIVE ELEMENT BINDING PROTEIN 2,** ABF4, ABRE BINDING FACTOR 4, AREB2, ATAREB2; **bZIP transcription factor with specificity for abscisic acid-responsive elements (ABRE).** Mediate ABA-dependent stress responses; **response to abscisic acid, response to salt stress, response to water deprivation.** | ATBZIP52, **BASIC LEUCINE-ZIPPER 52**, BZIP52 |
| basic leucine-zipper 1; ATBZIP1, **BASIC LEUCINE-ZIPPER 1**, BZIP1; Encodes a transcription activator is **a positive regulator of plant tolerance to salt, osmotic and drought stresses.** |  |
| **Basic leucine zipper 25** |  |
| **Basic region/leucine zipper motif protein 49** |  |
|  |  |
| **C2H2 and C2HC zinc fingers** superfamily protein | **C2H2-type zinc finger** family protein |
| **C2H2-like zinc finger** protein | **C2H2-type zinc finger** family protein |
| **C2H2-type zinc finger** family protein; EXPRESSED IN: flower; EXPRESSED DURING: **petal differentiation and expansion stage.** | **Zinc finger (C2H2 type**) family protein |
|  | **Zinc finger (C2H2 type)** family protein |
|  |  |
| **WRKY family** transcription factor | **WRKY DNA-binding protein 10** |
| **WRKY DNA-binding protein 60**; **Pathogen-induced transcription factor. F**orms protein complexes with itself and with WRKY40**. Coexpression with WRKY18 or WRKY40 made plants more susceptible to both *P. syringae* and *B. cinerea*.** WRKY18, WRKY40, and WRKY60 have partially redundant roles in response to the hemibiotrophic bacterial pathogens. | **WRKY DNA-binding protein 71** |
|  | **WRKY DNA-binding protein 54** |
|  |  |
| AGAMOUS-like; **AGAMOUS-LIKE 8**, AGL8, FRUITFULL, FUL: **MADS box gene negatively regulated by APETALA1**; cell differentiation, flower development, fruit development, **maintenance of inflorescence meristem identity, positive regulation of flower development.** | **AHL21, AT-HOOK MOTIF NUCLEAR LOCALIZED PROTEIN 21**, GIANT KILLER, GIK; **Direct target of AGAMOUS**. **Regulates patterning and differentiation of reproductive organs**; anther development, ovule development. |
|  | **AGAMOUS-like 48** |
|  | **AGAMOUS-like 20** |
|  | **AGAMOUS-like 23** |
|  |  |
| **Nuclear factor Y, subunit C10** | **Nuclear factor Y, subunit B8** |
| **Nuclear factor Y, subunit B3** | **Nuclear factor Y, subunit C6** |
|  |  |
| Related to AP2 1; **RAP2.1, RELATED TO AP2 1; encodes a member of the DREB subfamily A-5 of ERF/AP2 transcription factor family (RAP2.1).** The protein contains one AP2 domain. There are 16 members in this subfamily including RAP2.9 and RAP2.10. ethylene-activated signaling pathway, positive regulation of transcription, DNA-templated, regulation of transcription, DNA-templated, **response to cold, response to water deprivation.** | **AP2/B3-like transcriptional factor** family protein |
| Integrase-type DNA-binding superfamily protein (2.3): encodes a member of the **DREB subfamily A-6 of ERF/AP2 transcription factor family.** The protein contains one AP2 domain. **There are 8 members in this subfamily including RAP2.4. Ethylene-activated signaling pathway.** |  |
| Integrase-type DNA-binding superfamily protein (2.17); **encodes a member of the DREB subfamily A-6 of ERF/AP2 transcription factor family.** The protein contains one AP2 domain. **There are 8 members** in this subfamily including **RAP2.4**. |  |
| Integrase-type DNA-binding superfamily protein (2.01); **encodes a member of the DREB subfamily A-4 of ERF/AP2 transcription factor family.** The protein contains one AP2 domain. **There are 17 members in this subfamily including TINY.** |  |
|  |  |
| **GATA transcription factor 23** | **Squamosa promoter binding protein-like 8** |
| **Transcriptional factor B3 family protein ATREM1**, REM1, REM34, REPRODUCTIVE MERISTEM 1, REPRODUCTIVE MERISTEM 34; **Expressed specifically in reproductive meristems**, member of a moderately sized gene family distantly related to known plant DNA binding proteins. | Plus-3 domain-containing protein; **Encodes a yeast Paf1C subunit homolog required for the expression of the MADS box gene FLC and other members of the FLC/MAF MADS-box gene family.** |
|  | **MADS-box transcription factor** family protein |
|  |  |
| **Zinc ion binding; transcription regulators** | **TCP family transcription factor** |
|  | **TCP family transcription factor**; regulation **of circadian rhythm** |
|  | **Winged helix-turn-helix transcription repressor** DNA-binding |
|  | **Winged-helix DNA-binding transcription factor** family protein |
|  | **Dof-type zinc finger DNA-binding family protein ATDOF5.8** |
|  | **BES1-interacting Myc-like protein 2** |
|  | **KNOTTED1-like homeobox gene 5** |
|  | **GRAS family** transcription factor |
|  | EMB173, **EMBRYO DEFECTIVE 173**, FERTILIZATION INDEPENDENT SEED 1, FIS1, MEA, MEDEA, SDG5, SET DOMAIN-CONTAINING PROTEIN 5; **Encodes a putative transcription factor MEDEA (MEA) that negatively regulates seed development in the absence of fertilization**. Mutations in this locus result in embryo lethality. |
|  | **General transcription factor 2-related zinc finger** protein |
|  |  |

| **TRANSPORT** **IONIC AND OSMOTIC HOMEOSTASIS** | |
| --- | --- |
| **INDUCED** | **REPRESSED** |
| **Major facilitator superfamily** protein; FUNCTIONS IN: transporter activity; INVOLVED IN: transport; LOCATED IN: membrane; CONTAINS InterPro DOMAIN/s: **Biopterin transport-related protein BT1.** | **Nodulin-like** (InterPro:IPR010658), **Major facilitator superfamily** MFS-1; Expressed in: sperm cell, male gametophyte. |
| **Major facilitator superfamily** protein; CONTAINS InterPro DOMAIN/s: Major facilitator superfamily, general substrate transporter (InterPro:IPR016196), **Biopterin transport-related protein BT1.** | **Major facilitator superfamily** protein. |
|  |  |
| **Other SUGAR TRANSPORTERS** | |
| **INDUCED** | **REPRESSED** |
| **Major facilitator superfamily** protein; Functions in: carbohydrate transmembrane transporter activity, **sugar: hydrogen symporter activity**; Involved in: transport, transmembrane transport; **sugar transporter 1** | **Major facilitator superfamily** protein; Functions in: carbohydrate transmembrane transporter activity, **sugar: hydrogen symporter activity.** |
| **Major facilitator superfamily** protein; **sugar: hydrogen symporter** activity; **vacuolar glucose transporter 1** | **Major facilitator superfamily** protein; FUNCTIONS IN: carbohydrate transmembrane transporter activity, **sugar: hydrogen symporter activity; polyol/ monosaccharide transporter** **5.** |
| **Major facilitator superfamily** protein; Functions in: carbohydrate transmembrane transporter activity, **sugar: hydrogen symporter** activity; **Sugar/ inositol transporter** | **Vacuolar glucose transporter 1** |
| **Nodulin MtN3** family protein; **ATSWEET13**, SWEET13  **Encodes a member of the SWEET sucrose efflux transporter** family proteins. | **UDP-galactose transporter 3** |
| **Nucleotide-sugar transporter** family protein | **Nucleotide/sugar transporter** family protein |
| **Glucose-6-phosphate/phosphate translocator-related** |  |
|  |  |
| **METAL TRANSPORTERS** | |
| **INDUCED** | **REPRESSED** |
| **Heavy metal transport/detoxification** superfamily protein | Copper transporter 1 |
| ***Arabidopsis thaliana* NATURAL RESISTANCE ASSOCIATED MACROPHAGE PROTEIN 4**, ATNRAMP4, NATURAL RESISTANCE ASSOCIATED MACROPHAGE PROTEIN 4, NRAMP4; **Encodes a member of the Nramp2 metal transporter family**; like its homolog Atnramp3, localized in vacuolar membrane. **Anion transmembrane transport, cadmium ion transport, cellular iron ion homeostasis, cellular metal ion homeostasis,** defense response to bacterium, iron ion homeostasis, lead ion transport, manganese ion transport, metal ion transport, positive **regulation of reactive oxygen species** metabolic process, response to iron ion, response to **nematode** | **Aluminium activated malate transporter** family protein |
| **Cobalt ion transmembrane** transporters | **Magnesium transporter 4** |
|  | **Magnesium transporter CorA-like** family protein |
|  | **Heavy metal transport/detoxification** superfamily protein |
|  | **Zinc transporter 4** precursor |
|  | **ZIP metal ion transporter** family |
|  | **Cadmium tolerance 1** |
|  | **Magnesium transporter CorA-like** family protein |
|  | **Heavy metal transport/detoxification** superfamily protein |
|  | **Copper-exporting ATPase** / responsive-to-antagonist 1 / copper-transporting ATPase (RAN1). |
|  | **ZIP metal ion transporter** family |
|  |  |
| **ION TRANSPORTERS** | |
| **INDUCED** | **REPRESSED** |
| **Calcium-transporting ATPase,** putative | **Sodium hydrogen exchanger 4** |
| **Vacuolar H^+^-pumping ATPase 16 kDa proteolipid subunit 4**; vacuolar acidification. | **Cation exchanger 6** |
| ATCOX6B2, COX6B, **CYTOCHROME C OXIDASE 6B**, CYTOCHROME C OXIDASE 6B2; subunit 6b of cytochrome c oxidase; **hydrogen ion transmembrane transport, oxidation-reduction process, response to salt stress.** | **Cation exchanger 5** |
|  | **Vacuolar ATP synthase subunit C** (VATC) / V-ATPase C subunit / **vacuolar proton pump C subunit** (DET3) |
|  | **Vacuolar proton ATPase A2** |
|  |  |
| **NUCLEAR, MITOCHONDRIAL OR CHLOROPLAST TRANSPORTERS** | |
| **INDUCED** | **REPRESSED** |
| **Nuclear pore localisation protein NPL4** | **Mitochondrial import inner membrane translocase subunit Tim17**/Tim22/Tim23 family protein |
| **Chloroplast import apparatus 2**; CHLOROPLAST IMPORT APPARATUS 2, CIA2; Transcription regulator responsible for specific upregulation of the translocon genes atToc33 and atToc75 in leaves. **Involved in protein import into chloroplast.** | **Mitochondrial substrate carrier** family protein |
|  | **Mitochondrial substrate carrier** family protein |
|  |  |
| **AMINO ACID OR OLIGOPEPTIDE TRANSPORTERS** | |
| **INDUCED** | **REPRESSED** |
| **Peptide transporter 3** | **Amino acid permease** family protein |
| **Nodulin MtN21 /EamA-like transporter** family protein; UMAMIT11, USUALLY MULTIPLE ACIDS MOVE IN AND OUT TRANSPORTERS 11; **Encodes a plasma membrane-localized amino acid transporter likely involved in amino acid export in the developing seed.** | **Cationic amino acid** transporter |
|  | **Cationic amino acid transporter 6** |
|  | **Transmembrane amino acid transporter** family protein |
|  | **Major facilitator superfamily** protein; FUNCTIONS IN: transporter activity; Involved in: **oligopeptide transport**. |
|  |  |
|  |  |
| **LIPID/ STEROL TRANSPORTERS** | |
| **INDUCED** | **REPRESSED** |
| **Polyketide cyclase/dehydrase and lipid transport** superfamily protein | **Polyketide cyclase/dehydrase and lipid transport** superfamily protein |
|  | **Lipid transporter** |
|  |  |
| **VESICLE / PROTEIN TRANSPORT: ER, GOLGI, ETC.** | |
| **INDUCED** | **REPRESSED** |
| **Delta-adaptin** | Alpha-adaptin |
| MAG5, **MAIGO 5**; RGPR-related, **COPII vesicle coating**, protein transport | Exocyst complex component sec10 |
| GCN5L1 family protein; **BLOC SUBUNIT 1**, BLOS1; endosomal transport, **late endosome to vacuole transport, root development.** | **Homolog of yeast autophagy 18 (ATG18) G**; autophagy, protein transport, **response to starvation.** |
| ATEXO70E1, EXO70E1, **EXOCYST SUBUNIT EXO70 FAMILY PROTEIN E1**; exocytosis, **vesicle docking involved in exocytosis.** | **Secretory 1A** |
| ATRER1C1**; Encodes AtRER1C1, a Golgi membrane protein involved in returning the molecules that are exported from the endoplasmic reticulum (ER) to the Golgi apparatus** back to the ER (a mechanism known as retrieval). | **Vesicle transport V-snare 13** |
|  | **Vacuolar protein sorting 26B** |
|  | **SEC7-like guanine nucleotide exchange family protein;** BIG3, EDA10, EMBRYO SAC DEVELOPMENT ARREST 10; Encodes one of the functionally redundant ARF guanine-nucleotide exchange factors (ARF-GEFs). **Functions as regulators of post-Golgi trafficking.** |
|  | **Adaptin** family protein |
|  | **SNARE associated Golgi protein** family |
|  | **ATRER1C1**; Encodes AtRER1C1, **a Golgi membrane protein involved in returning the molecules that are exported from the endoplasmic reticulum (ER**) to the Golgi apparatus back to the ER (a mechanism known as retrieval). |
|  | **SNARE-like superfamily** protein; **located in chloroplast** |
|  | **Syntaxin/ t-SNARE** family protein; Involved in: **Golgi vesicle transport,** vesicle-mediated transport |
|  | **VESICLE TRANSPORT V-SNARE 13**; intra-Golgi vesicle-mediated transport. |
|  | **GOLGI SNARE 11**; ER to **Golgi vesicle-mediated transport,** intra-Golgi vesicle-mediated transport, protein transport. |
|  | **ATSFT12;** **Encodes a golgi localized QcSNARE involved in response to salt and osmotic stress**. **Overexpression confers increased resistance to NaCl, mannitol and LiCl.** SFT12 may act by mediating **vacuolar sequestration of NaCl and other ions.** |
|  | **Endomembrane protein 70** protein family; INVOLVED IN: transport; LOCATED IN: integral to membrane, Golgi apparatus, membrane. |
|  | **Bacterial sec-independent translocation protein mttA/Hcf106;** Functions in: protein transporter activity; Involved in: protein secretion, protein transport; Located in: **chloroplast thylakoid membrane, chloroplast envelope.** |
|  |  |
| **PHYTOHORMONE-RELATED TRANSPORT** | |
| **INDUCED** | **REPRESSED** |
| EMB30, **EMBRYO DEFECTIVE 30**, GN, GNOM, VAN7, VASCULAR NETWORK 7; Encodes a GDP/GTP exchange factor for small G-proteins of the ADP ribosylation factor (RAF) class, and as regulator of intracellular trafficking. Homologous to Sec7p and YEC2 from yeast. Involved in the specification of apical-basal pattern formation. Essential for cell division, expansion and adhesion.  **Anion transmembrane transport, basipetal auxin transport,** cell adhesion, **cell wall organization**, cytokinesis by cell plate formation, embryo development ending in seed dormancy. Embryonic pattern specification, endocytosis, endosome transport via multivesicular body sorting pathway, establishment of planar polarity, **guanine nucleotide transmembrane transport**, lateral root formation, longitudinal axis specification, organic anion transport, **phloem or xylem histogenesis, purine nucleoside transmembrane transport,** **root hair cell differentiation**, unidimensional cell growth, vesicle-mediated transport. | **Purine permease 4** |
| **AMINOPEPTIDASE M1**, APM1, ATAPM1; encodes an aminopeptidase, a ortholog of mouse microsomal AP (EC 3.4.11.2). **Auxin polar transport**, peptide catabolic process, proteolysis. | **Purine permease 11** |
| **Early-responsive to dehydration stress protein (ERD4**); **ion transport.** | ABCC5, ***Arabidopsis thaliana* ATP-BINDING CASSETTE C5**; encodes a **high-affinity inositol hexakisphosphate transporter that plays a role in guard cell signaling and phytate storage**; **Abscisic acid-activated signaling pathway involved in stomatal movement**, cellular potassium ion homeostasis, **response to salt stress,** stomatal opening. |
|  | ATPUP6, PUP6, **PURINE PERMEASE 6**; Member of a family of proteins related to PUP1, a purine transporter. May be involved in the **transport of purine and purine derivatives such as cytokinins, across the plasma membrane.** |
|  |  |
| **WATER TRANSPORTERS** | |
| **INDUCED** | **REPRESSED** |
| PIP1;4, PIP1E, **PLASMA MEMBRANE INTRINSIC PROTEIN 1;4**, PLASMA MEMBRANE INTRINSIC PROTEIN 1E, TMP-C, TRANSMEMBRANE PROTEIN C; a member of the plasma membrane intrinsic protein subfamily PIP1. **Response to water deprivation, transport, water transport** | **Aquaporin-like superfamily** protein |
|  |  |
| **PHOSPHATE TRANSPORTERS** | |
| **INDUCED** | **REPRESSED** |
|  | **EXS (ERD1/XPR1/SYG1)** family protein**; phosphate ion transport** |
|  | **Phosphate transporter 4;3** |
|  | **Phosphate transporter 1;9** |
|  |  |
| **OTHERS** | |
| **INDUCED** | **REPRESSED** |
| **Cyclic nucleotide gated channel 5** | **Cyclic nucleotide-gated channel 17** |
| **MATE efflux** family protein | **MATE efflux** family protein |
|  | **ATP-BINDING CASSETTE G14** |
|  | **Root hair specific 2**; MATE; antiporter activity, drug transmembrane transporter activity, transporter activity; **root hair elongation.** |
|  | UMAMIT41, **USUALLY MULTIPLE ACIDS MOVE IN AND OUT TRANSPORTERS 41**; **nodulin MtN21-like transporter family** protein. |
|  | MATE efflux family protein |
|  | **ATP-BINDING CASSETTE C7**; involved in response to other organism. |
|  | **NON-INTRINSIC ABC PROTEIN 5**; expressed in guard cell |
|  | **Endomembrane protein 70** protein family; CONTAINS InterPro DOMAIN/s: Nonaspanin (TM9SF); membrane fusion, **response to nematode.** Located in Golgi membrane. |
|  | **NITRATE TRANSPORTER 1.12,** NRT1.12; Encodes a low affinity nitrate transporter that is expressed in the plasma membrane. **Also found in the phloem of the major veins of leaves**. It is responsible for nitrate redistribution to young leaves. **Anion transmembrane transport, low-affinity nitrate transport, oligopeptide transport**. |
|  |  |

| **PHYTOHORMONES, DEVELOPMENT, (A)BIOTIC STRESS** | |
| --- | --- |
| **INDUCED** | **REPRESSED** |
| **ABA and/or MODIFIED BY SALT/ OSMOTIC STRESS** | |
| **Low-temperature-responsive protein 78 (LTI78) / desiccation-responsive protein 29A (RD29A);** COLD REGULATED 78, COR78, LOW-TEMPERATURE-INDUCED 78, LTI140, LTI78, RD29A, RESPONSIVE TO DESICCATION 29A; cold regulated gene, the 5' region of cor78 has cis-acting regulatory elements that can impart cold-regulated gene expression The mRNA is cell-to-cell mobile. **Hyperosmotic salinity response**, leaf senescence, **response to abscisic acid**, response to cold, response to desiccation, **response to osmotic stress, response to salt stress,** **response to water deprivation.** | **Arginine-rich cyclin 1**; Encodes an ania-6a type arginine-rich cyclin which **confers tolerance to LiCl and NaCl when expressed in yeast.** |
| **Cold, circadian rhythm, and RNA binding 1**; ATGRP8, CCR1, COLD, CIRCADIAN RHYTHM, AND RNA BINDING 1, GLYCINE-RICH PROTEIN 8, GLYCINE-RICH RNA-BINDING PROTEIN 8, GR-RBP8, GRP8, RBGA6, RNA-BINDING GLYCINE-RICH PROTEIN A6  **Encodes a glycine-rich protein with RNA binding domain** at the N-terminus. Protein is structurally similar to proteins induced by stress in other plants. **Gene expression is induced by cold.** Transcript undergoes circadian oscillations that is depressed by overexpression of AtGRP7. A substrate of the type III effector HopU1 (mono-ADP-ribosyltransferase). **Innate immune response, response to abscisic acid**, response to cold, response to cytokinin, **response to salt stress**, response to stress, response to zinc ion. |  |
| **RESPONSIVE TO DEHYDRATION 21B**, RD21B; **Granulin repeat cysteine protease family protein**; Functions in: cysteine-type peptidase activity, cysteine-type endopeptidase activity; Involved in: **response to salt stress**; Located in: **vacuole** |  |
|  |  |
| **ABSCISIC ACID-DEHYDRATION-SIGNAL TRANSDUCTION** | |
| **INDUCED** | **REPRESSED** |
| ATDOG1, **DELAY OF GERMINATION 1**, DOG1, GAAS5, GERMINATION ABILITY AFTER STORAGE 5**, GLUCOSE SENSING QTL 5**, GSQ5; Encodes DOG1 (DELAY OF GERMINATION 1). A quantitative trait locus involved in the control of seed dormancy. **Abscisic acid-activated signaling pathway**, regulation of seed dormancy process, seed dormancy process, **sugar mediated signaling pathway.** | **ABA DEFICIENT 3**, ABA3, ACI2, ALTERED CHLOROPLAST IMPORT 2, ATABA3, ATLOS5, LOS5, LOW OSMOTIC STRESS 5, SIR3, SIRTINOL RESISTANT 3; **Encodes molybdenum cofactor sulfurase.** **Involved in the conversion of ABA-aldehyde to ABA, the last step of abscisic acid (ABA) biosynthesis. Auxin-activated signaling pathway,** defense response to bacterium, molybdenum incorporation into molybdenum-molybdopterin complex, protein import into chloroplast stroma, **response to cold, response to heat, response to osmotic stress, response to salt stress, stomatal movement, sugar mediated signaling pathway.** |
| MAKR6, **MEMBRANE-ASSOCIATED KINASE REGULATOR 6**; (1-6) **abscisic acid-activated signaling pathway.** | **Dehydrin xero 1** |
| **SNF1-related protein kinase 2.9** |  |
| **D111/G-patch domain-containing protein**; Functions in: nucleic acid binding; **suppressor of ABI3-5** |  |
|  |  |
| **AUXIN RESPONSES-LEA-IAA-SAUR** | |
| **INDUCED** | **REPRESSED** |
| **SAUR-like auxin-responsive** protein family | **SAUR-like auxin-responsive** protein family |
| **Indole-3-acetate beta-D-glucosyltransferase**; UDP-glucose: indole-3-acetate beta-D-glucosyltransferase; **response to toxic substance.** | **Like AUXIN RESISTANT** 2; LAX2, Encodes LAX2 (LIKE AUXIN RESISTANT), **a member of the AUX1 LAX family of auxin influx carriers.** Required for the establishment of **embryonic root cell organization.** |
| **Iindole-3-acetic acid 7**; AUXIN RESISTANT 2, AXR2, IAA7, INDOLE-3-ACETIC ACID 7; **auxin-activated signaling pathway, gravitropism,** regulation of transcription, DNA-templated, **response to auxin, response to jasmonic acid, response to water deprivation, response to wounding**, transcription, DNA-templated | **Auxin response factor 21** |
| **Lateral root primordium (LRP) protein-related**; **LATERAL ROOT PRIMORDIUM 1**, LRP1; A member of SHI gene family. *Arabidopsis thaliana* has ten members that encode proteins with a RING finger-like zinc finger motif. Despite being highly divergent in sequence, many of the SHI-related genes are partially redundant in function and synergistically promote gynoecium, stamen and leaf development in Arabidopsis. **Expressed in lateral root primordia and induced by auxin**. **SWP1 is involved in the repression of LRP1 via histone deacetylation. Auxin biosynthetic process, auxin-activated signaling pathway**, multicellular organismal development, response to auxin, **root development.** | Indole-3-acetic acid 6; **IAA6, INDOLE-3-ACETIC ACID 6**, **SHORT HYPOCOTYL 1**, SHY1; An extragenic dominant suppressor of the hy2 mutant phenotype. **Auxin-activated signaling pathway, de-etiolation.** |
| ARAC3, ATROP6, RAC-LIKE 3, RAC3**, RHO-RELATED PROTEIN FROM PLANTS 6**, RHO1PS, ROP6; **A member of ROP GTPase gene family**; Encodes a Rho-like GTP binding protein. **auxin-activated signaling pathway, positive gravitropism, regulation of auxin mediated signaling pathway.** | **YUCCA 9 flavin monooxygenases.** |
| **SHI-RELATED SEQUENCE 5**, SRS5; A member of SHI gene family. Arabidopsis thaliana has ten members that encode proteins with a RING finger-like zinc finger motif. **Auxin biosynthetic** process**, auxin-activated signaling pathway**, **gynoecium development, multicellular organismal development.** | **SAUR-like auxin-responsive** protein family |
| CYP79A2, **CYTOCHROME P450 79A2**; Encodes cytochrome P450 CYP79A2. **defense** response to other organism, **glucosinolate biosynthetic process**, **indoleacetic acid biosynthetic process** | **SUPPRESSOR OF AUXIN RESISTANCE1** |
| SKIP2, **SKP1/ASK1-INTERACTING PROTEIN 2,** VFB4, VIER F-BOX PROTEINE 4; Encodes an SKP1 interacting partner (SKIP2).**Encodes an F-box protein**. Based on genetic analysis appears to be functionally redundant with VFB1,2, and 3. **When expression of all 4 genes is reduced plants show defects in growth and reduced expression of auxin response genes.** | **SAUR-like auxin-responsive** protein family |
| **PHOSPHOLIPASE A2-BETA**, PLA2-BETA; Encodes one of the four Arabidopsis phospholipase PLA2 parologs: AT2G06925 (PLA2-ALPHA), AT2G19690 (PLA2-BETA), AT4G29460 (PLA2-GAMMA) and AT4G29470 (PLA2-DELTA**). Involved in pollen development and germination and tube growth**. Also involved in stomatal opening in response to light**. gravitropism**, lipid catabolic process, phospholipid metabolic process, **pollen development, pollen germination, pollen tube growth, positive regulation of cell growth, r**egulation of **stomatal movement**, **response to auxin, response to light stimulus.** | **AUX/IAA transcriptional regulator** family protein |
| **Late embryogenesis abundant (LEA) hydroxyproline-rich** glycoprotein family | **Dormancy/ auxin associated** family protein |
| **Late embryogenesis abundant protein, putative / LEA protein**, putative | **Auxin efflux carrier** family protein |
| **Late embryogenesis abundant (LEA) hydroxyproline-rich** glycoprotein family | **AUXIN RESPONSE FACTOR 1** |
| **Late embryogenesis abundant protein, group 2; hydroxyproline-rich** glycoprotein family | **Indole-3-acetic acid inducible 9** |
| **Late embryogenesis abundant (LEA) hydroxyproline-rich** glycoprotein family; NDR1/HIN1-like 1 |  |
| **Glycine-rich protein / late embryogenesis abundant protein** |  |
|  |  |
| **BRASSINOESTEROIDS-DEVELOPMENT** | |
| **INDUCED** | **REPRESSED** |
| **BR enhanced expression 3**; BEE3, BR ENHANCED EXPRESSION 3  Encodes the brassinosteroid signaling component BEE3 (BR-ENHANCED EXPRESSION 3). **Positively modulates the shade avoidance syndrome in Arabidopsis seedlings**. | BEE1, **BR ENHANCED EXPRESSION 1**; Encodes the brassinosteroid signaling component BEE1 (BR-ENHANCED EXPRESSION 1). **Positively modulates the shade avoidance syndrome in Arabidopsis seedlings** |
|  |  |
| **CYTOKININ-CELL DIVISION-GROWTH-DEVELOPMENT** | |
| **INDUCED** | **REPRESSED** |
| ADA2B, **HOMOLOG OF YEAST ADA2 2B**, PROPORZ1, PRZ1  Transcriptional co-activator. **Essential for the developmental switch from cell proliferation to cell differentiation in response to variations in auxin and cytokinin concentrations.** | **Cytokinin response factor 2** |
|  | **ARR3; response regulator** 3; Type A response regulator highly similar to bacterial two-component response regulators. **Rapidly induced by cytokinin**. **Involved in red-light signaling. Acts redundantly with ARR3 in the control of circadian period in a cytokinin-independent manner.** |
|  |  |
| **ETHYLENE-JASMONIC ACID** | |
| **INDUCED** | **REPRESSED** |
| **Ethylene-dependent gravitropism-deficient and yellow-green-like 2**; S2P-like **putative metalloprotease**; involved in proteolysis | **Ethylene-dependent gravitropism-deficient and yellow-green-like 2** |
|  | **OPC-8:0 COA LIGASE1**, OPCL1; OPC-8:0 CoA ligase1 (OPCL1); FUNCTIONS IN: 4-coumarate-CoA ligase activity; Involved in: **phenylpropanoid metabolic process**, **jasmonic acid biosynthetic** process, response to **wounding**; Located in: peroxisome. |
|  |  |
| **GIBBERELLINS** | |
| **INDUCED** | **REPRESSED** |
|  | **Gibberellin-regulated** family protein |
|  | **Gibberellin 3-oxidase 2** |
|  | **Gibberellin 3-oxidase 1** |
|  | **RGA-LIKE 1, RGL, RGL1**; **Negative regulator of GA responses**, **member of GRAS family of transcription factors**. Also belongs to the **DELLA proteins** that restrain the cell proliferation and expansion that drives plant growth. **RGL1 may be involved in reducing ROS accumulation in response to stress by up-regulating the transcription of superoxide dismutases**. Rapidly degraded in response to GA. Involved in flower and fruit development, cell differentiation**, flower development**. **Gibberellic acid mediated signaling pathway, hyperosmotic salinity response**, **jasmonic acid mediated signaling pathway**, **negative regulation of gibberellic acid mediated signaling** pathway, **regulation of reactive oxygen species metabolic proces**s, regulation of transcription, DNA-templated, **response to abscisic acid, response to ethylene, response to gibberellin, response to salt stress, salicylic acid mediated signaling pathway.** |
|  |  |
| **GENERAL RESPONSE TO ABIOTIC STRESS** | |
| **INDUCED** | **REPRESSED** |
| ATTYDC, **L-TYROSINE DECARBOXYLASE, L-TYROSINE DECARBOXYLASE 1**, TYDC, TYRDC, TYRDC1; **Encodes a stress-induced tyrosine decarboxylase (TyrDC).** Recombinant (His)6-TyrDC expressed in E. coli catalyzes the conversion of L-tyrosine to tyramine. cellular amino acid metabolic process, **response to water deprivation, response to wounding**, tyramine biosynthetic process. | **Dehydration-induced protein (ERD15)** |
| ATSR2, ATSRPK1, **CBL-INTERACTING PROTEIN KINASE 7**, CIPK7, PKS7, **SNF1-RELATED PROTEIN KINASE 3.10**, SNRK3.10; Encodes a serine/threonine protein kinase with similarities to CBL-interacting protein kinases, SNF1 and SOS2. The mRNA is cell-to-cell mobile. **Response to cold.** | **ERD (early response to dehydration) six-like 1** |
|  | **Early-responsive to dehydration protein-related** / ERD protein-related |
|  | **Drought-repressed 4** |
|  | OLE2, OLEO2, **OLEOSIN 2**; **Encodes oleosin2, a protein found in oil bodies**, involved in seed lipid accumulation. lipid storage, **response to freezing**, seed germination, seed oil body biogenesis |
|  | **Low temperature and salt responsive** protein family |
|  | **Zinc knuckle (CCHC-type) family protein; cold shock domain protein 1** |
|  | **Senescence associated gene 20** |
|  |  |
| **OTHERS: DEFENSE, DEVELOPMENT** | |
| **INDUCED** | **REPRESSED** |
| **Cysteine/Histidine-rich C1** domain family protein | **Cysteine/Histidine-rich C1** domain family protein |
| **Cysteine/Histidine-rich C1** domain family protein | **Cysteine/Histidine-rich C1** domain family protein |
| DC1 domain-containing protein**; Cysteine/Histidine-rich C1 domain** family protein | **Cysteine/Histidine-rich C1** domain family protein |
|  | **Cysteine/Histidine-rich C1** domain family protein |
|  | **Cysteine/Histidine-rich C1** domain family protein |
|  |  |
| **Disease resistance protein (TIR-NBS-LRR class)** family | **Receptor like protein 21** |
| **Disease resistance protein (TIR-NBS-LRR class)** family | **Cysteine-rich RLK (RECEPTOR-like protein kinase) 2** |
| **Disease resistance-responsive (dirigent-like protein)** family protein | **RPM1 interacting protein 2** |
| **Cysteine-rich RLK (RECEPTOR-like protein kinase) 19** | **Disease resistance protein (CC-NBS-LRR class)** family |
|  |  |
| **Proteinase inhibitor**, propeptide | **Kunitz family trypsin and protease inhibitor** protein |
| **HOMOLOG OF RPW8 2**, HR2; **plant-type hypersensitive response, response to fungus**, response to other organism. | **BAX inhibitor 1** |
|  | **Pathogenesis-related** family protein |
|  | **CAP (Cysteine-rich secretory proteins, Antigen 5, and Pathogenesis-related 1 protein)** superfamily protein |
|  | ***Arabidopsis thaliana* METHYL ESTERASE 15**, ATMES15, MES15, METHYL ESTERASE 15, RHS9, ROOT HAIR SPECIFIC 9**; Encodes a protein predicted to act as a carboxylesterase. It has similarity to the SABP2 methyl salicylate esterase from tobacco but no enzymatic activity has been identified for this protein.** |
|  |  |
| **Cyclin p3;2**; cell cycle, cell division, **regulation of cell cycle,** **regulation of cyclin-dependent protein serine/threonine kinase activity.** | **Cell division control, Cdc6** |
| **Cyclin d5;1**; **DNA endoreduplication, cell division**, regulation of cell cycle. | **Cyclin b3;1** |
|  | **Cyclin D6;1** |
|  |  |
| APRR5, PRR5, **PSEUDO-RESPONSE REGULATOR 5**; **Encodes a pseudo-response regulator whose mutation affects various circadian-associated biological events such as flowering time in the long-day photoperiod conditions**, **red light sensitivity** of seedlings during early photomorphogenesis, and the period of free-running rhythms of certain clock-controlled genes including CCA1 and APRR1/TOC1 in constant white light. **Acts as transcriptional repressor of CCA1 and LHY.** | **FRIGIDA INTERACTING PROTEIN 1**, FIP1; Encodes one of the FRI interacting proteins: FRIGIDA INTERACTING PROTEIN 1 (FIP1)/At2g06005, FIP2/ At4g17060. **FRI (At4G00650) is a major determinant of natural variation in Arabidopsis flowering time** |
| SAP**, STERILE APETALA**; A recessive **mutation** in the Arabidopsis STERILE APETALA (SAP) causes **severe aberrations in inflorescence and flower and ovule development**. cell differentiation, flower development, megasporogenesis, regulation of transcription, (**Transducin/WD40 repeat-like** superfamily protein), | **SPX domain gene 2**; **cellular response to phosphate starvation** |
| **REDUCED MALE FERTILITY**, RMF**; Encodes a nuclear localized F-box protein** that is involved in tapetal layer degeneration and pollen development. Interacts with ASK1 and that interaction is mediated by the F-box domain;  **anther wall tapetum development, microgametogenesis.** | **Senescence-associated** family protein |
| ATTSO1, **CHINESE FOR 'UGLY'**, TSO1; **floral organ morphogenesis, regulation of cell division, regulation of meristem structural organization** (Tesmin/TSO1-like CXC domain-containing protein). | **SET domain-containing protein**; EZA1, SDG10, **SET DOMAIN-CONTAINING PROTEIN 10,** SWINGER, SWN**; Encodes a polycomb group protein.** Forms part of a large protein complex that can include VRN2 (VERNALIZATION 2), VIN3 (VERNALIZATION INSENSITIVE 3) and polycomb group proteins FERTILIZATION INDEPENDENT ENDOSPERM (FIE) and CURLY LEAF (CLF). **The complex has a role in establishing FLC (FLOWERING LOCUS C) repression during vernalization.** Performs a partially redundant role to MEA in controlling seed initiation by helping to suppress central cell nucleusendosperm proliferation within the FG |
| ATNMNAT, **NICOTINATE/NICOTINAMIDE MONONUCLEOTIDE ADENYLTRANSFERASE**, NMNAT; encodes a bi-functional enzyme that expresses both nicotinamide-nucleotide adenylyltransferase (2.7.7.1) and nicotinate-nucleotide adenylyltransferase (2.7.7.18)activity. 'de novo' NAD biosynthetic process from aspartate, **NAD biosynthetic process, biosynthetic process, pollen development, pollen tube growth.** |  |
| CBP1, **CCG-BINDING PROTEIN 1**, **MATERNAL EFFECT EMBRYO ARREST 14**, MEE14; Encodes CBP1, a regulator of transcription initiation in central cell-mediated pollen tube guidance. **defense response** to fungus, **embryo development ending in seed dormancy, pollen tube guidance.** |  |
|  |  |
|  |  |

| **OTHER** | |
| --- | --- |
| **INDUCED** | **REPRESSED** |
| **Agenet domain-**containing protein | **A20/AN1-like zinc finger** family protein |
| **N-acetylglucosaminylphosphatidylinositol de-N-acetylase** family protein | **AAR2 protein** family |
|  | **Similar to ASY2,** DNA binding [Arabidopsis thaliana] (TAIR:AT4G32200.1) |
|  |  |
|  | **Alpha/beta-Hydrolases** superfamily protein |
|  | **Alpha/beta-Hydrolases** superfamily protein |
|  | **Alpha/beta-Hydrolases** superfamily protein |
|  | **Alpha/beta-Hydrolases** superfamily protein |
|  |  |
| **ARM repeat** superfamily protein | **ARM repeat** superfamily protein |
| **ARM repeat** superfamily protein | **ARM repeat** superfamily protein |
| **ARM repeat** superfamily protein | **ARM repeat** superfamily protein |
|  | **ARM repeat** superfamily protein |
|  | **ARM repeat** superfamily protein |
|  | **ARM repeat** superfamily protein |
|  |  |
|  | **BTB and TAZ domain protein 3** |
|  | **BTB/POZ domain**-containing protein |
|  |  |
|  | **Bestrophin-like** protein |
|  |  |
|  | **BREVIS RADIX-like 1** |
|  | **BREVIS RADIX-like 3** |
|  |  |
| **CBS domain-containing protein with a domain of unknown function (DUF21)** | **Calcineurin-like metallo-phosphoesterase** superfamily protein |
|  | **Calcineurin-like metallo-phosphoesterase** superfamily protein |
|  | **Cornichon** family protein |
|  | **Cox19-like CHCH family** protein (TAIR:AT5G09570.1) |
|  | **Cryptdin protein-related** |
|  | **CTC-interacting domain 8** |
|  |  |
| **DCD (Development and Cell Death) domain** protein |  |
| **DDT domain-containing** protein |  |
| **Downregulated in DIF1 18** |  |
| Domains rearranged methyltransferase 2 |  |
|  |  |
| **Emsy N Terminus (ENT) domain**-containing protein  **RING/FYVE/PHD zinc finger** superfamily protein | Terminal **EAR1-like 2** |
|  | **ELMO/CED-12** family protein |
|  | **Embryo defective 1273** |
|  | **ENTH/VHS/GAT** family protein |
|  | **Evolutionarily conserved C-terminal region 11** |
|  | **EXS (ERD1/XPR1/SYG1)** family protein |
|  |  |
| **F-box** family protein | **F-box** and associated interaction domains-containing protein |
|  | **F-box** and associated interaction domains-containing protein |
|  | **F-box** and associated interaction domains-containing protein |
|  | **F-box** family protein |
|  | **F-box** family protein |
|  | **F-box** family protein |
|  | **F-box** family protein |
|  | **F-box** family protein |
|  | **F-box** family protein |
|  | **F-box** family protein |
|  |  |
| **FBD-like domain** family protein | **FBD / Leucine Rich Repeat** domains containing protein |
|  | **FK506-binding protein 12** |
|  |  |
| **Gamete-expressed 3** |  |
|  |  |
| **Galactose oxidase/kelch repeat** superfamily protein | **Galactose oxidase/kelch repeat** superfamily protein |
| **Galactose oxidase/kelch repeat** superfamily protein |  |
|  |  |
|  | **hAT transposon** superfamily protein |
|  | **Histone superfamily** protein |
|  |  |
| **Integral membrane protein hemolysin-III homolog** | **Integral membrane HRF1** family protein |
|  |  |
| **Encodes JASON**.  *jason* mutant produces diploid male gametes leading to triploid progeny. Diploid gametes in the jason mutant are generated by a defect in male meiosis II. |  |
|  |  |
| **LisH and RanBPM domains** containing protein |  |
|  |  |
|  | **LOB domain-containing protein 23** |
|  | **LOB domain-containing protein 24** |
|  | **LOB domain-containing protein 28** |
|  | **LOB domain-containing protein 33** |
|  |  |
| **Lung seven transmembrane receptor** family protein |  |
|  |  |
| **Membrane-associated progesterone binding protein 4** | **MAK10 homologue** |
|  | **MD-2-related lipid recognition domain**-containing protein |
|  |  |
| **Nudix hydrolase homolog 8** |  |
|  |  |
| **Pentatricopeptide repeat (PPR)** superfamily protein | **Pentatricopeptide repeat (PPR)** superfamily protein |
| **Pentatricopeptide repeat (PPR)** superfamily protein | **Pentatricopeptide repeat (PPR)** superfamily protein |
|  | **Pentatricopeptide repeat (PPR)** superfamily protein |
|  | **Pentatricopeptide repeat (PPR)** superfamily protein |
|  | **Pentatricopeptide repeat (PPR)** superfamily protein |
|  | **Pentatricopeptide repeat (PPR)** superfamily protein |
|  | **Pentatricopeptide repeat (PPR-like)** superfamily protein |
|  |  |
| **PHD finger family protein / SWIB complex BAF60b domain**-containing protein / GYF domain-containing protein  **Transducin/WD40 repeat-like** superfamily protein | **Phosphorylase** superfamily protein |
| **Proteophosphoglycan-related** | **Pleckstrin homology (PH) domain** superfamily protein |
| **Putative / PSI-N**, putative (PSAN) | **Peptidoglycan-binding LysM domain**-containing protein |
| **Pumilio 20** |  |
|  |  |
| **Receptor homology region transmembrane domain ring H2 motif protein 1** | **Receptor like protein 46** |
| **RING 1A** | Rhodanese-like domain-containing protein / **PPIC-type PPIASE domain-containing protein.** |
|  | **Rubber elongation factor** protein (REF) |
|  |  |
| **SET domain protein 14** (TAIR:AT3G61740.1) |  |
| **Seed gene 3** |  |
|  |  |
|  | **T-complex protein 11** |
|  |  |
| **Tetratricopeptide repeat (TPR)-like** superfamily protein | **Tetratricopeptide repeat (TPR)-like** superfamily protein |
|  | **Tetratricopeptide repeat (TPR)-like** superfamily protein |
|  | **Tetratricopeptide repeat (TPR)-like** superfamily protein |
|  | **Tetratricopeptide repeat (TPR)-like** superfamily protein |
|  |  |
| **TRAF-like** superfamily protein | **TRAF-like family** protein; Involved in: **response to salt stress;** Located in: **vacuole, chloroplast envelope.** |
|  | **TRAF-like** family protein |
|  | **TRAF-like** superfamily protein |
|  | **TRAF-like** family protein |
|  | **TRAF-like** family protein |
|  | **TRAF-like** family protein |
|  |  |
| **Uncoupling protein 2** |  |
|  |  |
| **Vacuolar calcium-binding protein**-related |  |
|  |  |
| **VQ motif-containing** protein | **VQ motif-containing** protein |
| **VQ motif-containing** protein |  |
|  |  |
|  | **WD-40 repeat family protein** / zfwd4 protein (ZFWD4) |
|  |  |
| **CHY-type/CTCHY-type/RING-type zinc finger** protein | **CSL zinc finger domain**-containing protein |
| **Zinc finger (C3HC4-type RING finger)** family protein | **Zinc finger (C3HC4-type RING finger)** family protein |
| **HCP-like superfamily** protein with MYND-type zinc finger |  |
|  |  |

| **SIGNAL TRANSDUCTION** | |
| --- | --- |
| **PROTEIN KINASES SUPERFAMILY** | |
| **INDUCED** | **REPRESSED** |
| **Protein kinase** superfamily protein | **Protein kinase** superfamily protein |
| **Protein kinase** superfamily protein | **Protein kinase** superfamily protein |
| **Protein kinase** superfamily protein | **Protein kinase** superfamily protein |
| **Protein kinase** superfamily protein | **Protein kinase** protein with **tetratricopeptide repeat** domain |
| **Protein kinase** superfamily protein | **Protein kinase** superfamily protein |
| **Protein kinase** superfamily protein | **Protein kinase** superfamily protein |
| **Protein kinase** superfamily protein | **Protein kinase** superfamily protein |
| **Protein kinase** superfamily protein | **Protein kinase** superfamily protein |
|  | **Protein kinase** superfamily protein |
|  | **Protein kinase** superfamily protein |
|  | **Protein kinase** superfamily protein |
|  | **Protein kinase** superfamily protein |
|  |  |
|  | **Protein kinase**-related |
|  | **Protein kinase**-related |
|  |  |
| **MAPKKK-MAPKK-MAPK** | |
| **INDUCED** | **REPRESSED** |
| **Mitogen-activated protein kinase kinase kinase 15** | **MAP kinase 9** |
|  | **MAP kinase 19** |
|  | **MAP kinase 11** |
|  | **Mitogen-activated protein kinase homolog 2** |
|  |  |
| **CALCIUM-DEPENDENT KINASES AND OTHER CA-ASSOCIATED PROTEINS** | |
| **INDUCED** | **REPRESSED** |
| **Calcium-dependent protein kinase 1** | **Calcium-binding EF hand** family protein |
| **Calcium-dependent protein kinase 22** | **Calcium-binding EF-hand** family protein |
| **EF hand calcium-binding** protein family | **Calcium-dependent protein kinase 32** |
| **Calcium-binding EF-hand** family protein | **Calcium-binding EF hand** family protein |
|  | **Calcium-dependent protein kinase 28** |
|  | **Calcium-dependent lipid-binding (CaLB domain)** family protein |
|  | **Calcium-binding EF hand** family protein |
|  | **Calcium-binding EF hand** family protein |
|  | **Calcium-binding EF hand** family protein |
|  | **EF hand calcium-binding** protein family |
|  |  |
| C**ALMODULIN, CALMODULIN-LIKE, CALMODULIN-BINDING PROTEINS** | |
| **INDUCED** | **REPRESSED** |
| **Calmodulin like 43** | **Calmodulin-binding** protein |
| **Calmodulin binding** | **Calmodulin-binding** family protein |
| **Calmodulin-binding** family protein |  |
|  | Signal responsive 1; **CALMODULIN-BINDING TRANSCRIPTION ACTIVATOR** 3, CAMTA3, SIGNAL RESPONSIVE 1, SR1, **Encodes a putative CAM binding transcription factor. Loss of function mutations show enhanced resistance to fungal and bacterial pathogens suggesting that CAMTA functions to suppress defense responses.** Defense response to bacterium, defense response to fungus, leaf senescence, positive regulation of transcription from RNA polymerase II promoter, regulation of transcription, DNA-templated, **response to cold.** |
|  |  |
| **OTHER KINASES** | |
| **INDUCED** | **REPRESSED** |
| **Mad3/BUB1 homology region 1**; CONTAINS InterPro DOMAIN/s: **Mitotic checkpoint serine/threonine protein kinase**, Bub1 | **CDC2C; protein serine/threonine kinase** activity |
| **Protein kinase protein with adenine nucleotide alpha hydrolases-like** domain; **response to stress.** | **Protein kinases similar to PID (WAG1 and WAG2**) were  found to be root-tip expressed protein kinases **that negatively**  **regulate a root-waving response.** |
| **Casein kinase II beta chain 1**; Forms a tetrameric complex with CKA1 (CKA1(2)CKB1(2)). **One known substrate of CK2 is Phytochrome Interacting Factor 1 (PIF1).** CK2-mediated phosphorylation **enhances the light-induced degradation of PIF1 to promote photomorphogenesis.** | TOPP4, **TYPE ONE SERINE/THREONINE PROTEIN PHOSPHATASE 4** |
|  | ATNEK1, **NEK1, NIMA-RELATED SERINE/THREONINE KINASE 1**; Encodes AtNEK1, a member of the NIMA-related serine/threonine kinases (Neks) that have been linked to cell-cycle regulation in fungi and mammals. **Plant Neks might be involved in plant development processes.** |
|  | **Wall associated kinase 3** |
|  | **PfkB-like carbohydrate kinase** family protein |
|  | **Shaggy-like kinase 42**; ATSK42, SHAGGY-LIKE KINASE 42, SK42; **hyperosmotic response**, protein phosphorylation, **response to salt stress.** |
|  | **ACT-like protein tyrosine kinase** family protein |
|  | **ATP binding; protein kinases**;protein serine/ threonine kinases |
|  |  |
| **LECTIN, LECTIN-KINASES, RECEPTOR-LIKE LECTINS** | |
| **INDUCED** | **REPRESSED** |
| **Mannose-binding lectin** superfamily protein; glucosinolate | **S-locus lectin protein kinase** family protein |
| **Mannose-binding lectin** superfamily protein; **jacalin-related lectin 23.** | Mannose-binding lectin superfamily protein |
| **Concanavalin A-like lectin** family protein | **Mannose-binding lectin** superfamily protein |
|  | **Jacalin-related lectin 23** |
|  | **S-locus lectin protein kinase** family protein |
|  |  |
| **OTHER DISEASE-RELATED RECEPTORS AND RECEPTOR KINASES** | |
| **INDUCED** | **REPRESSED** |
| **Interleukin-1 receptor-associated kinase 4** protein | **Receptor-like protein kinase-**related |
| **Toll-Interleukin-Resistance (TIR)** domain family protein | **Disease resistance protein (TIR-NBS-LRR class)** family |
| **Cysteine-rich RLK (RECEPTOR-like protein kinase) 19** |  |
| **Toll-Interleukin-Resistance (TIR)** domain family protein |  |
| **ATP binding ;GTP binding**; nucleotide binding; nucleoside-triphosphatases; **Disease resistance protein (CC-NBS-LRR class).** |  |
|  |  |
| **PHOSPHATASES** | |
| **INDUCED** | **REPRESSED** |
| **Phosphatidic acid phosphatase (PAP2**) family protein | **Phosphatidic acid phosphatase (PAP2)** family protein |
| **Phosphotyrosine protein phosphatases** superfamily protein | **Protein phosphatase 2A regulatory B** subunit family protein |
| **Protein phosphatase 2C** family protein | **Protein phosphatase 2A-2** |
| **Rhodanese/Cell cycle control phosphatase** superfamily protein | **Phospholipase C 2** |
| **Protein phosphatase 2C** family protein | **Lipid phosphate phosphatase 2** |
| **2-phosphoglycolate phosphatase 2** | **Phosphatidic acid phosphatase-related** / PAP2-related |
| **Phosphoinositide-specific phospholipase C** family protein | **Protein phosphatase 2C** family protein |
| **C-terminal domain phosphatase-like 4** | **Type one protein phosphatase 1** |
|  |  |
| **LEUCINE-RICH RECEPTOR KINASES** | |
| **INDUCED** | **REPRESSED** |
| **Leucine-rich receptor-like protein kinase** family protein | **Leucine-rich receptor-like protein kinase** family protein |
| **Leucine-rich repeat receptor-like protein kinase** family protein | **Leucine-rich repeat protein kinase** family protein |
| **Leucine-rich repeat receptor-like protein kinase** family protein | **Leucine-rich repeat protein kinase** family protein |
| **Leucine-rich receptor-like protein kinase** family protein | **Leucine-rich repeat protein kinase** family protein |
|  | **Leucine-rich repeat transmembrane protein kinase** |
|  |  |
| **PEPTIDE SIGNALING** | |
| **INDUCED** | **REPRESSED** |
| **Phytosulfokine 6 precursor** | **PEP1 receptor 2**; **Encodes PEPR2, a plasma membrane leucine-rich repeat receptor kinase functioning as a receptor for the Pep1 and Pep2 peptides**. Pep1 and Pep2 are amino acids that induce the transcription of **defense-related genes**. |
| Microsomal signal peptidase 12 kDa subunit (SPC12); FUNCTIONS IN: peptidase activity; INVOLVED IN: **signal peptide processing.** |  |
|  |  |
| **LIGHT-SIGNALLING, PHOTOTROPISM** | |
| **INDUCED** | **REPRESSED** |
| **Phototropic-responsive NPH3** family protein; FUNCTIONS IN: signal transducer activity; Involved in: **response to light stimulus**. | **Phototropic-responsive NPH3** family protein |
|  | **Phototropic-responsive NPH3** family protein |
|  | Phytochrome B |
|  | FAR1-related sequence 6; Involved in: **response to red or far red light.** |
|  | **Photolyase/ blue-light receptor 2** |
|  |  |
| **LIPID ASSOCIATED SIGNALING, METABOLISM** | |
| **INDUCED** | **REPRESSED** |
| **Arabidopsis phospholipase-like protein (PEARLI 4)** family | **TRAM, LAG1 and CLN8 (TLC) lipid-sensing domain** containing protein |
| **Phospholipase A2-beta** | Patched family protein; **ATNPC1-2, NIEMANN-PICK DISEASE TYPE C1-2;** Patched family protein; FUNCTIONS IN: hedgehog receptor activity; **regulation of sphingolipid biosynthetic process.** |
|  | **Lipase/ lipooxygenase, PLAT/LH2 family** protein |
|  | **Calcium-dependent lipid-binding (CaLB domain)** family protein |
|  | **Calcium-dependent lipid-binding (CaLB domain)** family protein |
|  | Sulfoquinovosyldiacylglycerol 2; cellular response to phosphate starvation, **glycolipid biosynthetic process, sulfolipid biosynthetic process.** |
|  | Serinc-domain containing serine and **sphingolipid biosynthesis protein.** |
|  |  |
| **G-PROTEIN DEPENDENT SIGNALING** | |
| **INDUCED** | **REPRESSED** |
| **ROP-interactive CRIB motif-containing protein 7**; encodes a member of a novel protein family that contains contain a CRIB (for Cdc42/Rac-interactive binding) motif required for their specific interaction with **GTP-bound Rop1 (plant-specific Rho GTPase).** | **GTP binding protein beta 1** |
| **AMP-dependent synthetase and ligase** family protein | **RHO-related protein from plants 10** |
| ATCRSH, **CA^2+^-ACTIVATED RELA/SPOT HOMOLOG**, CRSH; Ca2+-activated RelA/spot homolog (CRSH); Functions in: **GTP diphosphokinase activity**, calcium ion binding; Involved in: guanosine tetraphosphate metabolic process; Located in: chloroplast; **guanosine tetraphosphate metabolic process**, **response to starvation.** | **Prenylated RAB acceptor 1.G1** |
|  | **Zinc finger (Ran-binding)** family protein |
|  | **RAB geranylgeranyl transferase beta subunit 2** |
|  | **RHO guanyl-nucleotide exchange factor 1** |
|  | **RAB GTPase homolog A5E** |
|  |  |
| **SIGNALLING-OTHER** | |
| **INDUCED** | **REPRESSED** |
| **N-acetylglucosamine-1-phosphate uridylyltransferase 1**; GLCNA.UT1, GLCNAC1PUT1, N-ACETYLGLUCOSAMINE-1-PHOSPHATE URIDYLYLTRANSFERASE 1; Encodes a protein that functions as an N-acetylglucosamine-1-phosphate uridylyltransferase that catalyzes the formation of UDP-N-acetylglucosamine (UDP-GlcNAc). **This is an essential precursor for glycolipid and glycoprotein synthesis and is also used for regulatory protein modification in signaling pathways**. The enzyme can also catalyze the reverse reaction using both UDP-GlcNAc and the less common UDP-N-acetylgalactosamine as substrates. | **Hypoxia-responsive family protein** / zinc finger (**C3HC4-type RING finger**) family protein |
| **Sinapoylglucose 1** | **Uclacyanin** 3; response to stimulus; **Encodes blue copper-binding protein III** |
| ATPP2-A8**, PHLOEM PROTEIN 2-A8**, PP2-A8; phloem protein 2-A8 (PP2-A8); Functions in: carbohydrate binding; Involved in: signal transduction, **defense response**, **innate immune response** | ATPP2-B9, **PHLOEM PROTEIN 2-B9**, SKIP3, SKP1 INTERACTING PARTNER 3; encodes a protein containing an F-box domain and physically interacts with SCF subunit SKP1/ASK1. The protein also exhibits similarity in sequence to phloem protein 2 (PP2) from cucumber. |
| **CYTOCHROME P450, FAMILY 82, SUBFAMILY C, POLYPEPTIDE 4**", CYP82C4, The gene encodes a cytochrome P450 enzyme, CYP82C. **It is involved in the early Fe deficiency response**. The mRNA is cell-to-cell mobile. | **FBD / Leucine Rich Repeat** domains containing protein |
|  | **RING/FYVE/PHD zinc finger** superfamily protein |
|  |  |

| **ENERGY AND PHOTOSYNTHESIS** | |
| --- | --- |
| **NITROGEN-CARBOHYDRATE-LIPID CATABOLISM-GLYCOLISIS-KREBS CYCLE-PENTOSE PHOSPHATE PATHWAY** | |
| **INDUCED** | **REPRESSED** |
| **Fatty acid beta-oxidation** | **Cytochrome P450, family 77, subfamily A, polypeptide 4**; **Fatty acid oxidation** |
| **Phosphofructokinase 1** | **Phosphofructokinase** family protein |
| **Citrate synthase 2** Encodes a **peroxisomal citrate synthase** that is **expressed throughout seedling and shoot development** | **Pyruvate kinase** family protein |
| Pyruvate orthophosphate dikinase; PPDK, PYRUVATE ORTHOPHOSPHATE DIKINASE; Encodes a dual-targeted protein believed to act as a pyruvate, orthophosphate dikinase. These enzymes are normally associated with C4 photosynthesis which does not occur in Arabidopsis. **However, PPDK may play a role in remobilizing nitrogen during leaf senescence in Arabidopsis.** | **LIPOAMIDE DEHYDROGENASE 1**, LPD1, PTLPD1; encodes a plastid lipoamide dehydrogenase, subunit of the **pyruvate dehydrogenase complex which provides acetyl-CoA for de novo fatty acid biosynthesis.** The gene is highly expressed **in developing seeds**. |
| **Sucrose synthase 1** | **Phosphofructokinase** family protein |
| **ADP glucose pyrophosphorylase large subunit 1** | ACO3, **ACONITASE 3**; Encodes an aconitase that can catalyze the conversion of citrate to isocitrate through a cis-aconitate intermediate, indicating that it **may participate in the TCA cycle and other primary metabolic pathways**. Citrate metabolic process**, glyoxylate cycle**, isocitrate metabolic process, **response to abscisic acid**, response to cadmium ion, **response to oxidative stress, response to salt stress, tricarboxylic acid cycle.** |
|  | **Phosphoglucomutase/ phosphomannomutase** family protein |
|  | **Phosphomannomutase** |
|  | **6-phosphogluconate dehydrogenase** family protein |
|  | **Galactose mutarotase**-like superfamily protein; **probably functions to bind carbohydrates in enzymes that act on sugars.** |
|  |  |
| **ATP PRODUCTION_ATP-related ion pumping and metabolism** | |
| **INDUCED** | **REPRESSED** |
| **ATPase, V0 complex, subunit E** | **Vacuolar ATPase assembly integral membrane protein VMA21-like** domain |
| **Cytochrome C oxidase 6B** | **ATPase, AAA-type, CDC48** protein |
| **Cytochrome C oxidase polypeptide VIB** family protein | **P-type ATPase of Arabidopsis 2** |
| **Cytochrome b561/ferric reductase** transmembrane protein family | **Cytochrome c oxidase 19-1** |
|  | **Gamma subunit of Mt ATP synthase** |
|  | **ATPase, V0 complex, subunit E** |
|  | **ATPase, F0/V0 complex, subunit C** protein |
|  | **Cytochrome c oxidase 17** |
|  | **Electron transport SCO1/ SenC** family protein; embryo development, **mitochondrial respiratory chain complex IV assembly,** **respiratory chain complex IV assembly.** |
|  |  |
| **PHOTOSYNTHESIS, CALVIN CYCLE, PHOTORESPIRATION** | |
| **INDUCED** | **REPRESSED** |
| **Photosystem I reaction center subunit PSI-N**, chloroplast, | **Phosphoribulokinase** |
| **GLUTAMATE-1-SEMIALDEHYDE 2,1-AMINOMUTASE 2**, GSA2; glutamate-1-semialdehyde 2,1-aminomutase 2 (GSA2); Functions in: glutamate-1-semialdehyde 2,1-aminomutase activity, pyridoxal phosphate binding, transaminase activity, catalytic activity; Involved in: porphyrin biosynthetic process; LOCATED IN: chloroplast stroma, chloroplast, chloroplast envelop; **chlorophyll biosynthetic process**, **porphyrin-containing compound biosynthetic process, protoporphyrinogen IX biosynthetic process.** | **5-Formyltetrahydrofolate cycloligase** (5-CHO-THF cycloligase - AT5G13050.1) **regulates/influences under photorespiratory conditions the activity of another gene product, i.e. serine hydroxymethyltransferase (SHMT) due to accumulating amounts of 5-Formyltetrahydrofolate.** |
| **Glutamate: glyoxylate aminotransferase**; ALANINE-2-OXOGLUTARATE AMINOTRANSFERASE 1, AOAT1, GGAT1, GGT1, GLUTAMATE:GLYOXYLATE AMINOTRANSFERASE, GLUTAMATE:GLYOXYLATE AMINOTRANSFERASE 1; Identified by cloning the gene that corresponded to a purified protein having glyoxylate aminotransferase activity. **Localized to the peroxisome and thought to be involved in photorespiration/ metabolic salvage pathway. glycine biosynthetic process, photorespiration, response to hypoxia.** | **NADH-Ubiquinone/plastoquinone (complex I)** protein |
|  | **High chlorophyll fluorescence 153** |
|  | **Chlorophyll A/B-binding protein 2** |
|  | **SET domain-containing protein**; **Rubisco methyltransferase** family protein |
|  | **NADH-ubiquinone oxidoreductase B18 subunit**, putative |
|  | **PGR5-LIKE A**; Encodes PGRL1A, a transmembrane protein present in thylakoids. PGRL1A has a highly homologous isoform PGRL1B encoded by At4g11960. Plants lacking PGRL1 show perturbation of cyclic electron flow, similar to PGR5-deficient plants. PGRL1 and PGR5 interact physically and associate with PSI (photosystem I). The mRNA is cell-to-cell mobile. **Oxidation-reduction process, photosynthesis, photosynthetic electron transport in photosystem I.** |
|  | **2Fe-2S ferredoxin-like** superfamily protein |
|  | **2Fe-2S ferredoxin-like** superfamily protein |
|  |  |
| **CHLOROPLAST, MITOCHONDRIA, PEROXISOME BIOGENESIS** | |
| **INDUCED** | **REPRESSED** |
| **Chloroplast-localized ISCA-like protein**; **iron-sulfur cluster assembly**, protein maturation by iron-sulfur cluster transfer, **sulfur compound metabolic process.** | **Translocon at the inner envelope membrane of chloroplasts 110**; **Involved in protein import into the chloroplast and chloroplast biogenesis.** |
| **Plastid-lipid associated protein PAP** / **fibrillin** family protein | **SMAD/FHA domain-containing protein** ; Involved in: biological_process unknown; Located in: **chloroplast thylakoid membrane, chloroplast stroma.** |
|  |  |
|  | **Rubredoxin-like** superfamily protein |
|  |  |

| **REDOX** | |
| --- | --- |
| **INDUCED** | **REPRESSED** |
| **NAD(P)-binding Rossmann-fold** superfamily protein | **NAD(P)-binding Rossmann-fold** superfamily protein  tropinone reductase |
| **NAD(P)-binding Rossmann-fold** superfamily protein | **NAD(P)-binding Rossmann-fold** superfamily protein |
|  |  |
| **2-oxoglutarate (2OG) and Fe(II)-dependent oxygenase** superfamily protein | **2-oxoglutarate (2OG) and Fe(II)-dependent oxygenase** superfamily protein |
|  | **2-oxoglutarate (2OG) and Fe(II)-dependent oxygenase** superfamily protein |
|  | **2-oxoglutarate (2OG) and Fe(II)-dependent oxygenase** superfamily protein |
|  | **2-oxoglutarate (2OG) and Fe(II)-dependent oxygenase** superfamily protein |
|  |  |
| **Peroxidase superfamily** protein | **Acid phosphatase/vanadium-dependent haloperoxidase**-related protein |
| **Peroxidase superfamily** protein |  |
| **Peroxidase superfamily** protein |  |
|  |  |
|  |  |
| **Cytochrome P450, family 71, subfamily B, polypeptide 21** | **Cytochrome P450, family 705, subfamily A, polypeptide 19** |
| **Cytochrome P450, family 706, subfamily A, polypeptide 7** | **Cytochrome P450, family 94, subfamily D, polypeptide 1** |
| **Cytochrome P450, family 705, subfamily A, polypeptide 4** | **Cytochrome P450, family 94, subfamily D, polypeptide 2** |
|  | **Cytochrome P450, family 71, subfamily B, polypeptide 16** |
|  | **Cytochrome p450 72c1** |
|  |  |
| **Glutaredoxin** family protein | **Glutathione S-transferase zeta 1** |
| **Lactoylglutathione lyase family protein / glyoxalase I family protein**; carbohydrate metabolic process, **response to cadmium ion.** | **Lactoylglutathione lyase / glyoxalase I** family protein |
| **Metallo-hydrolase/ oxidoreductase superfamily protein; GLX2-2, GLY2, GLYOXALASE 2-2**, **glutathione biosynthetic process**, **methylglyoxal catabolic process to D-lactate via S-lactoyl-glutathione.** | **Glutaredoxin** family protein |
| **Glutathione S-transferase TAU 28**; ATGSTU28, GLUTATHIONE S-TRANSFERASE TAU 28, GSTU28, **Encodes glutathione transferase belonging to the tau class of GSTs**. **Glutathione metabolic process**, response to cadmium ion, **toxin catabolic process.** |  |
|  |  |
| **Selenoprotein, Rdx** type |  |
| **Selenium binding** |  |
|  |  |
| **Peroxin 14** | ***Arabidopsis thaliana* PEROXYGENASE 2** |
|  | **Peroxisomal NAD-malate dehydrogenase 2** |
|  | **Peroxisomal membrane 22 kDa** (Mpv17/PMP22) family protein |
|  |  |
|  | **Thioredoxin** superfamily protein |
|  | **Thioredoxin** family protein |
|  | **Thioredoxin** family protein |
|  |  |
|  | **FAD/NAD(P)-binding oxidoreductase** family protein |
|  | **FAD-linked oxidases** family protein |
|  | **Glutamate dehydrogenase** 2; cellular amino acid metabolic process, oxidation-reduction process, response to cadmium ion, **response to salt stress.** |
|  | **Oxidoreductase, 2OG-Fe(II) oxygenase** family protein |
|  | **NAD(P)-linked oxidoreductase** superfamily protein |
|  | **Pyridine nucleotide-disulphide oxidoreductase** family protein |
|  | **NAD(P)-linked oxidoreductase** superfamily protein |
|  | **Zinc-binding dehydrogenase** family protein |
|  | **Isopropylmalate dehydrogenase 3**; leucine biosynthetic process, oxidation-reduction process, **response to salt stress.** |
|  | **Methionine sulfoxide reductase B (**(MSRB1); Functions in: peptide-methionine-(S)-S-oxide reductase activity; Involved in: **response to oxidative stress.** |
|  | **NADP-malic enzyme 3** |
|  | **Zinc-binding alcohol dehydrogenase** family protein |
|  | **FMN-linked oxidoreductases** superfamily protein |
|  | **Metallo-hydrolase/oxidoreductase** superfamily protein |
|  |  |
|  | Expression of the gene is **downregulated in the presence of paraquat, an inducer of photoxidative stress.** |
|  |  |
|  | **Respiratory burst oxidase protein** F; ARABIDOPSIS THALIANA RESPIRATORY BURST OXIDASE HOMOLOG F, ATRBOH F, ATRBOHF, RBOH F, RBOHAP108, RBOHF, RESPIRATORY BURST OXIDASE PROTEIN F; Involved in **abscisic acid-activated signaling pathway**, carbohydrate homeostasis, **defense response** and **defense response by callose deposition. Also in** **ethylene-activated signaling pathway,** **hydrogen peroxide biosynthetic process, negative regulation of programmed cell death, osmo-sensory signaling pathway, oxidation-reduction process, reactive oxygen species metabolic process, regulation of stomatal movement, respiratory burst involved in defense response, response to ethylene.** |
|  |  |
|  | **Plant EC metallothionein-like protein, family 15** |
|  |  |
|  | **Peptidemethionine sulfoxide reductase 2**; ubiquitous enzyme that **repairs oxidatively damaged proteins.** |
|  |  |

| **DNA RNA TRANSCRIPTION TRANSLATION REPAIR RNA-SPLICING** | |
| --- | --- |
| **INDUCED** | **REPRESSED** |
| **P-loop containing nucleoside triphosphate hydrolases** superfamily protein | **P-loop containing nucleoside triphosphate hydrolases** superfamily protein |
| **P-loop containing nucleoside triphosphate hydrolases** superfamily protein | **P-loop containing nucleoside triphosphate hydrolases** superfamily protein |
| **P-loop containing nucleoside triphosphate hydrolases** superfamily protein | **P-loop containing nucleoside triphosphate hydrolases** superfamily protein |
|  | **P-loop containing nucleoside triphosphate hydrolases** superfamily protein |
|  | **P-loop containing nucleoside triphosphate hydrolases** |
|  |  |
| **DEAD box RNA helicase** family protein | **The helicase superfamily C-terminal domain**, associated  with DEXD-, DEAD- and DEAH-box proteins, is found in  a wide variety of helicases and helicase related proteins and it  is characterized by the presence of **a P-loop containing nucleoside triphosphate hydrolases.** The DEAD-box RNA helicase family comprise enzymes that participate in every aspect of RNA metabolism, **associated with a diverse range of cellular functions including response to abiotic stress.** |
| Putative mitochondrial **RNA helicase 1** | **RECQ helicase L4B** |
|  | **DEA(D/H)-box RNA helicase** family protein |
|  |  |
| **mRNA STABILITY/ SPLICING** | |
| **INDUCED** | **REPRESSED** |
| **PRP38 family** protein | **Arginine/serine-rich 45**; Encodes SR45, a member of the highly conserved family of serine/arginine-rich (SR) proteins, which play key roles in **pre-mRNA splicing and other aspects of RNA metabolism.** SR45 is a spliceosome protein, interacts with SR33 and the U1-70K protein of the U1 snRNP. **Also involved in plant sugar response.** |
| **PRP38** family protein; R**NA processing, RNA splicing, mRNA processing.** | **RNA-binding KH domain-containing** protein; **ENHANCED STRESS RESPONSE 1**, ESR1, HOS5, RCF3, REGULATOR OF CBF GENE EXPRESSION 3, SHI1, SHINY 1; Encodes a KH domain-containing putative RNA-binding protein RCF3. **Functions as a upstream regulator for heat stress-responsive gene expression and thermotolerance and mediation of jasmonate signalling**. Also shown to interact with HYL1 and is a component of the nuclear dicing body and to function in primary **miRNA processing. Heat acclimation, jasmonic acid mediated signaling pathway, primary miRNA processing, regulation of defense response to fungus, regulation of gene expression, response to osmotic stress.** |
| **HUA2 LIKE 3**, HULK3; HUA and HUA-LIKE (HULK) genes act redundantly to regulate a subset of essential genes, with some (or all) family members also having specific functions. The mRNA is cell-to-cell mobile. **Flower development, mRNA processing, regulation of transcription**, (**Tudor/PWWP/MBT domain-containing** protein). | **Mitochondrial RNAediting factor 1** |
| **31-KDA RNA BINDING PROTEIN**, ARABIDOPSIS THALIANA RNA BINDING PROTEIN, APPROXIMATELY 31 KD, ATRBP31, ATRBP33, CP31, RBP31; **Encodes a chloroplast RNA-binding protein**. A substrate of the type III effector HopU1 (mono-ADP-ribosyltransferase). **Required for editing and stability of specific chloroplast mRNAs.** RNA modification, RNA processing, RNA stabilization, base conversion or substitution editing, **cold acclimation, innate immune response, mRNA processing.** | APUM7, PUM7, **PUMILIO 7**; Encodes a member of the Arabidopsis Pumilio (APUM) proteins containing PUF domain (eight repeats of approximately 36 amino acids each). PUF proteins regulate both **mRNA stability and translation through sequence-specific binding to the 3' UTR of target mRNA transcripts.** |
| SWAP (**Suppressor-of-White-APricot**)/surp domain-containing protein / ubiquitin family protein; FUNCTIONS IN: **RNA binding; Involved in: RNA processing.** | **Spliceosome protein-related** |
| ATTLP2, TLP2, **TUBBY LIKE PROTEIN 2**; **regulation of transcription, DNA-templated**, response to fungus | **S1FA-like DNA-binding protein**; regulation of transcription; A cDNA encoding a specific binding activity for the tissue-specific negative cis-element S1F binding site of spinach rps1 was isolated from a spinach cDNA expression library. **This cDNA of 0.7 kb encodes an unusual small peptide of only 70 amino acids, with a basic domain which contains a nuclear localization signal and a putative DNA binding helix.** This protein, named S1Fa, is highly conserved between dicotyledonous and monocotyledonous plants. It may represent a novel class of DNA binding proteins. **The corresponding mRNA is accumulated more in roots and in etiolated seedlings than in green leaves.** This expression pattern is correlated with the tissue-specific function of the S1F binding site, which represses the rps1 promoter preferentially in roots and in etiolated plants. |
| APUM13, PUM13, **PUMILIO 13**; Encodes a member of the **Arabidopsis Pumilio (APUM) proteins containing PUF domain** (eight repeats of approximately 36 amino acids each). **PUF proteins regulate both mRNA stability and translation through sequence-specific binding to the 3' UTR of target mRNA transcripts** | **Poly(ADP-ribose) polymerase** |
| **Pseudouridine synthase** family protein; Functions in: pseudouridine synthase activity; Involved in: **pseudouridine synthesis, RNA modification** | **Ribosomal RNA processing 4** |
| **dsRNA-binding protein 5** | **RNase H domain-containing protein** [*Arabidopsis thaliana*] |
|  |  |
| **RNA BINDING** | |
| **INDUCED** | **REPRESSED** |
| **RNA-binding protein 45A** | **RNA polymerase III RPC4** |
| **RNA-binding KH domain-containing** protein | **RNA-binding (RRM/RBD/RNP motifs)** family protein |
| **RNA-binding (RRM/RBD/RNP motifs)** family protein | **RNA-binding protein 47C** |
| **RNA-binding (RRM/RBD/RNP motifs)** family protein; mRNA transport, **ALWAYS EARLY 4** | **RNA-binding (RRM/RBD/RNP motifs)** family protein |
| **RNA-binding (RRM/RBD/RNP motifs)** family protein | **RNA-binding (RRM/RBD/RNP motifs)** family protein |
| **RNA-binding** protein | **RNA-binding protein 47C'** |
| **RNA binding (RRM/RBD/RNP motifs)** family protein | **RNA-binding (RRM/RBD/RNP motifs)** family protein |
|  | **RNA binding** |
|  | **RNA-binding KH domain-containing** protein |
|  | **RNA-binding CRS1 / YhbY (CRM) domain** protein |
|  | **RNA-binding (RRM/RBD/RNP motifs)** family protein |
|  | **RNA-binding (RRM/RBD/RNP motifs)** family protein |
|  | **RNA-binding (RRM/RBD/RNP motifs)** family protein |
|  | **Chloroplast RNA-binding protein 29** |
|  | **Nuclear RNA polymerase C1** |
|  | **Glycine-rich RNA-binding protein 6** |
|  |  |
| **NUCLEASES** | |
| **INDUCED** | **REPRESSED** |
| **Adenine nucleotide alpha hydrolases-like** superfamily protein | **RNI-like superfamily protein; Leucine-rich repeat, ribonuclease inhibitor subtype** |
| **Ribonuclease II/R** family protein | **Restriction endonuclease, type II-like** superfamily protein |
| **Polynucleotidyl transferase, ribonuclease H-like** superfamily protein | **Exoribonuclease 4** |
|  | **Polynucleotidyl transferase, ribonuclease H-like** superfamily protein |
|  | **Polynucleotidyl transferase, ribonuclease H-like** superfamily protein |
|  | **Ribonuclease H-like superfamily** protein. |
|  | **Zinc ion binding; nucleic acid binding; hydrolases**, acting on acid anhydrides, in phosphorus-containing anhydrides |
|  | **Putative endonuclease** or glycosyl hydrolase |
|  |  |
|  |  |
| **TRANSCRIPTION-TRANSLATION** | |
| **INDUCED** | **REPRESSED** |
| **B-BOX DOMAIN PROTEIN 31**, BBX31; B-box type zinc finger family protein; Functions in: sequence-**specific DNA binding transcription factor activity**, zinc ion binding; Involved in: **response to UV-B, regulation of transcription** | **DNA directed RNA polymerase, 7 kDa subunit** |
| ***Arabidopsis thaliana* RHD SIX-LIKE 1**, ATRSL1, RHD SIX-LIKE 1, RSL1; RHD SIX-LIKE 1 (RSL1); FUNCTIONS IN: DNA binding, sequence-specific DNA binding transcription factor activity; Involved in: **regulation of transcription.** | **RNA-directed DNA polymerase (reverse transcriptase)-related** family protein |
| **tRNA synthetase class I** (I, L, M and V) family protein | **Polymerase/ histidinol phosphatase-like**; **DNA biosynthetic process, DNA replication.** |
| *Arabidopsis thaliana* **PROTEIN ARGININE METHYLTRANSFERASE 3**, ATPRMT3, PRMT3, PROTEIN ARGININE METHYLTRANSFERASE 3, metal ion binding, methyltransferase activity; REQUIRED FOR **ribosome biogenesis by affecting precursor ribosomal RNA processing.** | **TBP-associated factor 7**; Functions in: general **RNA polymerase II transcription factor activity**; Involved in: transcription initiation from RNA polymerase II promoter |
|  | UKL3, **URIDINE KINASE-LIKE 3**; biosynthetic process, **pyrimidine nucleobase metabolic process, pyrimidine nucleoside salvage.** |
|  | **SIN3-LIKE 4**, SNL4; Encodes a homolog of the **transcriptional repressor SIN3** (AT1G24190). |
|  | **RNA polymerase II transcription mediators** |
|  | **Transcription factor IIA, alpha/beta subunit** |
|  | Transcription activators |
|  | **RNA-directed DNA polymerase (reverse transcriptase)-related** family protein (TAIR:AT5G18880.1) |
|  | **CTP synthase** family protein; **DNA synthesis** |
|  | **RNA polymerase II transcription** **mediators** |
|  | **Nucleotidylyl transferase** superfamily protein |
|  | **L-Aspartase-like family protein**; purine r**ibonucleotide biosynthetic process**, **purine base biosynthetic process.** |
|  | **NEGATIVE ON TATA LESS2A**, NOT2A; NOT2 / NOT3 / NOT5 family; Functions in: **transcription regulator activity** |
|  | TAF11B, **TBP-ASSOCIATED FACTOR 11B**; Encodes TAF11b, a putative **TBP-associated factor (TBP: TATA binding protein).** |
|  | **Translation initiation factor 3 subunit H1** |
|  | **GTP binding Elongation factor Tu** family protein |
|  | **Glutamyl-tRNA reductase** family protein |
|  | **Ribosomal L18p/L5e** family protein |
|  | **60S acidic ribosomal** protein family |
|  | **Ribosomal protein L18ae** family |
|  | **MIF4G domain-containing protein**; Functions in: **translation initiation factor activity**; Involved in: translation, **RNA metabolic process.** |
|  |  |
| **CHROMATIN REMODELING-EPIGENETIC CHANGES-RNA SILENCING** | |
| **INDUCED** | **REPRESSED** |
| **Regulator of chromosome condensation (RCC1**) family protein | **Regulator of chromosome condensation (RCC1)** family protein |
| **Histone superfamily** protein | **MORF RELATED GENE 2**, MRG2; **chromatin remodeling, histone H4 acetylation**, **regulation of timing of transition from vegetative to reproductive phase.** |
| LDL2, **LSD1-LIKE2**, Encodes a homolog of human Lysine-Specific Demethylase1. **Involved in H3K4 methylation of target genes** including the flowering loci FLC and FWA. **Histone H3-K4 methylation,** oxidation-reduction process. | **SWI-SNF-related chromatin binding** protein |
| **Histone superfamily** protein | **RNA-DIRECTED DNA METHYLATION 1** |
| **Minichromosome maintenance (MCM2/3/5)** family protein; MCM7, PRL, PROLIFERA; Member of the minichromosome maintenance complex, involved in DNA replication initiation. **Abundant in proliferating and endocycling tissues**. Localized in the nucleus during G1, S and G2 phases of the cell cycle, and are released into the cytoplasmic compartment during mitosis. **Binds chromatin.** | **Histone-lysine N-methyltransferase ASHH3** |
| **Transcription regulatory protein SNF2**, putative; ARABIDOPSIS THALIANA BRAHMA, ATBRM, BRAHMA, BRM, CHA2, CHR2, CHROMATIN REMODELING 2; Encodes a SWI/SNF chromatin remodeling ATPase that upregulates transcription of all three CUC genes and is involved in the formation and/or maintenance of boundary cells during **embryogenesis**. Also mediates **repression of expression of seed storage proteins in vegetative tissues**. Interacts strongly with AtSWI3C, also with AtSWI3B, but not with AtSWI3A or AtSWI3D. **ATP-dependent chromatin remodeling, organ boundary specification between lateral organs and the meristem, regulation of gene expression, epigenetic, regulation of transcription.** | **SWIB/MDM2 domain superfamily protein**; BAF60, CHC1, SWP73B; **CHC1 is predicted to encode a protein that belongs to the chromodomain remodeling complex**; **DNA repair**, **regulation of flower development**, **regulation of gene expression, regulation of leaf development**, **response to UV-B**, **root development.** |
| **DNA methyltransferase 2**; C-5 methylation of cytosine, DNA mediated transformation, DNA methylation**, chromatin modification.** | Encodes a **HR demethylase that acts as a positive regulator of seed germination in the PHYB-PIL5-SOM pathway**.  **Cell surface receptor signaling pathway, histone H4-R3 methylation, positive regulation of seed germination.** |
| ATSWI3B, CHB2, **CHROMATIN REMODELING COMPLEX SUBUNIT B**, SWI3B, SWITCH SUBUNIT 3; Homologous to yeast SWI3 & RSC8, components of the SWI/SNF and RSC **chromatin remodeling complexes**. Interacts with BSH, AtSWI3A, SWI3C and FCA. Expressed ubiquitously. chromatin remodeling, **multicellular organismal development, regulation of transcription** | **Regulator of chromosome condensation (RCC1)** family protein  lipoamide dehydrogenase 1 (glycine decarboxylase). |
|  |  |
| **Argonaute family** protein | **PAZ domain-containing protein** / piwi domain-containing protein, **gene silencing by RNA.** |
|  |  |
|  | **Paired amphipathic helix (PAH2)** superfamily protein |
|  | **Paired amphipathic helix (PAH2)** superfamily protein |
|  | **Paired amphipathic helix (PAH2)** superfamily protein |
|  | **This entry represents the paired amphipathic helix (PAH) repeat. Sin3 proteins have at least three PAH domains (PAH1, PAH2, and PAH3).** **They are components of a co-repressor complex that silences transcription**, playing important roles in the transition between proliferation and differentiation. **Sin3 proteins are recruited to the DNA by various DNA-binding transcription factors such as the Mad family of repressors, Mnt/Rox, PLZF, MeCP2, p53, REST/NRSF, MNFbeta, Sp1, TGIF and Ume6 [**[**PMID: 11101889**](http://europepmc.org/abstract/MED/11101889)**]. Sin3 acts as a scaffold protein that in turn recruits histone-binding proteins RbAp46/RbAp48 and histone deacetylases HDAC1/HDAC2, which deacetylate the core histones resulting in a repressed state of the chromatin**. The PAH domains are protein-protein interaction domains through which Sin3 fulfils its role as a scaffold. The PAH2 domain of Sin3 can interact with a wide range of unrelated and structurally diverse transcription factors that bind using different interaction motifs. For example, the Sin3 PAH2 domain can interact with the unrelated Mad and HBP1 factors using alternative interaction motifs that involve binding in opposite helical orientations. |
|  |  |
| **DNA REPAIR-MEISOSIS** | |
| **INDUCED** | **REPRESSED** |
| ATMLH3, **MLH3, MUTL PROTEIN HOMOLOG 3**; **DNA mismatch repair protein similar to MutL**. Required for normal levels of meiotic crossovers. | **DNA glycosylase superfamily protein; DNA repair, base-excision repair.** |
| ATBRCA2(V), BRCA2(V), **BRCA2-LIKE B**, BRCA2B; Ortholog of breast cancer susceptibility protein 2. **Essential at meiosis**. Interacts with with both Rad51 and Dss1(I) or both Dmc1 and Dss1(I) in a tripartite complex. **Double-strand break repair via homologous recombination**, meiotic cell cycle. |  |
|  |  |
| **OTHER-DNA-RELATED** | |
| **INDUCED** | **REPRESSED** |
| **RNA 2'-phosphotransferase**, Tpt1 / KptA family | **Topoisomerase II-associated protein PAT1** |
| **F-box/RNI-like** superfamily protein | **F-box/RNI-like** superfamily protein |
| **F-box/RNI-like** superfamily protein | **F-box/RNI-like/FBD-like domains**-containing protein |
| **F-box/RNI-like** superfamily protein | **F-box/RNI-like/FBD-like domains**-containing protein |
| Protein with **RNI-like/FBD-like domains** |  |
|  |  |
| **Transposable_element**_gene | **Transposable_element**_gene |
|  | **Transposable_element**_gene |
|  | **F-box and associated interaction** domains-containing protein  contains InterPro domain. **Retrotransposon gag protein** |
|  | Contains InterPro domain. **Retrotransposon gag protein** |
|  |  |
| **Nucleic acid-binding, OB-fold-like** protein |  |
| **Nucleic acid-binding, OB-fold-like** protein FUNCTIONS IN: RNA binding, translation initiation factor activity |  |

| **miRNA** | |
| --- | --- |
| **INDUCED** | **REPRESSED** |
| MIR156F; miRNA; **MICRORNA156F, MIR156F**; Encodes a microRNA that targets several SPL family members, including SPL3,4, and 5. By regulating the expression of SPL3 (and probably also SPL4 and SPL5), this microRNA **regulates vegetative phase change. cellular response to phosphate starvation; leaf development, regulation of growth rate.** | **MIR395B; miRNA** |
| MICRORNA171A, **MIR171A; Encodes a microRNA that targets several SCL family members**. | **MIR393A; miRNA** |
|  | **MIR395E**; miRNA; **Encodes a microRNA that targets both APS and AST family members. Cellular response to sulfate starvation.** |
|  |  |
|  |  |

| **SECONDARY METABOLISM** | |
| --- | --- |
| **INDUCED** | **REPRESSED** |
| **S-adenosyl-L-methionine-dependent methyltransferases** superfamily protein | **S-adenosyl-L-methionine-dependent methyltransferases** superfamily protein |
|  | **S-adenosyl-L-methionine-dependent methyltransferases** superfamily protein |
|  | **S-adenosyl-L-methionine-dependent methyltransferases** superfamily protein |
|  | **S-adenosyl-L-methionine-dependent methyltransferases** superfamily protein |
|  | **S-adenosyl-L-methionine-dependent methyltransferases** superfamily protein |
|  | **S-adenosyl-L-methionine-dependent methyltransferases** superfamily protein |
|  | **S-adenosyl-L-methionine-dependent methyltransferases** superfamily protein |
|  | **S-adenosyl-L-methionine-dependent methyltransferases** superfamily protein |
|  | **S-adenosyl-L-methionine-dependent methyltransferases** |
|  | **S-adenosyl-L-methionine-dependent methyltransferases** superfamily protein |
|  |  |
| **Phospholipid N-methyltransferase;** methylation, **phosphatidylcholine biosynthetic process**, phospholipid biosynthetic process | **PHOSPHATIDYLSERINE DECARBOXYLASE 1**, PSD1; **phosphatidylethanolamine biosynthetic** process |
| **Acyl-CoA N-acyltransferases (NAT)** superfamily protein | **Fatty acid reductase 1** |
| ORP3C, **OSBP(OXYSTEROL BINDING PROTEIN)-RELATED PROTEIN 3C**; OSBP(oxysterol binding protein)-related protein 3C (ORP3C); FUNCTIONS IN: oxysterol binding; Involved in: **steroid metabolic process.** | **Acyl-activating enzyme 18** |
| acyl-CoA oxidases;oxidoreductases, acting on the CH-CH group of donors;FAD binding;oxidoreductases;acyl-CoA oxidases | **Fatty acid desaturase** family protein |
|  | **Phospholipid/glycerol acyltransferase** family protein |
|  | **Acyl-CoA sterol acyl transferase 1** |
|  | **Acyl-CoA N-acyltransferases (NAT)** superfamily protein |
|  | **Acyl-CoA N-acyltransferases (NAT)** superfamily protein |
|  | **Acyl-CoA N-acyltransferase** |
|  |  |
| **Camelliol C synthase 1** | **Terpenoid cyclases/Protein prenyltransferases** superfamily protein |
| **1-deoxy-D-xylulose 5-phosphate synthase 1** | **Protein prenylyltransferase** superfamily protein |
| **Terpenoid synthases** superfamily protein | **Mevalonate kinase** |
|  | **Terpene synthase 02** |
|  | **ERG28; homolog of yeast ergosterol28; sterol biosynthetic process** |
|  | **Protein prenylyltransferase** superfamily protein |
|  |  |
|  | **Carbamoyl phosphate synthetase B;** **arginine biosynthetic process,** cellular response to phosphate starvation. |
|  | branched-chain alpha-keto acid decarboxylase E1 beta subunit |
|  | **ACT domain-containing small subunit of acetolactate synthase protein**; **branched-chain amino acid biosynthetic process,** isoleucine biosynthetic process, valine biosynthetic process. |
|  | **Class-II DAHP synthetase family** protein; **aromatic amino acid family biosynthetic** process. |
|  | **Carbamoyl phosphate synthetase A**; **'de novo' UMP biosynthetic process, 'de novo' pyrimidine nucleobase biosynthetic proces**s, **arginine biosynthetic process, cellular response to phosphate starvation, glutamine metabolic** process. |
|  | **Isopropyl malate isomerase large subunit 1**; Encodes a methylthioalkylmalate isomerase involved in **glucosinolate biosynthesis. leucine biosynthetic process** |
|  | **VALINE-TOLERANT 1**; acetolactate synthase activity, amino acid binding; Involved in: **branched chain family amino acid biosynthetic process.** |
|  | **Cystathionine beta-synthase (CBS) protein; Met pathway regulation.** |
|  | **Ketol-acid reductoisomerase**; Ketol-acid reductoisomerases (KARI) catalyses two steps **in the biosynthesis of branched-chain amino acids.** |
|  |  |
| **Catabolic process, nitrile biosynthetic process** | **Flavin-monooxygenase glucosinolate S-oxygenase 2** |
|  | **Nitrilase/cyanide hydratase and apolipoprotein N-acyltransferase** family protein |
|  |  |
|  | **Thiamin pyrophosphokinase 2** |
|  | **Cobalamin biosynthesis CobW-like** protein |
|  | **Pyridoxamine 5'-phosphate oxidase** family protein |
|  | **ThiaminC** ; PY, PYRIMIDINE REQUIRING, THIAMINC, THIC; **Encodes a protein involved in thiamin biosynthesis**. |
|  | **Erythronate-4-phosphate dehydrogenase family protein**; **Pyridoxal-5'-phosphate is the active form of vitamin B6** and is an essential cofactor for many enzymes, particularly those involved **in amino acid metabolism.** |
|  |  |
|  | **Thioesterase superfamily** protein |
|  |  |
| **Protein N-terminal asparagine amidohydrolase** family protein; Protein N-terminal asparagine amidohydrolase (NTAN1) acts on the side-chain deamidation of N-terminal asparagine residues to aspartate. **It is required for the ubiquitin-dependent turnover of intracellular proteins that initiate with Met-Asn**. These proteins are acetylated on the retained initiator methionine and can subsequently be modified by the removal of N-acetyl methionine by acylaminoacid hydrolase (AAH). Conversion of the resulting N-terminal asparagine to aspartate by PNAD renders the protein susceptible to arginylation, polyubiquitination and degradation as specified **by the N-end rule.** NTAN1 does not act on substrates with internal or C-terminal asparagines and does not act on glutamine residues in any position. | **Amidase family** protein; **carbon-nitrogen ligase activity**, with glutamine as amido-N-donor |
| **Nicotinate/ nicotinamide mononucleotide adenyltransferase;** **Nicotinate/nicotinamide mononucleotide adenyltransferase-mediated regulation of NAD biosynthesis protects guard cells from reactive oxygen species in ABA-mediated stomatal movement in Arabidopsis.** | **N-terminal nucleophile aminohydrolases** (Ntn hydrolases) superfamily protein; PRESENT IN Class II glutamine amidotransferases. (**C: N METABOLISM**) |
| **Adenine nucleotide alpha hydrolases-like** superfamily protein | **Putative methyltransferase** family protein |
|  |  |
|  |  |

| **PROTEIN SYNTHESIS CHAPERONES** | |
| --- | --- |
| **INDUCED** | **REPRESSED** |
| **18S pre-ribosomal assembly protein gar2-related** | **Ribosomal protein L6** family protein |
| **Ribosomal protein L11 methyltransferase-related** | **Ribosomal protein L18** |
| **Ribosomal L18p/L5e** family protein | **Ribosomal protein L53**, mitocondrial. |
| **50S ribosomal protein-related** | **Ribosomal protein S24e** family protein |
| **Ribosomal protein L27** family protein | **Ribosomal protein L13e** family protein |
| **Ribosomal protein S13/S18** family | **60S acidic ribosomal** protein family |
| **Translation protein SH3-like** family protein | **40S RIBOSOMAL PROTEIN,** ATBBC1, BBC1, BREAST BASIC CONSERVED 1, RSU2; Encodes 60S ribosomal protein L13. Homolog of human breast basic conserved 1 (BBC1). R**esponse to cytokinin, ribosome biogenesis.** |
| **Ribosomal protein S8** family protein | **60S ribosomal protein L18A-1** |
| **Ribosomal protein S8e** family protein |  |
| **Ribosomal protein L34** |  |
| **Ribosomal protein S19e** family protein |  |
| **60S acidic ribosomal** protein family |  |
| **Ribosomal protein L18e/L15** superfamily protein |  |
| **Ribosomal protein L5** |  |
|  |  |
|  | **Ribosomal RNA adenine dimethylase** family protein |
|  | **Elongation factor P (EF-P**) family protein |
|  | **Aminoacyl-tRNA ligases**; nucleotide binding; ATP binding |
|  |  |
| **Chaperone DnaJ-domain** superfamily protein | **Heat shock protein 17.6A** |
| **Chaperone protein DNAJ-related** | **HSP20-like** chaperones superfamily protein |
| **GroES-like** family protein | **HSP20-like** chaperone |
|  | **HSP20-like** chaperones superfamily protein |
|  | **HSP20-like** chaperones superfamily protein |
|  | **Chaperone DnaJ-domain** superfamily protein |
|  | **Chaperone DnaJ-domain** superfamily protein |
|  | **Chaperone DnaJ-domain** superfamily protein |
|  | **Chaperone DnaJ-domain** superfamily protein |
|  | **Chaperone DnaJ-domain** superfamily protein |
|  | **DnaJ heat shock** family protein |
|  | **Chaperonin 10** |
|  | **Chaperonin-60 alpha** |
|  | **TCP-1/cpn60 chaperonin** family protein |
|  | **TCP-1/cpn60 chaperonin** family protein |
|  | GroES-like zinc-binding dehydrogenase family protein |
|  | **APR-LIKE 5**, APRL5, ATAPRL5**; Encodes a protein disulfide isomerase-like (PDIL) protein**, a member of a multigene family within the thioredoxin (TRX) superfamily. |
|  | **Cyclophilin-like peptidyl-prolyl cis-trans isomerase** family protein; accelerating protein folding by **catalysing the cis-trans isomerisation of proline imidic peptide bonds in oligopeptides**. |
|  |  |
|  |  |

| **PROTEOLYSIS** | |
| --- | --- |
| **INDUCED** | **REPRESSED** |
| **RING/U-box** superfamily protein | **RING/U-box** superfamily protein |
| **RING/U-box** superfamily protein | **RING-H2 finger A1A**; RHA1A, RING-H2 FINGER A1A |
| **RING/U-box** superfamily protein | **RING/U-box** superfamily protein |
| **RING/U-box** superfamily protein | **RING/U-box** superfamily protein |
| **RING/U-box** superfamily protein | **RING/U-box** superfamily protein |
| **RING/U-box** superfamily protein | **RING/U-box** superfamily protein |
|  | **RING/U-box** superfamily protein |
|  | **RING/U-box** superfamily protein |
|  | **RING/U-box** superfamily protein |
|  | **RING/U-box** superfamily protein |
|  | **RING/U-box** superfamily protein |
|  | **RING/U-box** superfamily protein with **ARM repeat domain** |
|  | **RING/U-box** superfamily protein |
|  | **RING/U-box** superfamily protein |
|  |  |
| **F-box associated ubiquitination effector** family protein | **Ubiquitin-specific protease family C19**-related protein |
| **Ubiquitin-like** superfamily protein | **Ubiquitin-conjugating enzyme 6** |
| **Protein / ubiquitin** family protein | **Ubiquitin-like** superfamily protein |
| **F-box associated ubiquitination effector** family protein | **F-box associated ubiquitination effector** family protein |
| **BTB/POZ domain**-containing protein; **protein ubiquitination** | **F-box associated ubiquitination effector** family protein |
| **REGULATORY PARTICLE TRIPLE-A ATPASE 4A**, RPT4A; 26S proteasome AAA-ATPase subunit RPT4a (RPT4a) mRNA, ER-associated ubiquitin-dependent protein catabolic process, positive regulation of RNA polymerase II transcriptional preinitiation complex assembly, **ubiquitin-dependent protein catabolic process.** | **Cullin family protein**; Functions in: ubiquitin protein ligase binding; Involved in: **ubiquitin-dependent protein catabolic process.** |
| **Peptidase C78, ubiquitin fold modifier-specific peptidase 1/ 2** | **Ubiquitin-like** superfamily protein |
|  | **Ubiquitin-like** superfamily protein |
|  |  |
| **Subtilisin-like serine endopeptidase** family protein | **RHOMBOID-like 1** |
|  | **DegP protease 3** |
|  | **Eukaryotic aspartyl protease** family protein |
|  |  |
| **Methionine aminopeptidase 2A**; Encodes a MAP2 like methionine aminopeptidase. In MAP1A mutant background plants show an increased sensitivity t**o fumagillin resulting in defects in development.** |  |
|  |  |
| **Prolyl oligopeptidase** family protein |  |
| Protease-related, ATDEG15, DEG15, **DEGRADATION OF PERIPLASMIC PROTEINS 15, Mutants in this gene are defective in the processing of pre-glyoxysomal malate dehydrogenase.** |  |
|  |  |
| Similar to **Ulp1 protease family protein** [Arabidopsis thaliana] | Similar to **Ulp1 protease family** protein [Arabidopsis thaliana]; **ubiquitin-like protein SUMO** |
| **Ulp1 protease** family protein |  |
|  |  |
| **Serine carboxypeptidase-like 9**; sinapoylglucose 1 | **Serine carboxypeptidase-like 15** |
|  |  |
|  | **Cysteine proteinases superfamily** protein |
|  | **Cysteine proteinases superfamily** protein |
|  | Similar to **cysteine-type peptidase** [Arabidopsis thaliana] |
|  | **Cysteine proteinases** superfamily protein |
|  | Similar to **Ulp1 protease family** protein [Arabidopsis thaliana] similar to **cysteine-type peptidase** [Arabidopsis thaliana]; similar to Ulp1 protease family protein |
|  |  |
| This finding suggests that these two proteases differ in the balance of their activities, with **ULP1 predominantly showing C-terminal endopeptidase activity And ULP2 acting mainly as an isopeptidase, so that ulp1**  **suppresses ulp2 by reducing the abundance of mature SUMO**  **available to become incorporated into conjugates**. Our data  suggest **that ESD4** may play a role similar to that of **ULP2 in**  **regulating that abundance of SUMO conjugates.** |  |
|  |  |
|  |  |
