# Supplementary material for: Overexpression of Grain Amaranth (Amaranthus hypochondriacus) AhERF or AhDOF Transcription Factors in Arabidopsis thaliana Increases Water Deficit- and Salt-Stress Tolerance, Respectively, via Contrasting Stress-Amelioration Mechanisms
Source: PLoS One. 2016 Oct 17;11(10):e0164280. doi: 10.1371/journal.pone.0164280 (PMC5066980; doi:10.1371/journal.pone.0164280)
Supplement: S6 Table — (DOCX) [file pone.0164280.s012.docx]

**S6 Table**. **GO categories found to be significantly modified in *AhERF-VII* overexpressing transgenic Arabidopsis plants in optimal conditions, under water-deficit stress (WS), or in recovery, after stress (R).**

| **ERF OPTIMAL (+)^1^** | **ERF OPTIMAL (-)^2^** | **ERF WS (+)** | **ERF WS(-)** | **ERF R (+)** | **ERF R (-)** |
| --- | --- | --- | --- | --- | --- |
|  | **ribonucleoside monophosphate biosynthetic process, ribonucleoside monophosphate metabolic process, nucleoside monophosphate biosynthetic process, nucleoside monophosphate metabolic process, ribonucleotide biosynthetic process, nucleotide biosynthetic process,, ribonucleotide metabolic process, deoxyribonucleotide metabolic process,** | **nucleobase biosynthetic process, nucleobase metabolic process, nucleobase-containing compound biosynthetic process, nucleobase-containing small molecule biosynthetic process** | **pyrimidine ribonucleoside monophosphate biosynthetic process, UMP biosynthetic process, 'de novo' pyrimidine nucleobase biosynthetic process, UMP metabolic process, pyrimidine nucleobase biosynthetic process, pyrimidine ribonucleoside monophosphate metabolic process, nucleobase biosynthetic process, pyrimidine nucleoside monophosphate biosynthetic process, ribonucleoside monophosphate biosynthetic process, nucleobase metabolic process, pyrimidine nucleoside monophosphate metabolic process, pyrimidine nucleobase metabolic process, ribonucleoside monophosphate metabolic process, nucleobase-containing compound biosynthetic process, nucleobase-containing small molecule biosynthetic process, nucleoside metabolic process, nucleoside monophosphate biosynthetic process, nucleoside monophosphate metabolic process, ribose phosphate diphosphokinase activity** |  | **UMP metabolic process, pyrimidine ribonucleoside monophosphate biosynthetic process, UMP biosynthetic process,'de novo' pyrimidine nucleobase biosynthetic process, pyrimidine ribonucleoside monophosphate metabolic process, pyrimidine nucleobase biosynthetic process, pseudouridine synthesis, pyrimidine nucleoside monophosphate biosynthetic process, pyrimidine ribonucleotide metabolic process, pyrimidine nucleoside monophosphate metabolic process, pyrimidine nucleobase metabolic process, pyrimidine nucleotide biosynthetic process, pyrimidine nucleotide metabolic process, pseudouridine synthase activity** |
|  |  |  |  |  |  |
|  |  | **purine nucleobase biosynthetic process, purine nucleobase metabolic process** |  |  |  |
|  |  | **biological adhesion, cell adhesion** |  |  |  |
|  |  | **phloem or xylem histogenesis, xylem development** |  |  |  |
|  |  | **cell wall organization, cell wall modification** |  |  |  |
|  |  | **pectinesterase activity** |  |  |  |
|  | **polysaccharide biosynthetic process, polysaccharide metabolic process** |  | **cell wall macromolecule catabolic process** |  |  |
|  | **secondary cell wall biogenesis** |  | **mannosyltransferase activity** |  |  |
|  | **cellulose metabolic process** |  |  |  |  |
|  | **cell wall biogenesis, cellular cell wall organization or biogenesis, cell wall organization or biogenesis** |  |  |  |  |
|  | **cell wall polysaccharide metabolic process, cellular polysaccharide biosynthetic process** |  |  |  |  |
|  | **cellulose synthase (UDP-forming) activity** |  |  |  |  |
|  | **cytoskeleton organization** |  |  |  |  |
| **actin nucleation, actin cytoskeleton organization, actin filament-based process, actin filament organization** |  | **actin nucleation** |  |  |  |
|  |  |  |  |  |  |
| **trichome morphogenesis, trichome differentiation hair cell differentiation** |  |  |  | **post-embryonic organ morphogenesis, cellular component morphogenesis, cellular developmental process, cell morphogenesis, cell differentiation, cell development, cell morphogenesis involved in differentiation growth, developmental growth involved in morphogenesis, unidimensional cell growth, developmental maturation, regulation of cell size** |  |
| **lateral root development, post-embryonic root development** |  |  |  |  |  |
| **longitudinal axis specification, organelle localization,** |  |  |  |  |  |
| **epidermal cell differentiation, ectoderm development, epidermis development, cell morphogenesis,** |  |  |  |  |  |
| **cell morphogenesis involved in differentiation, positive regulation of cell proliferation** | **endoplasmic reticulum tubular network organization, organelle assembly, establishment of localization, positive regulation of multicellular organismal process** |  |  |  |  |
|  |  | **multicellular organismal process, system development, organ development, multicellular organismal development, Golgi organization** |  | **cellular component organization, cellular component assembly** |  |
|  |  |  |  |  | **secondary shoot formation, shoot formation** |
| **plastid membrane organization, thylakoid membrane organization, cellular membrane organization, membrane organization,** | **cellular macromolecular complex assembly, cellular component assembly, cellular macromolecular complex subunit organization, macromolecular complex assembly, macromolecular complex subunit organization,** |  |  | **reproductive process, maintenance of inflorescence meristem identity, pollen sperm cell differentiation, developmental process involved in reproduction, floral organ morphogenesis, pollen maturation, tube development, pollen tube development, floral organ formation, floral meristem determinacy** |  |
|  | **cellular component biogenesis,** |  |  |  |  |
| **cellular component morphogenesis, cellular component organization** |  |  |  |  |  |
| **leaf senescence, senescence, organ senescence** | **positive regulation of seed germination** | **Senescence, organ senescence** |  |  |  |
| **ovule development, embryonic axis specification,** |  |  | **maintenance of inflorescence meristem identity** |  |  |
| **Cytokinesis, cytokinesis by cell plate formation, cell division, cell cycle cytokinesis, cell cycle process** |  |  |  |  | **phragmoplast microtubule organization** |
| **plastid organization, establishment of plastid localization, chloroplast relocation, plastid localization** |  |  |  |  |  |
| **establishment of organelle localization** |  |  |  |  |  |
|  |  |  |  |  |  |
| **gene silencing, gene silencing by RNA, posttranscriptional gene silencing by RNA, posttranscriptional gene silencing** | **chromatin assembly** |  |  |  |  |
| **regulation of gene expression, epigenetic** | **RNA methylation** |  |  |  |  |
| **covalent chromatin modification** | **7-methylguanosine RNA capping** |  |  |  |  |
|  |  |  |  |  |  |
| **negative regulation of DNA metabolic process** |  |  |  |  |  |
| **DNA unwinding involved in replication, DNA duplex unwinding** |  |  |  |  |  |
| **DNA methylation, DNA alkylation, DNA modification** |  |  |  |  |  |
| **somatic cell DNA recombination** | **DNA packaging** |  |  |  |  |
| **DNA geometric change** | **DNA conformation change** |  |  |  |  |
| **3'-5' exonuclease activity** | **nucleosome assembly, nucleosome organization** |  | **DNA photolyase activity** | **nuclease activity** | **ribonuclease H activity** |
|  | **spindle assembly, spindle organization** |  | **transcription regulator activity** | **mismatched DNA binding** | **spliceosomal complex assembly** |
|  | **protein-DNA complex assembly** |  | **sequence-specific DNA binding transcription factor activity** |  | **translation elongation factor activity** |
|  | **transcription regulator activity** |  | **spliceosomal complex assembly** |  |  |
|  | **sequence-specific DNA binding transcription factor activity** |  | **siRNA binding** |  |  |
|  |  |  |  | **translation repressor activity**  **translation regulator activity**  **structural constituent of ribosome** |  |
|  |  |  |  |  |  |
|  |  |  |  |  | **diacylglycerol O-acyltransferase activity, acylglycerol O-acyltransferase activity, transferase activity, transferring acyl groups** |
| **cellular aromatic compound metabolic process, aromatic compound biosynthetic process** |  |  |  | **flavone biosynthetic process, flavone metabolic process, flavonol metabolic process, flavonol biosynthetic process** |  |
| **phenylpropanoid metabolic process** |  |  |  | **cytokinin biosynthetic process, cytokinin metabolic process** |  |
|  |  |  |  | **primary amine oxidase activity** |  |
|  |  |  |  | **chitinase activity** |  |
|  |  |  |  | **branched-chain amino acid metabolic process** |  |
|  | **primary metabolic process** |  |  |  |  |
|  | **nitrile biosynthetic process, nitrile metabolic process** |  | **nitrile metabolic process** |  |  |
|  | **vitamin biosynthetic process, water-soluble vitamin biosynthetic process, tetrahydrofolate metabolic process, vitamin B6 biosynthetic process, vitamin B6 metabolic process** |  | **pyridoxal phosphate metabolic process**  **pyridoxal phosphate biosynthetic process, vitamin B6 biosynthetic process, vitamin B6 metabolic process** |  |  |
|  | **pentacyclic triterpenoid biosynthetic process; pentacyclic triterpenoid metabolic process, triterpenoid biosynthetic process, triterpenoid metabolic** |  |  |  |  |
|  | **cellular carbohydrate biosynthetic process** |  |  |  |  |
|  |  |  |  |  |  |
| **small molecule metabolic process, small molecule catabolic process** |  |  |  |  |  |
| **protein acetylation** |  | **protein acetylation** |  |  |  |
| **protein acylation** |  |  |  |  |  |
|  |  |  |  |  |  |
| **starch biosynthetic process** |  |  |  |  |  |
| **monosaccharide metabolic process** |  |  |  |  |  |
|  |  |  |  |  |  |
|  |  |  | **fatty acid metabolic process, fatty acid biosynthetic process** |  | **fatty acid metabolic process**  **fatty acid beta-oxidation, fatty acid oxidation, fatty acid biosynthetic process, lipid oxidation, cellular lipid metabolic process** |
|  |  |  |  |  | **triglyceride biosynthetic process** |
|  | **glycerolipid biosynthetic process** | **regulation of carbohydrate biosynthetic process, monosaccharide metabolic process** |  |  |  |
|  |  | **cellular carbohydrate catabolic process** |  |  |  |
| **cellular modified amino acid metabolic process** |  |  |  |  |  |
| **maintenance of protein location** |  |  |  |  |  |
|  |  | **cellular protein modification process** |  |  |  |
| **fatty acid ligase activity, long-chain fatty acid-CoA ligase activity** |  |  |  |  |  |
| **fatty acid synthase activity** |  |  |  |  |  |
| **ligase activity, forming carbon-sulfur bonds** |  |  |  |  |  |
|  |  |  |  |  |  |
| **carbohydrate kinase activity, 6-phosphofructokinase activity, phosphofructokinase activity** |  | **6-phosphofructokinase activity, phosphofructokinase activity** |  |  |  |
|  |  |  |  |  |  |
|  | **transmembrane transport, ion transmembrane transport** |  |  |  | **intra-Golgi vesicle-mediated transport, transmembrane transport, Golgi vesicle transport, sugar transmembrane transporter activity, cation: sugar symporter activity, sugar :hydrogen symporter activity, solute: hydrogen symporter activity, solute: cation symporter activity, symporter activity, carbohydrate transmembrane transporter activity, secondary active transmembrane transporter activity** |
|  | **secretion by cell, secretion** |  |  |  |  |
|  | **transport**  **mitochondrial transport** |  |  |  |  |
|  | **protein secretion** |  |  |  |  |
|  | **oligopeptide transport, peptide transport** |  |  |  |  |
|  | **transporter activity, substrate-specific transporter activity, active transmembrane transporter activity, substrate-specific transmembrane transporter activity,, transmembrane transporter activity, carboxylic acid transmembrane transporter activity, organic acid transmembrane transporter activity, protein transmembrane transporter activity, cation transmembrane transporter activity, cation-transporting ATPase activity, metal ion transmembrane transporter activity, tricarboxylic acid transmembrane transporter activity, amino acid transmembrane transporter activity, protein transporter activity, amine transmembrane transporter activity, protein transmembrane transporter activity, purine nucleobase transmembrane transporter activity**  **GO:0015368 calcium: cation antiporter activity, macromolecule transmembrane transporter activity, transmembrane transporter activity, nucleobase transmembrane transporter activity** |  |  |  |  |
| **water transmembrane transporter activity, water channel activity** |  |  |  |  |  |
|  |  |  | **drug transmembrane transport, drug transport, acidic amino acid transmembrane transporter activity, active transmembrane transporter activity, carboxylic acid transmembrane transporter activity, amino acid transmembrane transporter activity, organic acid transmembrane transporter activity, amine transmembrane transporter activity, cationic amino acid transmembrane transporter activity** |  |  |
|  |  |  |  | **calcium-transporting ATPase activity, calcium ion transmembrane transporter activity, organic anion transmembrane transporter activity, protein transporter activity**  **intramolecular transferase activity,** |  |
|  |  | **cellular di-, tri-valent inorganic cation homeostasis** |  | **cellular di-, tri-valent inorganic cation homeostasis, cellular cation homeostasis, cellular iron ion homeostasis, cellular ion homeostasis, cation homeostasis, cellular chemical homeostasis** |  |
|  |  |  |  |  |  |
|  |  |  |  |  |  |
|  |  | **acting on acid anhydrides, catalyzing transmembrane movement of substances, ATPase activity, coupled to transmembrane movement of substances, ATPase activity, coupled to movement of substances, P-P-bond-hydrolysis-driven transmembrane transporter activity, primary active transmembrane transporter activity, active transmembrane transporter activity, ATPase activity, coupled** |  |  |  |
|  | **heme biosynthetic process, heme metabolic process** |  | **PSII associated light-harvesting complex II catabolic process** |  | **porphyrin-containing compound metabolic process, tetrapyrrole metabolic process** |
|  |  |  | **heme biosynthetic process, heme metabolic process** |  | **pigment catabolic process** |
| **hydrolase activity, acting on glycosyl bonds, hydrolase activity, hydrolyzing O-glycosyl compounds** |  | **positive regulation of transferase activity, hydrolase activity,** | **porphyrin-containing compound biosynthetic process** |  | **chlorophyll catabolic process** |
| **cyclase activity** |  | **response to arsenic-containing substance, arsenate reductase (glutaredoxin) activity** |  |  |  |
|  |  | **PSII associated light-harvesting complex II catabolic process** |  |  |  |
|  |  | **photosynthesis, light harvesting** |  |  |  |
| **DNA-dependent ATPase activity** | **proton-transporting two-sector ATPase complex assembly, proton-transporting ATPase activity, rotational mechanism, P-P-bond-hydrolysis-driven protein** |  |  | **ATP metabolic process, ATPase activity** | **CTP synthase activity,** |
|  |  |  |  |  |  |
|  | **proteolysis involved in cellular protein catabolic process, cellular protein catabolic process, protein catabolic process, proteolysis, ubiquitin-dependent protein catabolic process, modification-depend proteasomal protein catabolic process ent protein catabolic process, omega peptidase activity, proteasome binding,**  **aspartic-type peptidase activity, aspartic-type endopeptidase activity, aspartic-type peptidase activity, ubiquitin binding, aspartic-type endopeptidase activity, endopeptidase activity, peptidase activity, peptidase activity, acting on L-amino acid peptides, ubiquitin thiolesterase activity.** |  | **ubiquitin binding** |  | **glycoprotein catabolic process** |
|  | **modification-dependent macromolecule catabolic process, cellular macromolecule catabolic process, macromolecule catabolic process** | **cellular catabolic process, cellular macromolecule catabolic process, macromolecule catabolic process, catabolic process, aldehyde catabolic process** |  | **catabolic process, cellular catabolic process, cofactor catabolic process, coenzyme catabolic process, regulation of biological quality.** |  |
|  |  |  | **negative regulation of peptidase activity, regulation of peptidase activity, regulation of endopeptidase activity, regulation of proteolysis, negative regulation of endopeptidase activity, negative regulation of hydrolase activity, serine-type endopeptidase inhibitor activity** |  |  |
|  | **glycoside catabolic process** |  | **glycoside catabolic process** |  |  |
|  | **S-glycoside catabolic process, glycosinolate catabolic process, glucosinolate catabolic process** |  |  |  |  |
|  | **sulfur compound catabolic process** |  |  |  |  |
|  |  |  |  |  |  |
| **ARF guanyl-nucleotide exchange factor activity, guanyl-nucleotide exchange factor activity** |  |  |  |  |  |
| **GTPase binding** |  |  |  |  |  |
|  |  | **positive regulation of protein kinase activity, positive regulation of kinase activity** |  |  |  |
|  |  | **phosphotransferase activity, alcohol group as acceptor** |  |  |  |
|  |  | **protein kinase activity, transferase activity, transferring phosphorus-containing groups** |  |  |  |
|  |  | **calmodulin-dependent protein kinase activity** |  |  |  |
|  |  |  | **MAP kinase activity** |  |  |
|  |  |  | **diphosphotransferase activity** |  |  |
|  |  |  |  |  | **response to external stimulus** |
|  | **defense response to oomycetes** |  |  | **response to drug** |  |
| **response to nitric oxide** | **cellular response to nitric oxide** |  |  | **response to endogenous stimulus** |  |
|  |  |  |  |  |  |
|  |  |  |  | **response to drug** |  |
|  |  |  |  | **response to endogenous stimulus** |  |
| **response to symbiotic, response to symbiont** |  |  |  |  |  |
| **xenobiotic metabolic process, cellular response to xenobiotic stimulus** |  |  |  |  |  |
| **salicylic acid metabolic process, salicylic acid biosynthetic process** |  |  |  | **salicylic acid biosynthetic process** |  |
| **regulation of response to stress** |  |  |  |  |  |
| **cellular response to phosphate starvation** |  |  |  |  |  |
|  | **negative regulation of apoptotic process, regulation of apoptotic process, apoptotic process** |  |  |  |  |
|  |  | **methylglyoxal catabolic process to D-lactate, methylglyoxal catabolic process, methylglyoxal metabolic process** |  |  |  |
|  |  | **auxin conjugate metabolic process** |  |  |  |
|  |  | **response to monosaccharide stimulus, response to hexose stimulus** |  |  |  |
|  |  | **lactate metabolic process** |  |  |  |
|  | **small molecule metabolic process** |  |  |  |  |
|  | **ribonucleoside-diphosphate reductase activity, thioredoxin disulfide as acceptor, oxidoreductase activity, acting on CH or CH2 groups, disulfide as acceptor, oxidoreductase activity, acting on CH or CH2 groups, protein-disulfide reductase activity** | **oxidoreductase activity, acting on the CH-CH group of donors, oxygen as acceptor, oxidoreductase activity, acting on phosphorus or arsenic in donors, disulfide as acceptor, oxidoreductase activity, acting on phosphorus or arsenic in donors** | **acyl-CoA dehydrogenase activity, oxidoreductase activity, acting on the CH-CH group of donors** |  |  |
|  |  |  |  | **response to red light** | **red light signaling pathway,**  **cellular response to red light,**  **response to red light** |
|  | **'de novo' IMP biosynthetic process, IMP metabolic process, IMP biosynthetic process** |  | **inositol phosphate metabolic process, myo-inositol hexakisphosphate biosynthetic proce, myo-inositol hexakisphosphate metabolic process, inositol phosphate biosynthetic process, inositol biosynthetic process, inositol metabolic process,**  **inositol tetrakisphosphate kinase activity** |  |  |
|  |  |  |  |  | **hydrogen peroxide catabolic process, cellular response to hydrogen peroxide** |
|  |  |  |  | **regulation of hormone levels** | **auxin transport, hormone transport** |
|  |  |  | **oxylipin biosynthetic process, oxylipin metabolic process, jasmonic acid metabolic process, jasmonic acid biosynthetic process** |  | **oxylipin metabolic process, oxylipin biosynthetic, jasmonic acid metabolic process, jasmonic acid biosynthetic process** |
|  |  |  | **response to herbivore** |  |  |
|  |  |  | **polyol biosynthetic process** |  |  |
|  |  |  | **response to ethylene stimulus** |  |  |
|  |  |  |  |  |  |
|  |  | **ATP binding, identical protein binding, protein binding** |  | **4 iron, 4 sulfur cluster binding** |  |
|  | **zinc ion binding** | **small protein activating enzyme activity** |  | **ferric iron binding, cation binding**  **ion binding, metal cluster binding**  **iron-sulfur cluster binding, calmodulin binding, chitin binding, protein phosphorylated amino acid binding, metal ion binding** |  |
|  | **protein polymerization, protein complex binding** |  |  | **cellular protein complex assembly, protein binding** |  |
|  | **small conjugating protein binding** |  |  |  |  |
|  | **structural molecule activity** |  |  |  |  |
|  |  |  |  |  |  |

**1:** Enriched categories are shown in a light green background.

**2:** Depleted categories are shown in a light red background.
