# Supplementary material for: Overexpression of Grain Amaranth (Amaranthus hypochondriacus) AhERF or AhDOF Transcription Factors in Arabidopsis thaliana Increases Water Deficit- and Salt-Stress Tolerance, Respectively, via Contrasting Stress-Amelioration Mechanisms
Source: PLoS One. 2016 Oct 17;11(10):e0164280. doi: 10.1371/journal.pone.0164280 (PMC5066980; doi:10.1371/journal.pone.0164280)
Supplement: S7 Table — (DOCX) [file pone.0164280.s013.docx]

**S7 Table. GO categories found to be significantly modified in *AhDof-AI* overexpressing transgenic Arabidopsis plants in optimal conditions and under salt stress (SS).**

| **DOF OPTIMAL (+)** | **DOF OPTIMAL (-)** | **DOF SS (+)** | **DOF SS (-)** |
| --- | --- | --- | --- |
|  |  |  |  |
| regulation of nucleobase-containing compound metabolic process, nucleobase-containing compound metabolic process. | purine ribonucleotide biosynthetic process, purine nucleoside triphosphate biosynthetic process, purine ribonucleoside triphosphate biosynthetic process, ribonucleoside triphosphate biosynthetic process. | nucleobase-containing compound metabolic process, regulation of nucleobase-containing compound metabolic process, 5-carbamoylmethyluridine metabolic process. | purine nucleotide biosynthetic process. |
|  |  |  |  |
|  | pyrimidine nucleobase purine nucleobase, nucleobase, nucleobase-containing compound and uracil transmembrane transporter activity, nucleobase: cation symporter activity. |  |  |
|  |  |  |  |
|  | Copper, copper ion, substrate-specific, cation, secondary active, ion, phosphate ion, inorganic phosphate, tricarboxylic acid, glucose, monosaccharide, hexose , carbohydrate, sugar, , metal ion, transition metal ion, organic acid transport, transporter activity, and/ or transmembrane transporter activity.  Solute: cation symporter activity, symporter activity, transporting ATPase activity, solute: hydrogen symporter activity, sugar: hydrogen symporter activity, cation: sugar symporter activity. | Lipid transport | Copper ion, hydrogen, proton, ion, cation and monovalent inorganic,  monosaccharide and hexose transport, transport activity and/ or transmembrane transport. |
| Regulation of intracellular protein transport, protein import, protein localization to organelle, regulation of nucleocytoplasmic transport, iron chelate transmembrane transporter activity,  iron-nicotianamine transmembrane transporter activity | Vesicle-mediated transport, protein secretion.  Intracellular transport. | Protein targeting to chloroplast. | Protein import into nucleus, translocation. |
|  |  |  |  |
| biological adhesion, cell adhesion. |  | quercetin 7-O-glucosyltransferase activity. | coumarin biosynthetic process,  coumarin metabolic process. |
| lignin biosynthetic process |  | beta-mannosidase activity |  |
|  |  | UDP-N-acetylglucosamine diphosphorylase activity, UDP-N-acetylgalactosamine diphosphorylase activity |  |
|  |  |  |  |
|  |  | photoperiodism, , long-day photoperiodism |  |
|  |  | negative regulation of cell size |  |
|  |  | regulation of growth rate |  |
|  |  | organ boundary specification between lateral organs and the meristem, formation of anatomical boundary, formation of organ boundary, regionalization, organ development organ formation, organ morphogenesis |  |
|  |  |  |  |
|  |  | developmental process , regulation of meristem structural organization |  |
|  |  | pattern specification process, adaxial/abaxial pattern specification, axis specification, polarity specification of adaxial/abaxial axis, specification of axis polarity, adaxial/abaxial axis specification. |  |
|  |  |  |  |
|  |  | Flowering photoperiodism, maintenance of inflorescence meristem identity, long-day photoperiodism, flowering, vegetative to reproductive phase transition of meristem, reproductive structure development, flower development |  |
| Cellular process involved in reproduction, regulation of meiosis I, regulation of reciprocal meiotic recombination, negative regulation of reciprocal meiotic recombination, negative regulation of meiosis. |  | Developmental process involved in reproduction, reproduction, reproductive process, floral organ development, floral whorl development. |  |
|  |  |  |  |
|  |  | anatomical structure development |  |
|  |  | multicellular organismal development, multicellular organismal process |  |
|  |  | system development |  |
|  |  |  |  |
|  |  | post-embryonic development, post-embryonic organ development | embryo sac development |
|  |  |  | De-etiolation |
|  |  |  |  |
| organelle organization |  |  |  |
|  | Localization, establishment of localization, establishment of localization in cell, cellular localization |  | Establishment of localization |
|  | cellular component assembly |  |  |
|  | shoot development, shoot system development, shoot morphogenesis |  |  |
|  | cytokinesis by cell plate formation, cell cycle cytokinesis |  |  |
|  |  |  |  |
|  | Cellular macromolecular complex subunit organization, cellular macromolecular complex assembly, macromolecular complex assembly, macromolecular complex subunit organization |  | Macromolecular complex subunit organization, macromolecular complex assembly, cellular macromolecular complex subunit organization, cellular macromolecular complex assembly, cellular component assembly |
|  |  |  | cellular macromolecule catabolic process |
| lytic vacuole organization | vacuole organization | lytic vacuole organization |  |
| cellular component organization | cellular component biogenesis, cellular component |  |  |
| mitochondrion organization |  |  |  |
| membrane organization, cellular membrane organization. |  |  |  |
|  |  |  |  |
|  | cofactor metabolic process, cofactor biosynthetic |  |  |
|  |  |  |  |
|  | coenzyme biosynthetic process, coenzyme metabolic process |  |  |
|  | acetyl-CoA metabolic process, acetyl-CoA biosynthetic process |  |  |
|  | organic substance metabolic process |  |  |
|  | small molecule metabolic process |  |  |
| regulation of cellular process, regulation of metabolic process, regulation of cellular metabolic process, regulation of cellular biosynthetic process | cellular process | regulation of cellular process, regulation of cellular metabolic process, regulation of metabolic process, regulation of cellular biosynthetic process |  |
| regulation of biological quality, regulation of biological process, biological regulation |  | regulation of biological process, biological regulation |  |
| regulation of macromolecule metabolic process, regulation of macromolecule biosynthetic process |  | regulation of macromolecule metabolic process, regulation of macromolecule biosynthetic process |  |
|  |  |  |  |
| regulation of biosynthetic process |  | regulation of biosynthetic process |  |
| regulation of primary metabolic process |  | regulation of primary metabolic process |  |
|  |  |  |  |
| lipid binding , omega-6 fatty acid desaturase activity | lipid biosynthetic process  cellular lipid metabolic process, lipid metabolic process, |  | lipid metabolic process, lipid metabolic process, |
|  | fatty acid catabolic process, fatty acid oxidation, lipid oxidation, fatty acid beta-oxidation |  | regulation of fatty acid metabolic process |
|  | phosphatidylglycerol biosynthetic process, phosphatidylglycerol metabolic process |  | phosphatidylglycerol biosynthetic process, phosphatidylglycerol metabolic process |
|  |  |  | glycerophospholipid biosynthetic process, glycerophospholipid metabolic process |
| phospholipase activity | phospholipid biosynthetic process |  |  |
|  | phosphatidylinositol metabolic process, phosphatidylinositol biosynthetic process |  | phosphatidylinositol metabolic process, phosphatidylinositol biosynthetic process |
|  |  |  | small molecule biosynthetic process |
|  |  |  |  |
| polyamine catabolic process, polyamine metabolic process |  | regulation of nitrogen compound metabolic process |  |
| regulation of nitrogen compound metabolic process, nitrogen compound metabolic process |  |  |  |
| cellular biogenic amine catabolic process, cellular biogenic amine metabolic process |  |  |  |
|  |  | intramolecular transferase activity, transferring amino groups |  |
|  | cellular amide metabolic process |  |  |
|  |  |  |  |
|  | hexose catabolic process |  |  |
|  | monosaccharide catabolic process |  |  |
|  | monosaccharide metabolic process, hexose metabolic process, glucose metabolic process |  | glucose metabolic process, carbohydrate biosynthetic process |
|  |  |  |  |
|  | glycerolipid biosynthetic, glycerophospholipid biosynthetic process, glycerophospholipid metabolic process |  | glycerolipid biosynthetic process, glycerolipid metabolic process |
|  |  |  | macromolecule catabolic process |
|  |  |  |  |
| riboflavin synthase activity | vitamin biosynthetic process, pyridoxal phosphate metabolic process, pyridoxal phosphate biosynthetic process |  | vitamin biosynthetic process, water-soluble vitamin biosynthetic process, water-soluble vitamin metabolic process, thiamine-containing compound biosynthetic process, thiamine-containing compound metabolic process, thiamine metabolic process |
|  |  |  |  |
| sinapoyltransferase activity |  | sinapoyltransferase activity |  |
|  | isoprenoid metabolic process, isoprenoid biosynthetic process |  |  |
| oxysterol binding, sterol binding, steroid binding | sterol metabolic process, steroid biosynthetic process, steroid metabolic process, sterol biosynthetic process |  |  |
|  | pentacyclic triterpenoid metabolic process, pentacyclic triterpenoid biosynthetic process, triterpenoid biosynthetic process, triterpenoid metabolic process |  |  |
|  |  |  |  |
| monoterpene biosynthetic process, monoterpene metabolic process | terpenoid metabolic process, terpenoid biosynthetic process |  |  |
|  |  |  |  |
| glycogen metabolic process | glucan biosynthetic process | glycogen metabolic process |  |
|  |  |  | starch biosynthetic process |
|  | monocarboxylic acid metabolic process |  | maltose metabolic process  hexose metabolic process, hexose biosynthetic process disaccharide metabolic process  oligosaccharide metabolic process  dTDP-rhamnose metabolic process, monosaccharide metabolic process, monosaccharide biosynthetic process |
|  |  |  | dTDP-rhamnose biosynthetic process  cellular carbohydrate biosynthetic process, rhamnose metabolic process rhamnose biosynthetic process |
|  |  |  | hexose catabolic process  monosaccharide catabolic process |
|  |  |  |  |
| cellular modified amino acid catabolic process | aspartate family amino acid catabolic process |  | branched-chain amino acid biosynthetic process, branched-chain amino acid metabolic process |
|  |  |  |  |
| farnesyltranstransferase activity, farnesyl-diphosphate farnesyltransferase activity | glycoprotein metabolic process, glycoprotein biosynthetic process | UDP-N-acetylgalactosamine metabolic process |  |
|  | macromolecule glycosylation, protein glycosylation, glycosylation |  |  |
|  |  |  |  |
|  |  |  | post-translational protein modification, protein refolding |
|  |  |  |  |
|  | ribonucleoprotein complex assembly, ribonucleoprotein complex biogenesis |  | ribonucleoprotein complex assembly |
|  |  |  |  |
| exonuclease activity |  |  |  |
|  | DNA repair, response to DNA damage stimulus |  |  |
|  | postreplication repair |  | postreplication repair |
|  | DNA conformation change |  |  |
|  | ATP-dependent 3'-5' DNA helicase activity, 3'-5' DNA helicase activity |  |  |
|  |  |  |  |
| DNA replication initiation |  |  |  |
| DNA duplex unwinding |  |  |  |
| DNA geometric change |  |  |  |
| DNA unwinding involved in replication |  |  |  |
|  |  |  |  |
|  | Spliceosomal complex assembly |  | Spliceosomal complex assembly |
|  | 7-methylguanosine RNA capping |  |  |
|  | nuclear-transcribed mRNA catabolic process, deadenylation-independent decay |  |  |
|  | deadenylation-independent decapping of nuclear-transcribed mRNA |  |  |
|  |  |  | RNA splicing, mRNA splicing, via spliceosome |
|  |  |  | miRNA catabolic process |
|  |  |  | transcription initiation from RNA polymerase II promoter |
|  |  |  | miRNA metabolic process |
|  |  |  | ncRNA catabolic process |
|  |  |  | RNA splicing, via transesterification reactions with bulged adenosine as nucleophile |
|  |  |  | RNA splicing, via transesterification reactions |
|  |  |  | mRNA metabolic process |
|  |  |  |  |
| RNA biosynthetic process, RNA metabolic process, regulation of RNA metabolic process |  | RNA biosynthetic process, RNA metabolic process, regulation of RNA metabolic process, RNA processing |  |
|  |  |  |  |
| regulation of chromosome organization, chromosome organization |  |  |  |
|  | chromatin assembly, chromatin organization | ATP-dependent chromatin remodeling |  |
| histone acetylation |  | methylated histone residue binding |  |
| DNA methylation, DNA alkylation, regulation of histone H4 acetylation, DNA modification |  | regulation of gene expression, epigenetic |  |
| DNA-directed RNA polymerase activity |  |  |  |
| RNA polymerase activity |  | gene silencing by miRNA |  |
|  |  | regulation of gene expression, regulation of transcription, DNA-dependent |  |
|  |  | posttranscriptional gene silencing by RNA |  |
|  |  | posttranscriptional gene silencing |  |
| transcription, DNA-dependent, regulation of transcription, DNA-dependent |  | transcription, DNA-dependent |  |
| sequence-specific DNA binding transcription factor activity |  | sequence-specific DNA binding transcription factor activity |  |
| transcription regulator activity |  | transcription regulator activity |  |
| regulation of gene expression |  |  |  |
| DNA binding |  | DNA binding |  |
|  |  | 3'(2'),5'-bisphosphate nucleotidase activity  nucleotidase activity |  |
| isoleucyl-tRNA aminoacylation |  | isoleucyl-tRNA aminoacylation |  |
|  |  |  |  |
|  | response to abiotic stimulus |  |  |
|  | response to stimulus |  | cellular response to stimulus |
|  |  |  |  |
|  | response to blue light, response to red light, response to radiation, response to light stimulus, cellular response to red light, response to red or far red light, response to far red light, response to light intensity, response to low light intensity stimulus, cellular response to blue light, red light signaling pathway  organization, cellular response to radiation, cellular response to light stimulus |  | response to red light, red light signaling pathway, cellular response to red light |
|  |  |  |  |
|  | pigment accumulation in response to UV light, pigmentation, pigment accumulation in tissues in response to UV light, pigment accumulation in tissue, anthocyanin accumulation in tissues in response to UV light, pigment accumulation |  |  |
|  |  |  |  |
|  |  |  | response to protein stimulus |
|  |  | response to zinc ion |  |
| response to fructose stimulus |  |  |  |
| zinc ion binding, calcium ion binding | response to cadmium ion |  |  |
|  | response to inorganic substance |  |  |
|  | response to temperature stimulus |  |  |
|  |  |  |  |
|  | response to stress |  |  |
|  | cellular response to stress |  | cellular response to stress |
|  | phagocytosis |  |  |
|  | response to metal ion |  |  |
|  | signal transducer activity  molecular transducer activity |  |  |
|  | receptor signaling protein activity, receptor signaling protein |  |  |
|  |  |  | cellular response to chemical stimulus |
|  |  |  | detection of external stimulus |
|  |  |  | cellular response to phosphate starvation |
|  |  |  |  |
|  | stomatal complex morphogenesis, stomatal complex development |  | stomatal complex development, stomatal complex morphogenesis |
|  |  |  |  |
|  |  | response to xenobiotic stimulus |  |
|  |  | response to biotic stimulus |  |
|  |  | response to flooding |  |
|  |  | regulation of nitric oxide metabolic process |  |
|  | response to salicylic acid stimulus |  | systemic acquired resistance, salicylic acid mediated signaling pathway, response to salicylic acid stimulus  cellular protein catabolic process |
|  |  |  | abscisic acid mediated signaling pathway, cellular response to abscisic acid stimulus |
|  |  |  | response to 1-aminocyclopropane-1-carboxylic acid, response to ethylene stimulus |
|  |  | positive regulation of reactive oxygen species metabolic process | reactive oxygen species metabolic process, response to hydrogen peroxide, response to reactive oxygen species, cellular response to oxidative stress |
|  |  |  | response to salt stress |
|  |  |  | response to osmotic stress |
|  |  |  | polyol biosynthetic process |
|  |  |  | hormone-mediated signaling pathway |
|  | gibberellin 3-beta-dioxygenase activity |  |  |
|  | IAA-amino acid conjugate hydrolase activity |  |  |
|  |  |  |  |
|  | MAP kinase activity, serine/threonine kinase activity |  | MAPK cascade |
|  |  | tyrosine decarboxylase activity | protein dephosphorylation |
| AMP-activated protein kinase activity |  |  |  |
| protein kinase C binding |  |  |  |
|  |  |  |  |
| GTPase activator activity | G-protein coupled receptor activity |  |  |
| Ras GTPase activator activity, Ran GTPase activator activity |  |  |  |
|  |  |  |  |
| homeostatic process | copper ion homeostasis cellular copper ion homeostasis, cellular di-, tri-valent inorganic cation homeostasis |  |  |
|  |  |  |  |
|  |  |  |  |
|  | alcohol metabolic process |  | alcohol biosynthetic process  alcohol metabolic process |
|  |  |  |  |
|  |  |  | pyruvate metabolic process |
|  |  |  | gluconeogenesis |
| mitochondrial respiratory chain complex assembly, energy reserve metabolic process, photosystem II oxygen evolving complex assembly | carbon fixation, electron transport chain, ATP biosynthetic process, ribulose-bisphosphate carboxylase activity (RUBISCO), 3-deoxy-7-phosphoheptulonate synthase activity (Calvin cycle) | energy reserve metabolic process, mitochondrial respiratory chain complex assembly | mitochondrial electron transport, NADH to ubiquinone |
| Pentose pathway: 5-phosphoribose 1-diphosphate metabolic process, ribose phosphate biosynthetic process, 5-phosphoribose 1-diphosphate biosynthetic process, ribose phosphate metabolic process, D-ribose biosynthetic process | pentose-phosphate shunt, NADPH regeneration, NADP metabolic process |  |  |
|  | cytochrome b6f complex assembly |  |  |
|  | quinone cofactor metabolic process, quinone cofactor biosynthetic process |  |  |
|  | nicotinamide nucleotide metabolic process, nicotinamide metabolic process |  |  |
|  | nucleoside triphosphate biosynthetic process |  |  |
|  |  |  | iron-sulfur cluster assembly, metallo-sulfur cluster assembly |
|  |  | glutamate-1-semialdehyde 2,1-aminomutase activity (chlorophyll synthesis) |  |
|  |  | photorespiration: glycine:2-oxoglutarate aminotransferase activity |  |
|  |  |  |  |
|  | AMP binding, adenyl nucleotide binding  purine nucleoside binding, copper ion binding, nucleoside binding, ATP binding, adenyl ribonucleotide binding |  |  |
|  |  |  |  |
|  | oxidoreductase activity, acting on the CH-CH group of donors, oxidoreductase activity, acting on paired donors, with incorporation or reduction of molecular oxygen, 2-oxoglutarate as one donor, and incorporation of one atom each of oxygen into both donors |  |  |
|  |  |  |  |
|  |  | selenium binding |  |
|  |  |  |  |
| transferase activity |  | structural molecule activity  2'-phosphotransferase activity  tRNA 2'-phosphotransferase activity |  |
|  |  |  |  |
|  |  |  |  |
