# Supplementary material for: Overexpression of Grain Amaranth (Amaranthus hypochondriacus) AhERF or AhDOF Transcription Factors in Arabidopsis thaliana Increases Water Deficit- and Salt-Stress Tolerance, Respectively, via Contrasting Stress-Amelioration Mechanisms
Source: PLoS One. 2016 Oct 17;11(10):e0164280. doi: 10.1371/journal.pone.0164280 (PMC5066980; doi:10.1371/journal.pone.0164280)
Supplement: S8 Table — (DOCX) [file pone.0164280.s014.docx]

**S8 Table**. **Modified mMicroRNA gene expression.** MicroRNA genes whose expression was up- (U)or down-regulated (D) in (**A**) OE- *AHERF-VII* or OE-*AhDof-AI* transgenic plants maintained in optimal growing conditions (Op), subjected to water-deficit (WS) or salt (SS) stress, or undergoing recovery (R) after WS.

| **ERF-OE U-Op** | **ERF-OE D-Op** | **ERF-OE U-WS** | **ERF-OE D-WS** | **ERF-OE U-R** | **ERF-OE D-R** |
| --- | --- | --- | --- | --- | --- |
| MIR166D^1^ | MIR395B | MIR398C | MIR393A | MIR164C | MIR399C |
| MIR164B | MIR395A | MIR164C | MIR395B |  | MIR393A |
|  |  |  |  |  | MIR395B |
|  |  |  |  |  | MIR169C |
|  |  |  |  |  | MIR165B |
|  |  |  |  |  | MIR393B |
|  |  |  |  |  |  |

| **Dof-OE U-Op** | **Dof-OE D-Op** | **Dof-OE U-SS** | **Dof-OE D-SS** |
| --- | --- | --- | --- |
| MIR166D | MIR395B^2^ | MIR156F | MIR393A |
| MIR398C | MIR395E | MIR171A | MIR395B |
|  | MIR168B |  | MIR395E |
|  |  |  |  |

^1^Encodes a microRNA that targets several HD-ZIPIII family members including PHV, PHB, REV, ATHB-8, and ATHB-15.

HOMEODOMAIN LEUCINE ZIPPER CLASS III (HD-Zip III) proteins represent a group of transcription factors that have been extensively implicated in the regulation of primary and secondary vascular tissue pattern formation, as well as lateral organ and cambial polarity in herbaceous annual plants.

This gene family regulates apical embryo patterning, embryonic shoot meristem formation, organ polarity, vascular development, and meristem function.

^2^395B: Encodes a microRNA that targets both APS (ATP sulfurylase [APS]) and AST (involved in anthocyanin biosynthesis OR asparagine synthetase-1) family members. Cellular response to phosphate starvation, cellular response to sulfate starvation, cellular response to sulfur starvation

^3^393A: Encodes a microRNA that targets several TIR1/AFB family members and one bHLH family member. Auxin-activated signaling pathway, cellular response to nitrate.
